# From vision toward best practices: Evaluating *in vitro* transcriptomic points of departure for application in risk assessment using a uniform workflow

**DOI:** 10.3389/ftox.2023.1194895

**Published:** 2023-05-23

**Authors:** Anthony J. F. Reardon, Reza Farmahin, Andrew Williams, Matthew J. Meier, Gregory C. Addicks, Carole L. Yauk, Geronimo Matteo, Ella Atlas, Joshua Harrill, Logan J. Everett, Imran Shah, Richard Judson, Sreenivasa Ramaiahgari, Stephen S. Ferguson, Tara S. Barton-Maclaren

**Affiliations:** ^1^ Existing Substances Risk Assessment Bureau, Healthy Environments and Consumer Safety Branch, Health Canada, Ottawa, ON, Canada; ^2^ Environmental Health Science and Research Bureau, Healthy Environments and Consumer Safety Branch, Health Canada, Ottawa, ON, Canada; ^3^ Department of Biology, University of Ottawa, Ottawa, ON, Canada; ^4^ Department of Biochemistry, University of Ottawa, Ottawa, ON, Canada; ^5^ Center for Computational Toxicology and Exposure, US Environmental Protection Agency, Durham, NC, United States; ^6^ Division of Translational Toxicology, Mechanistic Toxicology Branch, National Institute of Environmental Health Sciences, National Institutes of Health, Durham, NC, United States

**Keywords:** new approach methods, NAMs, transcriptomics, benchmark dose (BMD) modeling, *in vitro* to *in vivo* extrapolation (IVIVE), chemical safety

## Abstract

The growing number of chemicals in the current consumer and industrial markets presents a major challenge for regulatory programs faced with the need to assess the potential risks they pose to human and ecological health. The increasing demand for hazard and risk assessment of chemicals currently exceeds the capacity to produce the toxicity data necessary for regulatory decision making, and the applied data is commonly generated using traditional approaches with animal models that have limited context in terms of human relevance. This scenario provides the opportunity to implement novel, more efficient strategies for risk assessment purposes. This study aims to increase confidence in the implementation of new approach methods in a risk assessment context by using a parallel analysis to identify data gaps in current experimental designs, reveal the limitations of common approaches deriving transcriptomic points of departure, and demonstrate the strengths in using high-throughput transcriptomics (HTTr) to derive practical endpoints. A uniform workflow was applied across six curated gene expression datasets from concentration-response studies containing 117 diverse chemicals, three cell types, and a range of exposure durations, to determine tPODs based on gene expression profiles. After benchmark concentration modeling, a range of approaches was used to determine consistent and reliable tPODs. High-throughput toxicokinetics were employed to translate *in vitro* tPODs (µM) to human-relevant administered equivalent doses (AEDs, mg/kg-bw/day). The tPODs from most chemicals had AEDs that were lower (i.e., more conservative) than apical PODs in the US EPA CompTox chemical dashboard, suggesting *in vitro* tPODs would be protective of potential effects on human health. An assessment of multiple data points for single chemicals revealed that longer exposure duration and varied cell culture systems (e.g., 3D vs. 2D) lead to a decreased tPOD value that indicated increased chemical potency. Seven chemicals were flagged as outliers when comparing the ratio of tPOD to traditional POD, thus indicating they require further assessment to better understand their hazard potential. Our findings build confidence in the use of tPODs but also reveal data gaps that must be addressed prior to their adoption to support risk assessment applications.

## 1 Introduction

Every year, new substances are introduced into the global marketplace with limited toxicity information, while presently registered chemicals that are existing on the market are continually used and re-purposed into a myriad of products available to consumers. Thus, higher-throughput techniques are required to effectively contextualize and predict the hazard potential posed by chemicals to facilitate risk assessment activities. A large number of substances, including but not limited to, those on Canada’s Domestic Substances List, present a challenge for chemicals management programs, particularly when inadequate data are available. Historically, data-driven assessments used animal (principally rodent) models from traditional standardized protocols designed to address acute (immediate effects from single dose), as well as sub-chronic and chronic (longer-term effects from repeated dose) toxicological assessments (Collins et al., 2008; Barile, 2013). Regulatory agencies, including those within Canada, are taking steps to implement new approach methods (NAMs) and evolving scientific approaches to address the limitations of traditional methods (e.g., hindrance on data generation as a result of time, cost, and labour-intensive practices) as well as ethical concerns of animal use, as part of the paradigm shift to next-generation risk assessment strategies (Bhuller et al., 2021).

Transcriptomics provides a high-throughput means of producing large datasets covering a broad range of molecular responses to potentially hazardous substances. Transcriptomic technologies have been in use for over two decades in molecular biology and recent innovations have enhanced their specificity and dynamic range to enable implementation in risk assessment activities. Application of transcriptomics for understanding toxicology (toxicogenomics) includes the analysis and interpretation of changes in gene expression caused by exposure to potentially hazardous substances to explain their prospective adverse effects (Chepelev et al., 2015; Moffat et al., 2015; Farmahin et al., 2017; Johnson et al., 2020; Krewski et al., 2020). High-throughput transcriptomics (HTTr) enables rapid evaluation of global changes in gene expression profiles in cell culture models to identify potential chemical toxicities (Harrill et al., 2021). Efficient use of HTTr is supported by the availability of computational pipelines that process large transcriptomic datasets and can be uniformly applied across numerous chemicals and exposure levels (Mezencev and Subramaniam, 2019; Verheijen et al., 2022). Benchmark dose (BMD) modeling has been applied to derive transcriptomic points of departure (tPODs) in a manner, that is, analogous to the production and application of apical PODs using traditional approaches (Thomas et al., 2013; Moffat et al., 2015; Webster et al., 2015; Farmahin et al., 2017). Given the involvement of gene expression pathways in cellular regulation, transcriptomic changes may provide opportunities for inference to a variety of contexts related to risk assessment. This includes quantifying potency, informing mode of action/mechanistic information, and characterizing adverse effects that support weight of evidence approaches to evaluate chemicals (North and Vulpe, 2010; Bourdon-Lacombe et al., 2015).

Recently, a logic framework was proposed to examine the potential to develop and apply transcriptomic methods to refine, or even replace, the current risk assessment paradigm that relies on traditional apical PODs (Johnson et al., 2022). This framework supports the current shift away from the identification of specific critical effect endpoints in animal models toward establishing conservative tPODs that are sufficiently protective to meet the contemporary needs of regulatory agencies (Stucki et al., 2022). This logic framework aligns with current efforts to facilitate efficient chemical screening using *in vitro* HTTr.

The aim of the current work was to demonstrate the capability of HTTr to derive protective tPODs by applying a uniform analysis across a diverse chemical space, building on the foundation of approaches from previous works deriving tPODs using various experimental designs (Farmahin et al., 2017; Pagé-Larivière et al., 2019; Ramaiahgari et al., 2019; Thomas, 2019; Ewald et al., 2022). This study was conducted for the purpose of building confidence in the application of these technologies by revealing the associated uncertainty and potential variability that corresponds to *in vitro* POD derivation using transcriptomic data. To achieve this, *in vitro* tPODs were compared to apical endpoints by employing *in vitro* to *in vivo* extrapolation (IVIVE) to generate an administered equivalent dose (AED). More specifically, we used high-throughput toxicokinetic (httk) modeling to translate tPODs (µM) to AEDs (mg/kg-bw/day). The AED provides a valuable endpoint to determine the human relevance of tPODs for the purpose of chemical prioritization and/or screening level risk assessment. They also provide insight into the applicability of these, and similar NAM-based approaches, as potential endpoints for human health risk assessment. This work supports regulatory initiatives in the efforts to establish best practices and scientific confidence in the use of NAMs to produce protective human health relevant thresholds further inspiring the shift toward the reduction and replacement of animals for toxicity testing (van der Zalm et al., 2022).

## 2 Materials and methods

### 2.1 Study design

**FIGURE 1:**
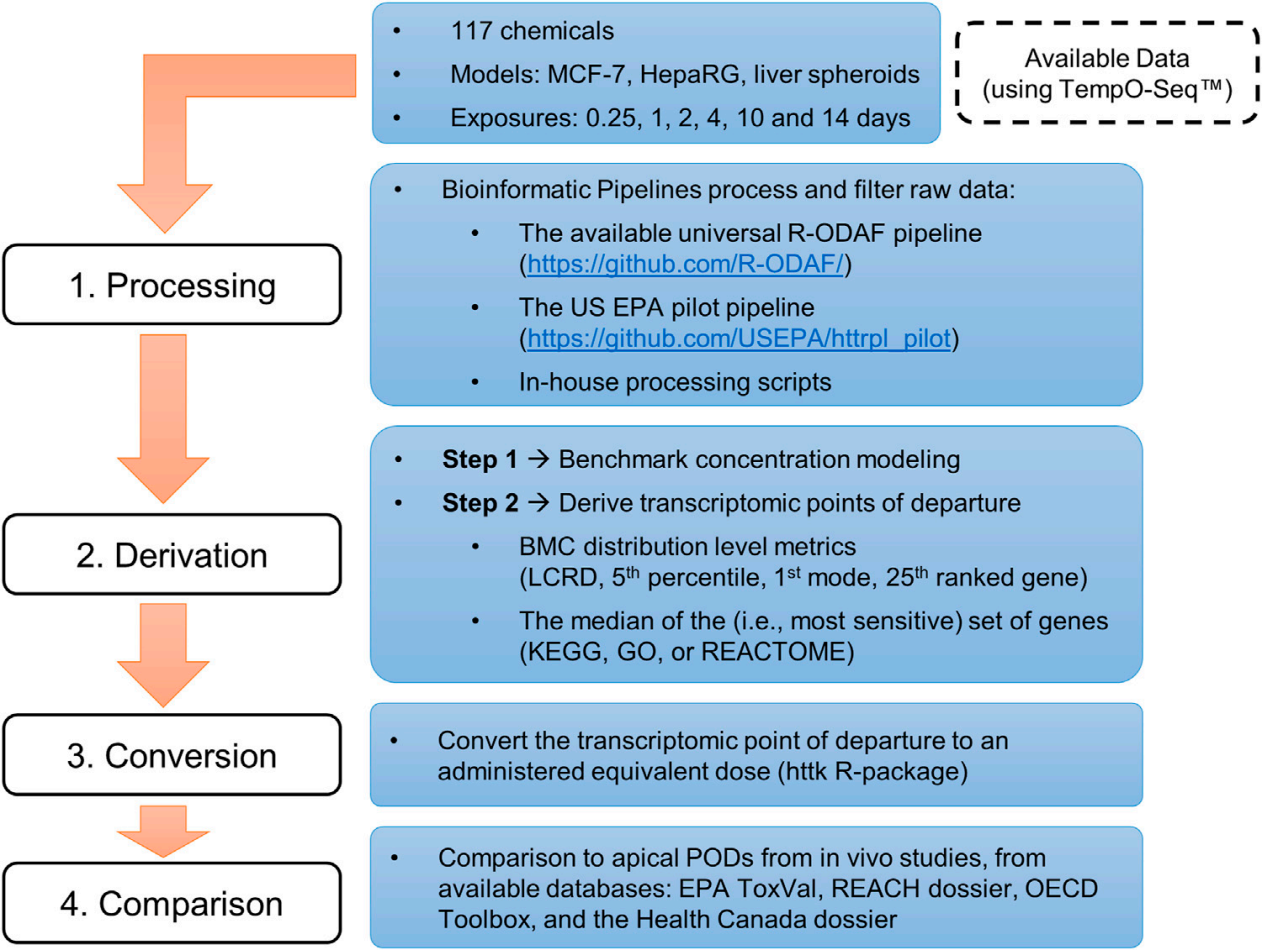
Overview of the workflow used to compile available datasets and derive *in vitro* points of departure for comparison with *in vivo* apical points of departure from curated databases.

This investigation used data from multiple studies that were selected based on availability (i.e., published studies using multiple, publicly available transcriptomic datasets), specifically *in vitro* HTTr data analyzed using the TempO-Seq™ platform (BioSpyder Technologies, Inc., Carlsbad, CA), and refined using a bioinformatic pipeline. Study selection resulted in data for a total of 179 concentration-response experiments of various experimental designs, spanning 117 chemicals. An overview of these datasets is presented in Table 1 and a detailed list of chemicals and experiments is available in the Supplementary Appendix S1. Further details on chemical preparation, working solutions, cell cultures and exposure, details on TempO-Seq library building, and an overview of QA/QC protocols with removal of designated outliers are available in the original publications (Ramaiahgari et al., 2019; Buick et al., 2021; Harrill et al., 2021; Reardon et al., 2021; Rowan-Carroll et al., 2021; Matteo et al., 2022). tPODs were derived using a variety of approaches as described below. The tPODs were subject to IVIVE using a widely available program (httk R-package, v2.2.1) to account for the large scope of chemicals (Pearce et al., 2017) before comparison with high quality data extracted from commonly used regulatory databases (i.e., data from previous assessments using traditionally-derived PODs). An overview of the steps from collection of raw/processed data to the final comparison of AEDs and apical PODs is detailed below and depicted in a schematic workflow (Figure 1).

**TABLE 1:** List of chemicals with available *in vitro* datasets.

Dataset	Chemical Name	Abbreviated Name	CASRN	Model(s)	Exposure (Days)
OECD 2022	2-(4-hydroxyphenyl)sulfonylphenol	2,4-BPS	5397-34-2	MCF-7	2
OECD 2022	2-[(4-Hydroxyphenyl)methyl]phenol	2,4-BPF	2467-03-0	MCF-7	2
OECD 2022	2-[4-(Benzyloxy)benzene-1-sulfonyl]phenol	BPS-MPE	63134-33-8	MCF-7	2
OECD 2022	4-(4-hydroxyphenyl)sulfonylphenol	4,4-BPS	80-09-1	MCF-7	2
OECD 2022	4-[(4-hydroxyphenyl)methyl]phenol	4,4-BPF	620-92-8	MCF-7	2
OECD 2022	4-((4-Isopropoxyphenyl)sulfonyl)phenol	D8	95235-30-6	MCF-7	2
OECD 2022	4,4'-Bis(p-tolylsulfonylureido)diphenylmethane	BTUM	151882-81-4	MCF-7	2
OECD 2022	4,4'-Sulfonylbis[2-(prop-2-en-1-yl)phenol]	TGSA	41481-66-7	MCF-7	2
OECD 2022	4-{4-[(Prop-1-en-2-yl)oxy]benzene-1-sulfonyl}phenol	BPS-MAE	97042-18-7	MCF-7	2
OECD 2022	Bis (4-chorophenyl) Sulfone	Bis4CPS	80-07-9	MCF-7	2
OECD 2022	Bisphenol A	BPA	80-05-7	MCF-7	2
OECD 2022	Bisphenol A diglycidyl ether	BADGE	1675-54-3	MCF-7	2
OECD 2022	Bisphenol AF	BPAF	1478-61-1	MCF-7	2
OECD 2022	Bisphenol AP	BPAP	1571-75-1	MCF-7	2
OECD 2022	Bisphenol C	BPC	14868-03-2	MCF-7	2
OECD 2022	Dexamethasone	Dex	50-02-2	MCF-7	2
OECD 2022	Β-Estradiol	Estradiol	50-28-2	MCF-7	2
OECD 2022	Pergafast 201	Perg201	232938-43-1	MCF-7	2
Harrill et al.	3,5,3'-Triiodothyronine	Triiodothyronine	6893-02-3	MCF-7	0.25
Harrill et al.	4-Cumylphenol		599-64-4	MCF-7	0.25
Harrill et al.	4-Hydroxytamoxifen		68392-35-8	MCF-7	0.25
Harrill et al.	4-Nonylphenol (branched)	4-Nonylphenol	84852-15-3	MCF-7	0.25
Harrill et al.	Amiodarone hydrochloride	Amiodarone HCl	19774-82-4	MCF-7	0.25
Harrill et al.	Atrazine		1912-24-9	MCF-7	0.25
Harrill et al.	Bifenthrin		82657-04-3	MCF-7	0.25
Harrill et al.	Bisphenol A	BPA	80-05-7	MCF-7	0.25
Harrill et al.	Bisphenol B	BPB	77-40-7	MCF-7	0.25
Harrill et al.	Butafenacil		134605-64-4	MCF-7	0.25
Harrill et al.	Cladribine		4291-63-8	MCF-7	0.25
Harrill et al.	Clofibrate		637-07-0	MCF-7	0.25
Harrill et al.	Clomiphene citrate (1:1)	Clomiphene Cit	50-41-9	MCF-7	0.25
Harrill et al.	Cyanazine		21725-46-2	MCF-7	0.25
Harrill et al.	Cycloheximide		66-81-9	MCF-7	0.25
Harrill et al.	Cypermethrin		52315-07-8	MCF-7	0.25
Harrill et al.	Cyproconazole		94361-06-5	MCF-7	0.25
Harrill et al.	Cyproterone acetate	Cyproterone Ace	427-51-0	MCF-7	0.25
Harrill et al.	Farglitazar		196808-45-4	MCF-7	0.25
Harrill et al.	Fenofibrate		49562-28-9	MCF-7	0.25
Harrill et al.	Fenpyroximate (Z,E)		111812-58-9	MCF-7	0.25
Harrill et al.	Flutamide		13311-84-7	MCF-7	0.25
Harrill et al.	Fomesafen		72178-02-0	MCF-7	0.25
Harrill et al.	Fulvestrant		129453-61-8	MCF-7	0.25
Harrill et al.	Genistein		446-72-0	MCF-7	0.25
Harrill et al.	Imazalil		35554-44-0	MCF-7	0.25
Harrill et al.	Lactofen		77501-63-4	MCF-7	0.25
Harrill et al.	Lovastatin		75330-75-5	MCF-7	0.25
Harrill et al.	Maneb		12427-38-2	MCF-7	0.25
Harrill et al.	Nilutamide		63612-50-0	MCF-7	0.25
Harrill et al.	Perfluorooctanesulfonic acid	PFOS	1763-23-1	MCF-7	0.25
Harrill et al.	Perfluorooctanoic acid	PFOA	335-67-1	MCF-7	0.25
Harrill et al.	Prochloraz		67747-09-5	MCF-7	0.25
Harrill et al.	Propiconazole		60207-90-1	MCF-7	0.25
Harrill et al.	Pyraclostrobin		175013-18-0	MCF-7	0.25
Harrill et al.	Reserpine		50-55-5	MCF-7	0.25
Harrill et al.	Rotenone		83-79-4	MCF-7	0.25
Harrill et al.	Simazine		122-34-9	MCF-7	0.25
Harrill et al.	Simvastatin		79902-63-9	MCF-7	0.25
Harrill et al.	Sirolimus		53123-88-9	MCF-7	0.25
Harrill et al.	Tetrac		67-30-1	MCF-7	0.25
Harrill et al.	Thiram		137-26-8	MCF-7	0.25
Harrill et al.	Trichostatin A		58880-19-6	MCF-7	0.25
Harrill et al.	Trifloxystrobin		141517-21-7	MCF-7	0.25
Harrill et al.	Troglitazone		97322-87-7	MCF-7	0.25
Harrill et al.	Vinclozolin		50471-44-8	MCF-7	0.25
Harrill et al.	Ziram		137-30-4	MCF-7	0.25
Ramaiahgari et al.	Acetaminophen	APAP	103-90-2	HepaRG	4
Ramaiahgari et al.	Aflatoxin B1	AFB1	1162-65-8	HepaRG	4
Ramaiahgari et al.	Aspirin		50-78-2	HepaRG	4
Ramaiahgari et al.	Benzo(a)pyrene	B[a]P	50-32-8	HepaRG	4
Ramaiahgari et al.	Caffeine		58-08-2	HepaRG	4
Ramaiahgari et al.	Chenodeoxycholic acid	CDCA	474-25-9	HepaRG	4
Ramaiahgari et al.	Chlorpromazine	CPZ	50-53-3	HepaRG	4
Ramaiahgari et al.	Cyclophosphamide monohydrate	Cyclophosphamide	6055-19-2	HepaRG	4
Ramaiahgari et al.	Diphenhydramine hydrochloride	Diphenhydramine	147-24-0	HepaRG	4
Ramaiahgari et al.	Fenofibric acid	FFA	42017-89-0	HepaRG	4
Ramaiahgari et al.	Levofloxacin hydrate	Levofloxacin	138199-71-0	HepaRG	4
Ramaiahgari et al.	Menadione		58-27-5	HepaRG	4
Ramaiahgari et al.	N-Nitrosodimethylamine	DMN	62-75-9	HepaRG	4
Ramaiahgari et al.	Omeprazole	OMP	73590-58-6	HepaRG	4
Ramaiahgari et al.	Potassium chloride	KCl	7447-40-7	HepaRG	4
Ramaiahgari et al.	Rifampicin	RIF	13292-46-1	HepaRG	4
Ramaiahgari et al.	Ritonavir		155213-67-5	HepaRG	4
Ramaiahgari et al.	Rosiglitazone		122320-73-4	HepaRG	4
Ramaiahgari et al.	Sucrose		57-50-1	HepaRG	4
Ramaiahgari et al.	Tamoxifen		10540-29-1	HepaRG	4
Ramaiahgari et al.	Troglitazone		97322-87-7	HepaRG	4
Ramaiahgari et al.	Trovafloxacin mesylate	Trovafloxacin	147059-75-4	HepaRG	4
Ramaiahgari et al.	Valproic acid	VPA	99-66-1	HepaRG	4
Ramaiahgari et al.	Phenobarbital sodium	PB	57-30-7	HepaRG	4
PFAS	2H,2H,3H,3H-Perfluorooctanoic acid	5:3 Acid	914637-49-3	Spheroids	1, 10
PFAS	4:2 Fluorotelomer sulfonic acid	4:2 FtS	757124-72-4	Spheroids	1, 10
PFAS	6:2 Fluorotelomer alcohol	6:2 FtOH	647-42-7	Spheroids	1, 10
PFAS	6:2 Fluorotelomer phosphate monoester	6:2 monoPAP	57678-01-0	Spheroids	1, 10
PFAS	6:2 Fluorotelomer sulfonic acid	6:2 FtS	27619-97-2	Spheroids	1, 10
PFAS	8:2 Fluorotelomer alcohol	8:2 FtOH	678-39-7	Spheroids	1, 10
PFAS	8:2 Fluorotelomer phosphate monoester	8:2 monoPAP	57678-03-2	Spheroids	1, 10
PFAS	8:2 Fluorotelomer sulfonic acid	8:2 FtS	39108-34-4	Spheroids	1, 10
PFAS	Perfluorobutanesulfonic acid	PFBS	375-73-5	Spheroids	1, 4, 10, 14
PFAS	Perfluorobutanoic acid	PFBA	375-22-4	Spheroids	1, 10
PFAS	Perfluorodecanesulfonic acid	PFDS	335-77-3	Spheroids	1, 4, 10, 14
PFAS	Perfluorodecanoic acid	PFDA	335-76-2	Spheroids	1, 10
PFAS	Perfluoroheptanesulfonic acid	PFHpS	375-92-8	Spheroids	1, 10
PFAS	Perfluoroheptanoic acid	PFHpA	375-85-9	Spheroids	1, 10
PFAS	Perfluorohexanesulfonic acid	PFHxS	355-46-4	Spheroids	1, 10
PFAS	Perfluorohexanoic acid	PFHxA	307-24-4	Spheroids	1, 10
PFAS	Perfluorononanoic acid	PFNA	375-95-1	Spheroids	1, 10
PFAS	Perfluorooctanoic acid	PFOA	335-67-1	Spheroids	1, 4, 10, 14
PFAS	Perfluorooctanesulfonic acid	PFOS	1763-23-1	Spheroids	1, 4, 10, 14
PFAS	Perfluorooctanesulfonamide	PFOSA	754-91-6	Spheroids	1, 10
PFAS	Perfluoropentanoic acid	PFPeA	2706-90-3	Spheroids	1, 10
PFAS	Perfluorotetradecanoic acid	PFTeDA	376-06-7	Spheroids	1, 10
PFAS	Perfluoroundecanoic acid	PFUnA	2058-94-8	Spheroids	1, 10
Buick et al.	2-Deoxy-D-glucose	2DD-Glucose	154-17-6	HepaRG	2
Buick et al.	Aflatoxin B1	AFB1	1162-65-8	HepaRG	2
Buick et al.	Benzo(a)pyrene	B[a]P	50-32-8	HepaRG	2
Buick et al.	Cisplatin		15663-27-1	HepaRG	2
Buick et al.	Cyclophosphamide monohydrate	Cyclophosphamide	6055-19-2	HepaRG	2
Buick et al.	Cytosine arabinoside	Cyt arabinoside	147-94-4	HepaRG	2
Buick et al.	Eugenol		97-53-0	HepaRG	2
Buick et al.	Methyl methanesulfonate	M-mSulfonate	66-27-3	HepaRG	2
Buick et al.	N-Nitroso-N-ethylurea	N-Nitrosourea	759-73-9	HepaRG	2
Buick et al.	Propyl gallate		121-79-9	HepaRG	2
Buick et al.	Urea		57-13-6	HepaRG	2
Buick et al.	Zidovudine (azidothymidine)		30516-87-1	HepaRG	2

OECD 2022—Listing of Bisphenols from Health Canada OECD Case-study, Harrill et al.—Listing of chemicals from published dataset in Harrill et al. (2021), Ramaiahgari et al.—Listing of chemicals from published dataset in Ramaiahgari et al. (2019), PFAS–Listing of chemicals from published datasets in Reardon et al. (2021) and Rowan-Carroll et al. (2021), Buick et al.—Listing of chemicals from published dataset in Buick et al. (2021).

### 2.2 Datasets and exposure conditions

Six datasets were identified and selected for evaluation from previously published works as well as a published OECD case study on integrated approaches to testing and assessment (IATA) conducted by Health Canada (listed in Table 1). Within each dataset it was indicated when studies used human whole transcriptome (∼20,000 probes) kits, or reduced coverage using a subset of genes with the S1500+ panel (∼3,000 probes). Recent work evaluated the robustness of the S1500+ platform and demonstrated that this template is an acceptable surrogate for the whole transcriptome (Lee et al., 2021). A brief overview of the experimental designs (by cell model) has been provided below but readers are referred to the respective published studies for more detailed descriptions. The results herein were interpreted in the same manner regardless of the platform used.

Human breast cancer cells (Michigan Cancer Foundation-7; MCF-7) were depleted of estrogen for 48 h prior to exposure for 2 days to 16 bisphenols and bisphenol alternatives at ten concentrations ranging from 0.0005 to 100 µM alongside dimethyl sulfoxide (DMSO) solvent controls. The experiment included a positive control (17β-estradiol, range 0.0001–10 nM) and a non-estrogenic control (dexamethasone, range 0.0001–1 µM) (Matteo et al., 2022; OECD. Series on Testing and Assessment 373, 2022). A second dataset was produced with MCF-7 cells that were treated for 6 hours (0.25 days) with 44 different substances at concentrations between 0.03 and 100 µM (and DMSO solvent controls) alongside three chemicals (genistein, sirolimus, and trichostatin A) at a single concentration. These three chemicals were included for reference purposes but since a single concentration was used for each of them, we were not able to use them for concentration-response modeling (Harrill et al., 2021). Both experiments used human whole transcriptome TempO-Seq™ profiling.

Human HepaRG™ cells in differentiated (Hepa-D) and non-differentiated (proliferated, Hepa-P) states were exposed to 25 chemicals for 4 days (at 10 concentrations with half-log spacing) and assessed using the S1500+ platform (Ramaiahgari et al., 2019). A second set of human HepaRG™ cells were exposed to 12 potentially genotoxic (i.e., DNA damage inducing) chemicals for 2 days over a range of concentrations specific to each chemical and assessed using the S1500+ platform (Buick et al., 2021) and time-matched to solvent controls.

Primary human liver cell spheroids (3D spheroids; from 10 different human liver donors) were exposed to 4 PFAS (PFBS, PFOS, PFDS, and PFOA), including exposures of 1, 4, 10, and 14 days over a range of concentrations from 0.02 to 100 µM using the S1500+ platform alongside time-matched DMSO solvent controls (Rowan-Carroll et al., 2021). The 3D spheroid data was expanded to 23 per- and polyfluoroalkyl substances (PFAS) but limited to 1 and 10 day exposures based on previous work demonstrating optimal exposure of these chemicals assessed using the S1500+ platform (Reardon et al., 2021).

### 2.3 Data processing and handling

All included data were obtained in the form of count tables (read counts for each probe in each sample) generated using the Templated Oligo detection assay (TempO-Seq™, from Biospyder) that were processed and subjected to QA/QC analysis as outlined in the original publications. Previous analysis of TempO-Seq™ data from a variety of publicly available datasets had found that choice of aligners and normalization methods used to process data did not significantly influence expression outcomes, demonstrating the robust nature of results produced using this target RNA-seq platform (Everett et al., 2022). Thus, our meta-analysis of multiple transcriptomic datasets used the original pre-processed data that were produced from independent bioinformatic pipelines. In several of the studies (Buick et al., 2021; Matteo et al., 2022), a general bioinformatics pipeline designed to process transcriptomic data for regulatory applications was used for data pre-processing, known as the Omics Data Analysis Framework for Regulatory Application (https://github.com/R-ODAF/) (Verheijen et al., 2022). Other studies used the US EPA pilot pipeline (https://github.com/USEPA/httrpl_pilot) (Ramaiahgari et al., 2019; Harrill et al., 2021), or other custom analysis pipelines (i.e., TempO-SeqR, v3.0, provided by BioSpyder for aligning TempO-Seq data, alongside custom data pre-processing steps in R) (Reardon et al., 2021; Rowan-Carroll et al., 2021; OECD. Series on Testing and Assessment 373, 2022).

All the datasets in this study used a similar strategy to process data, starting with FASTQ files generated from the sequencing results of a TempO-Seq™ workflow, and ending with a tabular matrix. In this tabular matrix, the genes are represented in rows and samples are represented in columns, and the values in the matrix are the output (i.e., counts) from the alignment step. The steps in handling the data from these experiments include: 1) data quality assessment, 2) study-wide alignment quality controls; and 3) downstream applications to derive a tPOD. The first step, pre-processing, deals primarily with assessing the quality of the high-throughput sequencing data used in the study and creating the initial count matrix. Study-wide QC strategies were based largely on those described in Harrill et al., 2021, which aim to eliminate low-quality samples based on alignment to reference sequences and the distribution of gene counts in individual samples compared to all treatment-related samples in the dataset (i.e., calculating quality metrics such as alignment rates, panel coverage in terms of the number of active probes, etc., from the count matrix). Finally, downstream applications include calculating gene-level, pathway-level, or signature-level BMCs and subsequently deriving a tPOD, the results of which are filtered based on several statistical rules (e.g., R-ODAF criteria) at the per-gene or per-probe level. In this meta-analysis, we reanalyzed the datasets under consideration to compare methods for tPOD derivation.

### 2.4 Derivation of *in vitro* points of departure using transcriptomic concentration-response data

tPODs were derived from *in vitro* concentration response data using BMC modeling prior to conversion to AEDs. The workflow below describes the steps in the methodology developed to derive AEDs from original processed data. Prior to importing into BMDExpress software (v2.3) for analysis, the count matrix was imported to a DESeq2 object, and the size factor was normalized to log_2_ counts per million (CPM) as a separate function for each sample concentration (Yang et al., 2007; Phillips et al., 2019). A single project file (.bm2) was created using a consolidated listing of chemicals that was inclusive of all experimental conditions for analysis within BMDExpress. It is important to note that concentration data (generally with a minimum of three concentrations and solvent controls) are required to identify the BMC of responsive genes within the data. Here, a minimum of five concentrations were included along with a solvent control. The BMC modeling was performed separately for each experimental model for each test chemical and only used those samples (i.e., replicate concentrations) that passed all QC filters as defined by the criteria within their respective studies. Williams Trend tests (Williams, 1971) were applied to filter out probes that did not show a concentration-response (i.e., probes passing filters had *p*-values <0.05). Additional filtering was also applied to remove probes that did not achieve a fold-change (FC) of at least 1.5 in at least one concentration. To calculate BMC values for probes, the best-fit curve was selected from a series of possible models including Power, Linear, Polynomial 2, and Exponential 2, 3, 4, and 5 models. Best-fit models for probes were selected based on a nested chi-square test cut-off of 0.05 to select among the linear and polynomial models that was followed by the lowest Akaike Information Criterion that estimates the quality of each model relative to the other models. A full description of the modeling parameters is available in the BMDExpress2 published online documentation (US EPA BMDExpress2, n.d.; Phillips et al., 2019). Additional parameters applied for modeling included: restrict power equal or greater than one; maximum iterations of 250; confidence interval of 0.95, and benchmark response factor of one standard deviation (i.e., BMR of 1 SD). Post-filtering criteria included removing BMCs with a goodness-of-fit test *p*-value less than 0.1, a ratio of BMC upper bound (BMCU) divided by the BMC lower bound (BMCL) greater than 40; and removing BMCs that were greater than the highest exposure concentration. Probe IDs representing select genes passing all filtering criteria were converted to their corresponding Entrez Identifiers and were carried forward to be used for tPOD derivation.

We evaluated seven approaches to calculate tPODs falling under two separate tracks, using concentration-response modeling performed with log_2_ CPM data using BMDExpress. The first track used the distribution of genes (e.g., percentiles, numbered rank, and mode), while the second used the median gene BMC value of the lowest (i.e., most sensitive) gene set from a selection of available, open-source, curated, and peer-reviewed pathway databases. All of the included methods from both tracks are described in more detail below and density plots for each chemical meeting the minimum criteria to derive selected tPODs are provided in Supplementary Appendix S2.

#### 2.4.1 The fifth percentile

The fifth percentile gene was calculated using the BMC of the gene closest to the fifth percentile value of the BMC distribution. It represents the lower bound fifth percentile of all filtered gene BMCs commonly employed in previous work using the US EPA Toxicity Forecasting database (Paul Friedman et al., 2019) to derive *in vitro* PODs and has been employed within previous studies (Reardon et al., 2021; Rowan-Carroll et al., 2021) to derive tPODs [i.e., represented mathematically as floor (0.05 x # BMCs)]. The fifth percentile is a conservative metric value for the tPOD that targets the most responsive genes that may contribute to the toxicological response.

#### 2.4.2 The first mode

Several previous studies used genes from the first mode of the BMD frequency distributions (Qutob et al., 2018; Farmahin et al., 2019; Pagé-Larivière et al., 2019; Alcaraz et al., 2021). In the current study, density estimation was used with forward, backward, and centered differencing to estimate the first and second derivatives. The first mode was defined as the first point at which the first derivative changes from positive to negative with a negative second derivative (second derivative test).

#### 2.4.3 The 25th lowest ranked gene BMC

All genes with BMCs were ranked from lowest to highest. The 25th rank-ordered gene was set as the threshold and the BMC of this gene was used to indicate the concentration where a defined change in the transcriptome has occurred (Reardon et al., 2021; Matteo et al., 2022). Those chemicals that were unable to produce a representative value for this tPOD (i.e., did not have at least 25 responsive genes with BMCs) were identified as “inactive” based on this approach and alternatives methods were considered for tPOD derivation.

#### 2.4.4 The LCRD

The lowest consistent response dose (LCRD) was performed as described in Crizer et al. (2021). The LCRD is identified as the lowest BMC in a rank order of gene BMCs where all subsequent ratio values from adjacently ranked BMCs are within 1.66 (thereby eliminating unrealistically low gene BMCs that may be biological noise). BMCs in the ranked group are declared the consistent response group of BMCs (CRGB) as defined by Crizer et al. (2021), because all sequential BMCs in this group have at least one BMC, that is, within 1/4 log difference in value. The lowest BMC in the CRGB is then identified as the LCRD. It has been recognized that this method may be sensitive to a few exceptionally low BMCs (i.e., deriving an estimate that is overly conservative), particularly for an extrapolated BMC below the lowest dose. Here, a modification of the LCRD is presented where the BMCs were grouped as stated above except the LCRD was the lowest dose from the largest CRGB. This eliminates the issue of small groups (*n* = 2 or 3) of genes with low BMCs being defined as the LCRD.

#### 2.4.5 The lowest gene set

This approach to tPOD derivation was defined by the United States National Toxicology Program (now the Division of Translational Toxicology: DTT) (National Toxicology Program, 2018). In the current study, to capture the most sensitive gene set, genes and their associated BMC values were matched to their corresponding gene sets using three well-known public databases: 1) Gene Ontology (GO) Biological Process (Harris et al., 2008); 2) Reactome Pathways (Fabregat et al., 2017); and 3) Kyoto Encyclopedia of Genes and Genomes (KEGG) (Kanehisa et al., 2017). Gene sets that contained at least three genes with a BMC representing at least 5% of the gene set (based on total annotated genes) were selected. The lowest gene set approach was established to extract tPODs using genomic dose-response modeling, that is, linked to meaningful biological change while reducing the influence of background (i.e., noise) as the gene sets define biological functions/processes (National Toxicology Program, 2018). However, it should be noted that the function of the selected gene set is not considered when interpreting the data.

### 2.5 Conversion of transcriptomic points of departure to administered equivalent doses

IVIVE was used to convert derived tPODs (μM) to AEDs (mg/kg-bw/day), a more aligned metric for comparison with apical PODs from *in vivo* data. Each derived tPOD was converted to an AED using reverse dosimetry and available data within httk. The AED is the theoretical dose required to reach a given steady-state plasma concentration (*C*_*ss*_; Wetmore et al., 2015). In brief, the httk package version 2.2.1 (Pearce et al., 2017) in R (R Core Team: R Foundation for Statistical Computing, 2020) was used for IVIVE. To predict the *C*_*ss*_ in the current study, the three-compartment steady-state toxicokinetic model (“3compartmentss”) (modified from Wetmore et al., 2012; 2015) was used. The parameters required for this model are intrinsic hepatic clearance and plasma protein binding. Full absorption by gut is assumed when data on the fraction of compound absorbed by the gut was not available (Wetmore et al., 2012). The httk package also provides tools to perform Monte Carlo sampling that represents the inter-individual variability within the population (Ring et al., 2017). A similar approach to Paul Friedman et al. (2019) was used within the current work, wherein the *C*_*ss*_ was calculated using the “calc_mc_oral_equivalent” function in httk with the default parameters and output using the value depicted by the 95th quantile.

Then, the resultant *C*_*ss*_ was used to calculate the AED (mg/kg-bw/day) using Eq. 1:

$$AED\;\left(\frac{\frac{mg}{kg}}{day}\right)=bioactivity\;concentration\;\left(µM\right)\times \frac{\frac{1\;\frac{mg}{kg}}{day}}{C_{ss}\;\left(µM\right)}$$ (1)

### 2.6 Comparison of administered equivalent dose to corresponding *in vivo* data

For the purpose of the current study, *in vivo* data was collected from available databases for comparison with *in vitro* derived endpoints (e.g., AEDs). Data was extracted from the EPA CompTox (CompTox Chemicals Dashboard (epa.gov), as well as databases from the publicly available ECHA REACH dossier (Understanding REACH - ECHA (europa.eu), the OECD QSAR Toolbox (The OECD QSAR Toolbox - OECD), and dossiers available through Health Canada (Chemical substances - Canada.ca). The lowest value available from oral repeat dose (sub-chronic and chronic), developmental toxicology and reproductive toxicology studies was selected as the apical POD (Table 2). An expanded list was considered in cases of chemicals with an available lowest apical POD from multiple study types (Supplementary Appendix S3). The lowest apical POD from *in vivo* data was selected from available endpoints, including the no-observed–adverse-effect-level (NOAEL); lowest-observed-adverse–affect-level (LOAEL); no-observed-effect-level (NOEL); no-effect-level (NEL); lowest-observed-effect-level (LOEL); or the lowest-effect-level (LEL).

**TABLE 2:** List of chemicals with available *in vivo* datasets.

	Name	CASRN	Animal	Study Type	Method	Effect Level (mg/kg-bw/day)
EPA ToxVal	4-Cumylphenol	599-64-4	Rat	Repeat Dose	NOAEL	50
	AFB1	1162-65-8	Human	Repeat Dose	BMDL01	0.000078
	Atrazine	1912-24-9	Mouse	Reproductive	NOEL	0.001
	Bifenthrin	82657-04-3	Rat	Developmental	NOAEL	1
	Bis4CPS	620-92-8	Rat	Repeat Dose	LOEL	20
	BPA	80-05-07	Rat	Developmental	NOAEL	0.015
	Cyanazine	21725-46-2	Rat	Repeat Dose	NOAEL	0.005
	Cyclophosphamide	6055-19-2		Repeat Dose	Cancer	0.57
	Cyproterone ace	427-51-0	Mouse	Repeat Dose	NOAEL	125
	Eugenol	97-53-0	Rat	Repeat Dose	NOAEL	57
	Fenofibrate	49562-28-9	Mouse	Developmental	LEL	11.7
	Flutamide	13311-84-7	Rat	Repeat Dose	NOAEL	10
	Genistein	446-72-0	Rat	Developmental	NEL	20
	Lovastatin	75330-75-5	Mouse	Repeat Dose	NOAEL	30
	PFHxA	307-24-4	Rat	Repeat Dose	NOAEL	200
	PFOA	335-67-1	Mouse	Reproductive	LOEL	0.02
	Prochloraz	67747-09-5	Dog	Repeat Dose	NOAEL	2.5
	Propiconazole	60207-90-1	Mouse	Repeat Dose	NOAEL	2.7
	Pyraclostrobin	175013-18-0	Rat	Repeat Dose	NOAEL	3.4
	Reserpine	50-55-5	Mouse	Repeat Dose	NOAEL	0.12
	Rotenone	83-79-4	Rat	Developmental	LOAEL	0.75
	Simazine	122-34-9	Mouse	Reproductive	NOEL	0.005
	Trifloxystrobin	141517-21-7	Rabbit	Developmental	NOAEL	10
	Troglitazone	97322-87-7	Mouse	Repeat Dose	NOAEL	1200
	Vinclozolin	50471-44-8	mouse	Reproductive	LOEL	1
	Zidovudine	30516-87-1	Mouse	Repeat Dose	LEL	100
REACH	2DD-Glucose	66-13-6	rat	Reproductive	NOAEL	20
	BPAF	1478-61-1	Rat	Repeat Dose	NOAEL	3.5
	BPS	80-09-01	Rat	Reproductive / Developmental	NOAEL	10
	Cyproconazole	94361-06-5	Rat	Reproductive	NOAEL	1
	Estradiol	50-28-2	Rabbit	Developmental	NOEL	0.0003
	Lactofen	77501-63-4	Rat	Reproductive	NOEL	2.5
	TGSA	41481-66-7	Rat	Repeat Dose	NOEL	15
	Thiram	137-26-8	Dog	Repeat Dose	NOEL	0.84
	Urea	57-13-6		Developmental	NOAEL	500
OECD	B[a]P	50-32-8		Repeat Dose	LOEL	0.05
	Fenpyroximate	111812-58-9		Reproductive	LOEL	8.45
	Imazalil	35554-44-0		Reproductive	NOAEL	5
	PFOS	1763-23-1		Reproductive	NOAEL	0.03
HC	Propyl gallate	121-79-9		Repeat Dose	NOAEL	135

Under circumstances where a designated NOAEL or LOAEL was not available for select chemicals, an alternative value was selected to represent the *in vivo* endpoint. For aflatoxin B1 (AFB1), the lowest outcome available was the lower-bound BMD (BMDL). For cyclophosphamide, only endpoints from studies of carcinogenicity were available, resulting in a designated “cancer unit” used to represent the apical POD. For eugenol, the highest no effect level (HNEL) was established as the lowest apical POD for comparison. A concerted effort was made to capture the largest number of chemicals by collecting data using commonly available regulatory databases.

## 3 Results and discussion

### 3.1 A transcriptomic workflow for a diverse chemical space

Transcriptomic technologies provide information on gene expression and the initiation of molecular changes that occur prior to the development of apical effects. Mounting evidence supports that these alterations can be used to establish molecular-based PODs that are human health-protective in the absence of predicting a specific hazard (Johnson et al., 2022). Such a transcriptomic effect level would ideally identify exposure concentrations equal to or below those causing critical effects associated with adverse outcome pathways (Ankley et al., 2010). Previous studies using short-term animal exposure data have demonstrated that the use of both *in vitro* and *in vivo* derived tPODs are comparable or even more sensitive than apical PODs derived using regulatory guideline studies (Bhat et al., 2013; Thomas et al., 2013; Johnson et al., 2020; LaRocca et al., 2020). To accelerate the adoption of NAMs, frameworks to increase confidence for application in the regulatory decision-making process have been proposed that include the key elements of “fitness for purpose, human biological relevance, technical characterization, data integrity and transparency, and independent review” (van der Zalm et al., 2022). The meta-analysis described herein applies these concepts to increase scientific confidence in the use of tPODs, and their corresponding AEDs derived from *in vitro* data to further demonstrate support that this approach results in equal or greater protection of human health.

The workflow includes datasets that have previously undergone data processing which allows for the derivation of tPODs from a range of approaches (Figure 1). The recently published R-ODAF pipeline has been used for previous datasets generated at Health Canada. Although this pipeline is proposed as a “baseline reference” for data processing prior to BMC modeling (Verheijen et al., 2022), it is acknowledged that best practices have yet to be developed for analyses in the field of toxicogenomics, particularly for application in risk assessment frameworks. It has been argued that flexibility is required because methods and approaches should ideally be fit-for-purpose (Buesen et al., 2017). Practically, it is unlikely that a universal tPOD would be considered sufficient across all potential outcomes, or as a single best practice, and a range of approaches across diverse *in vitro* datasets should be considered. Integration of new methods into regulatory decision-making requires best practices and interpretation procedures to establish alignment and consistency of use for human health risk assessment. To work toward this goal, a subset of tPODs was identified based on data-distribution (e.g., percentiles, modes, and ranked genes), data-driven (e.g., LCRD), and/or pathway-related (e.g., gene set) approaches. Furthermore, applying a “baseline reference” approach such as the R-ODAF to as many datasets as possible alleviates some of the difficulty involved in comparing studies undertaken at various times and/or under different conditions. The current work used HTTr datasets with a defined workflow to investigate a variety of tPOD derivation methods that identify a point of concerted molecular change from exposure to a diverse set of environmental chemicals.

### 3.2 A general comparison of approaches

**FIGURE 2:**
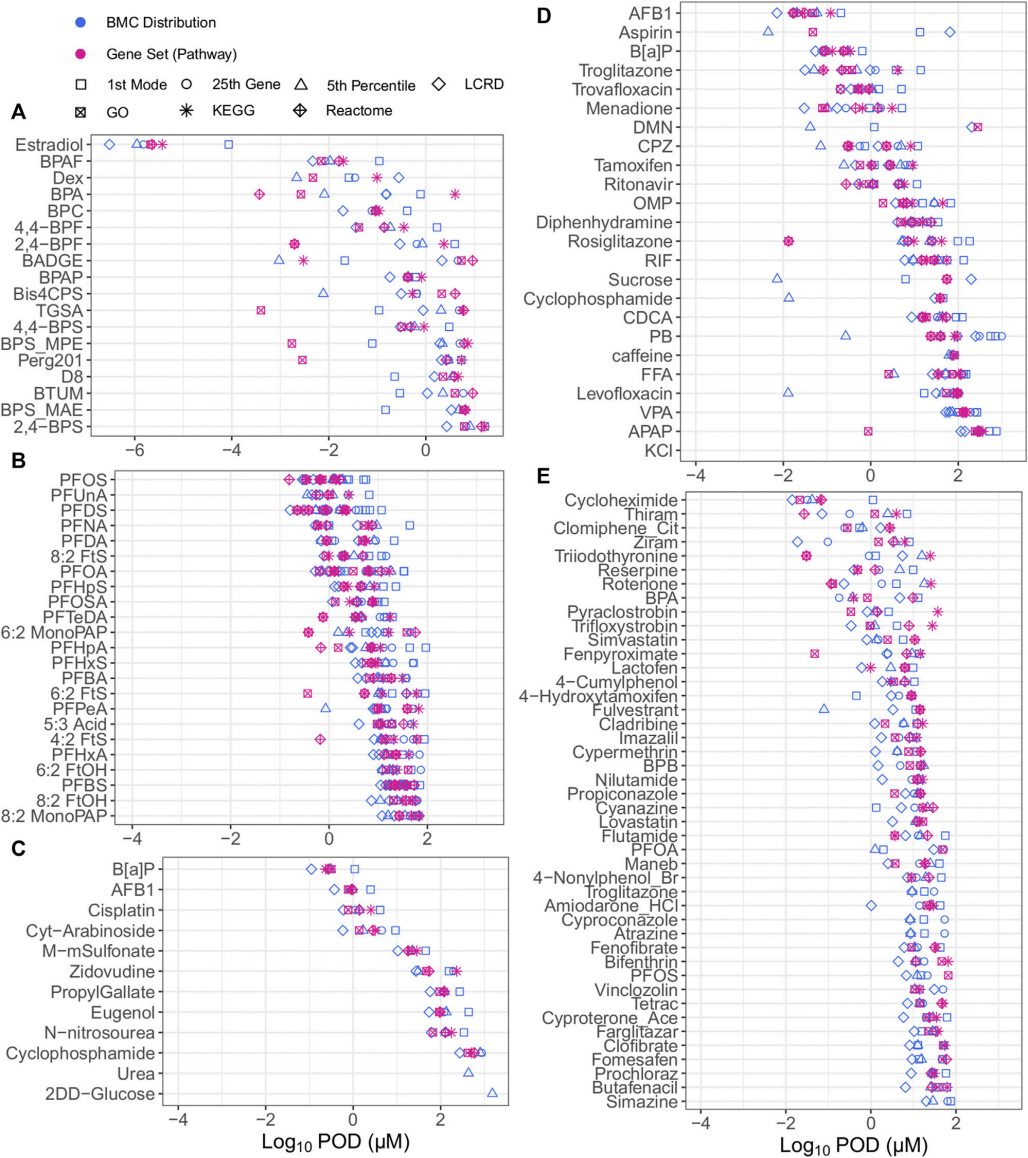
All tPODs of chemicals from the bisphenol case study group from Health Canada data in human MCF7 cells **(A)**, PFAS data from Reardon et al. and Rowan-Carroll et al. in human liver spheroids **(B)**, Buick et al., from exposed human liver HepaRG cells **(C)**, Ramaiahgari et al. from exposed human liver HepaRG cells **(D)** and Harrill et al. in human MCF7 cells **(E)**.

When examining all of the included approaches to tPOD derivation, bisphenols and bisphenol alternatives were the most potent chemicals but had the tPODs with the highest variability across all datasets (Figure 2); a comprehensive list of all tPODs for all chemicals within their defined datasets is provided in Supplementary Appendix S4. The overall median log_10_ value for all tPODs within the bisphenols group of exposed MCF-7 cells was −0.33 µM (Figure 2A), compared to values of 0.90, 0.91, 0.99, and 1.74 µM from investigations of PFAS (Figure 2B), or data from Ramaiahgari et al., (Figure 2D), Harrill *et al.*, (Figure 2E), and Buick et al., (Figure 2C), respectively. The degree of concordance between tPODs was evaluated by calculating the difference between the lowest (minimum) and highest (maximum) tPOD for each chemical (i.e., the higher the value of difference the lower the agreement between tPODs). Comparing between datasets, the median log_10_ difference from least to most agreement was 1.87 > 1.67 > 1.26 > 1.07 > 0.84 µM for the bisphenols case study, Ramaiahgari et al., HC PFAS studies, Harrill et al., and Buick et al., respectively (Figure 2). Thus, the bisphenols dataset had the largest median log difference across tPODs spanning multiple orders of magnitude (Figure 2A). Concordance between different tPODs on the same chemical within the Ramaiahgari et al. dataset was also quite low, but unlike bisphenols this was attributed to only a few outlier chemicals with individual tPODs derived using the fifth percentile that were orders of magnitude lower than tPODs derived using other approaches (Figure 2D). In contrast, other datasets such as the PFAS studies and those chemicals evaluated from Buick et al., yielded concordant and consistent tPODs regardless of the metric used (Figures 2B, C). This small range between tPODs of PFAS and those from Buick et al. is likely a consequence of the study design. Specifically, the use of a limited range of exposure concentrations that results in tighter groupings of derived tPODs. Overall, the data revealed the fifth percentile to be lower, in some cases by more than an order of magnitude, than the median of all tPODs for select chemicals, including Bis4CPS (Figure 2A); PFPeA (Figure 2B); aspirin, DMN, CPZ, sucrose, cyclophosphamide, PB, levofloxacin (Figure 2D), and fulvestrant (Figure 2E). Except for select bisphenol alternatives, the general agreement for the majority of observed tPODs across datasets of diverse chemicals reinforces the robustness and pursuit for the practical application of transcriptomic data for chemical potency ranking, grouping and risk assessment.

Although numerous approaches may be considered to derive tPODs for chemicals across a diverse space, specific approaches may over- or underestimate chemical potency. It was observed that tPODs derived using the fifth percentile produced high potency rankings for sucrose and other chemicals used as low hazard reference chemicals that have been rarely associated with liver toxicity (caffeine, levofloxacin, and aspirin) (Ramaiahgari et al., 2019). Furthermore, we note that percentile gene BMCs and the extent of transcriptional change (e.g., the total number of responsive genes) may be influenced by the top dose/concentration. For example, the fifth percentile tPOD for cyclophosphamide was 0.013 µM (derived from 14 genes fitting BMC models) in the Ramaiahgari et al., dataset using HepaRG cells that were exposed up to a top concentration of 300 μM. In contrast, the tPOD for this same chemical was 804.3 µM from the Buick et al. dataset using the same cell type where exposure ranges were up to 10,000 μM, thereby producing a more bioactive response (394 genes fitting BMCs). Although previously found to be effective when deriving a tPOD from a wealth of assays using ToxCast (Paul Friedman et al., 2019), deriving a tPOD at the single gene level (such as the fifth percentile) with transcriptomic data may produce inaccurate results (i.e., significant over- or underestimations), and should be interpreted with caution.

Alternatives to the fifth percentile include approaches with requirements and filters to reduce the potential for mischaracterizing chemical potency that includes, but is not limited to, the BMC distribution requiring at least 25 genes, or a gene set with a minimum of three genes and/or 5% of a pathway. The 25th ranked gene tPOD requires a minimum amount of biological activity as described by 25 concentration-responsive genes for tPOD derivation, decreasing the likelihood of chemicals with low bioactivity being identified as having the potential for toxicity. Although interpreted as an arbitrary value, the 25th ranked gene BMC in previous work provided consistent potency rankings of PFAS along with representing a toxicological response in approximately 0.1% of genes in the genome (Reardon et al., 2021). The LCRD tPOD “identifies the most sensitive non-outlier feature, that is, the plausibly representative lowest dose level where a consistent response in biological features is observed”, to identify a point of toxicological relevance (Crizer et al., 2021). Here, although there are a few exceptions, as an alternative approach to the fifth percentile the LCRD was generally the lowest and most conservative tPOD based on the distribution of BMCs across all chemicals, making it a promising candidate for deriving protective tPODs. In contrast to this, the first mode was predominantly the least conservative estimate in our study, consisting of the lowest proportion of tPODs. This approach requires the presence of a mode and, thus, a sufficient extent of biological activity. Previous work examining responses to 1060 chemicals across a battery of 815 *in vitro* assay endpoints suggested that the first mode generally corresponded to a disruption of specific biomolecular targets or pathways (e.g., receptors or enzymes) and generalized disruption of cellular machinery (Judson et al., 2016). However, observed effects at higher concentrations (i.e., higher modes) are often characterized by a larger number of affected pathways compared to the first mode, and represent dysregulation of cellular machinery that leads to cell stress and cytotoxicity. Thus, selecting the first mode as a tPOD provides an estimate at which initial molecular events are triggered.

As highlighted, there are multiple options when considering a means of obtaining a tPOD and the general agreement observed among chemicals for most datasets underscores the robust nature of gene expression data. For ease of comparison, the results, advantages, and disadvantages of all of the aforementioned approaches have been tabulated (Table 3). In summary, deriving tPODs using percentiles (e.g., the fifth) could lead to mischaracterization of a chemicals potency when interpreting the results; whereas other approaches such as the 25th ranked gene, the LCRD, the first mode, and the lowest gene set (further described below) provide viable alternatives to derive relevant and protective tPODs to facilitate risk assessment activities.

**TABLE 3:** Summary table of advantages, disadvantages, and results of evaluated approaches for derivation of transcriptomic points of departure.

	Result	Advantages	Disadvantages
The 5th percentile	The 5th percentile is a conservative value for tPOD derivation that targets the lowest and most responsive genes. Produces the highest potency ranking for select substances, even for chemicals identified as ‘non-toxic’ reference chemicals. Not considered as a reliable approach to derive tPODs using gene expression data.	Simplified calculation of estimate that uses the 5th percentile gene from the full distribution of genes with modeled BMCs. No additional models or calculations are required beyond BMD modeling software.	Subject to influence from the experimental design (e.g., the top dose/concentration). Subject to influence by the extent of transcriptional change (i.e., total number of genes with BMCs).
1st Mode^a^	The 1st mode uses those genes from the identified first mode based on the frequency of distribution of genes with BMCs. Considered the least conservative but is a consistent and simple approach to derive tPODs using distributions.	Consistently low (i.e., conservative) estimate across majority of listed chemicals. Corresponds with disrupted biomolecular targets and pathways, and provides an estimate of the initiation of molecular events Judson et al. (2016)	Requires sufficient biological activity (i.e., genes with BMCs) for identification and calculation of modes.
25th Ranked Gene^a^	The 25th ranked gene is set as a threshold that is identified from a relative potency ranking of the lowest to highest genes with BMCs. Considered a simple approach deriving tPODs using the BMC distribution that represents approximately 0.1 % of the genome.	Simplified calculation using the 25th ranked gene by potency based on the distribution of genes with modeled BMCs. No additional models or calculations are required beyond BMD modeling software.	Requires a minimal amount of biological activity (e.g., 25 concentration-responsive genes). Excludes select chemicals with insufficient biological activity (e.g., either non-liver toxic, or highly cytotoxic chemicals that do not meet a minimum required concentration-responsive genes).
LCRD^a^	The LCRD is the value of the lowest of rank-ordered BMCs using consistent response groups of BMCs Crizer et al. (2021). Represents the primary approach to derive conservative tPODs for most of the listed chemicals that were carried forward for comparison with apical PODs.	Uses a calculated derivation for tPOD derivation that represents a point of toxicological relevance that is considered a consistent response of all biological features. Predominantly the lowest and most conservative tPOD of all approaches based on the distribution of BMCs across all chemicals, and suggested as a promising candidate for deriving protective and conservative tPODs.	Requires more complex modeling to obtain a relevant tPOD based on the literature.
Lowest Gene Set^a^	The lowest and most sensitive gene set is widely accepted as a means of obtaining a tPOD as outlined in a guidance document National Toxicology Program (2018). Reliable approach in scenarios with sufficient biological activity to derive tPODs using the median value of the lowest and most potent (i.e., sensitive) gene set. tPODs primarily defined using annotations from the largest and most comprehensive database (e.g., GO).	Established and reliable approach to derive tPODs using dose-response models that reflect meaningful changes in biology while reducing the influence of background. Option to choose from multiple available databases to represent annotations used to define gene sets (e.g., GO, KEGG or REACTOME).	Requires more complex modeling and additional parameters and filtering to obtain a relevant tPOD based gene sets. Requires sufficient biological activity (i.e., genes with BMCs) to define subsets of genes related to gene sets and pathways.

a Approaches that were considered to be alternatives to be applied *in lieu* of approaches using percentiles (e.g., 5th) to derive a transcriptomic point of departure.

### 3.3 Conversion to human relevant exposure values

**FIGURE 3:**
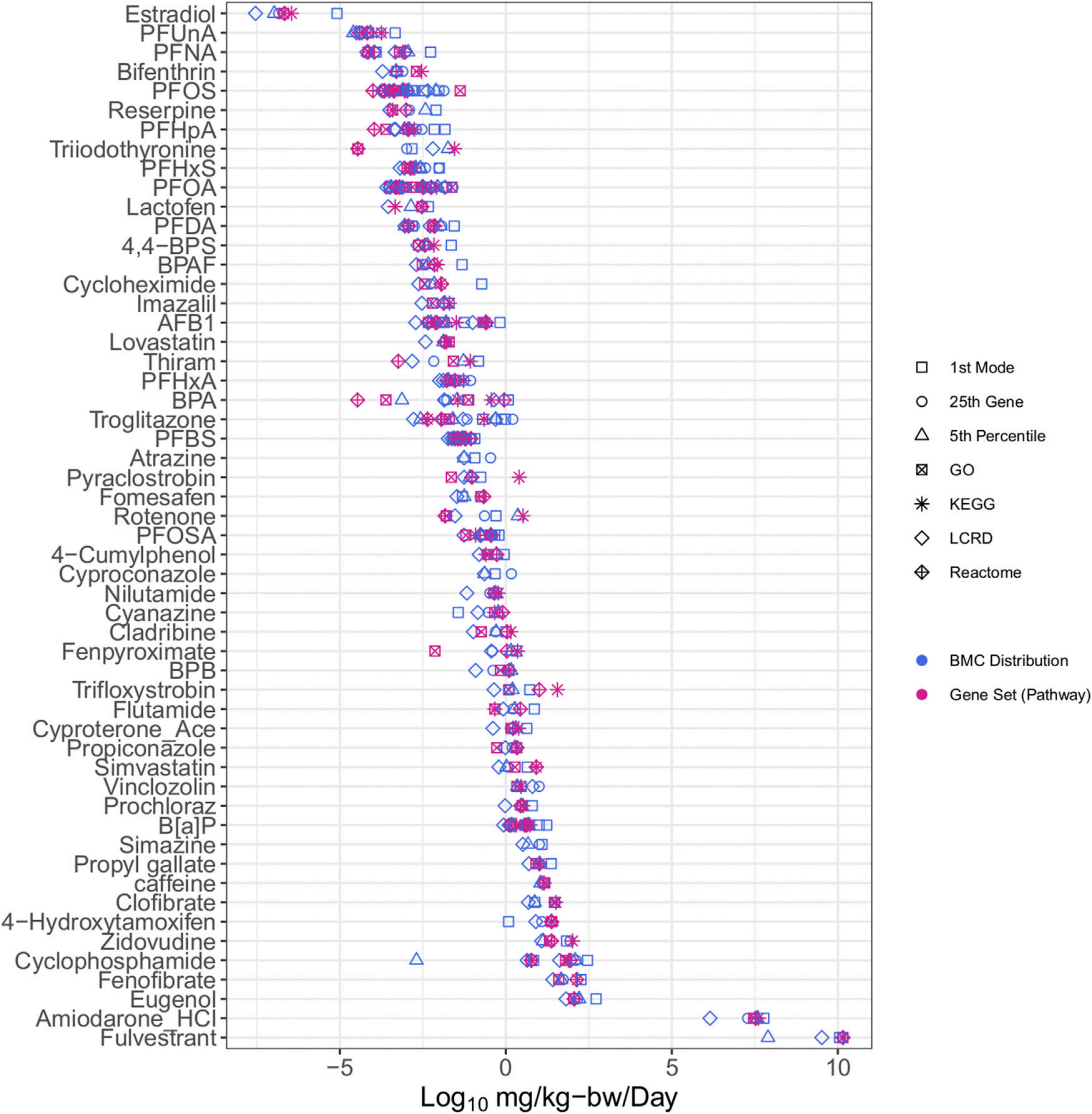
All case study chemicals with available data for conversion using IVIVE ranked by median potency of AEDs.

The integration of IVIVE within the workflow allowed for determination of a dose that converts the tPOD (µM) to an estimate of the surrogate bioactivity POD (i.e., the AED in mg/kg-bw/day). Chemicals with AEDs (mg/kg-bw/day) were ranked by relative potency (Figure 3). The list was reduced from the 117 chemicals with tPODs (Figure 2) to 54 chemicals that could be modeled in the httk R-package (Figure 3). The bisphenols and alternatives group (specifically BPA and BPAF) that were previously ranked as some of the highest and most potent chemicals based on derived tPODs were no longer among the most highly potent and instead were within the middle of the relative chemical potency ranking. Furthermore, the PFAS group that was not particularly potent based on tPODs (Figure 2) included 7 of the top 12 most potent chemicals when ranked by their AEDs (Figure 3). Other select chemicals (2DD-glucose, TGSA, Bis4CPS, and urea) had apical PODs from animal data but AEDs from BMC distributions or gene set approaches could not be produced. These chemicals were subsequently excluded from further analysis.

The conversion of tPODs to AEDs had two primary impacts on the results and interpretation. First, it reduced the number of chemicals that could be analyzed because of a lack of data availability for IVIVE. Second, AED derivation altered the relative potency ranking of chemicals. *In silico* models are currently being employed to predict the parameters needed to broaden the application of NAM-based PODs toward “characterisation, validation and reporting of Physiologically Based Kinetic (PBK) models for regulatory purposes” under the OECD (OECD, 2021). These models allow for the determination of a C_*SS*_ and are a critical step in creating relevant exposure estimates from *in vitro* data that could be applied in the context of human health risk assessment. Here, the AED was employed as a quantitative estimate to serve as a protective human-relevant effect level that was anticipated to be lower than potential apical adverse outcomes. This process has been previously employed to determine protective estimates for potentially hazardous chemicals using data derived from the US EPA toxicological forecast (ToxCast) database (Paul Friedman et al., 2019; Health Canada, 2021). The primary advantage of combining IVIVE with human cell models or tissues is that a correction factor for sources of uncertainty related to interspecies differences from animal-derived data may not be necessary for developing regulatory values (Bos et al., 2020). Furthermore, although it is beyond the intent of the current work, the httk platform also has the potential to accommodate input of parameters from *in silico* predictions such as for the fraction of chemical not bound to protein and intrinsic hepatic clearance, to model the C_ss_ for chemicals not listed within the httk chemical library.

The implementation of IVIVE using *in silico* models and corresponding model assumptions also have potential limitations. In our study, a lack of available data on the parameters necessary for IVIVE reduced the overall sample size (i.e., number of chemicals included in our study); this is particularly problematic when evaluating “data-poor” chemicals that are also outside the applicability domain of models used to provide predicted input parameters. The AEDs produced using the PFAS data were influenced by IVIVE resulting in their increased potency ranking. The generalized parameters and assumptions of IVIVE models may not have adequately captured their toxicokinetic properties and disposition, resulting in overestimation of their chemical potency (e.g., prolonged half-life) (Fenton et al., 2021). Thus, although there is some inherent uncertainty associated with httk models due to the (conservative) assumptions necessary to allow for minimal data input and high-throughput data processing, it has been demonstrated to be a useful approach to provide protective AEDs from *in vitro* data. Here, httk was used to convert tPODs to AEDs to determine practical, human-relevant effect levels that would allow risk assessors to make informed decisions, even when traditional animal data was not available. Ongoing efforts at Health Canada, as part of international collaborations, are working to characterize the impact of different IVIVE models for reverse dosimetry, *in vitro* disposition models, as well as parameterization of models using various data streams, on the refinement to the AED estimate. Such work will increase confidence in the use of IVIVE approaches and provide guidance on when higher tier models should be considered based on chemical space and context of use.

**FIGURE 4:**
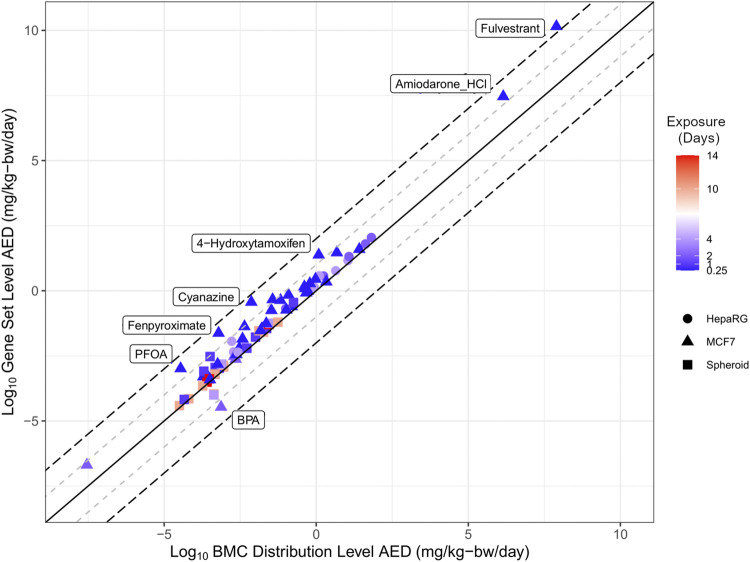
Correlation plot of the relationship between the lowest derived AED from using the BMC distribution level and gene set level approaches. The solid line represents perfect agreement between data on the *x*-axis with data on the *y*-axis, with a range of one (+/−1 log10, grey dashed line) or two (+/−2 log10, black dashed line) orders of magnitude.

The estimate from the lowest and most conservative approach for each individual chemical was identified for both the BMC distribution (blue markers, Figure 3) and gene set level (magenta markers, Figure 3) approaches were carried forward to evaluate the extent of correlation between different tracks (Figure 4). Overall, there was a degree of agreement (within one order of magnitude of perfect agreement) for the majority of chemicals (∼90%), even considering differences in experimental design including exposure time, model type and approach used to derive tPOD [Pearson correlation, *r* = 0.98 (*p* < 0.0001)]. Of these chemicals, select data points (from BPA, PFOA, fenpyroximate, cyanazine, 4-hydroxytamoxifen, and amiodarone hydrochloride) were more than an order of magnitude outside the range of agreement (labeled in Figure 4), and fulvestrant was significantly (greater than two orders of magnitude) outside the range of agreement. For BMC distribution level values, over the chemical space, the lowest AED for the majority of substances was the LCRD (45 of 54), followed by the fifth percentile (6 of 54), and the remaining three represented by the first mode and 25th ranked gene. Thus, the AEDs of over 90% of concentration-response experiments were based on the LCRD and only a small number of chemicals were represented by the fifth percentile (atrazine, BPA, caffeine, fulvestrant, PFDA, and vincolozolin). As previously discussed, the fifth percentile is subject to bias from the concentration exposure range; this may explain fulvestrant being significantly outside the range of agreement.

Overall, the results demonstrate a high degree of concordance between BMC distribution and gene set level AEDs produced using a diverse set of approaches. The application of httk generated human relevant AEDs from *in vitro* derived tPODs to produce practical estimates for potentially hazardous and data-poor chemicals.

### 3.4 Comparing *in vitro* to *in vivo* derived data

**FIGURE 5:**
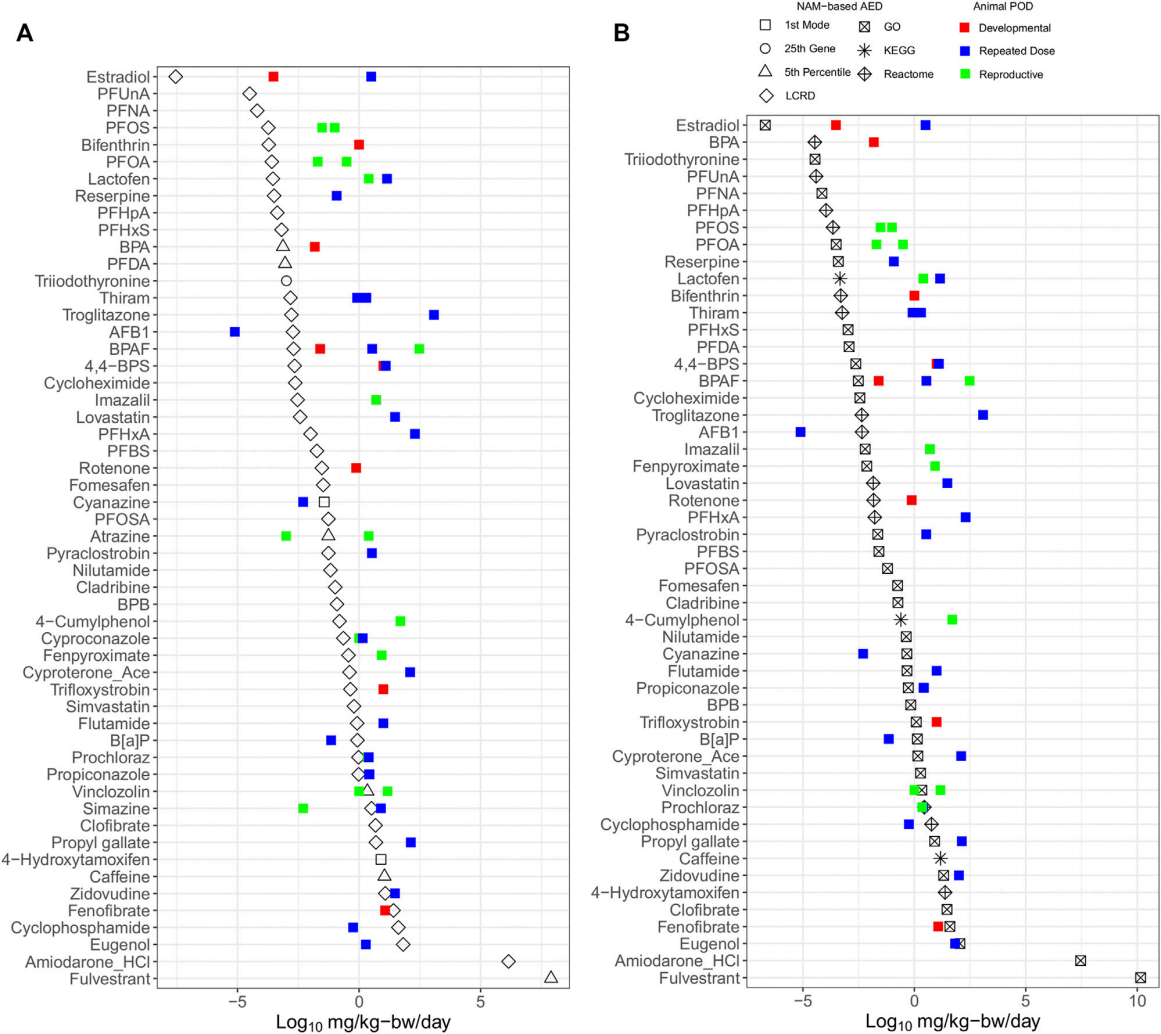
Relative potency ranking of points of departure for each chemical selected from the lowest value derived using BMC distribution **(A)** and gene-set **(B)** level AEDs, compared to *in vivo* apical points of departure.

The AED for each chemical was selected from the lowest value of either the BMC distribution (Figure 5A) or lowest gene set (Figure 5B) for comparison with apical PODs derived using *in vivo* data from regulatory databases (Table 2). The AEDs (black) were generally lower than apical PODs from developmental (red), reproductive (green) and repeat dose (blue) studies (Figure 5). Using the BMC distribution, the lowest AEDs were derived using the LCRD, fifth percentile, first mode, or 25th ranked gene approaches for 45, 6, 2, and 1 of 54 substances, respectively, wherein for most (47 chemicals), there was an observed pattern of lower tPOD-based AEDs than apical PODs (Figure 5A). Another commonly employed method of tPOD derivation uses the median value of the lowest/most-sensitive gene set as a measure of potency (National Toxicology Program, 2018). AEDs derived from the lowest gene set found that most of the observed AEDs (45 of 50) followed the same pattern and were also lower than apical PODs (Figure 5B). Most AEDs from the lowest/most sensitive gene set were derived from tPODs using the gene ontology (GO) database (33 of 50), followed by REACTOME (14 of 50), and KEGG (3 of 50) (Figure 5B). A general pattern of lower/more conservative AEDs was observed, suggesting that for the majority of chemicals, NAM-based AEDs using either distribution or gene set based approaches are at least equal to, or more conservative than conventional apical endpoint PODs selected for risk assessment purposes.

The LCRD was the most consistent approach from the BMC distribution and GO was the consistent data source for the lowest median gene set level approach amongst the multiple methods of tPOD derivation examined. As previously discussed, the LCRD is a recently developed data-driven approach to derive PODs using transcriptomic data. Our results indicate this approach to be well-suited to derive conservative tPODs across a diverse chemical space. Previous work using functional enrichment and predictive modeling to identify lowest median genes sets showed that results differed when assessing equivalent pathways from different databases; thus, database choice is a significant factor when using pathway-centric approaches (Mubeen et al., 2019). The size of the gene set database may also be a significant factor, as the number of pathways present will influence results (Mubeen et al., 2022). Our finding that the GO database produced the highest proportion of gene set AEDs was likely attributed to its size, with a larger number of available human annotations compared to REACTOME and KEGG. An optimal database for gene sets has yet to be established. Thus, at present we recommend including multiple databases to ensure that a broad biological space is queried and that a conservative AED is derived.

AEDs may be used to provide a protective dose below which toxicity is not expected to occur in support of chemical screening. Previously, AEDs derived using the fifth percentile of values from a large set of *in vitro* assays using available ToxCast data were compared with apical PODs from *in vivo* data to derive a practical risk-based metric for prioritization and assessment activities; namely, the bioactivity exposure ratio (BER) (Paul Friedman et al., 2019; Health Canada, 2021). During a formal risk assessment, the most appropriate critical effect level from animal studies is typically selected after a thorough review that includes a study quality evaluation, wherein the lowest value recorded as the apical POD may not always be the most fitting estimate for comparison with estimates of exposure. The rationale for exclusion may be based on limitations in the study design, the quality of the data or reporting, or may not consider a particular sub-population that represents the exposure scenario of interest. For example, the NOAEL from a developmental or reproductive toxicity study may be most suitable when evaluating the risk for women during pregnancy and their developing children but in some cases may also be generalized to include women of childbearing age or men. Here, a comparative assessment indicated that transcriptomic based AEDs were equally or more protective than the majority of apical PODs, regardless of the study type considered (e.g., repeated dose, reproductive, or developmental toxicity). Such AEDs may be applied in the same manner to create human relevant BERs, reinforcing the effectiveness of NAM-based approaches to create protective, human relevant PODs for consideration in risk assessment.

Additional chemicals had a sufficient number of modeled genes to derive BMCs from distribution level approaches (53, 62, and 100 total genes with BMCs for cyproconazole, atrazine, and simazine, respectively, Figure 5A) but not gene set level approaches (Figure 5B). There is likely an insufficient number of these genes with BMCs that could be mapped to a gene set in each of the KEGG, GO, or REACTOME databases after application of parameters and filters. Thus, although our study had a somewhat limited sample size because of data availability, the results included both focused datasets with numerous chemicals from a single class (e.g., PFAS and bisphenol alternatives) as well as datasets with substances from broader classes (e.g., Ramaiahgari et al., and Harrill et al.). These findings support observations that transcriptomic AEDs derived from a variety of approaches provide a conservative endpoint for evaluating chemical potencies and emphasize the importance of including multiple databases when mapping genes for AED derivations that use gene sets.

### 3.5 Identifying outliers using ratios

A Log_10_Ratio was used to compare AEDs from *in vitro* data with apical PODs from traditional data (adapted from Paul Friedman et al., 2019). The ratio was produced using the log_10_ (mg/kg-bw/day) units and calculated by the difference between NAM-based AEDs and traditional PODs with the following equation (Eq. 2).

$${\mathrm{Log}}_{10}Ratio={\mathrm{Log}}_{10}{POD}_{Traditional}-{\mathrm{Log}}_{10}{AED}_{NAM}$$ (2)

**FIGURE 6:**
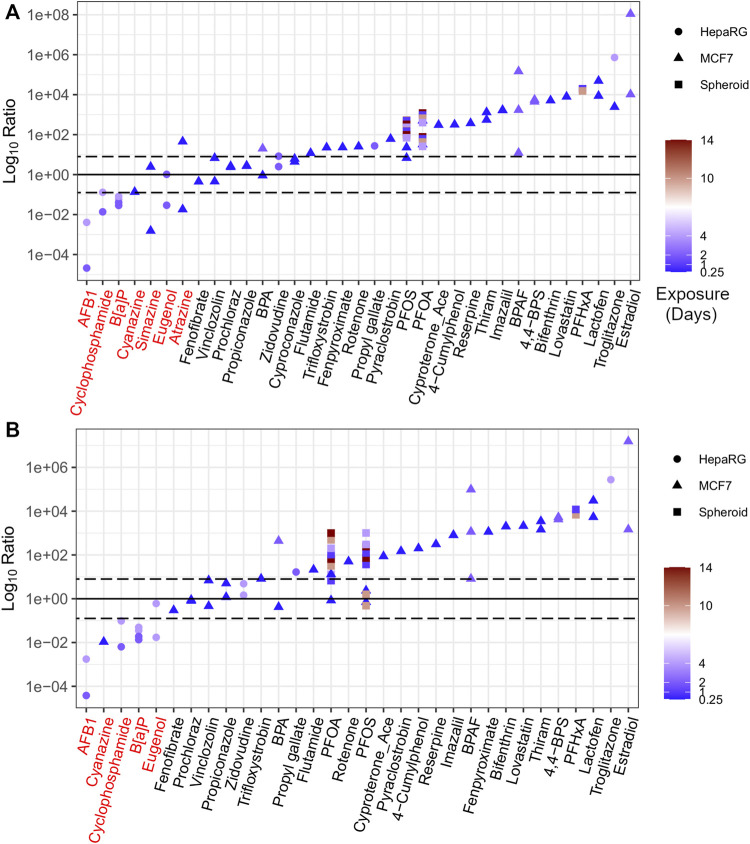
The ratio of *in vitro* derived AEDs to apical PODs ranked using BMC distribution level **(A)** and gene set level **(B)** approaches. Chemicals highlighted in red represent outliers based on ratio less than 0.

The majority of values for the Log_10_Ratio were > 0, with NAM-based AEDs being more conservative than traditional PODs (Figure 6). Using BMC distribution approaches, 28 of 35 chemicals (corresponding to 60 of 72 included data points, or 83% of the dataset) had a median Log_10_Ratio > 0 indicating that these AEDs were lower than apical PODs (Figure 6A). Using gene set level approaches, 27 of 32 chemicals (corresponding to 56 of 66 included data points, or 85% of the dataset) had AEDs lower than apical PODs (Figure 6B).

In contrast, those chemicals that were flagged as outliers (with a Log_10_Ratio < 0) included three chemicals identified as triazine herbicides (simazine, atrazine, and cyanazine), as well as B[a]P, eugenol, cyclophosphamide, and AFB1 (Figure 6). A detailed description for each of these chemicals, including the *in vitro* AED, the comparative apical POD, and a rationale to define those chemicals as outliers is detailed in the supplementary information (Annex A). In brief, atrazine, cyanazine, and simazine were included in previous work as triazine herbicides for their capacity to inhibit photosystem-II that were not intended to be active within the cell model of interest (MCF-7) and, as a result, were outside of the applicability domain for the target cell culture system resulting in non-conservative tPODs (Harrill et al., 2021). B[a]P has numerous routes of exposure for humans that results in developmental, reproductive, and immunological toxicity *in vivo* (US EPA, 2017) that may not be captured within the current *in vitro* model (HepaRG). The higher *in vitro* POD for eugenol than apical PODs may also be attributed to the limited applicability domain of the *in vitro* models used to derive the tPOD and subsequent AED for this chemical. Cyclophosphamide requires metabolic activation, primarily through the liver, in order to induce varying degrees of toxicity (Ayash et al., 1992; Moghe et al., 2015; Groehler et al., 2016). AFB1 also requires bioactivation in order to enact hepatocarcinogenesis resulting in AED estimates that were higher (i.e., less conservative) than epidemiological PODs from incidences of human liver cancer (EFSA, 2007). Although the HepaRG cell models used within the *in vitro* studies are considered to be more metabolically active than more simple *in vitro* human hepatocyte models (Lübberstedt et al., 2011), they likely do not maintain a high enough metabolic capacity in conventional 2D static culture models to induce the level of toxicity observed with cyclophosphamide or AFB1 *in vivo*. This limitation could be resolved by using a more sophisticated model such as a liver spheroid or microtissue, or via the addition of a metabolic component (e.g., an S9 fraction) to provide a more accurate *in vitro* tPOD and corresponding AED.

The Log_10_Ratio provides an efficient means for evaluating the effectiveness of *in vitro* PODs to derive conservative estimates for numerous substances across a broad chemical space. Overall, we found that the lowest and most conservative value from the range of derived AEDs was lower (i.e., protective) than available *in vivo* derived apical PODs for the same chemicals with a few exceptions (e.g., outlier chemicals). Within this context, the workflow could be applied in a tiered framework to highlight or flag potentially hazardous or problematic chemicals for further research, data generation or risk assessment.

### 3.6 Examining design considerations and uncertainty when applying transcriptomic data for risk assessment

#### 3.6.1 Study design

**FIGURE 7:**
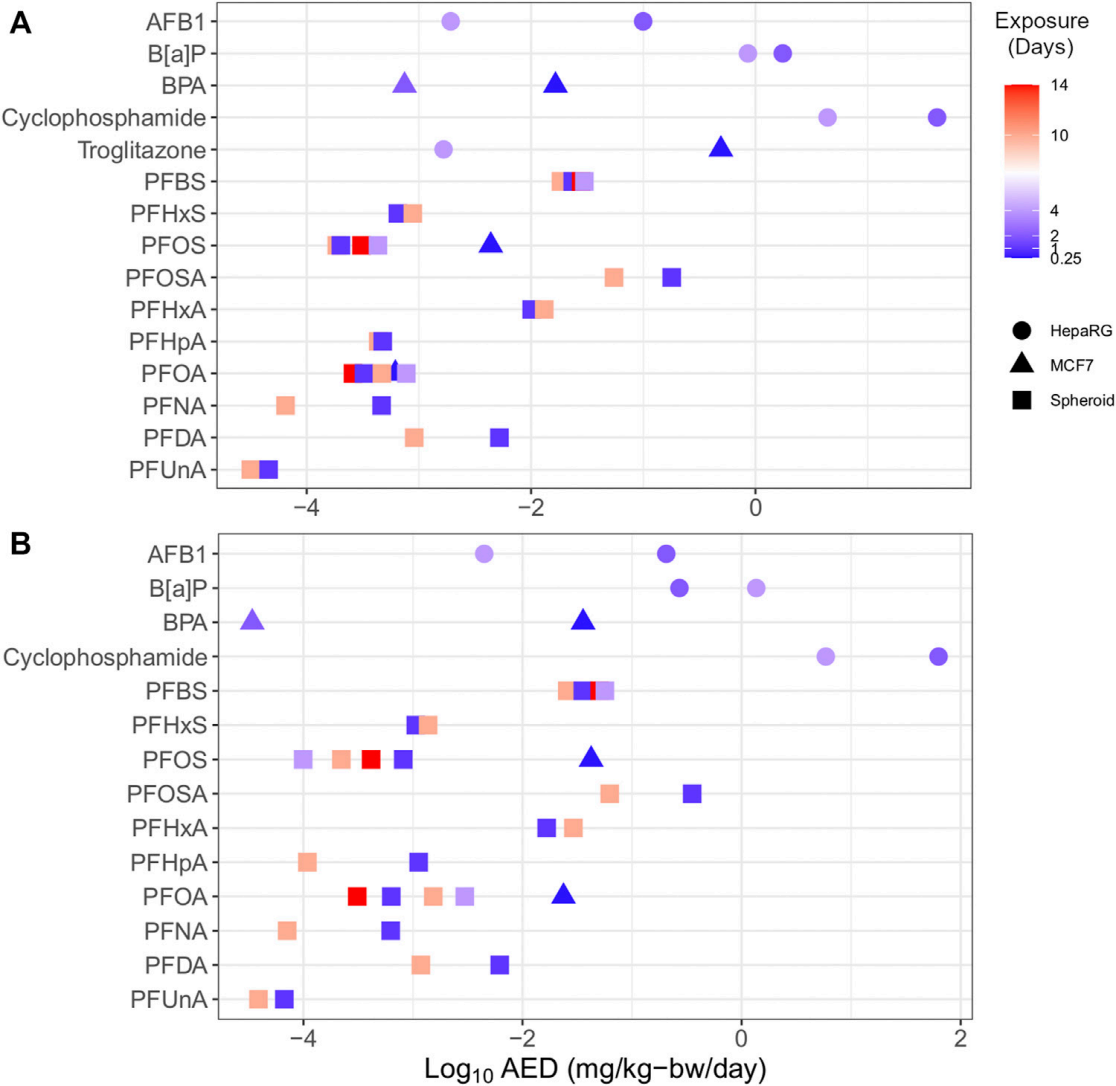
Plot of substances with multiple endpoints demonstrating the influence of time (by colour; range 0.25–14 days) and model (shape) on transcriptomic AEDs derived from *in vitro* data using BMC distribution level **(A)** and gene set level **(B)** approaches.

Multiple HTTr data sets could be used to examine the effect of study design parameters on tPODs and AEDs. Specifically, increasing the duration of exposure and/or model complexity generally corresponded to increased chemical potency (Figure 7). PFAS represented the majority of chemicals with varied data points (e.g., multiple time points and cell models), including carboxylates (PFHxA, PFHpA, PFOA, PFNA, PFDA, and PFUnA), sulfonates (PFBS, PFHxS, and PFOS), and a longer-chain perfluorosulfonamide (PFOSA). With a few exceptions, the PFAS showed agreement (within one order of magnitude) between multiple AEDs produced from experiments using these different designs. Outside of PFAS, AFB1, B[a]P, BPA, cyclophosphamide, and troglitazone from independent experiments produced multiple AEDs that, with the exception of B[a]P, were different by orders of magnitude. Considering all of these chemicals, there was a trend of increasing potency (i.e., a decreased AED) with increased exposure duration. For specific chemicals with available data, AEDs derived using more complex 3D liver spheroid models (e.g., PFOS) or HepaRG cells (e.g., troglitazone) were lower than AED from MCF-7 cell models. These outcomes suggest a need to consider uncertainty relating to the study design used, such as the exposure duration and model when applying these values in risk assessment applications.

#### 3.6.2 Uncertainty relating to apical endpoints

Prior to discussion of the uncertainties related to *in vitro* tPOD and AED derivation, it is important to consider the main sources of uncertainty related to traditional approaches and conventional animal testing. Currently, PODs for health effects are derived using modeled apical measures such as NOAELs, LOAELs, and BMDs from relevant studies. Uncertainty factors are commonly applied to PODs to compensate for limitations, knowledge deficiencies, and uncertainties in the data. Such limitations have been previously discussed (Kimmel and Gaylor, 1988; Leisenring and Ryan, 1992; Haber et al., 2018). Sources of uncertainty include dose selection and dose spacing that are identified when defining a NOAEL or LOAEL using a limited range of doses within the experimental design. Moreover, the dose–response is not accounted for in NOAEL or LOAEL derivation as these estimates are derived based on the effect observed at a single dose. Among different studies the observed experimental response in animal models may vary, making inter-study comparisons challenging (Kimmel and Gaylor, 1988; Leisenring and Ryan, 1992; Haber et al., 2018). Recent work has attempted to quantify inherent biological and protocol variability of inter-laboratory results using curated reference data for acute oral rat LD_50_ that resulted in values that varied by approximately ±0.24 log_10_ units (mg/kg) (Karmaus et al., 2022). Pham et al., performed a statical evaluation and estimated a variance of 0.5–0.6 log_10_ units (mg/kg/day) for LEL and/or LOAEL values using critical effect level outcomes (e.g., target organ, clinical chemistry, or in-life observation) from *in vivo* studies within the publicly available EPA Toxicity Reference Database (Pham et al., 2019; 2021). Herein, the use of apical PODs provided a reference point to compare and contrast the NAM-based effect levels; specifically, the results suggest that *in vitro* derived tPODs and AEDs are generally more conservative than apical PODs. Given the range of AEDs observed based on multiple studies within the current work, it is also important to recognize that similar ranges, and uncertainties exist for apical PODs and strategies are used to account for these within decision-making frameworks. The identification and quantitation of uncertainty are currently being investigated as part of on-going efforts to implement *in vitro* derived data into current and future risk assessment strategies.

#### 3.6.3 Uncertainty relating to in vitro models to derive AEDs

Aspects of the design, including the *in vitro* experimental model used, as well as the selection of the dose range and spacing of the exposure are subject to uncertainty. Thus, aspects of the design may influence the magnitude of AEDs from derived tPODs that reflect the observed chemical potency. There are numerous factors that should be considered in order to provide a practical fit-for-purpose molecular-based dose estimate. One primary factor is the selection of an appropriate and representative *in vitro* model. For example, immortalized cancer derived cell lines are frequently used in toxicology experiments because they are widely available, easily cultured and facilitate reproducibility (i.e., the same cells can be used for all experiments). However, these cell lines may not reflect the response of non-cancer tissues. Primary cells directly derived from humans retain tissue-specific characteristics but have low proliferative potential and are typically only used in a limited number of experiments (Liu et al., 2020). Immortalized cancer cell lines typically lack the capacity to metabolically activate chemicals to produce an accurate AED. The use of model mixtures including spheroids and microtissues consisting of primary cells derived from multiple donors may provide an effective means to capture more human-relevant responses. These complex, multi-donor models reduce any donor-specific biases in the data output. Furthermore, immortalized cell lines are generally maintained *in vitro* in a monolayer or in suspension, whereas microtissues using 3D models containing multiple cell types better reflect *in vivo* characteristics (Proctor et al., 2017; Reardon et al., 2021; Rowan-Carroll et al., 2021). Although complex spheroid models may be more suitable, they have limitations related to donor heterogeneity (i.e., sampled from a limited number of individuals), cost-efficiency, and availability (Fraczek et al., 2013; Zeilinger et al., 2016; Ruoß et al., 2020). Spheroid models were observed to be more sensitive for PFASs, but data within these more complex models was not available for the identified outlier chemicals that are known to require metabolic activation, such as cyclophosphamide, AFB1, and B[a]P.

In our study, the emphasis was on the derivation of protective tPODs that did not dive into the underlying mechanistic data to predict mode-of-action. However, it is acknowledged that the mechanism of toxicity can be an important consideration as specific tested cell lines and cell types have shown cell-dependent differential sensitivity to specific chemicals (Lawal and Ellis, 2010; Robert et al., 2014). Further to this, immortalized and transformed cell lines that have abnormal/unstable karyotypes may not produce the transcriptomic responses that are consistent with expected responses in normal human tissues (Kleensang et al., 2016). Cell monocultures do not reflect the complexity of organisms or represent the heterogeneity of the human population. As mentioned previously, metabolism (biotransformation) of chemicals *in vivo* may be different from that observed in exposed cell lines (Wilk-Zasadna et al., 2015). Metabolic pathways and resultant active by-products or metabolites are often difficult to fully predict but are also important considerations for method development and integration that would be required in the context of the paradigm shift to non-animal testing and assessment approaches. Overall, there are a variety of potential uncertainties that must be brought to bear relating to the *in vitro* cell models used. These aspects of non-animal models are being widely studied in parallel to improve the understanding related to impact for use in the derivation of effect levels and to develop methods that will address the inherent challenges of *in vitro* models. Increasingly in vivo-relevant models will inevitably lead to increased confidence in NAM-based approaches in the future. In the interim and to advance this area, there is a continued need to evaluate the fit-for-purpose use of the NAM in the context of the information gap being addressed. In parallel, the way in which apical effects from animal studies are used for regulatory decision making in light of their respective uncertainties must be reflected upon.

Along with cell system and model, the selection of dose-range and dose-spacing were identified as sources of potential uncertainty. The range and spacing of exposure concentrations of chemicals within the design can influence the potency of chemicals represented by derived tPODs and subsequent conversion to AEDs. Inaccurate selection of concentrations compromises the ability of *in vitro* models to reproduce the initiated changes as a result of exposures that are representative of *in vivo* biology, influencing the biological activity and initiation of a given mode of action. This consideration goes beyond the aim of the current work to produce conservative and protective estimates and may influence the outcomes of a predictive toxicology analysis aimed at providing a biological basis for the selection of tPODs.

## 4 Conclusion

Overall, this meta-analysis provides evidence that *in vitro* tPODs and AEDs, in the majority of cases, are equal to or more protective estimates when compared to those derived using traditional animal toxicity tests. The results support that there is a high level of correlation between the different approaches evaluated to derive tPODs. However, we caution the use of the fifth percentile for *in vitro* transcriptomics, because of the considerable influence from the top concentrations within these studies on these tPODs. The diversity of chemicals and experimental conditions within the dataset analyzed revealed sources of uncertainties for consideration when shifting to the use of NAM-based data in regulatory applications. Nonetheless, the workflow used here produced multiple AEDs that could be used in screening and prioritization to flag chemicals of greater potential concern for further assessment. Our findings support the current movement transitioning to the use of non-animal test methods in chemical risk assessment, and align with global initiatives that include, but are not limited to, the inter-governmental accelerating the pace of chemical risk assessment (APCRA) collaboration, numerous omics-based initiatives within the OECD, and speciality NAM-based working groups within the Health and Environmental Sciences Institute (HESI). It was demonstrated that transcriptomics reliably detected perturbations in gene expression as a result of chemical exposure within *in vitro* models, outcomes that support the first principle in the proposed logic framework to incorporate omics-based data into the regulatory chemical testing and assessment paradigm (Johnson et al., 2022).

## Data Availability

The data analyzed in this study is subject to the following licenses/restrictions: The datasets included in the current study were all from previously published works. The data was approved to be used and obtained from the original authors and those studies have been directly cited in the current work. Datasets may be made available at the discretion of the original authors. Requests to access these datasets should be directed to OECD, 2022, AR (anthony.reardon@hc-sc.gc.ca); Harrill et al., LE (everett.logan@epa.gov); Ramaiaghari et al., SF (stephen.ferguson@nih.gov); PFAS, Andrea Rowan-Carroll (andrea.rowan-carroll@hc-sc.gc.ca); Buick et al., Julie Buick (julie.buick@hc-sc.gc.ca).
