# Supplementary material for: From vision toward best practices: Evaluating in vitro transcriptomic points of departure for application in risk assessment using a uniform workflow
Source: Front Toxicol. 2023 May 23;5:1194895. doi: 10.3389/ftox.2023.1194895 (PMC10242042; doi:10.3389/ftox.2023.1194895)
Supplement: Supplementary file 1 [file Table1.docx]

**Table 1**. List of chemicals with available in vitro datasets

| **Dataset** | **Chemical Name** | **Abbreviated Name** | **CASRN** | **Model(s)** | **Exposure** (Days) |
| --- | --- | --- | --- | --- | --- |
| OECD 2022 | 2-(4-hydroxyphenyl)sulfonylphenol | 2,4-BPS | 5397-34-2 | MCF-7 | 2 |
| OECD 2022 | 2-[(4-Hydroxyphenyl)methyl]phenol | 2,4-BPF | 2467-03-0 | MCF-7 | 2 |
| OECD 2022 | 2-[4-(Benzyloxy)benzene-1-sulfonyl]phenol | BPS-MPE | 63134-33-8 | MCF-7 | 2 |
| OECD 2022 | 4-(4-hydroxyphenyl)sulfonylphenol | 4,4-BPS | 80-09-1 | MCF-7 | 2 |
| OECD 2022 | 4-[(4-hydroxyphenyl)methyl]phenol | 4,4-BPF | 620-92-8 | MCF-7 | 2 |
| OECD 2022 | 4-((4-Isopropoxyphenyl)sulfonyl)phenol | D8 | 95235-30-6 | MCF-7 | 2 |
| OECD 2022 | 4,4'-Bis(p-tolylsulfonylureido)diphenylmethane | BTUM | 151882-81-4 | MCF-7 | 2 |
| OECD 2022 | 4,4'-Sulfonylbis[2-(prop-2-en-1-yl)phenol] | TGSA | 41481-66-7 | MCF-7 | 2 |
| OECD 2022 | 4-{4-[(Prop-1-en-2-yl)oxy]benzene-1-sulfonyl}phenol | BPS-MAE | 97042-18-7 | MCF-7 | 2 |
| OECD 2022 | Bis (4-chorophenyl) Sulfone | Bis4CPS | 80-07-9 | MCF-7 | 2 |
| OECD 2022 | Bisphenol A | BPA | 80-05-7 | MCF-7 | 2 |
| OECD 2022 | Bisphenol A diglycidyl ether | BADGE | 1675-54-3 | MCF-7 | 2 |
| OECD 2022 | Bisphenol AF | BPAF | 1478-61-1 | MCF-7 | 2 |
| OECD 2022 | Bisphenol AP | BPAP | 1571-75-1 | MCF-7 | 2 |
| OECD 2022 | Bisphenol C | BPC | 14868-03-2 | MCF-7 | 2 |
| OECD 2022 | Dexamethasone | Dex | 50-02-2 | MCF-7 | 2 |
| OECD 2022 | Β-Estradiol | Estradiol | 50-28-2 | MCF-7 | 2 |
| OECD 2022 | Pergafast 201 | Perg201 | 232938-43-1 | MCF-7 | 2 |
| Harrill *et al*. | 3,5,3'-Triiodothyronine | Triiodothyronine | 6893-02-3 | MCF-7 | 0.25 |
| Harrill *et al*. | 4-Cumylphenol |  | 599-64-4 | MCF-7 | 0.25 |
| Harrill *et al*. | 4-Hydroxytamoxifen |  | 68392-35-8 | MCF-7 | 0.25 |
| Harrill *et al*. | 4-Nonylphenol (branched) | 4-Nonylphenol | 84852-15-3 | MCF-7 | 0.25 |
| Harrill *et al*. | Amiodarone hydrochloride | Amiodarone HCl | 19774-82-4 | MCF-7 | 0.25 |
| Harrill *et al*. | Atrazine |  | 1912-24-9 | MCF-7 | 0.25 |
| Harrill *et al*. | Bifenthrin |  | 82657-04-3 | MCF-7 | 0.25 |
| Harrill *et al*. | Bisphenol A | BPA | 80-05-7 | MCF-7 | 0.25 |
| Harrill *et al*. | Bisphenol B | BPB | 77-40-7 | MCF-7 | 0.25 |
| Harrill *et al*. | Butafenacil |  | 134605-64-4 | MCF-7 | 0.25 |
| Harrill *et al*. | Cladribine |  | 4291-63-8 | MCF-7 | 0.25 |
| Harrill *et al*. | Clofibrate |  | 637-07-0 | MCF-7 | 0.25 |
| Harrill *et al*. | Clomiphene citrate (1:1) | Clomiphene Cit | 50-41-9 | MCF-7 | 0.25 |
| Harrill *et al*. | Cyanazine |  | 21725-46-2 | MCF-7 | 0.25 |
| Harrill *et al*. | Cycloheximide |  | 66-81-9 | MCF-7 | 0.25 |
| Harrill *et al*. | Cypermethrin |  | 52315-07-8 | MCF-7 | 0.25 |
| Harrill *et al*. | Cyproconazole |  | 94361-06-5 | MCF-7 | 0.25 |
| Harrill *et al*. | Cyproterone acetate | Cyproterone Ace | 427-51-0 | MCF-7 | 0.25 |
| Harrill *et al*. | Farglitazar |  | 196808-45-4 | MCF-7 | 0.25 |
| Harrill *et al*. | Fenofibrate |  | 49562-28-9 | MCF-7 | 0.25 |
| Harrill *et al*. | Fenpyroximate (Z,E) |  | 111812-58-9 | MCF-7 | 0.25 |
| Harrill *et al*. | Flutamide |  | 13311-84-7 | MCF-7 | 0.25 |
| Harrill *et al*. | Fomesafen |  | 72178-02-0 | MCF-7 | 0.25 |
| Harrill *et al*. | Fulvestrant |  | 129453-61-8 | MCF-7 | 0.25 |
| Harrill *et al*. | Genistein |  | 446-72-0 | MCF-7 | 0.25 |
| Harrill *et al*. | Imazalil |  | 35554-44-0 | MCF-7 | 0.25 |
| Harrill *et al*. | Lactofen |  | 77501-63-4 | MCF-7 | 0.25 |
| Harrill *et al*. | Lovastatin |  | 75330-75-5 | MCF-7 | 0.25 |
| Harrill *et al*. | Maneb |  | 12427-38-2 | MCF-7 | 0.25 |
| Harrill *et al*. | Nilutamide |  | 63612-50-0 | MCF-7 | 0.25 |
| Harrill *et al*. | Perfluorooctanesulfonic acid | PFOS | 1763-23-1 | MCF-7 | 0.25 |
| Harrill *et al*. | Perfluorooctanoic acid | PFOA | 335-67-1 | MCF-7 | 0.25 |
| Harrill *et al*. | Prochloraz |  | 67747-09-5 | MCF-7 | 0.25 |
| Harrill *et al*. | Propiconazole |  | 60207-90-1 | MCF-7 | 0.25 |
| Harrill *et al*. | Pyraclostrobin |  | 175013-18-0 | MCF-7 | 0.25 |
| Harrill *et al*. | Reserpine |  | 50-55-5 | MCF-7 | 0.25 |
| Harrill *et al*. | Rotenone |  | 83-79-4 | MCF-7 | 0.25 |
| Harrill *et al*. | Simazine |  | 122-34-9 | MCF-7 | 0.25 |
| Harrill *et al*. | Simvastatin |  | 79902-63-9 | MCF-7 | 0.25 |
| Harrill *et al*. | Sirolimus |  | 53123-88-9 | MCF-7 | 0.25 |
| Harrill *et al*. | Tetrac |  | 67-30-1 | MCF-7 | 0.25 |
| Harrill *et al*. | Thiram |  | 137-26-8 | MCF-7 | 0.25 |
| Harrill *et al*. | Trichostatin A |  | 58880-19-6 | MCF-7 | 0.25 |
| Harrill *et al*. | Trifloxystrobin |  | 141517-21-7 | MCF-7 | 0.25 |
| Harrill *et al*. | Troglitazone |  | 97322-87-7 | MCF-7 | 0.25 |
| Harrill *et al*. | Vinclozolin |  | 50471-44-8 | MCF-7 | 0.25 |
| Harrill *et al*. | Ziram |  | 137-30-4 | MCF-7 | 0.25 |
| Ramaiahgari *et al*. | Acetaminophen | APAP | 103-90-2 | HepaRG | 4 |
| Ramaiahgari *et al*. | Aflatoxin B1 | AFB1 | 1162-65-8 | HepaRG | 4 |
| Ramaiahgari *et al*. | Aspirin |  | 50-78-2 | HepaRG | 4 |
| Ramaiahgari *et al*. | Benzo(a)pyrene | B[a]P | 50-32-8 | HepaRG | 4 |
| Ramaiahgari *et al*. | Caffeine |  | 58-08-2 | HepaRG | 4 |
| Ramaiahgari *et al*. | Chenodeoxycholic acid | CDCA | 474-25-9 | HepaRG | 4 |
| Ramaiahgari *et al*. | Chlorpromazine | CPZ | 50-53-3 | HepaRG | 4 |
| Ramaiahgari *et al*. | Cyclophosphamide monohydrate | Cyclophosphamide | 6055-19-2 | HepaRG | 4 |
| Ramaiahgari *et al*. | Diphenhydramine hydrochloride | Diphenhydramine | 147-24-0 | HepaRG | 4 |
| Ramaiahgari *et al*. | Fenofibric acid | FFA | 42017-89-0 | HepaRG | 4 |
| Ramaiahgari *et al*. | Levofloxacin hydrate | Levofloxacin | 138199-71-0 | HepaRG | 4 |
| Ramaiahgari *et al*. | Menadione |  | 58-27-5 | HepaRG | 4 |
| Ramaiahgari *et al*. | N-Nitrosodimethylamine | DMN | 62-75-9 | HepaRG | 4 |
| Ramaiahgari *et al*. | Omeprazole | OMP | 73590-58-6 | HepaRG | 4 |
| Ramaiahgari *et al*. | Potassium chloride | KCl | 7447-40-7 | HepaRG | 4 |
| Ramaiahgari *et al*. | Rifampicin | RIF | 13292-46-1 | HepaRG | 4 |
| Ramaiahgari *et al*. | Ritonavir |  | 155213-67-5 | HepaRG | 4 |
| Ramaiahgari *et al*. | Rosiglitazone |  | 122320-73-4 | HepaRG | 4 |
| Ramaiahgari *et al*. | Sucrose |  | 57-50-1 | HepaRG | 4 |
| Ramaiahgari *et al*. | Tamoxifen |  | 10540-29-1 | HepaRG | 4 |
| Ramaiahgari *et al*. | Troglitazone |  | 97322-87-7 | HepaRG | 4 |
| Ramaiahgari *et al*. | Trovafloxacin mesylate | Trovafloxacin | 147059-75-4 | HepaRG | 4 |
| Ramaiahgari *et al*. | Valproic acid | VPA | 99-66-1 | HepaRG | 4 |
| Ramaiahgari *et al*. | Phenobarbital sodium | PB | 57-30-7 | HepaRG | 4 |
| PFAS | 2H,2H,3H,3H-Perfluorooctanoic acid | 5:3 Acid | 914637-49-3 | Spheroids | 1, 10 |
| PFAS | 4:2 Fluorotelomer sulfonic acid | 4:2 FtS | 757124-72-4 | Spheroids | 1, 10 |
| PFAS | 6:2 Fluorotelomer alcohol | 6:2 FtOH | 647-42-7 | Spheroids | 1, 10 |
| PFAS | 6:2 Fluorotelomer phosphate monoester | 6:2 monoPAP | 57678-01-0 | Spheroids | 1, 10 |
| PFAS | 6:2 Fluorotelomer sulfonic acid | 6:2 FtS | 27619-97-2 | Spheroids | 1, 10 |
| PFAS | 8:2 Fluorotelomer alcohol | 8:2 FtOH | 678-39-7 | Spheroids | 1, 10 |
| PFAS | 8:2 Fluorotelomer phosphate monoester | 8:2 monoPAP | 57678-03-2 | Spheroids | 1, 10 |
| PFAS | 8:2 Fluorotelomer sulfonic acid | 8:2 FtS | 39108-34-4 | Spheroids | 1, 10 |
| PFAS | Perfluorobutanesulfonic acid | PFBS | 375-73-5 | Spheroids | 1, 4, 10, 14 |
| PFAS | Perfluorobutanoic acid | PFBA | 375-22-4 | Spheroids | 1, 10 |
| PFAS | Perfluorodecanesulfonic acid | PFDS | 335-77-3 | Spheroids | 1, 4, 10, 14 |
| PFAS | Perfluorodecanoic acid | PFDA | 335-76-2 | Spheroids | 1, 10 |
| PFAS | Perfluoroheptanesulfonic acid | PFHpS | 375-92-8 | Spheroids | 1, 10 |
| PFAS | Perfluoroheptanoic acid | PFHpA | 375-85-9 | Spheroids | 1, 10 |
| PFAS | Perfluorohexanesulfonic acid | PFHxS | 355-46-4 | Spheroids | 1, 10 |
| PFAS | Perfluorohexanoic acid | PFHxA | 307-24-4 | Spheroids | 1, 10 |
| PFAS | Perfluorononanoic acid | PFNA | 375-95-1 | Spheroids | 1, 10 |
| PFAS | Perfluorooctanoic acid | PFOA | 335-67-1 | Spheroids | 1, 4, 10, 14 |
| PFAS | Perfluorooctanesulfonic acid | PFOS | 1763-23-1 | Spheroids | 1, 4, 10, 14 |
| PFAS | Perfluorooctanesulfonamide | PFOSA | 754-91-6 | Spheroids | 1, 10 |
| PFAS | Perfluoropentanoic acid | PFPeA | 2706-90-3 | Spheroids | 1, 10 |
| PFAS | Perfluorotetradecanoic acid | PFTeDA | 376-06-7 | Spheroids | 1, 10 |
| PFAS | Perfluoroundecanoic acid | PFUnA | 2058-94-8 | Spheroids | 1, 10 |
| Buick *et al*. | 2-Deoxy-D-glucose | 2DD-Glucose | 154-17-6 | HepaRG | 2 |
| Buick *et al*. | Aflatoxin B1 | AFB1 | 1162-65-8 | HepaRG | 2 |
| Buick *et al*. | Benzo(a)pyrene | B[a]P | 50-32-8 | HepaRG | 2 |
| Buick *et al*. | Cisplatin |  | 15663-27-1 | HepaRG | 2 |
| Buick *et al*. | Cyclophosphamide monohydrate | Cyclophosphamide | 6055-19-2 | HepaRG | 2 |
| Buick *et al*. | Cytosine arabinoside | Cyt arabinoside | 147-94-4 | HepaRG | 2 |
| Buick *et al*. | Eugenol |  | 97-53-0 | HepaRG | 2 |
| Buick *et al*. | Methyl methanesulfonate | M-mSulfonate | 66-27-3 | HepaRG | 2 |
| Buick *et al*. | N-Nitroso-N-ethylurea | N-Nitrosourea | 759-73-9 | HepaRG | 2 |
| Buick *et al*. | Propyl gallate |  | 121-79-9 | HepaRG | 2 |
| Buick *et al*. | Urea |  | 57-13-6 | HepaRG | 2 |
| Buick *et al*. | Zidovudine (azidothymidine) |  | 30516-87-1 | HepaRG | 2 |

OECD 2022 – Listing of Bisphenols from Health Canada OECD Case-study, Harrill *et al*. – Listing of chemicals from published dataset in Harrill *et al*. 2021, Ramaiahgari *et al*. – Listing of chemicals from published dataset in Ramaiahgari *et al*. 2019, PFAS – Listing of chemicals from published datasets in Reardon *et al.* 2021 and Rowan-Carroll *et al*. 2021, Buick *et al*. – Listing of chemicals from published dataset in Buick *et al*. 2022.

**Table 2**. List of chemicals with available in vivo datasets

|  | **Name** | **CASRN** | **Animal** | **Study Type** | **Method** | **Effect Level**  (mg/kg-bw/day) |
| --- | --- | --- | --- | --- | --- | --- |
| **EPA ToxVal** | 4-Cumylphenol | 599-64-4 | Rat | Repeat Dose | NOAEL | 50 |
|  | AFB1 | 1162-65-8 | Human | Repeat Dose | BMDL01 | 0.000078 |
|  | Atrazine | 1912-24-9 | Mouse | Reproductive | NOEL | 0.001 |
|  | Bifenthrin | 82657-04-3 | Rat | Developmental | NOAEL | 1 |
|  | Bis4CPS | 620-92-8 | Rat | Repeat Dose | LOEL | 20 |
|  | BPA | 80-05-07 | Rat | Developmental | NOAEL | 0.015 |
|  | Cyanazine | 21725-46-2 | Rat | Repeat Dose | NOAEL | 0.005 |
|  | Cyclophosphamide | 6055-19-2 |  | Repeat Dose | Cancer | 0.57 |
|  | Cyproterone ace | 427-51-0 | Mouse | Repeat Dose | NOAEL | 125 |
|  | Eugenol | 97-53-0 | Rat | Repeat Dose | NOAEL | 57 |
|  | Fenofibrate | 49562-28-9 | Mouse | Developmental | LEL | 11.7 |
|  | Flutamide | 13311-84-7 | Rat | Repeat Dose | NOAEL | 10 |
|  | Genistein | 446-72-0 | Rat | Developmental | NEL | 20 |
|  | Lovastatin | 75330-75-5 | Mouse | Repeat Dose | NOAEL | 30 |
|  | PFHxA | 307-24-4 | Rat | Repeat Dose | NOAEL | 200 |
|  | PFOA | 335-67-1 | Mouse | Reproductive | LOEL | 0.02 |
|  | Prochloraz | 67747-09-5 | Dog | Repeat Dose | NOAEL | 2.5 |
|  | Propiconazole | 60207-90-1 | Mouse | Repeat Dose | NOAEL | 2.7 |
|  | Pyraclostrobin | 175013-18-0 | Rat | Repeat Dose | NOAEL | 3.4 |
|  | Reserpine | 50-55-5 | Mouse | Repeat Dose | NOAEL | 0.12 |
|  | Rotenone | 83-79-4 | Rat | Developmental | LOAEL | 0.75 |
|  | Simazine | 122-34-9 | Mouse | Reproductive | NOEL | 0.005 |
|  | Trifloxystrobin | 141517-21-7 | Rabbit | Developmental | NOAEL | 10 |
|  | Troglitazone | 97322-87-7 | Mouse | Repeat Dose | NOAEL | 1200 |
|  | Vinclozolin | 50471-44-8 | mouse | Reproductive | LOEL | 1 |
|  | Zidovudine | 30516-87-1 | Mouse | Repeat Dose | LEL | 100 |
| **REACH** | 2DD-Glucose | 66-13-6 | rat | Reproductive | NOAEL | 20 |
|  | BPAF | 1478-61-1 | Rat | Repeat Dose | NOAEL | 3.5 |
|  | BPS | 80-09-01 | Rat | Reproductive / Developmental | NOAEL | 10 |
|  | Cyproconazole | 94361-06-5 | Rat | Reproductive | NOAEL | 1 |
|  | Estradiol | 50-28-2 | Rabbit | Developmental | NOEL | 0.0003 |
|  | Lactofen | 77501-63-4 | Rat | Reproductive | NOEL | 2.5 |
|  | TGSA | 41481-66-7 | Rat | Repeat Dose | NOEL | 15 |
|  | Thiram | 137-26-8 | Dog | Repeat Dose | NOEL | 0.84 |
|  | Urea | 57-13-6 |  | Developmental | NOAEL | 500 |
| **OECD** | B[a]P | 50-32-8 |  | Repeat Dose | LOEL | 0.05 |
|  | Fenpyroximate | 111812-58-9 |  | Reproductive | LOEL | 8.45 |
|  | Imazalil | 35554-44-0 |  | Reproductive | NOAEL | 5 |
|  | PFOS | 1763-23-1 |  | Reproductive | NOAEL | 0.03 |
| **HC** | Propyl gallate | 121-79-9 |  | Repeat Dose | NOAEL | 135 |

**Table 3**. Summary table of advantages, disadvantages, and results of evaluated approaches for derivation of transcriptomic points of departure

|  | **Result** | **Advantages** | **Disadvantages** |
| --- | --- | --- | --- |
| 5^th^ percentile | The 5^th^ percentile is a conservative value for tPOD derivation that targets the lowest and most responsive genes.  Produces the highest potency ranking for select substances, even for chemicals identified as ‘non-toxic’ reference chemicals.  Not considered as a reliable approach to derive tPODs using gene expression data. | Simplified calculation of estimate that uses the 5^th^ percentile gene from the full distribution of genes with modeled BMCs.  No additional models or calculations are required beyond BMD modeling software. | Subject to influence from the experimental design (e.g., the top dose/concentration).  Subject to influence by the extent of transcriptional change (i.e., total number of genes with BMCs). |
| 1^st^ Mode^a^ | The 1^st^ mode uses those genes from the identified first mode based on the frequency of distribution of genes with BMCs.  Considered the least conservative but is a consistent and simple approach to derive tPODs using distributions. | Consistently low (i.e., conservative) estimate across majority of listed chemicals.  Corresponds with disrupted biomolecular targets and pathways, and provides an estimate of the initiation of molecular events (Judson et al., 2016). | Requires sufficient biological activity (i.e., genes with BMCs) for identification and calculation of modes. |
| 25^th^ Ranked Gene^a^ | The 25^th^ ranked gene is set as a threshold that is identified from a relative potency ranking of the lowest to highest genes with BMCs.  Considered a simple approach deriving tPODs using the BMC distribution that represents approximately 0.1 % of the genome. | Simplified calculation using the 25^th^ ranked gene by potency based on the distribution of genes with modeled BMCs.  No additional models or calculations are required beyond BMD modeling software. | Requires a minimal amount of biological activity (e.g., 25 concentration-responsive genes).  Excludes select chemicals with insufficient biological activity (e.g., either non-liver toxic, or highly cytotoxic chemicals that do not meet a minimum required concentration-responsive genes). |
| LCRD^a^ | The LCRD is the value of the lowest of rank-ordered BMCs using consistent response groups of BMCs (Crizer et al., 2021)  Represents the primary approach to derive conservative tPODs for most of the listed chemicals that were carried forward for comparison with apical PODs | Uses a calculated derivation for tPOD derivation that represents a point of toxicological relevance that is considered a consistent response of all biological features  Predominantly the lowest and most conservative tPOD of all approaches based on the distribution of BMCs across all chemicals, and suggested as a promising candidate for deriving protective and conservative tPODs | Requires more complex modeling to obtain a relevant tPOD based on the literature |
| Lowest Gene Set^a^ | The lowest and most sensitive gene set is widely accepted as a means of obtaining a tPOD as outlined in a guidance document (National Toxicology Program, 2018).  Reliable approach in scenarios with sufficient biological activity to derive tPODs using the median value of the lowest and most potent (i.e., sensitive) gene set.  tPODs primarily defined using annotations from the largest and most comprehensive database (e.g., GO). | Established and reliable approach to derive tPODs using dose-response models that reflect meaningful changes in biology while reducing the influence of background.  Option to choose from multiple available databases to represent annotations used to define gene sets (e.g., GO, KEGG or REACTOME). | Requires more complex modeling and additional parameters and filtering to obtain a relevant tPOD based gene sets.  Requires sufficient biological activity (i.e., genes with BMCs) to define subsets of genes related to gene sets and pathways. |

^a^Approaches that were considered to be alternatives to be applied in lieu of approaches using percentiles (e.g., 5th) to derive a transcriptomic point of departure.
