# Supplementary material for: From vision toward best practices: Evaluating in vitro transcriptomic points of departure for application in risk assessment using a uniform workflow
Source: Front Toxicol. 2023 May 23;5:1194895. doi: 10.3389/ftox.2023.1194895 (PMC10242042; doi:10.3389/ftox.2023.1194895)
Supplement: Supplementary file 2 [file DataSheet1.ZIP › Reardon_Supporting Information/Appendix_2_PODPlots.pdf]

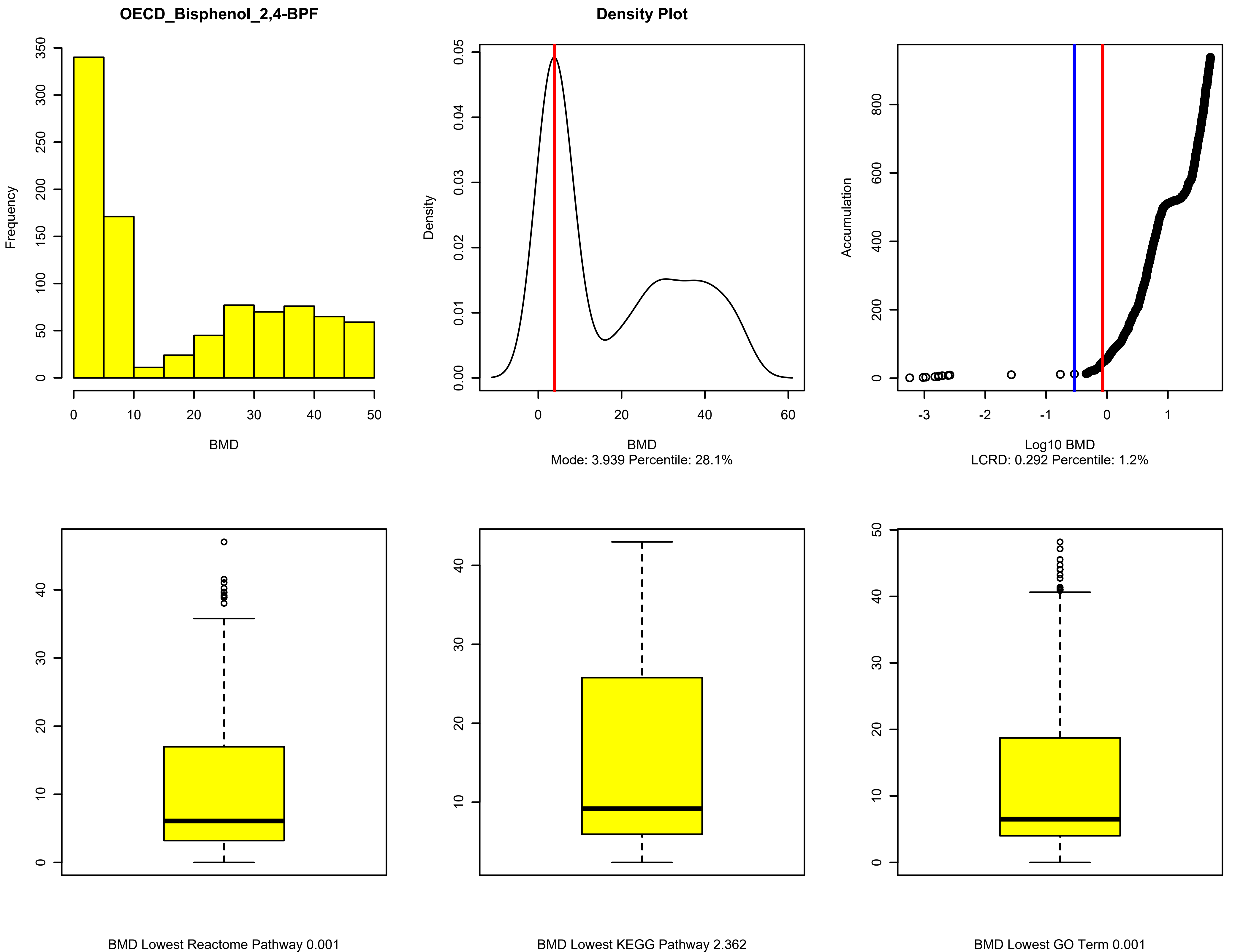
OECD_Bisphenol_2,4-BPF
Frequency
BMD
Density Plot
Density
BMD
Mode: 3.939 Percentile: 28.1%
Accumulation
Log10 BMD
LCRD: 0.292 Percentile: 1.2%
BMD Lowest Reactome Pathway 0.001
BMD Lowest KEGG Pathway 2.362
BMD Lowest GO Term 0.001

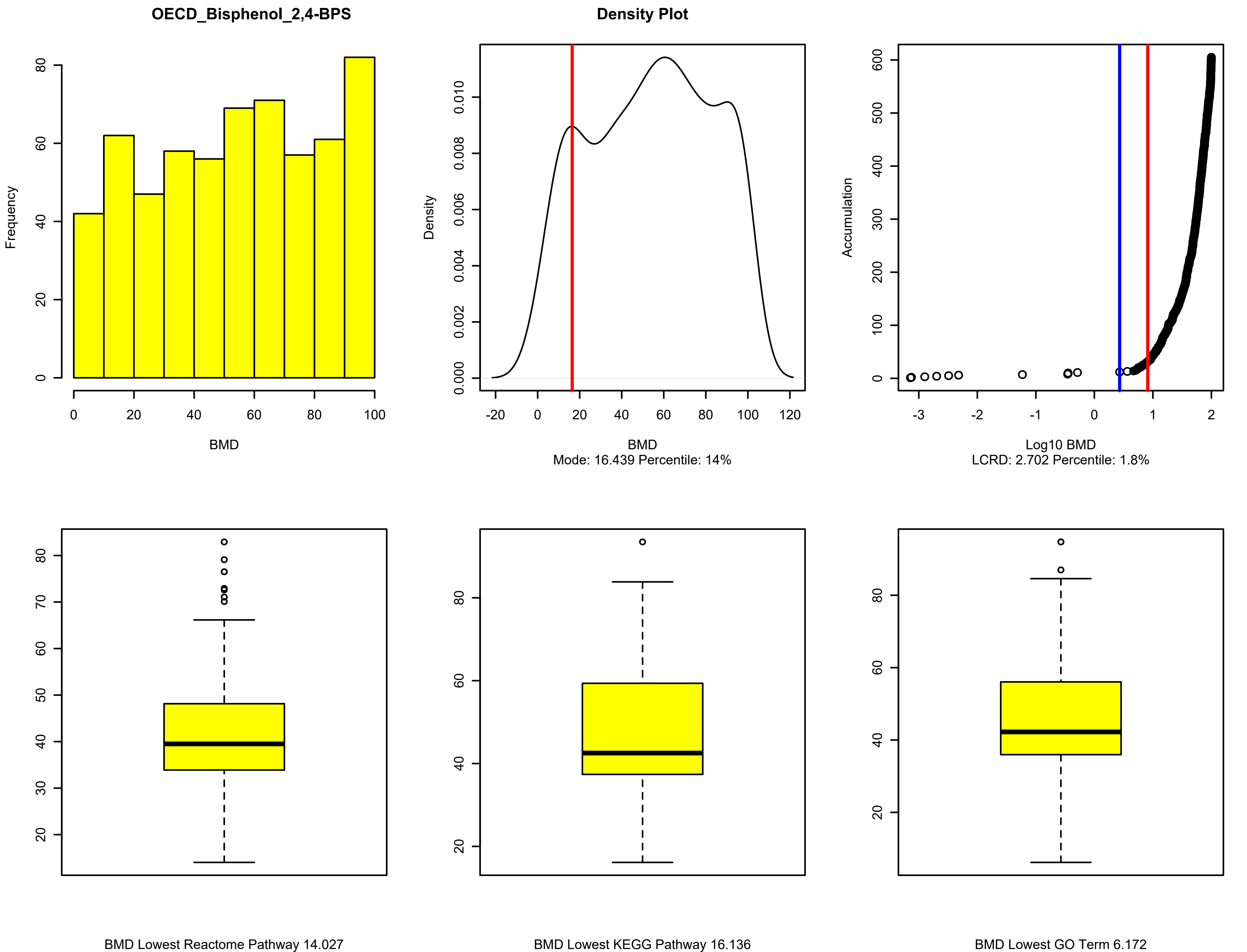
OECD_Bisphenol_2,4-BPS
Frequency
0
20
40
60
80
0
20
40
60
80
100
BMD
Density Plot
Density
0.000
0.002
0.004
0.006
0.008
0.010
-20
0
20
40
60
80
100
120
BMD
Mode: 16.439 Percentile: 14%
Accumulation
0
100
200
300
400
500
600
-3
-2
-1
0
1
2
Log10 BMD
LCRD: 2.702 Percentile: 1.8%
20
30
40
50
60
70
80
BMD Lowest Reactome Pathway 14.027
20
40
60
80
BMD Lowest KEGG Pathway 16.136
20
40
60
80
BMD Lowest GO Term 6.172

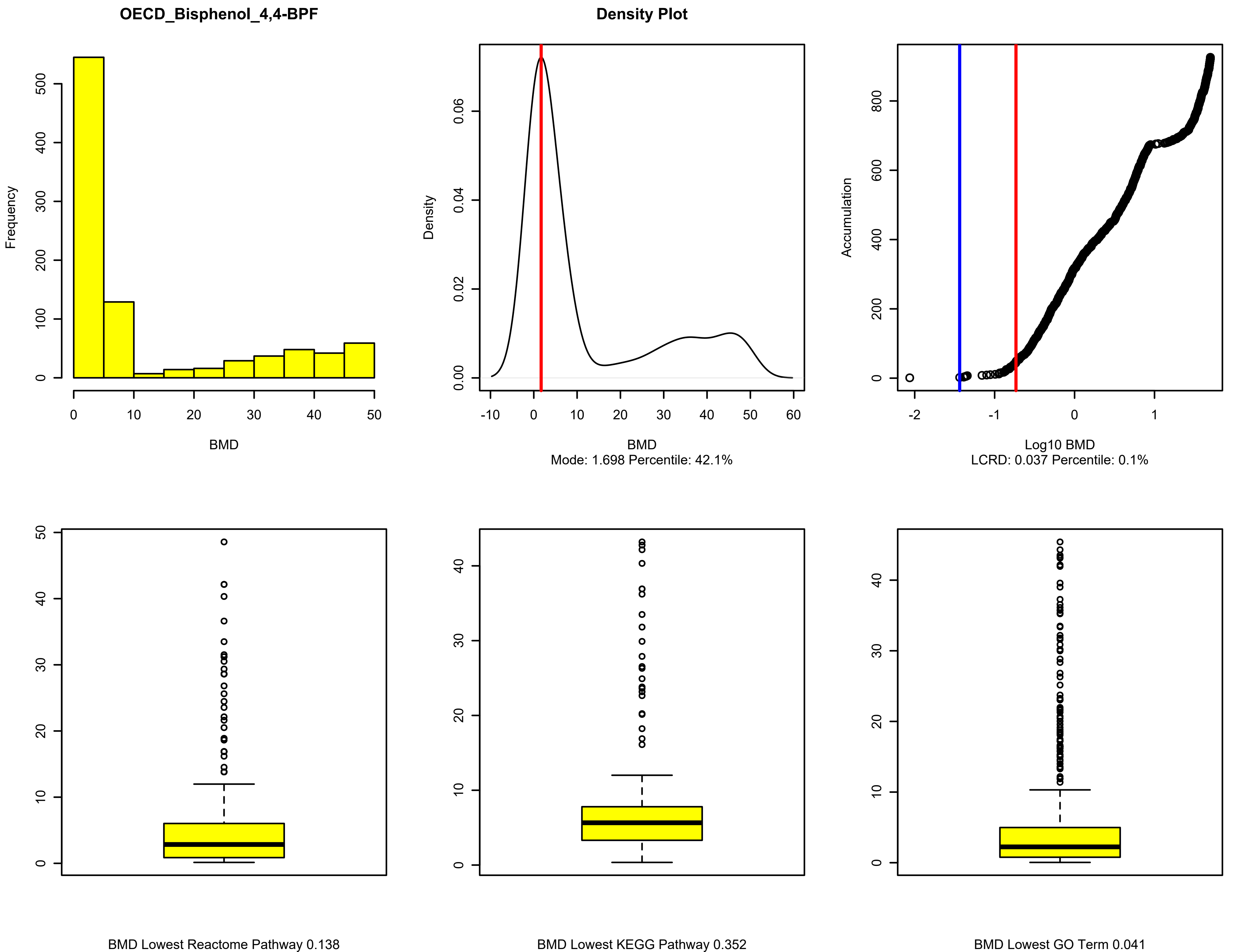
OECD_Bisphenol_4,4-BPF
Frequency
BMD
Density Plot
Density
BMD
Mode: 1.698 Percentile: 42.1%
Accumulation
Log10 BMD
LCRD: 0.037 Percentile: 0.1%
BMD Lowest Reactome Pathway 0.138
BMD Lowest KEGG Pathway 0.352
BMD Lowest GO Term 0.041

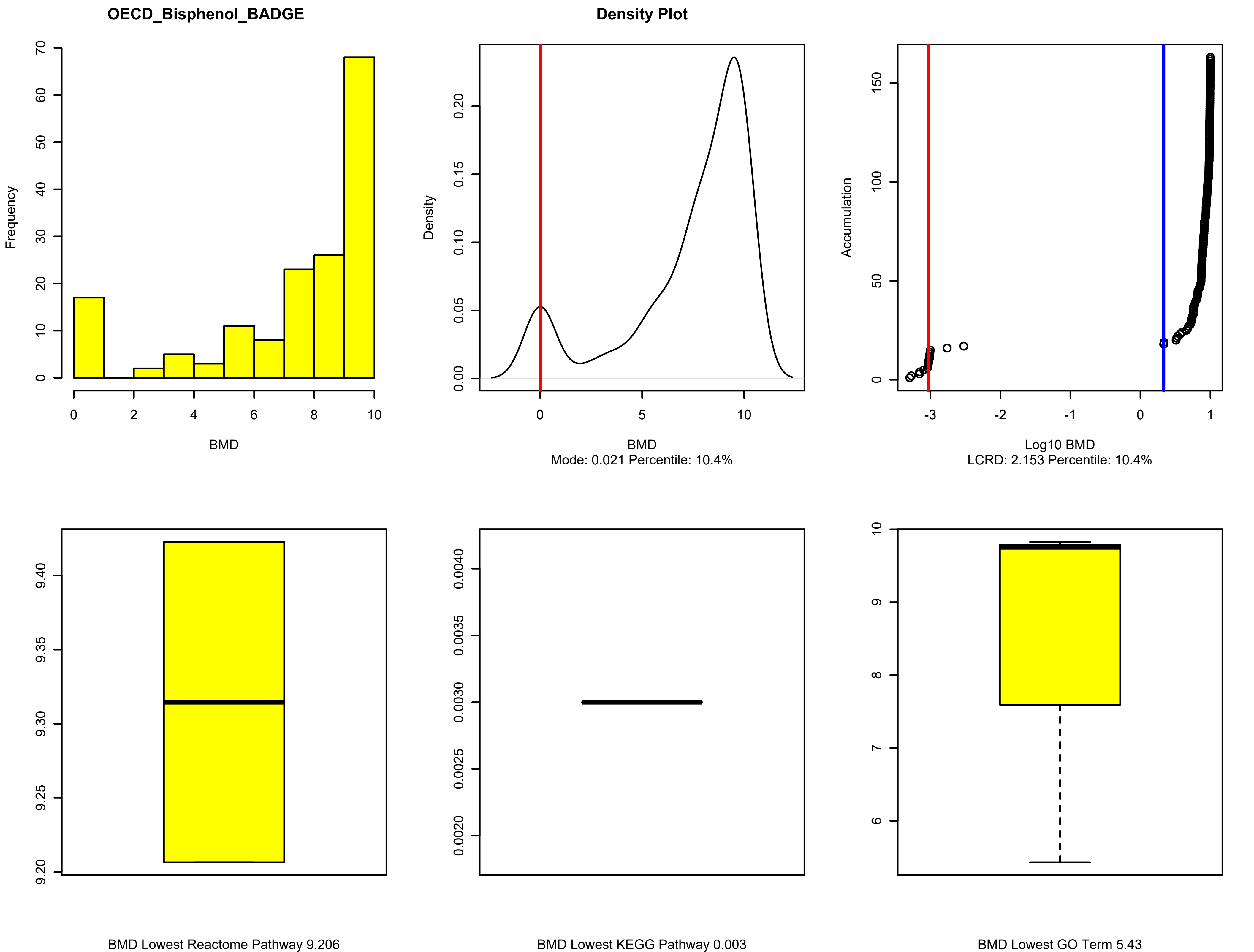
OECD_Bisphenol_BADGE
Frequency
BMD
Density Plot
Density
BMD
Mode: 0.021 Percentile: 10.4%
Accumulation
Log10 BMD
LCRD: 2.153 Percentile: 10.4%
BMD Lowest Reactome Pathway 9.206
BMD Lowest KEGG Pathway 0.003
BMD Lowest GO Term 5.43

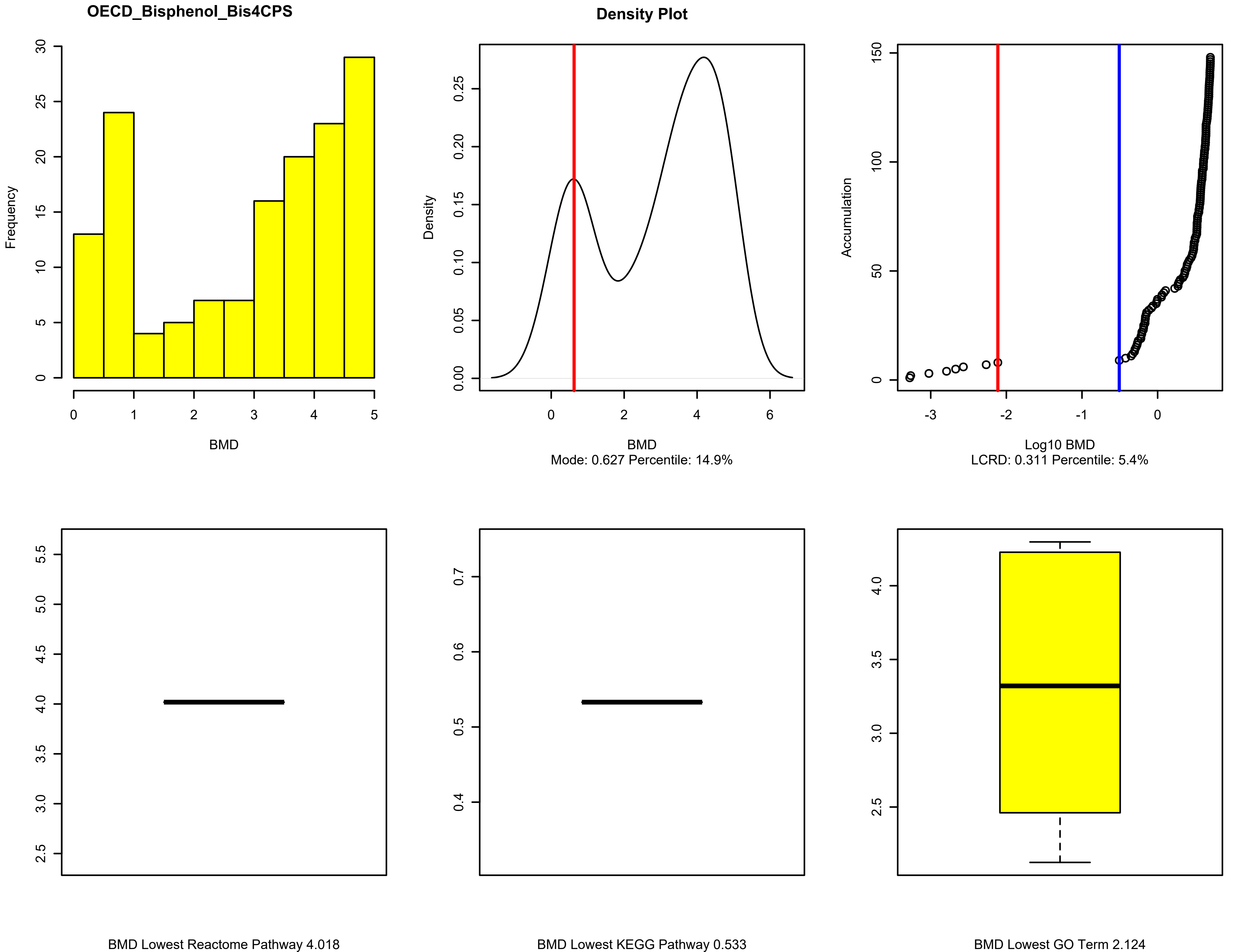
OECD_Bisphenol_Bis4CPS
Frequency
BMD
Density Plot
Density
BMD
Mode: 0.627 Percentile: 14.9%
Accumulation
Log10 BMD
LCRD: 0.311 Percentile: 5.4%
BMD Lowest Reactome Pathway 4.018
BMD Lowest KEGG Pathway 0.533
BMD Lowest GO Term 2.124

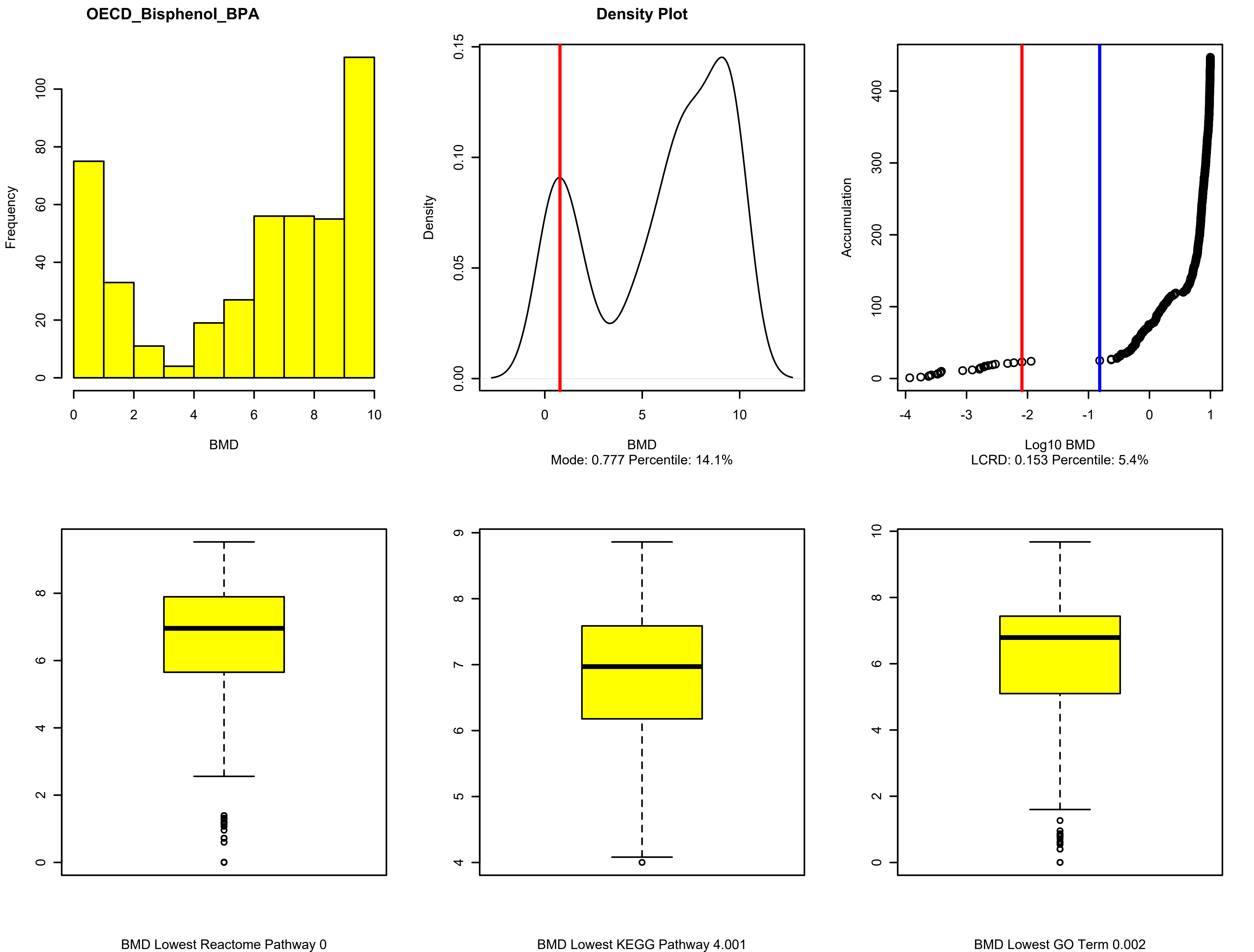

OECD_Bisphenol_BPA
Frequency
BMD
Density Plot
Density
BMD
Mode: 0.777 Percentile: 14.1%
Accumulation
Log10 BMD
LCRD: 0.153 Percentile: 5.4%
BMD Lowest Reactome Pathway 0
BMD Lowest KEGG Pathway 4.001
BMD Lowest GO Term 0.002

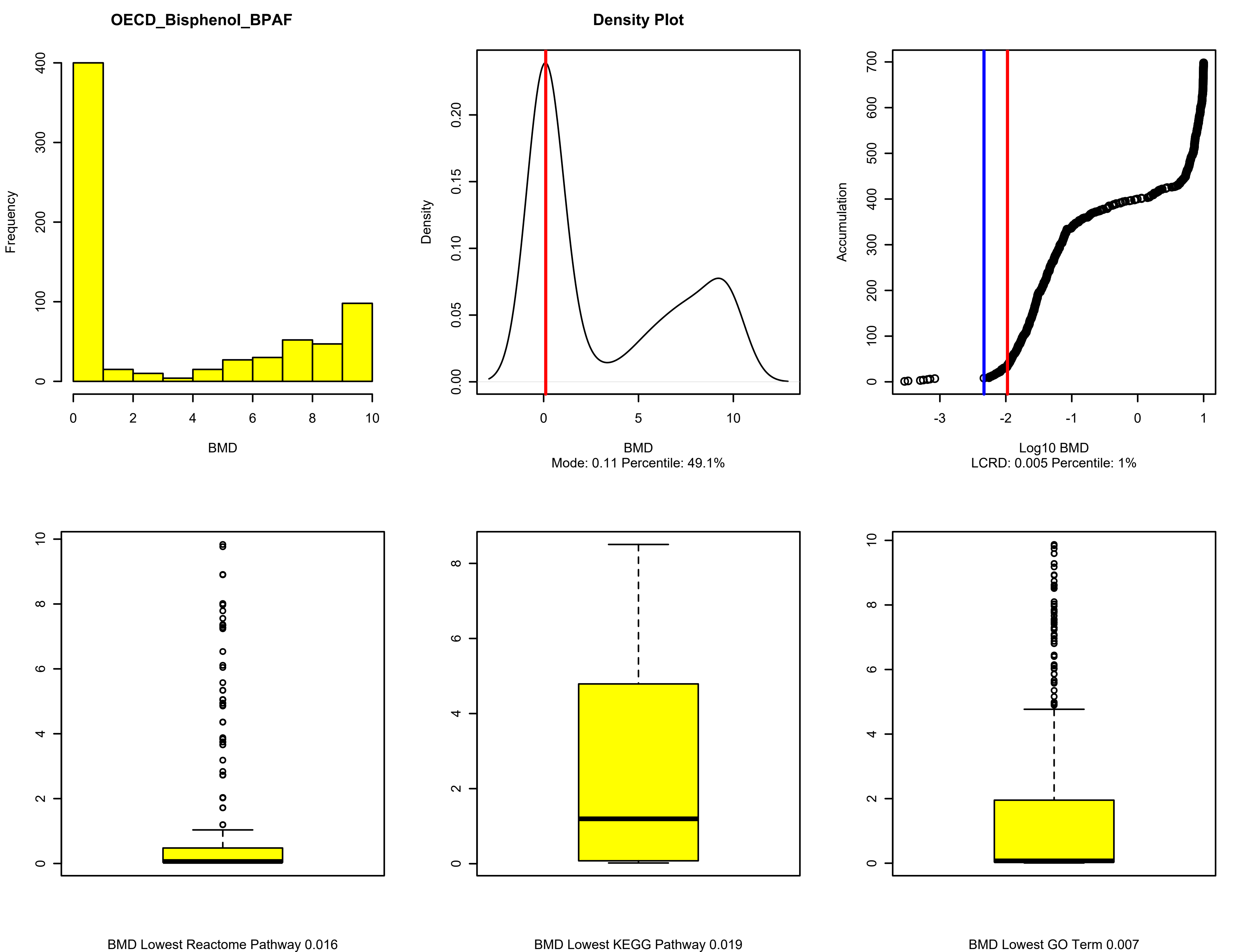
OECD_Bisphenol_BPAF
Frequency
BMD
Density Plot
Density
BMD
Mode: 0.11 Percentile: 49.1%
Accumulation
Log10 BMD
LCRD: 0.005 Percentile: 1%
BMD Lowest Reactome Pathway 0.016
BMD Lowest KEGG Pathway 0.019
BMD Lowest GO Term 0.007

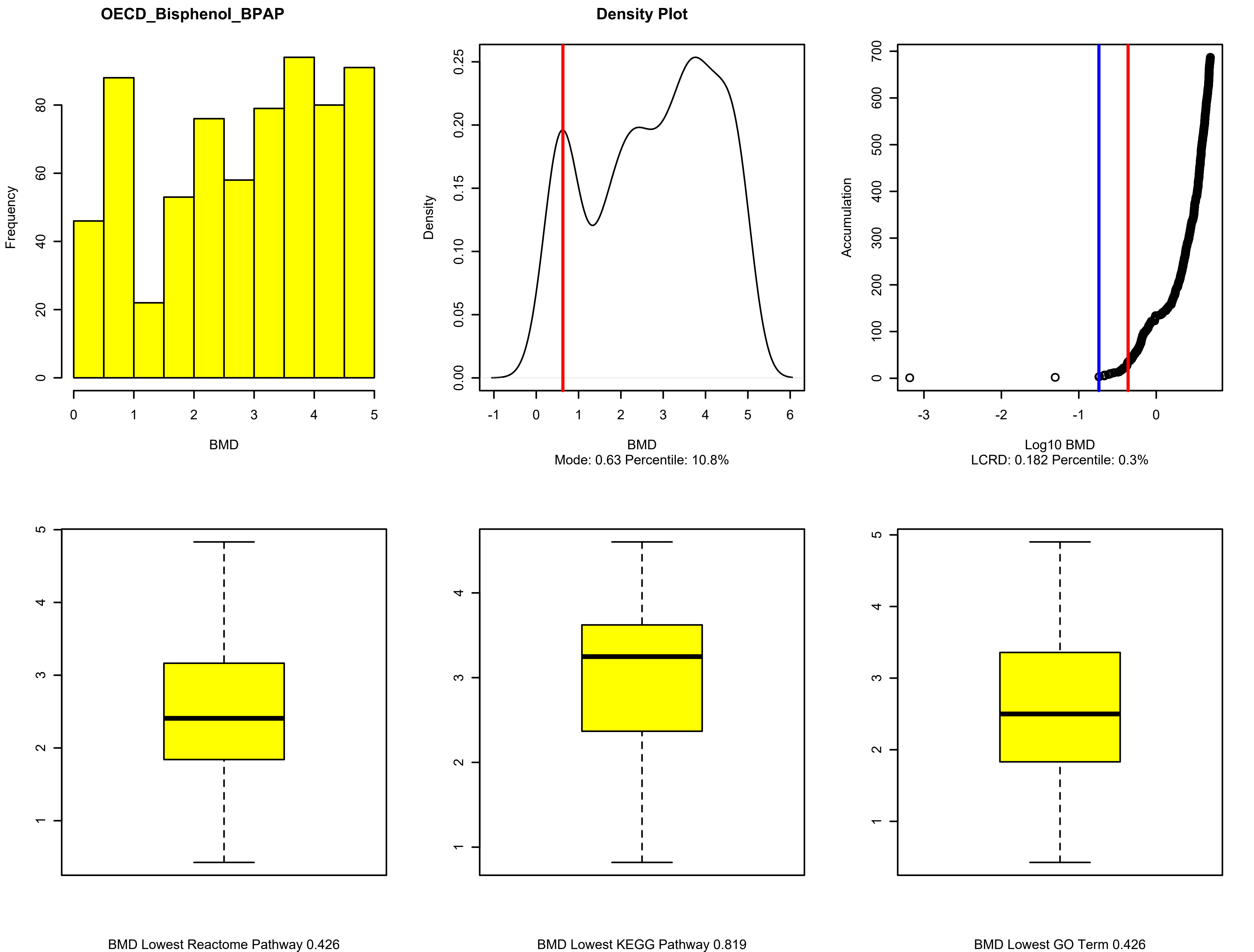

OECD_Bisphenol_BPAP
Frequency
BMD
Density Plot
Density
BMD
Mode: 0.63 Percentile: 10.8%
Accumulation
Log10 BMD
LCRD: 0.182 Percentile: 0.3%
BMD Lowest Reactome Pathway 0.426
BMD Lowest KEGG Pathway 0.819
BMD Lowest GO Term 0.426

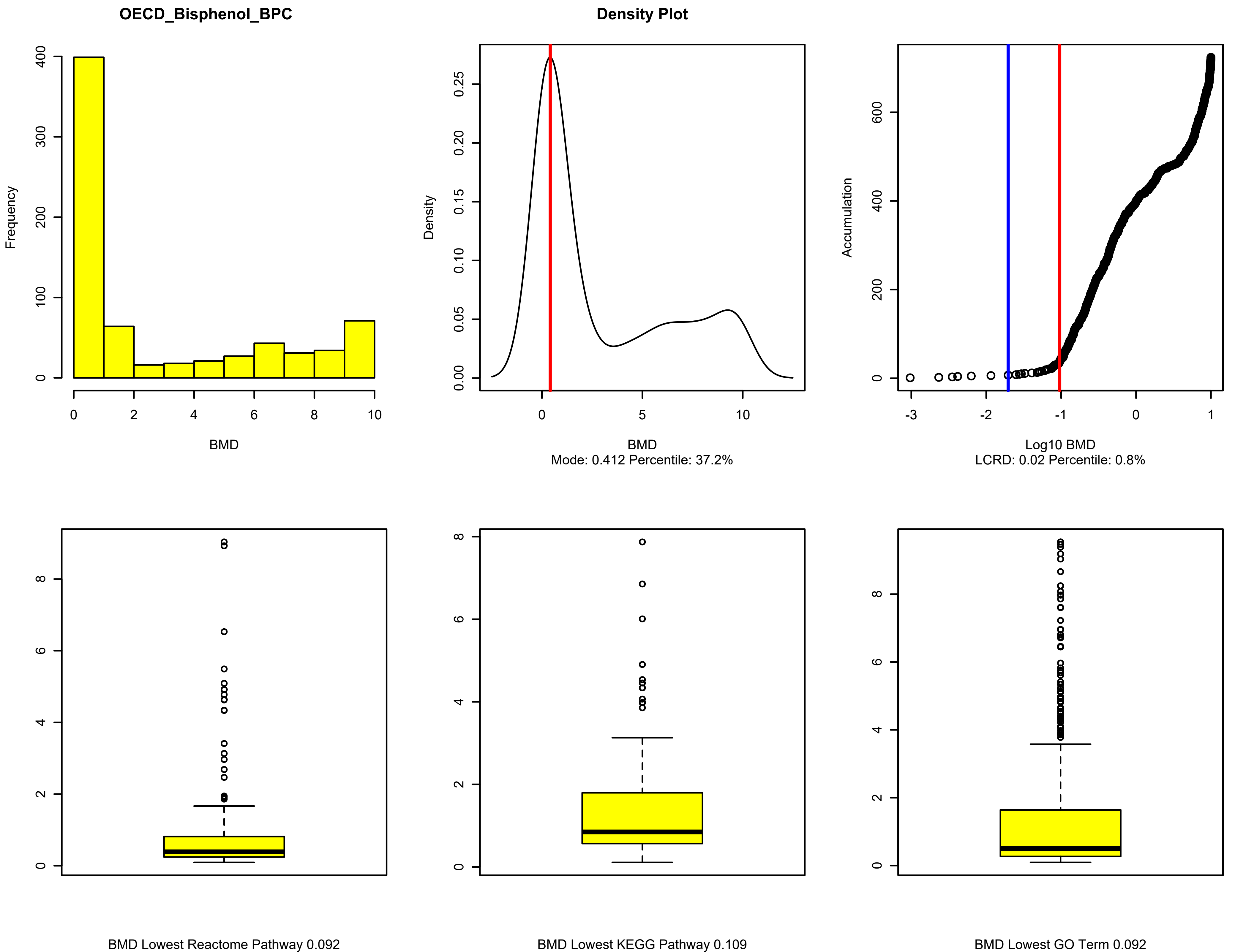
OECD_Bisphenol_BPC
Frequency
BMD
Density Plot
Density
BMD
Mode: 0.412 Percentile: 37.2%
Accumulation
Log10 BMD
LCRD: 0.02 Percentile: 0.8%
BMD Lowest Reactome Pathway 0.092
BMD Lowest KEGG Pathway 0.109
BMD Lowest GO Term 0.092

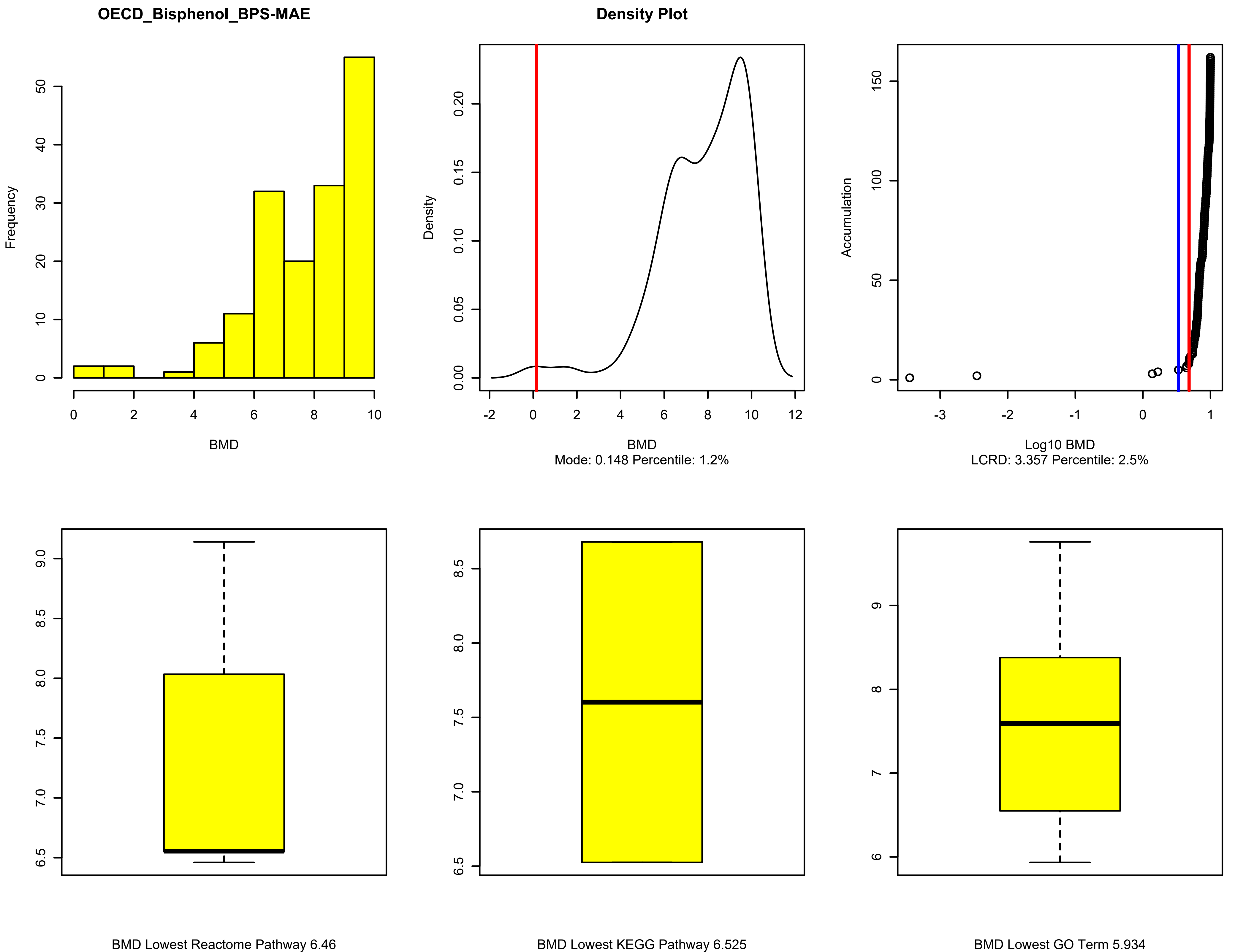
OECD_Bisphenol_BPS-MAE
Density Plot
Frequency
BMD
Density
BMD
Mode: 0.148 Percentile: 1.2%
Accumulation
Log10 BMD
LCRD: 3.357 Percentile: 2.5%
BMD Lowest Reactome Pathway 6.46
BMD Lowest KEGG Pathway 6.525
BMD Lowest GO Term 5.934

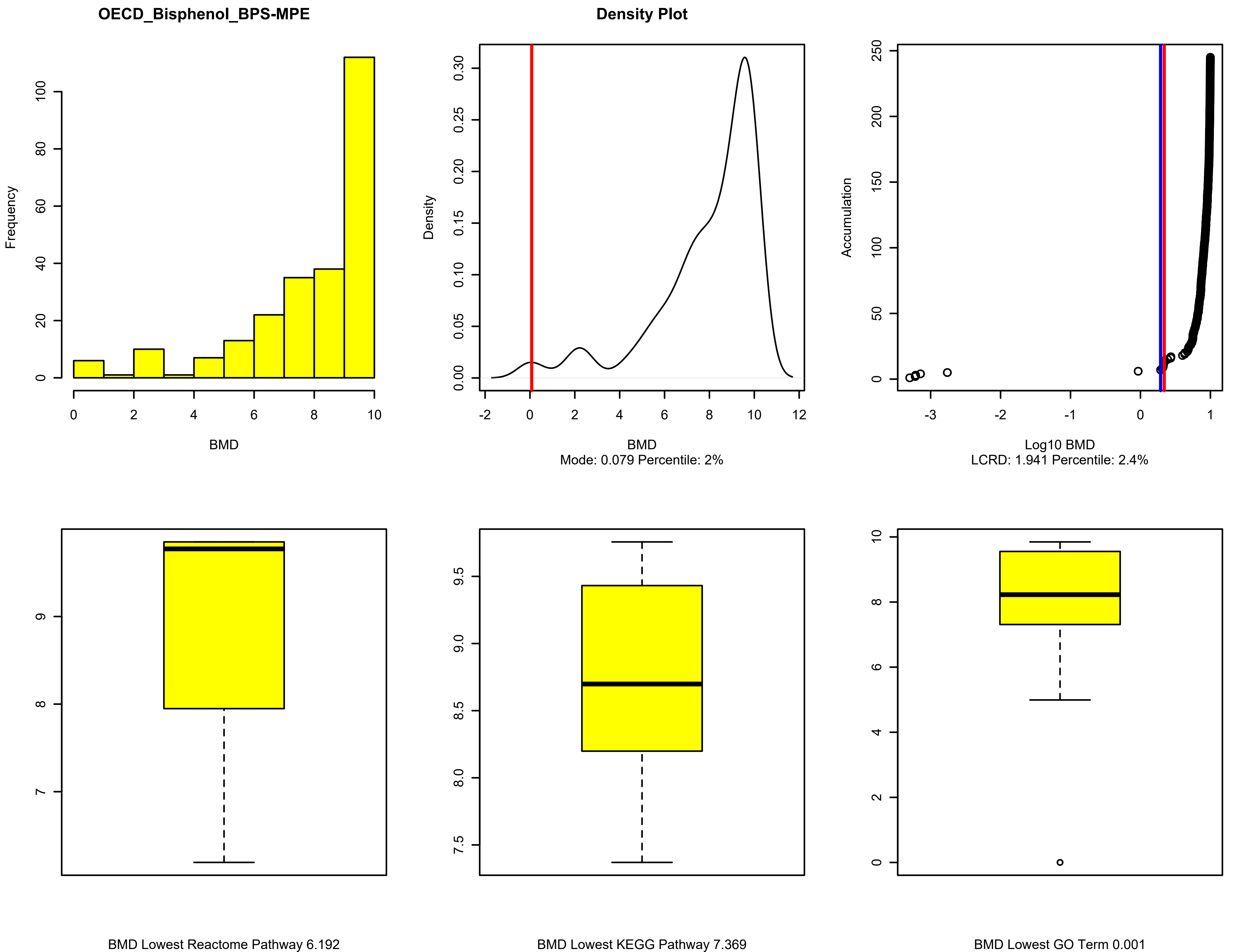
OECD_Bisphenol_BPS-MPE
Frequency
BMD
Density Plot
Density
BMD
Mode: 0.079 Percentile: 2%
Accumulation
Log10 BMD
LCRD: 1.941 Percentile: 2.4%
BMD Lowest Reactome Pathway 6.192
BMD Lowest KEGG Pathway 7.369
BMD Lowest GO Term 0.001

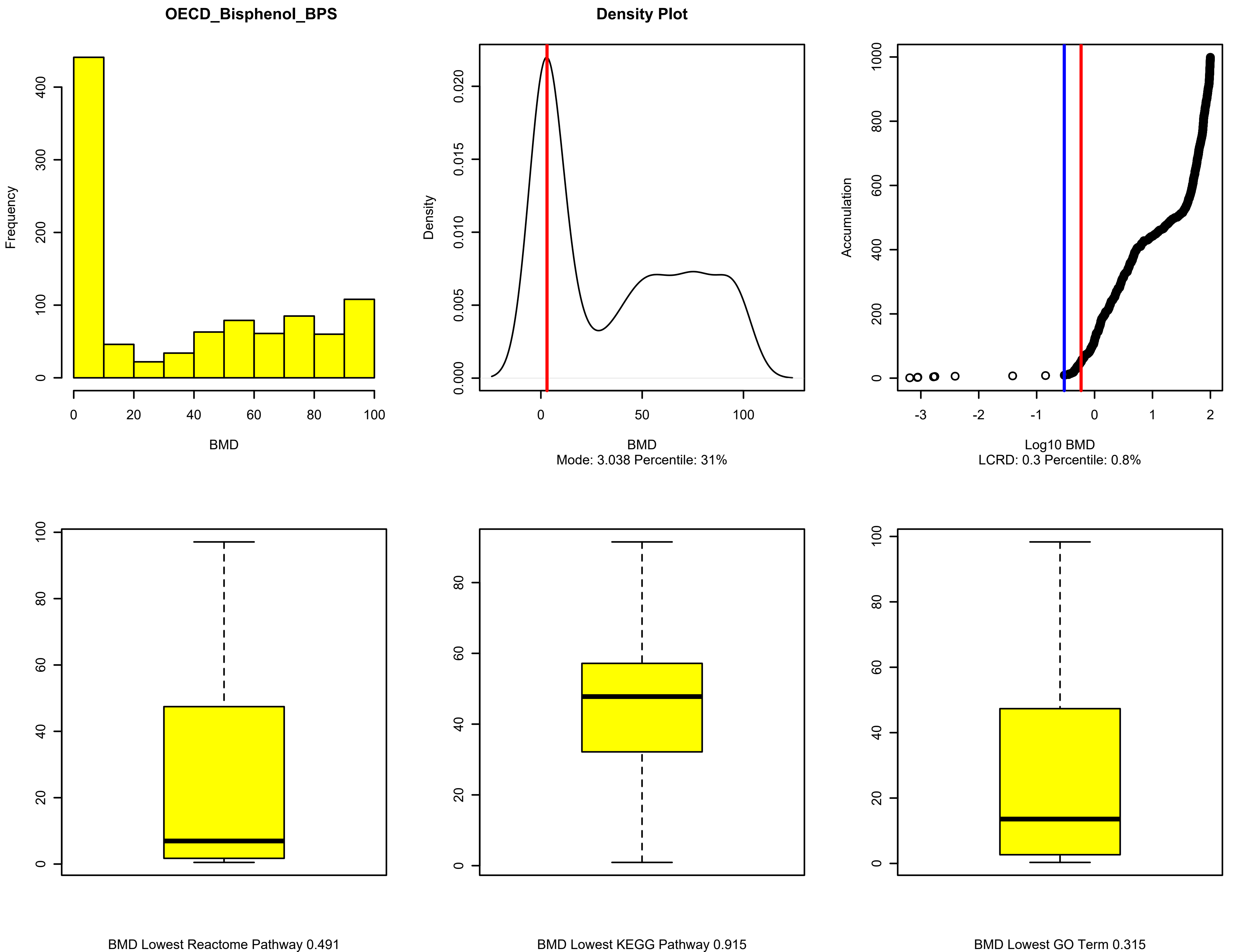
OECD_Bisphenol_BPS
Frequency
BMD
Density Plot
Density
BMD
Mode: 3.038 Percentile: 31%
Accumulation
Log10 BMD
LCRD: 0.3 Percentile: 0.8%
BMD Lowest Reactome Pathway 0.491
BMD Lowest KEGG Pathway 0.915
BMD Lowest GO Term 0.315

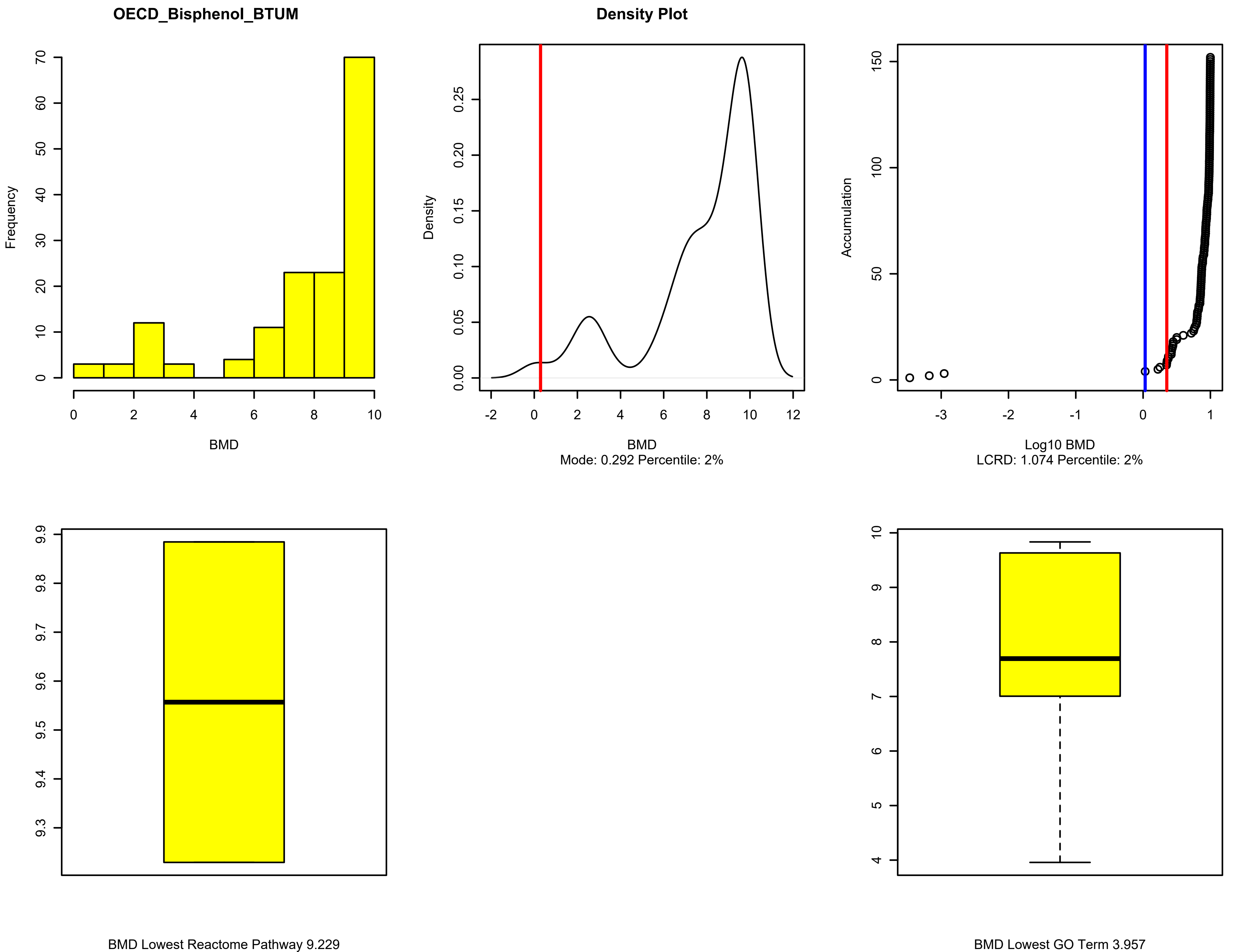

OECD_Bisphenol_BTUM
Frequency
BMD
Density Plot
Density
BMD
Mode: 0.292 Percentile: 2%
Accumulation
Log10 BMD
LCRD: 1.074 Percentile: 2%
BMD Lowest Reactome Pathway 9.229
BMD Lowest GO Term 3.957

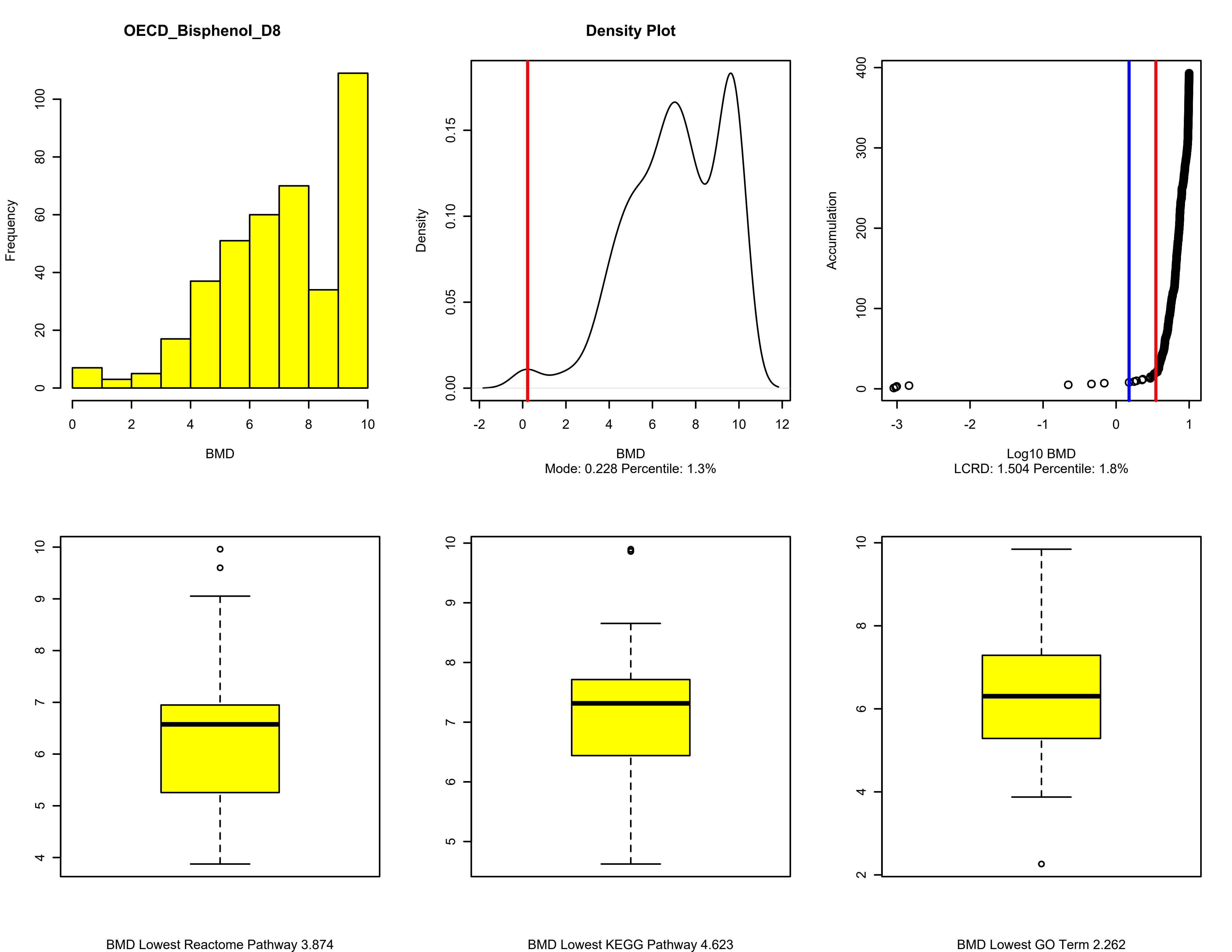

OECD_Bisphenol_D8
Frequency
BMD
Density Plot
Density
BMD
Mode: 0.228 Percentile: 1.3%
Accumulation
Log10 BMD
LCRD: 1.504 Percentile: 1.8%
BMD Lowest Reactome Pathway 3.874
BMD Lowest KEGG Pathway 4.623
BMD Lowest GO Term 2.262

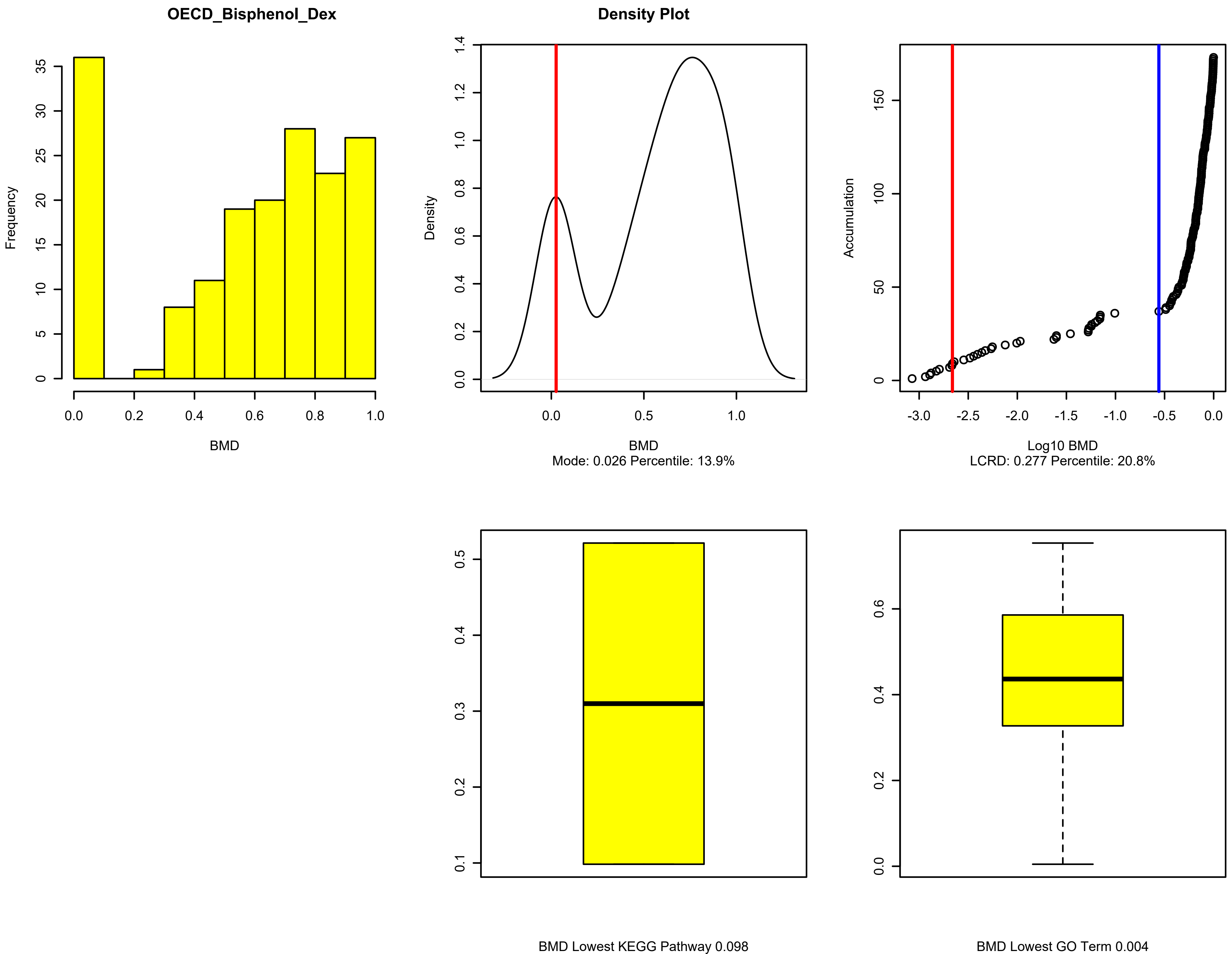
OECD_Bisphenol_Dex
Frequency
BMD
Density Plot
Density
BMD
Mode: 0.026 Percentile: 13.9%
Accumulation
Log10 BMD
LCRD: 0.277 Percentile: 20.8%
BMD Lowest KEGG Pathway 0.098
BMD Lowest GO Term 0.004

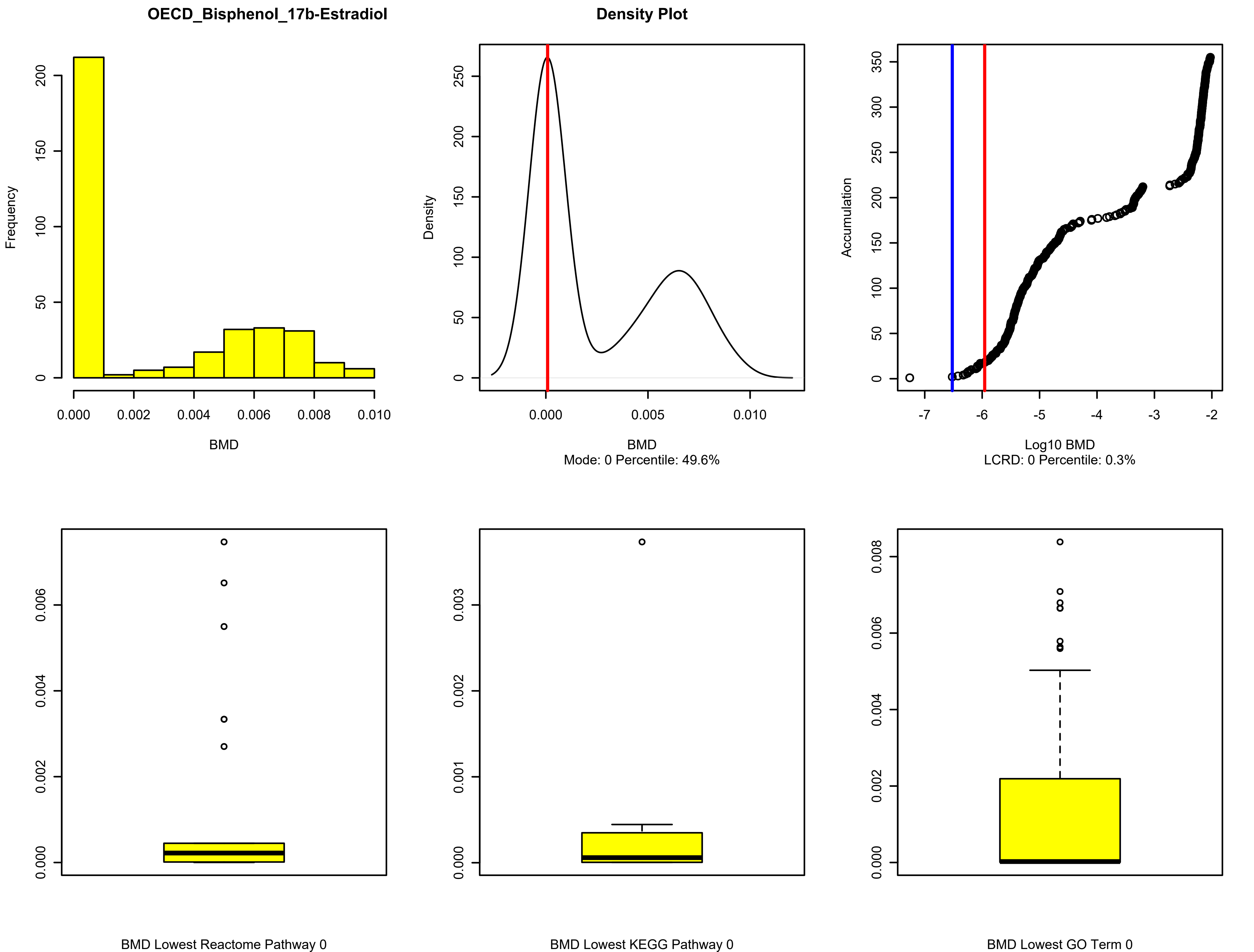

OECD_Bisphenol_17b-Estradiol
Density Plot
Frequency
BMD
Density
BMD
Mode: 0 Percentile: 49.6%
Accumulation
Log10 BMD
LCRD: 0 Percentile: 0.3%
BMD Lowest Reactome Pathway 0
BMD Lowest KEGG Pathway 0
BMD Lowest GO Term 0

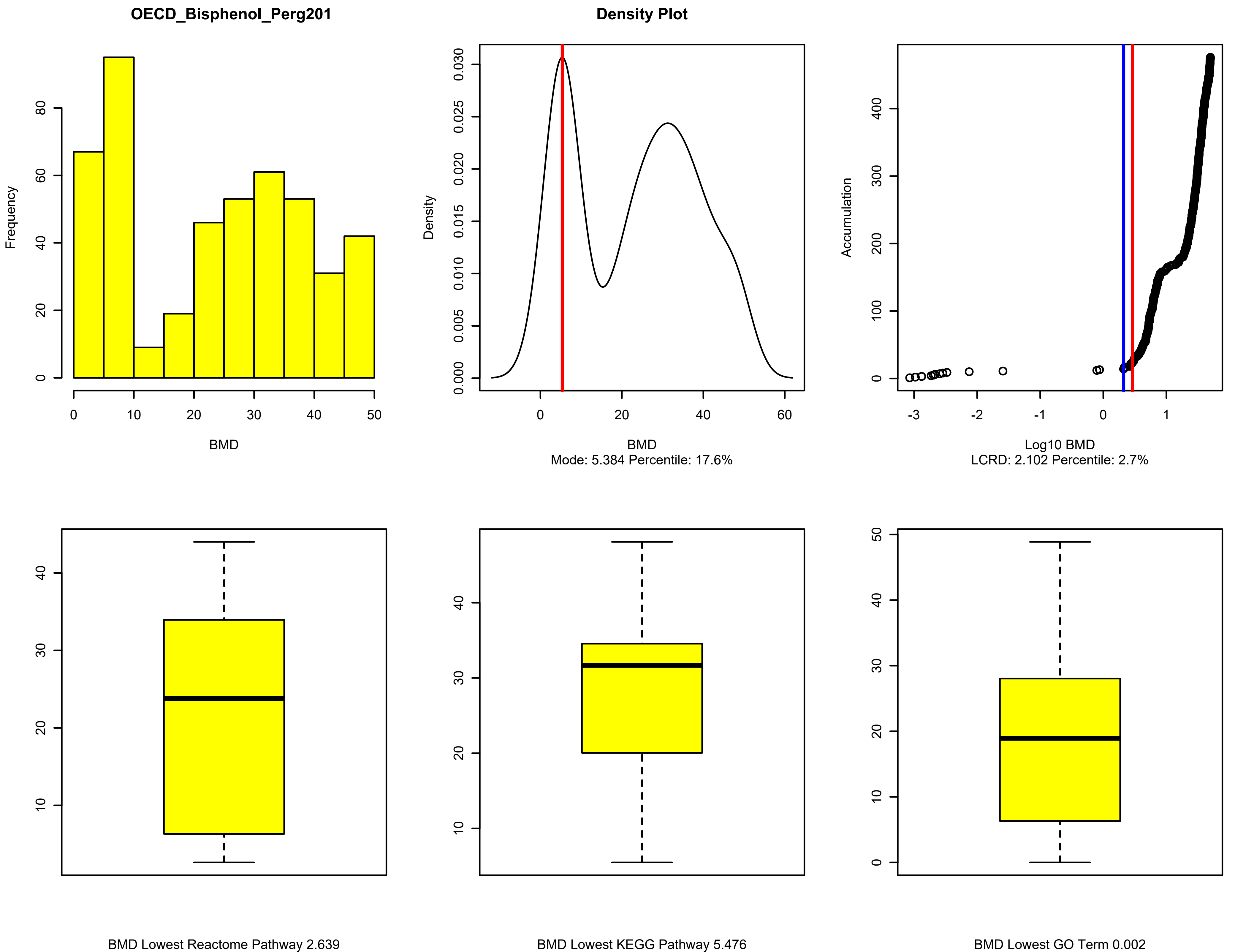

OECD_Bisphenol_Perg201
Frequency
BMD
Density Plot
Density
BMD
Mode: 5.384 Percentile: 17.6%
Accumulation
Log10 BMD
LCRD: 2.102 Percentile: 2.7%
BMD Lowest Reactome Pathway 2.639
BMD Lowest KEGG Pathway 5.476
BMD Lowest GO Term 0.002

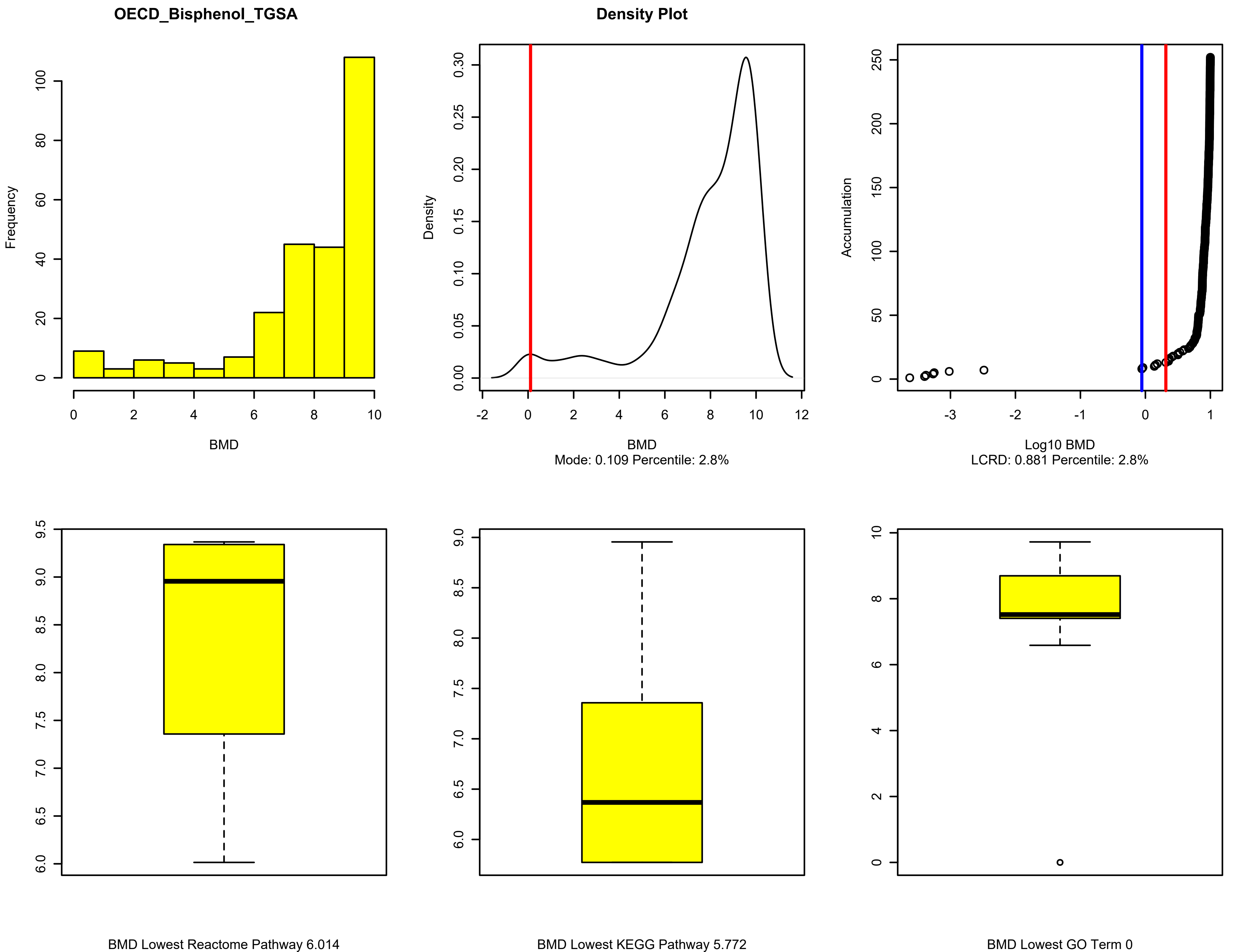
OECD_Bisphenol_TGSA
Frequency
BMD
Density Plot
Density
BMD
Mode: 0.109 Percentile: 2.8%
Accumulation
Log10 BMD
LCRD: 0.881 Percentile: 2.8%
BMD Lowest Reactome Pathway 6.014
BMD Lowest KEGG Pathway 5.772
BMD Lowest GO Term 0

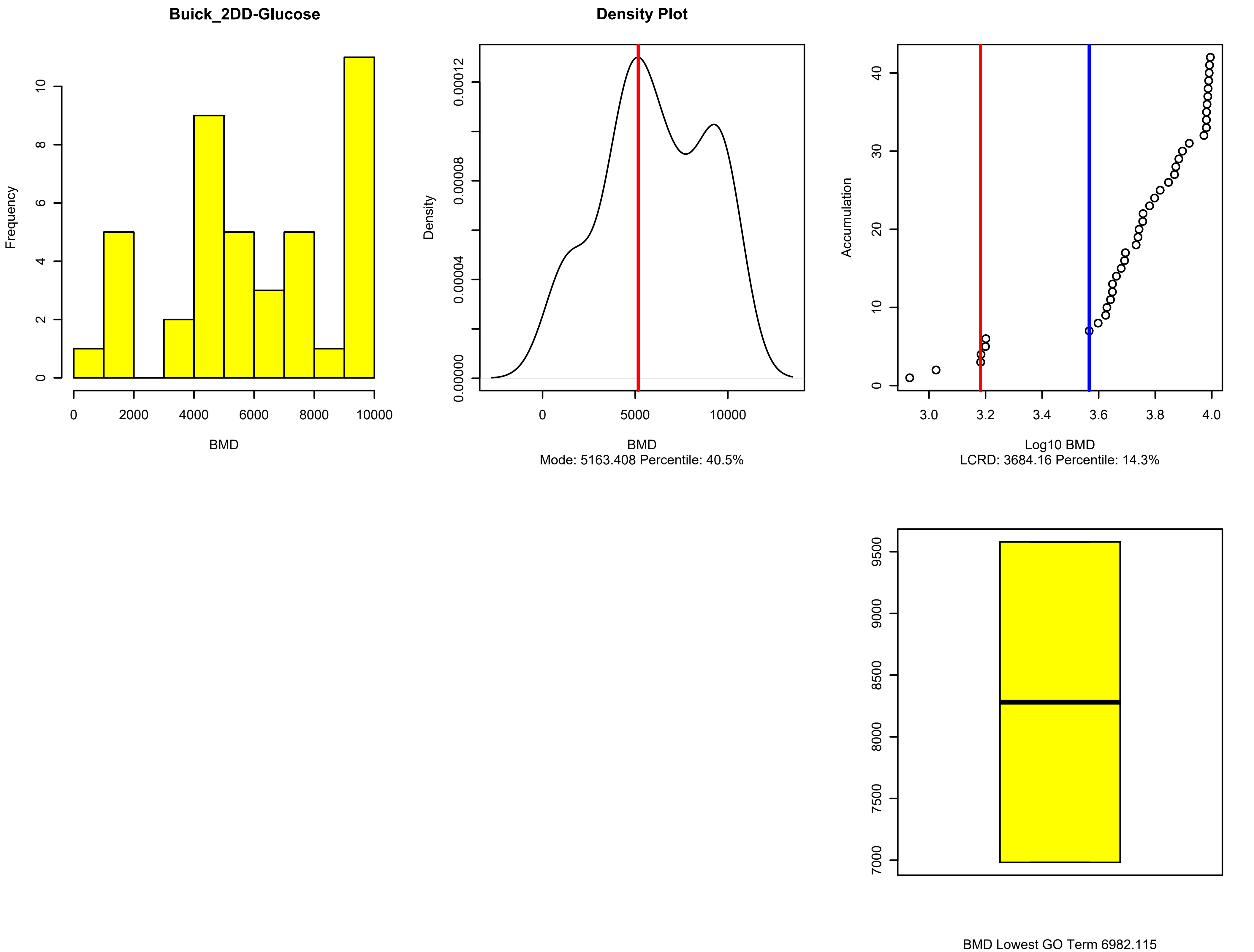

Buick_2DD-Glucose
Frequency
BMD
Density Plot
Density
BMD
Mode: 5163.408 Percentile: 40.5%
Accumulation
Log10 BMD
LCRD: 3684.16 Percentile: 14.3%
BMD Lowest GO Term 6982.115

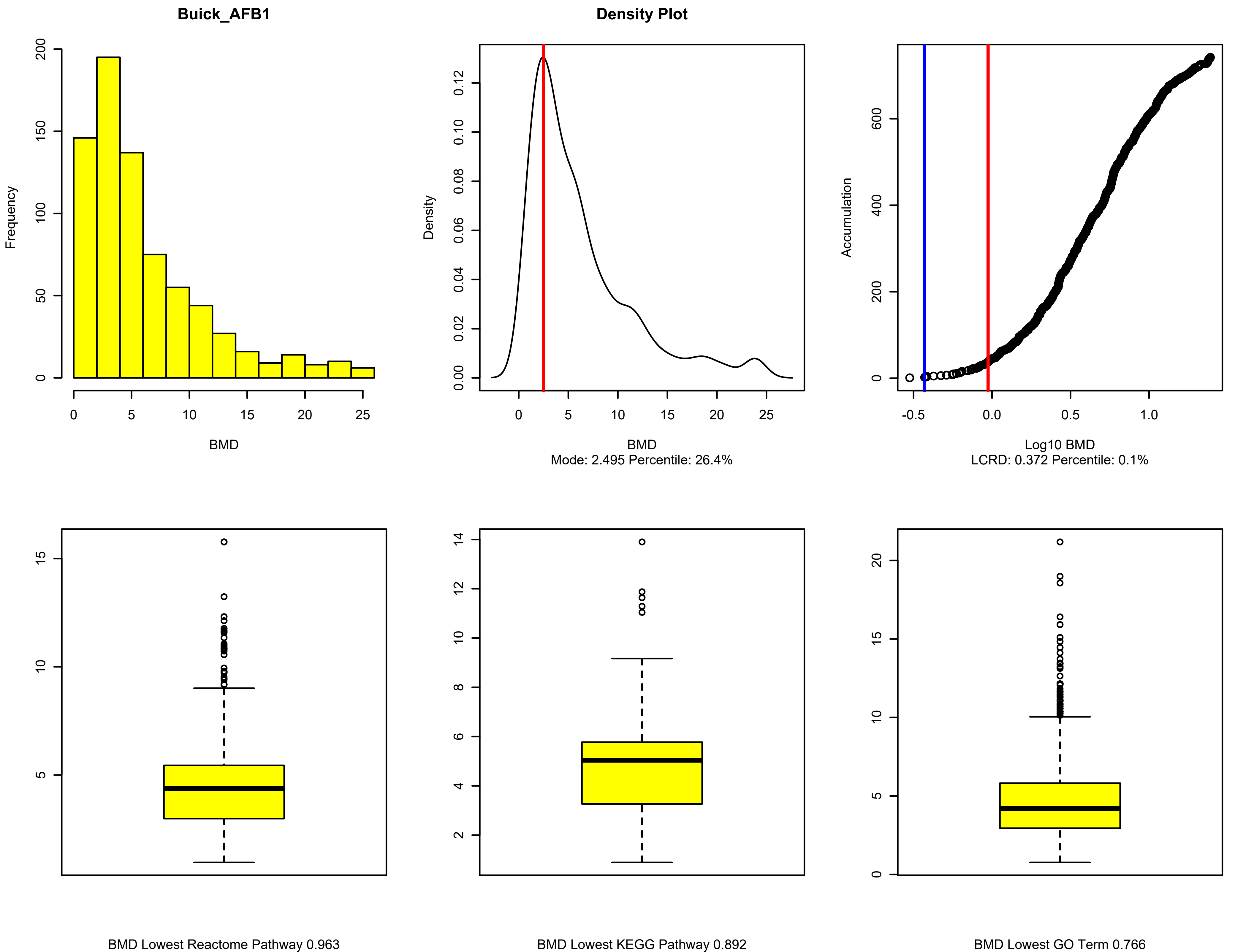
Buick_AFB1
Frequency
BMD
Density Plot
Density
BMD
Mode: 2.495 Percentile: 26.4%
Accumulation
Log10 BMD
LCRD: 0.372 Percentile: 0.1%
BMD Lowest Reactome Pathway 0.963
BMD Lowest KEGG Pathway 0.892
BMD Lowest GO Term 0.766

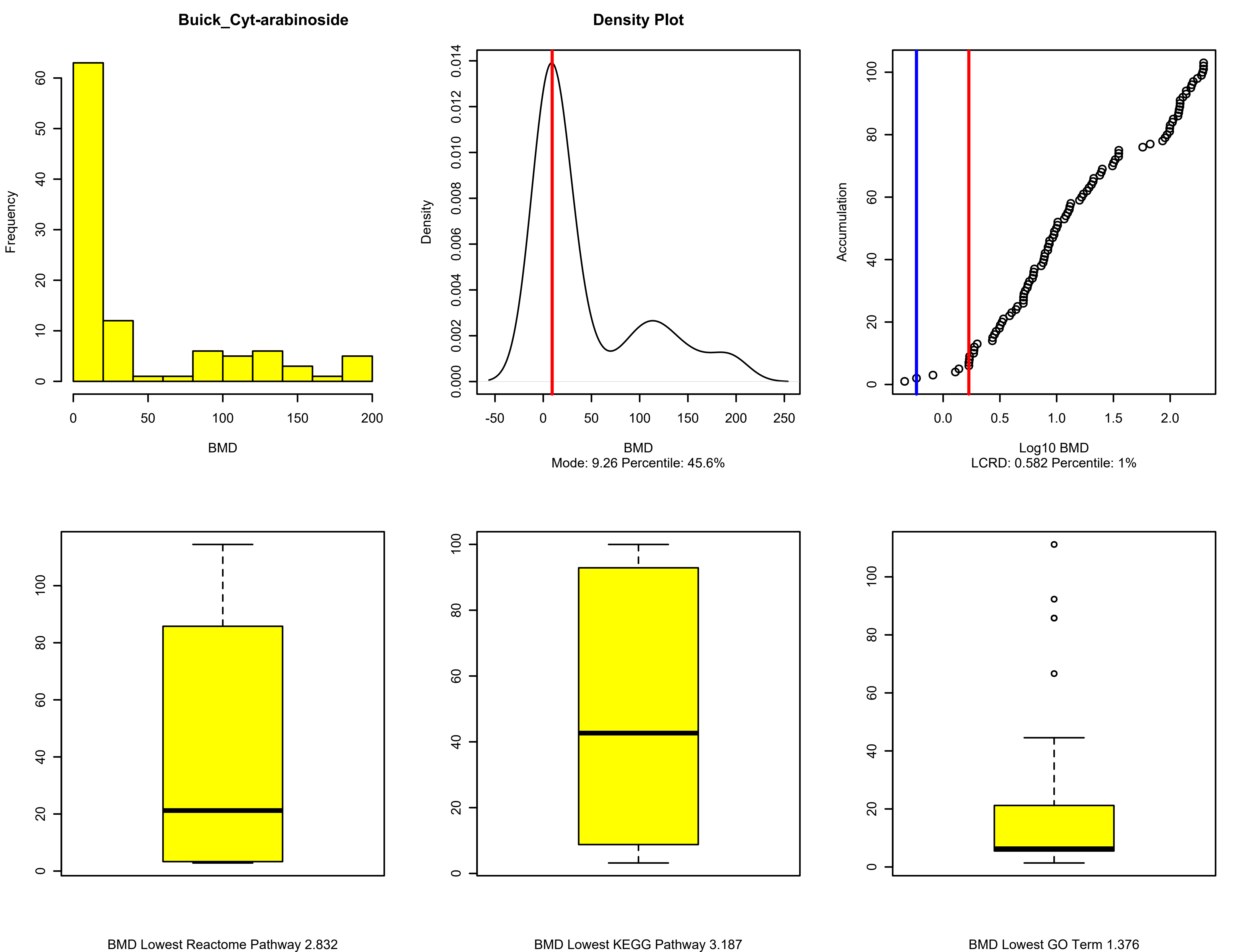

Buick_Cyt-arabinoside
Frequency
BMD
Density Plot
Density
BMD
Mode: 9.26 Percentile: 45.6%
Accumulation
Log10 BMD
LCRD: 0.582 Percentile: 1%
BMD Lowest Reactome Pathway 2.832
BMD Lowest KEGG Pathway 3.187
BMD Lowest GO Term 1.376

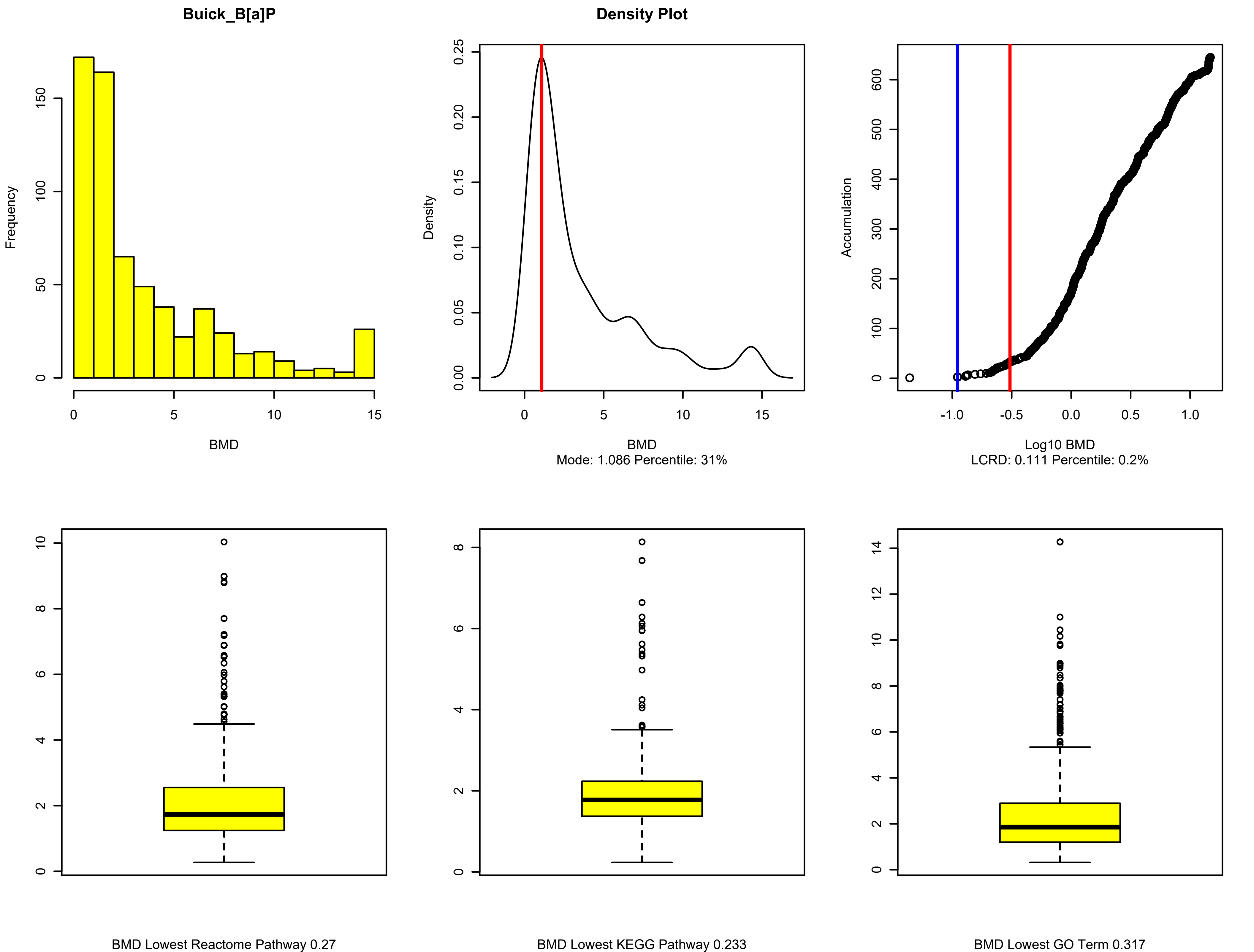
Buick_B[a]P
Frequency
BMD
Density Plot
Density
BMD
Mode: 1.086 Percentile: 31%
Accumulation
Log10 BMD
LCRD: 0.111 Percentile: 0.2%
BMD Lowest Reactome Pathway 0.27
BMD Lowest KEGG Pathway 0.233
BMD Lowest GO Term 0.317

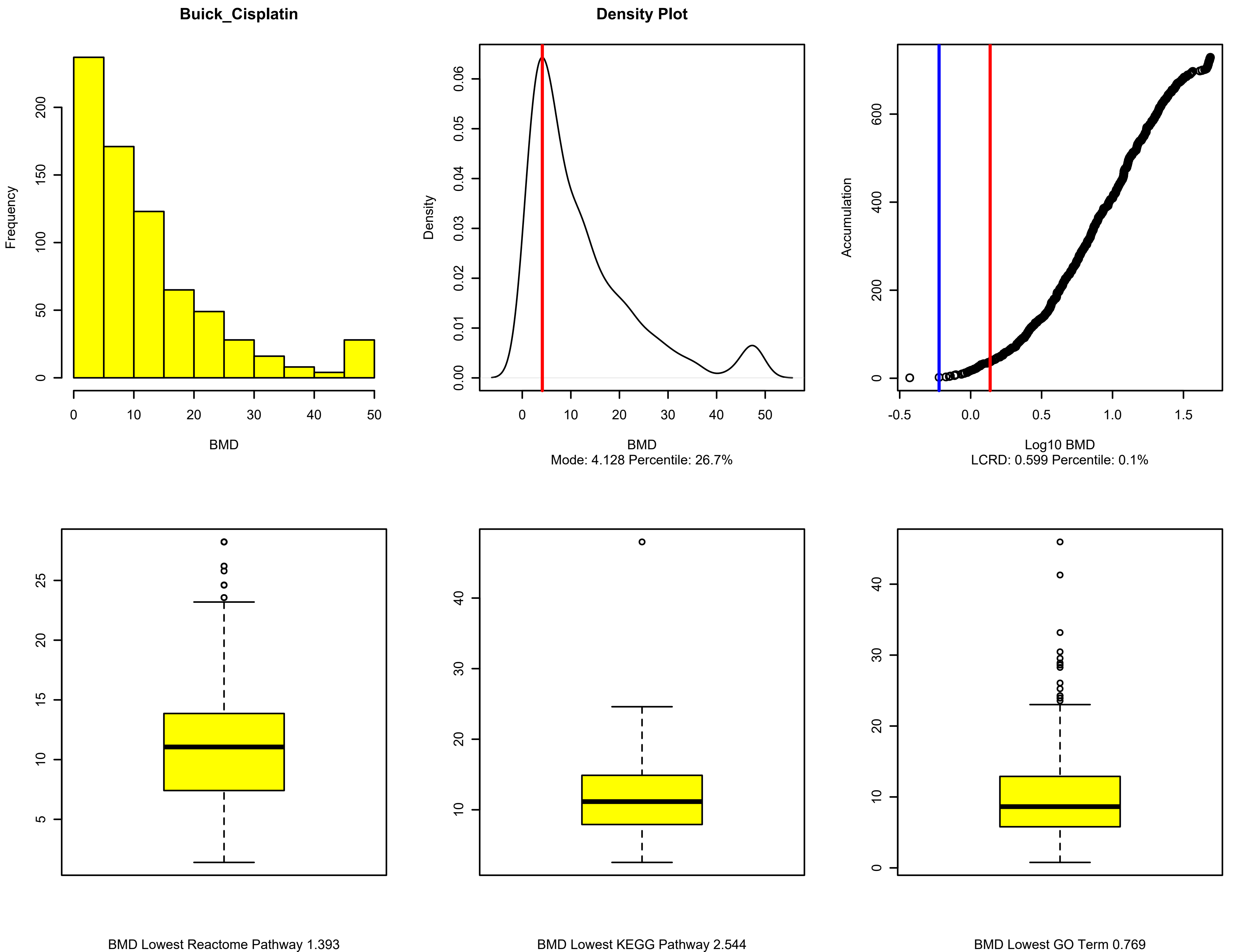

Buick_Cisplatin
Frequency
BMD
Density Plot
Density
BMD
Mode: 4.128 Percentile: 26.7%
Accumulation
Log10 BMD
LCRD: 0.599 Percentile: 0.1%
BMD Lowest Reactome Pathway 1.393
BMD Lowest KEGG Pathway 2.544
BMD Lowest GO Term 0.769

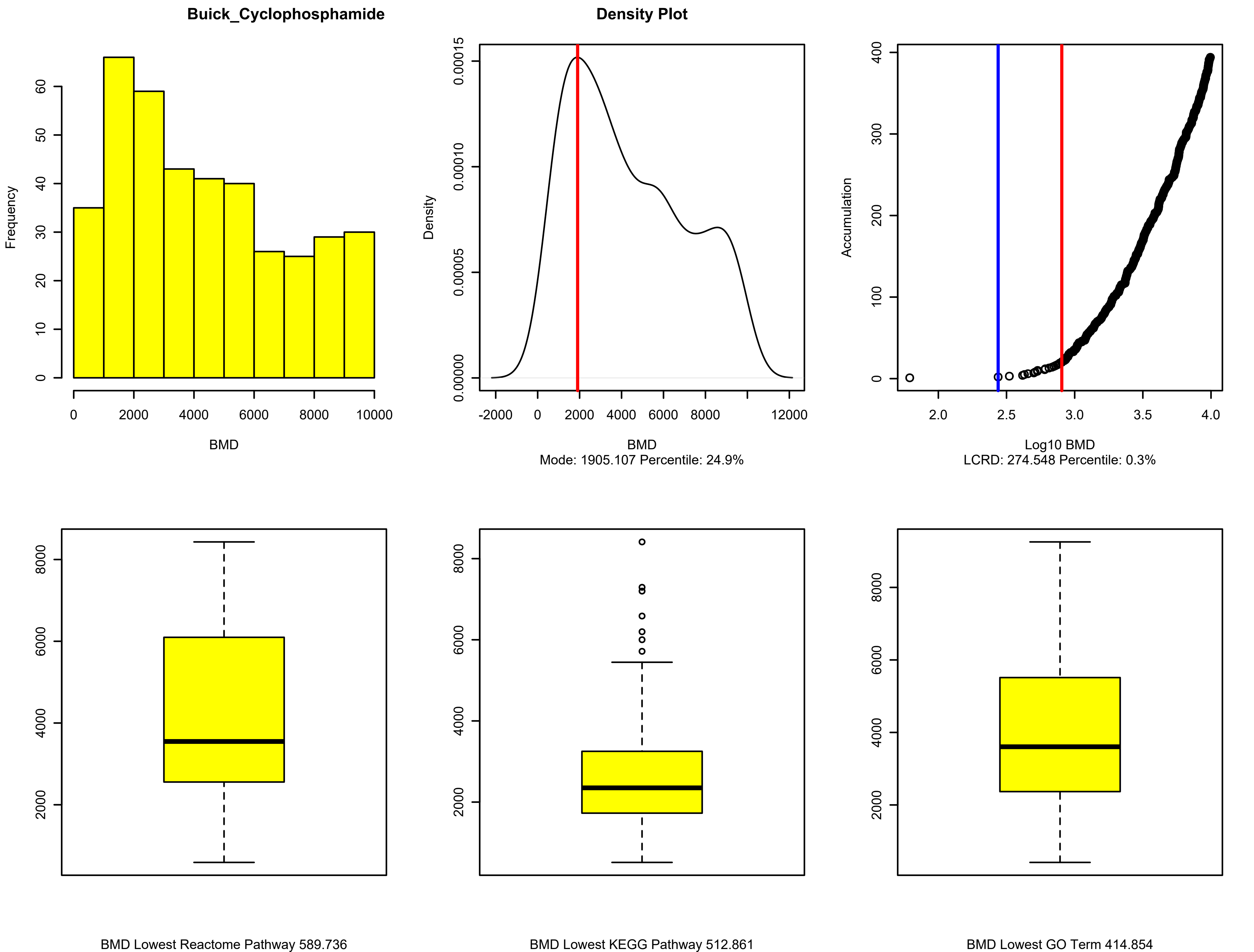

Buick_Cyclophosphamide
Frequency
0
10
20
30
40
50
60
0
2000
4000
6000
8000
10000
BMD
Density Plot
Density
0.00000
0.00005
0.00010
0.00015
-2000
0
2000
4000
6000
8000
12000
BMD
Mode: 1905.107 Percentile: 24.9%
Accumulation
0
100
200
300
400
2.0
2.5
3.0
3.5
4.0
Log10 BMD
LCRD: 274.548 Percentile: 0.3%
2000
4000
6000
8000
BMD Lowest Reactome Pathway 589.736
2000
4000
6000
8000
BMD Lowest KEGG Pathway 512.861
2000
4000
6000
8000
BMD Lowest GO Term 414.854

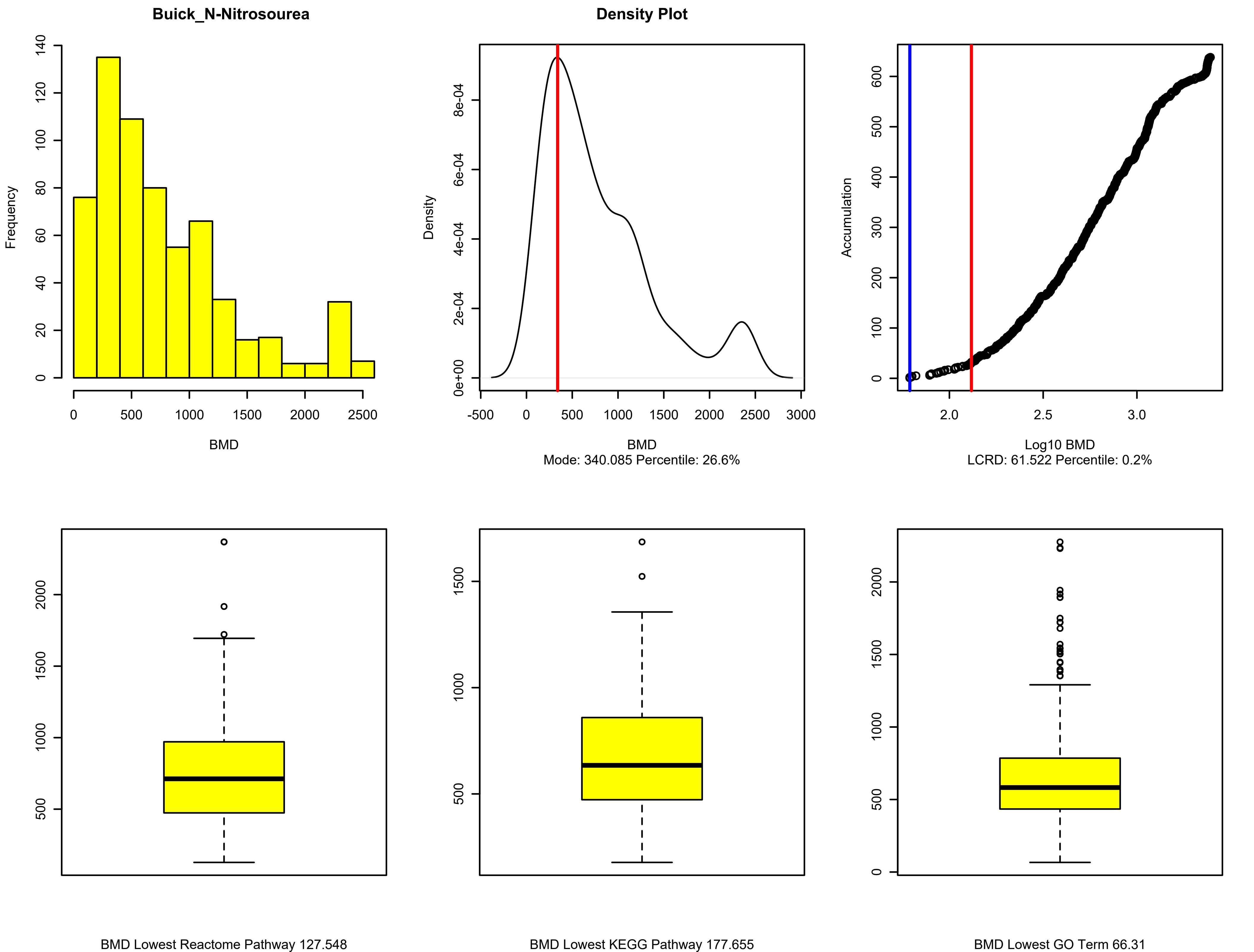
Buick_N-Nitrosourea
Frequency
BMD
Density Plot
Density
BMD
Mode: 340.085 Percentile: 26.6%
Accumulation
Log10 BMD
LCRD: 61.522 Percentile: 0.2%
BMD Lowest Reactome Pathway 127.548
BMD Lowest KEGG Pathway 177.655
BMD Lowest GO Term 66.31

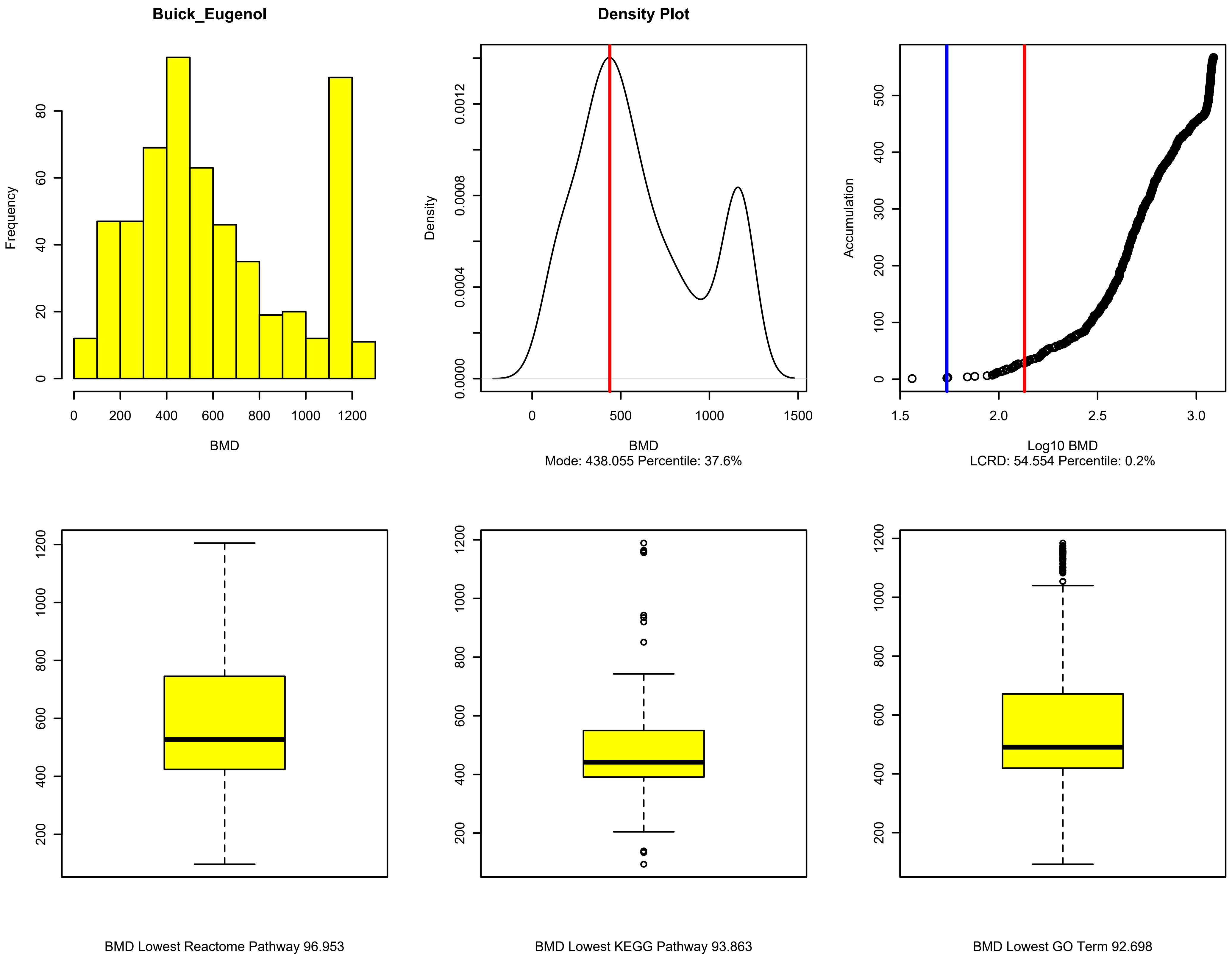

Buick_Eugenol
Frequency
BMD
Density Plot
Density
BMD
Mode: 438.055 Percentile: 37.6%
Accumulation
Log10 BMD
LCRD: 54.554 Percentile: 0.2%
BMD Lowest Reactome Pathway 96.953
BMD Lowest KEGG Pathway 93.863
BMD Lowest GO Term 92.698

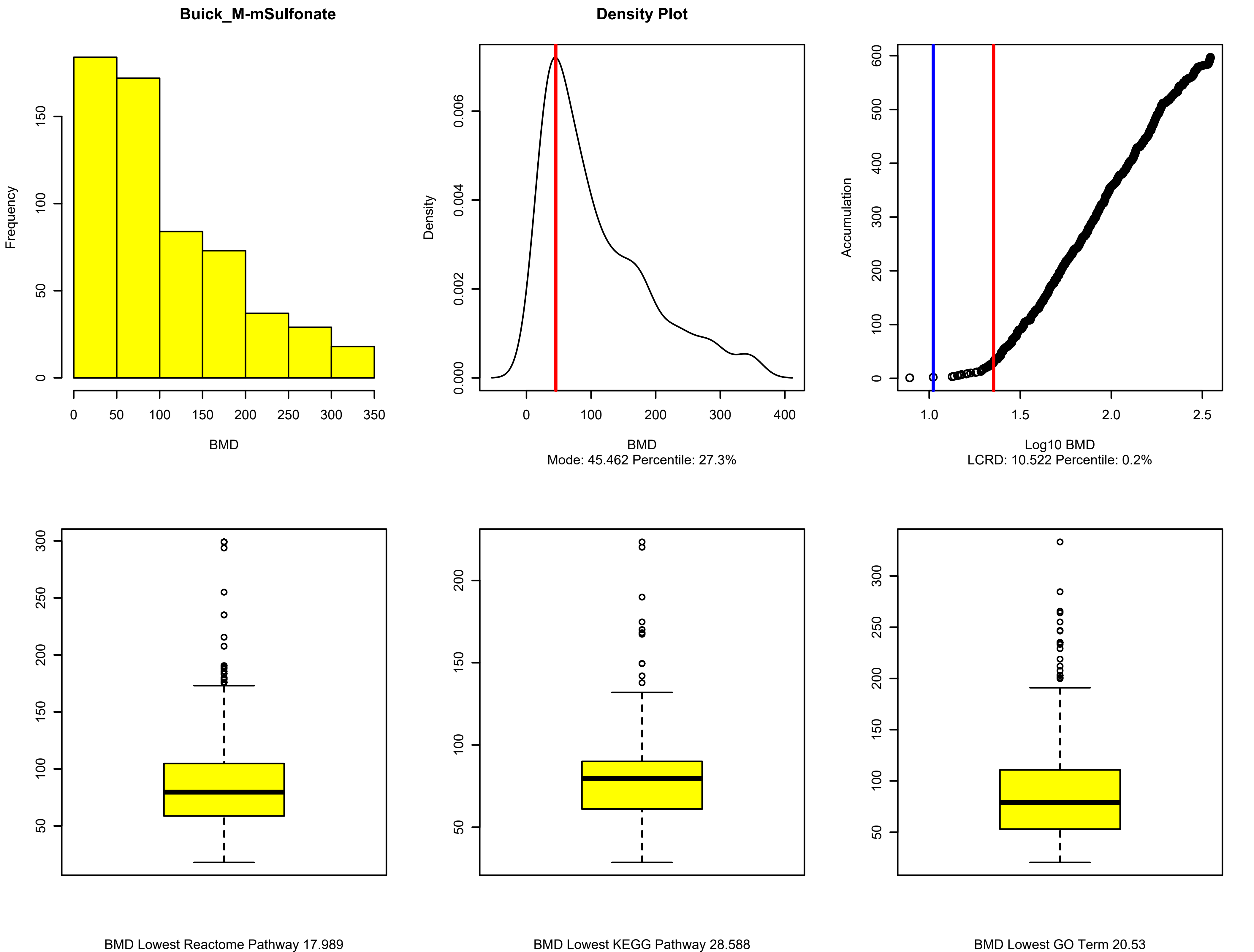

Buick_M-mSulfonate
Frequency
BMD
Density Plot
Density
BMD
Mode: 45.462 Percentile: 27.3%
Accumulation
Log10 BMD
LCRD: 10.522 Percentile: 0.2%
BMD Lowest Reactome Pathway 17.989
BMD Lowest KEGG Pathway 28.588
BMD Lowest GO Term 20.53

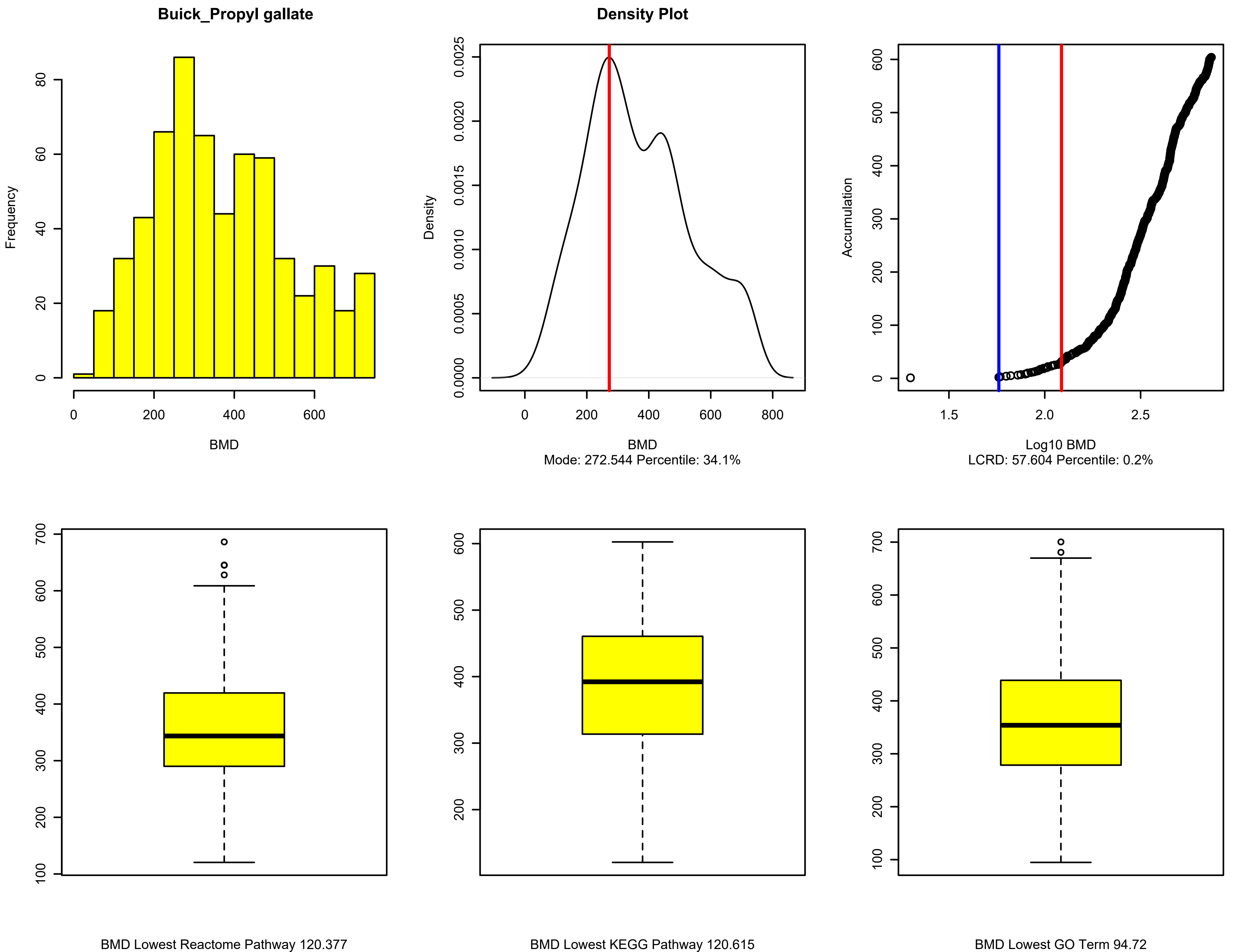
Buick_Propyl gallate
Frequency
BMD
0
20
40
60
80
0
200
400
600
Density Plot
Density
BMD
Mode: 272.544 Percentile: 34.1%
0.0000
0.0005
0.0010
0.0015
0.0020
0.0025
0
200
400
600
800
Accumulation
Log10 BMD
LCRD: 57.604 Percentile: 0.2%
0
100
200
300
400
500
600
1.5
2.0
2.5
100
200
300
400
500
600
700
BMD Lowest Reactome Pathway 120.377
200
300
400
500
600
BMD Lowest KEGG Pathway 120.615
100
200
300
400
500
600
700
BMD Lowest GO Term 94.72

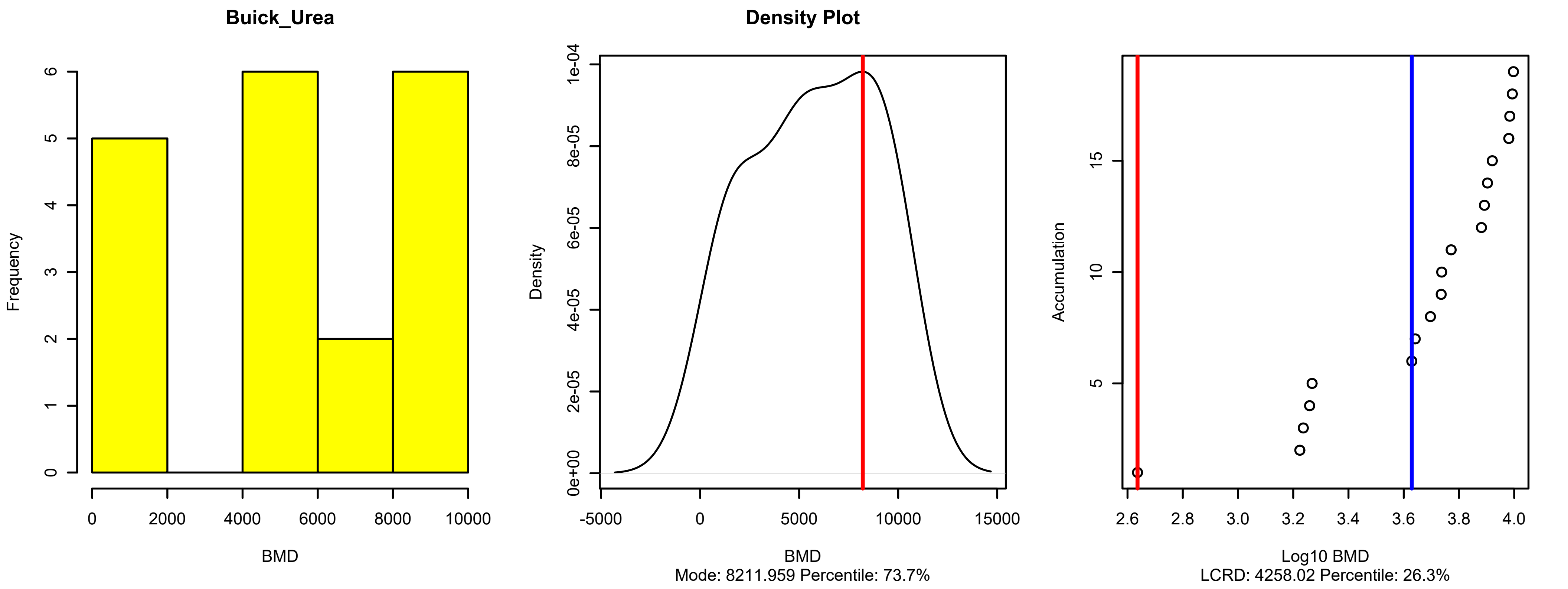

Buick_Urea
Frequency
BMD
Density Plot
Density
BMD
Mode: 8211.959 Percentile: 73.7%
Accumulation
Log10 BMD
LCRD: 4258.02 Percentile: 26.3%

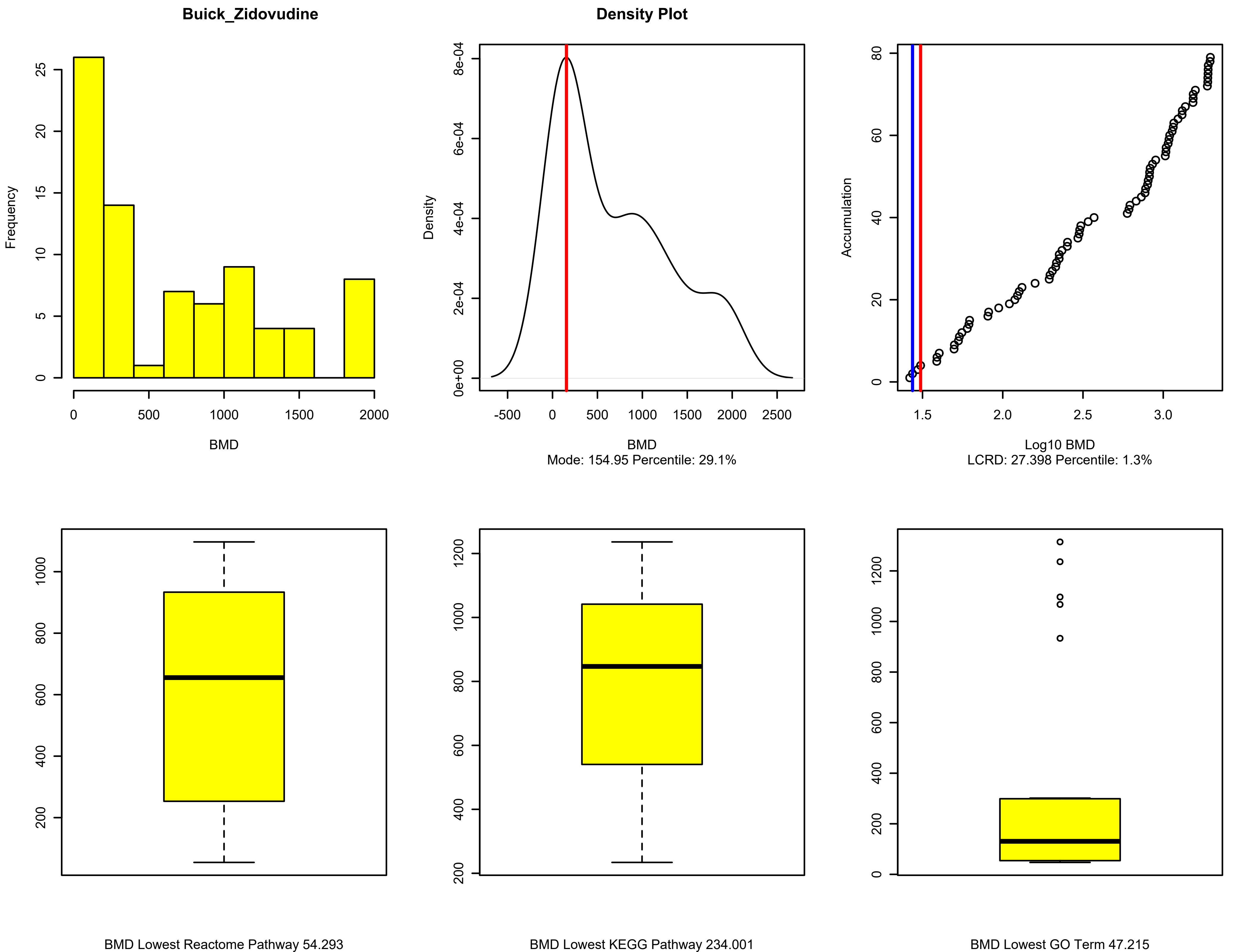
Buick_Zidovudine
Frequency
BMD
Density Plot
Density
BMD
Mode: 154.95 Percentile: 29.1%
Accumulation
Log10 BMD
LCRD: 27.398 Percentile: 1.3%
BMD Lowest Reactome Pathway 54.293
BMD Lowest KEGG Pathway 234.001
BMD Lowest GO Term 47.215

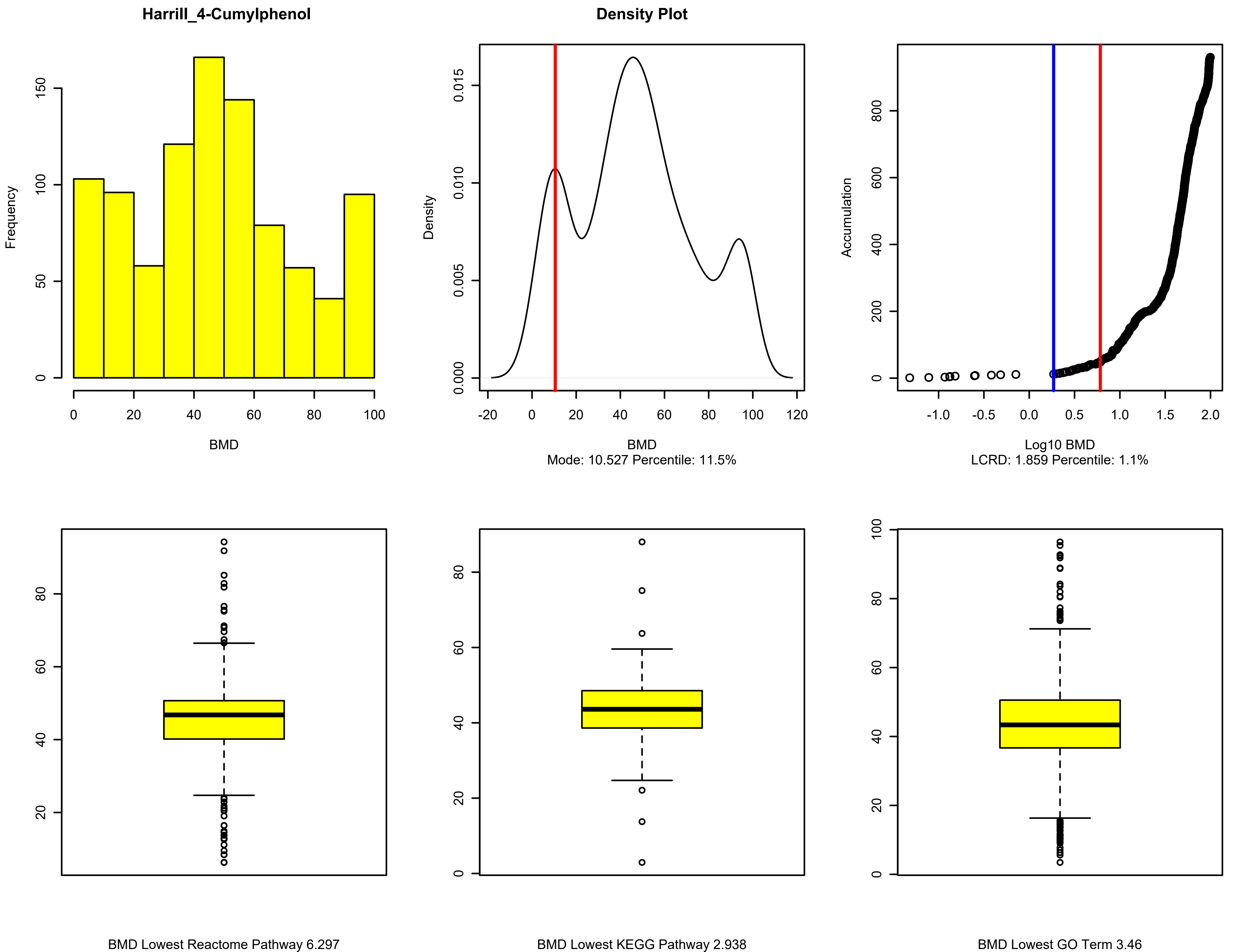
Harrill_4-Cumylphenol
Frequency
BMD
Density Plot
Density
BMD
Mode: 10.527 Percentile: 11.5%
Accumulation
Log10 BMD
LCRD: 1.859 Percentile: 1.1%
BMD Lowest Reactome Pathway 6.297
BMD Lowest KEGG Pathway 2.938
BMD Lowest GO Term 3.46

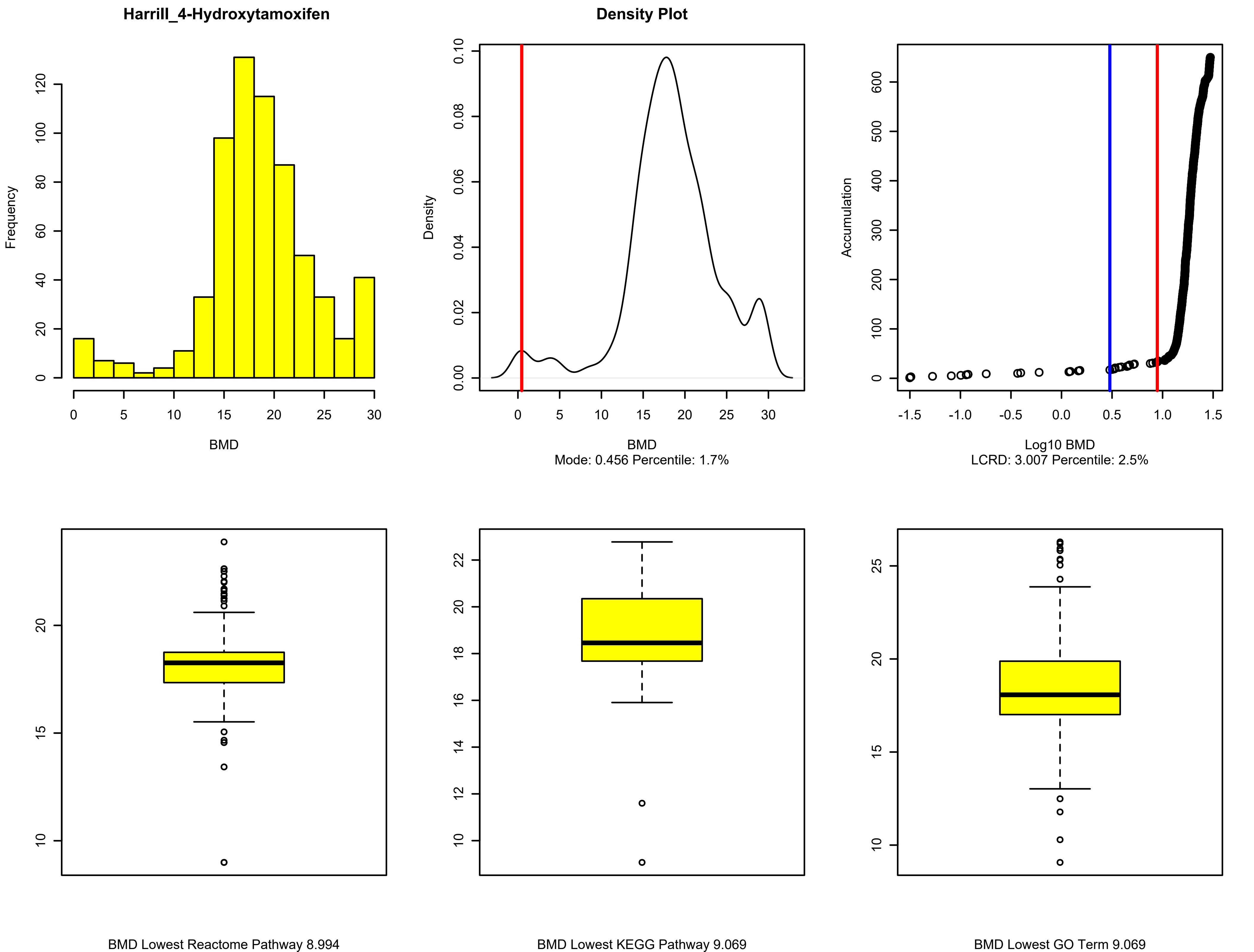
Harrill_4-Hydroxytamoxifen
Frequency
BMD
Density Plot
Density
BMD
Mode: 0.456 Percentile: 1.7%
Accumulation
Log10 BMD
LCRD: 3.007 Percentile: 2.5%
BMD Lowest Reactome Pathway 8.994
BMD Lowest KEGG Pathway 9.069
BMD Lowest GO Term 9.069

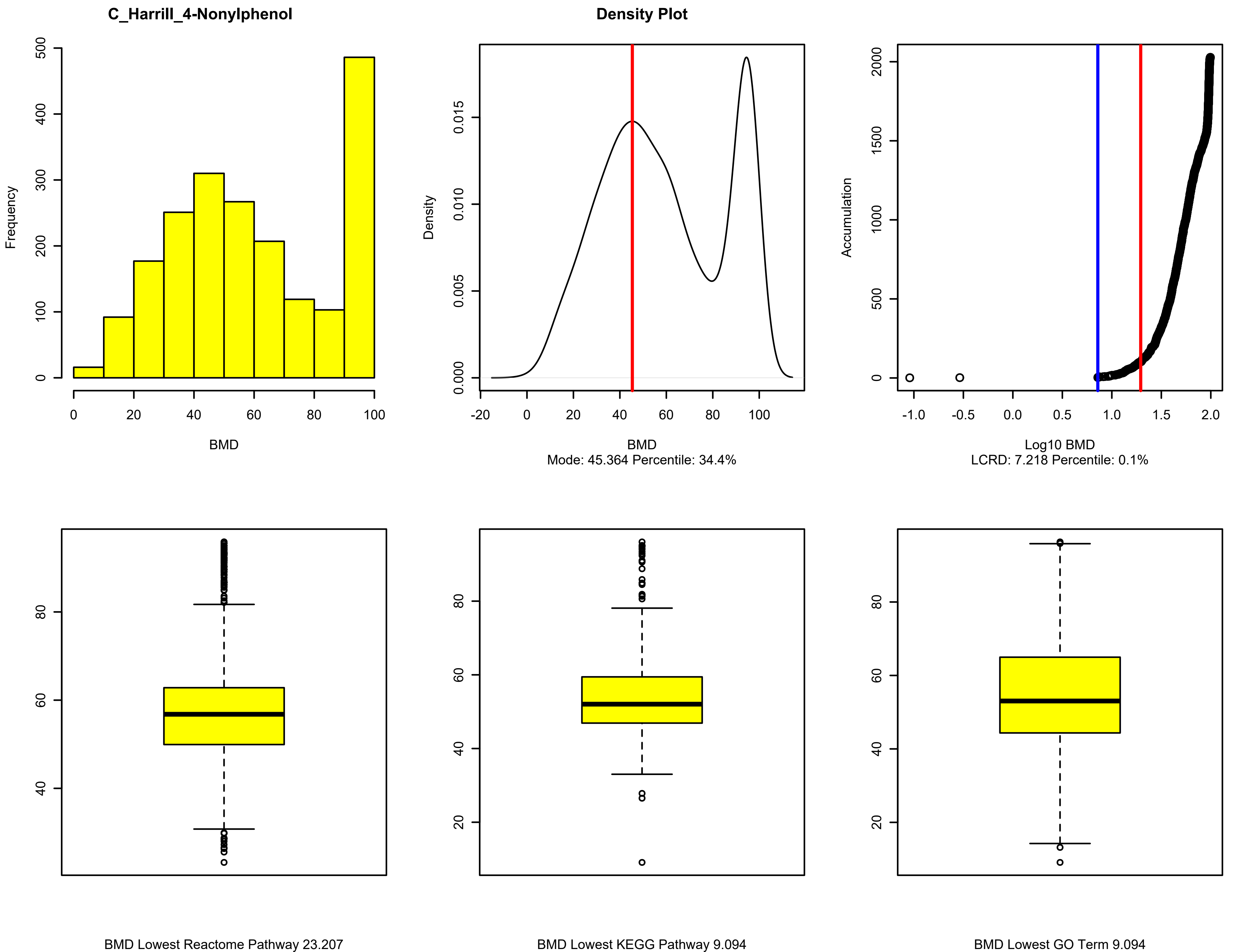
C_Harrill_4-Nonylphenol
Frequency
BMD
Density Plot
Density
BMD
Mode: 45.364 Percentile: 34.4%
Accumulation
Log10 BMD
LCRD: 7.218 Percentile: 0.1%
BMD Lowest Reactome Pathway 23.207
BMD Lowest KEGG Pathway 9.094
BMD Lowest GO Term 9.094

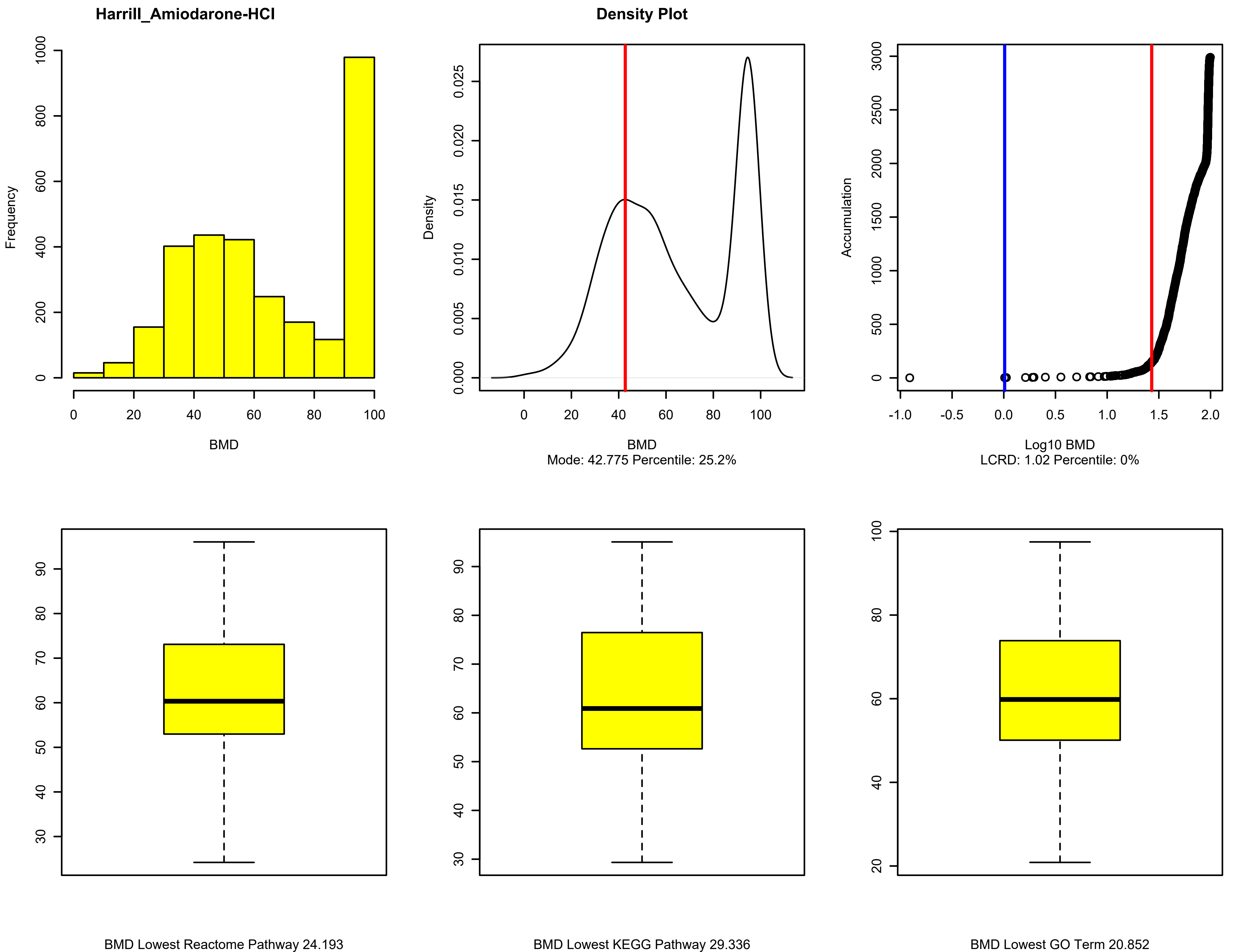
Harrill_Amiodarone-HCl
Frequency
BMD
Density Plot
Density
BMD
Mode: 42.775 Percentile: 25.2%
Accumulation
Log10 BMD
LCRD: 1.02 Percentile: 0%
BMD Lowest Reactome Pathway 24.193
BMD Lowest KEGG Pathway 29.336
BMD Lowest GO Term 20.852

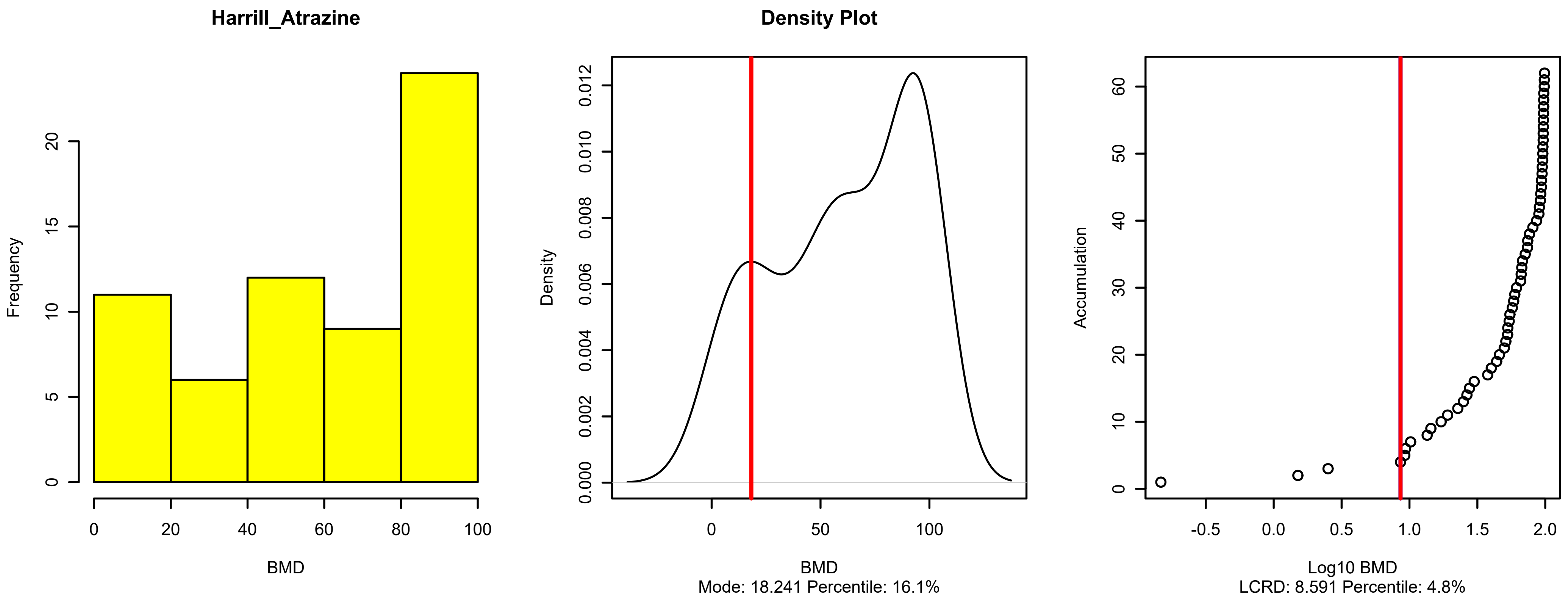

Harrill_Atrazine
Frequency
BMD
Density Plot
Density
BMD
Mode: 18.241 Percentile: 16.1%
Accumulation
Log10 BMD
LCRD: 8.591 Percentile: 4.8%

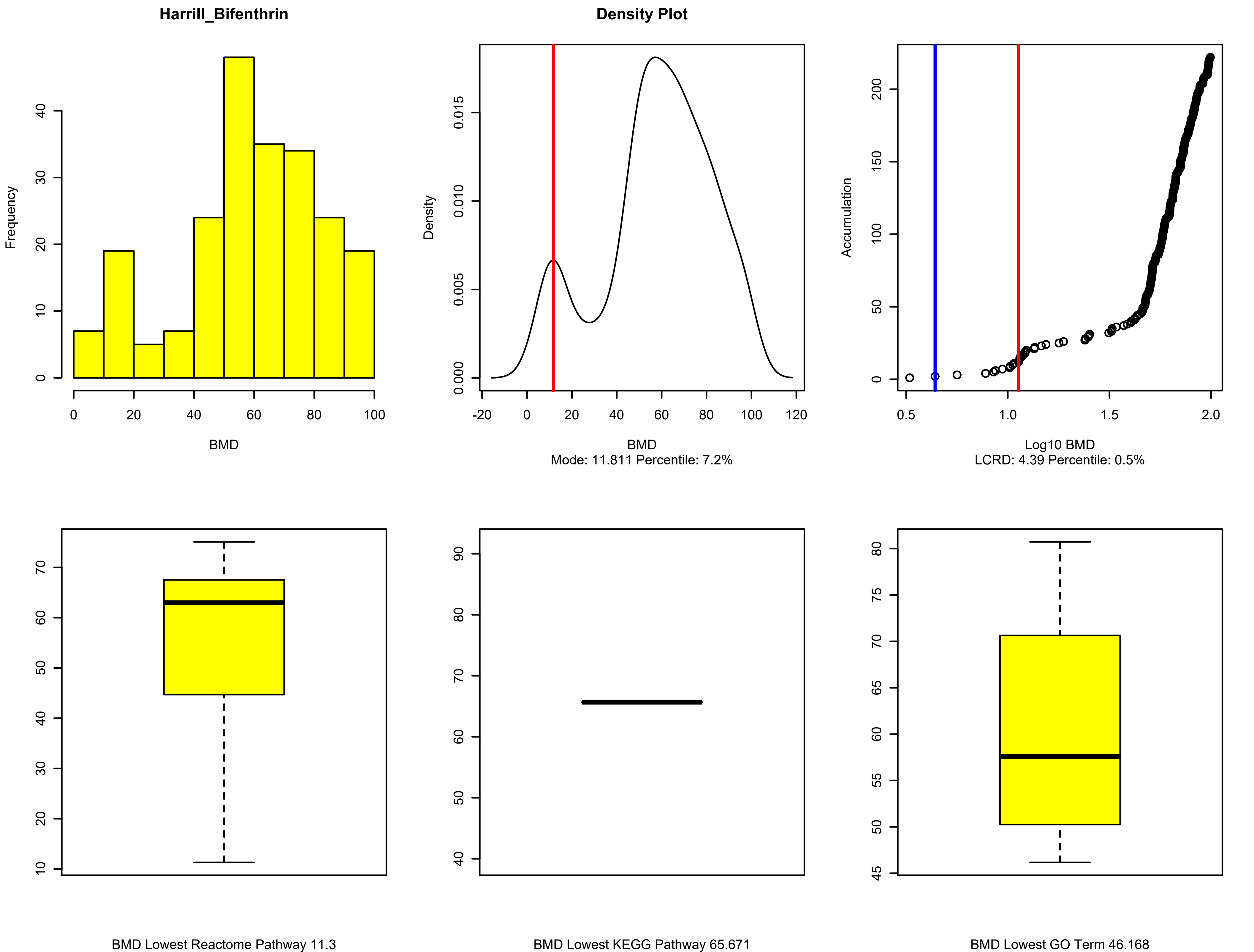
Harrill_Bifenthrin
Frequency
BMD
Density Plot
Density
BMD
Mode: 11.811 Percentile: 7.2%
Accumulation
Log10 BMD
LCRD: 4.39 Percentile: 0.5%
BMD Lowest Reactome Pathway 11.3
BMD Lowest KEGG Pathway 65.671
BMD Lowest GO Term 46.168

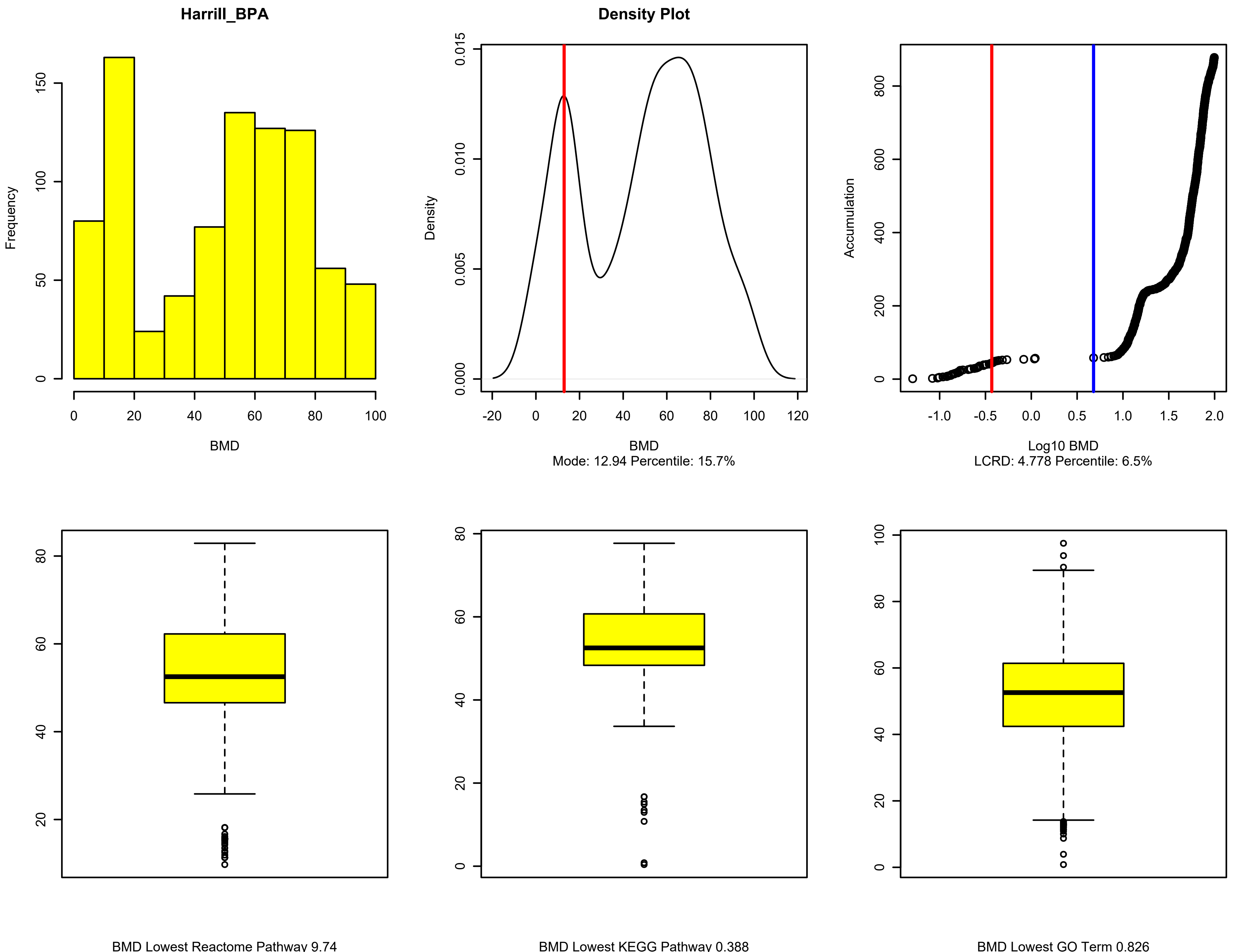

Harrill_BPA
Frequency
BMD
Density Plot
Density
BMD
Mode: 12.94 Percentile: 15.7%
Accumulation
Log10 BMD
LCRD: 4.778 Percentile: 6.5%
BMD Lowest Reactome Pathway 9.74
BMD Lowest KEGG Pathway 0.388
BMD Lowest GO Term 0.826

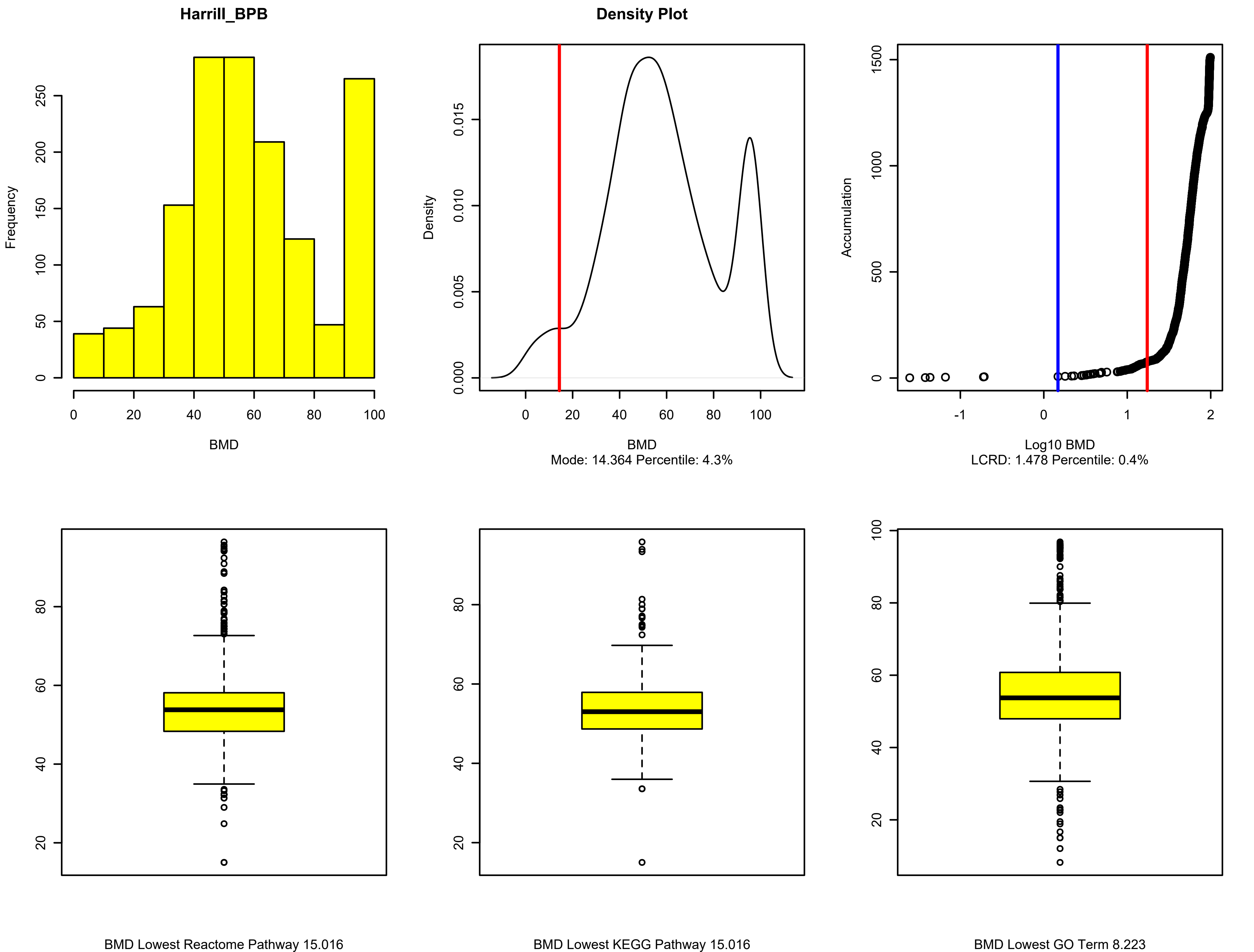
Harrill_BPB
Frequency
BMD
Density Plot
Density
BMD
Mode: 14.364 Percentile: 4.3%
Accumulation
Log10 BMD
LCRD: 1.478 Percentile: 0.4%
BMD Lowest Reactome Pathway 15.016
BMD Lowest KEGG Pathway 15.016
BMD Lowest GO Term 8.223

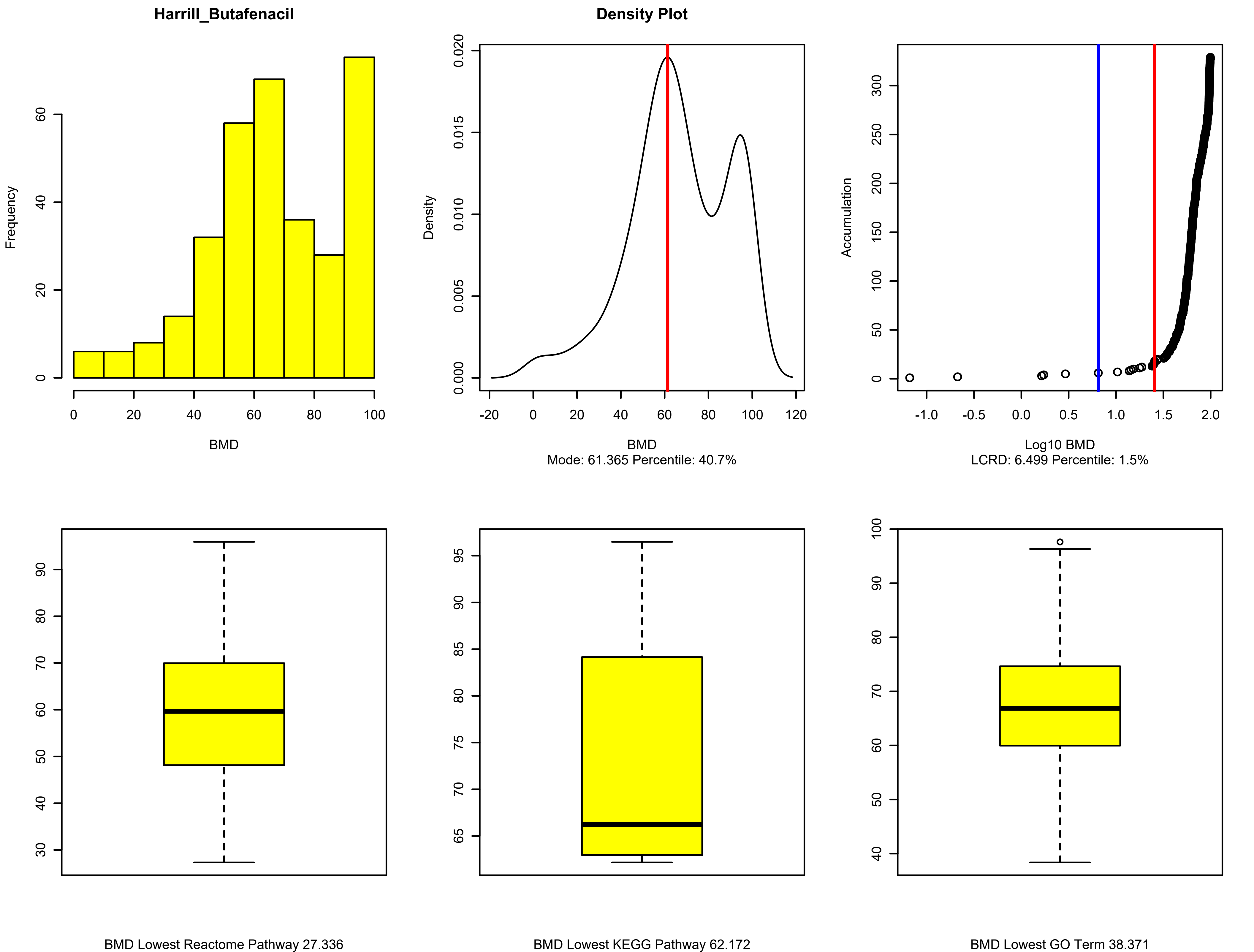
Harrill_Butafenacil
Frequency
BMD
Density Plot
Density
BMD
Mode: 61.365 Percentile: 40.7%
Accumulation
Log10 BMD
LCRD: 6.499 Percentile: 1.5%
BMD Lowest Reactome Pathway 27.336
BMD Lowest KEGG Pathway 62.172
BMD Lowest GO Term 38.371

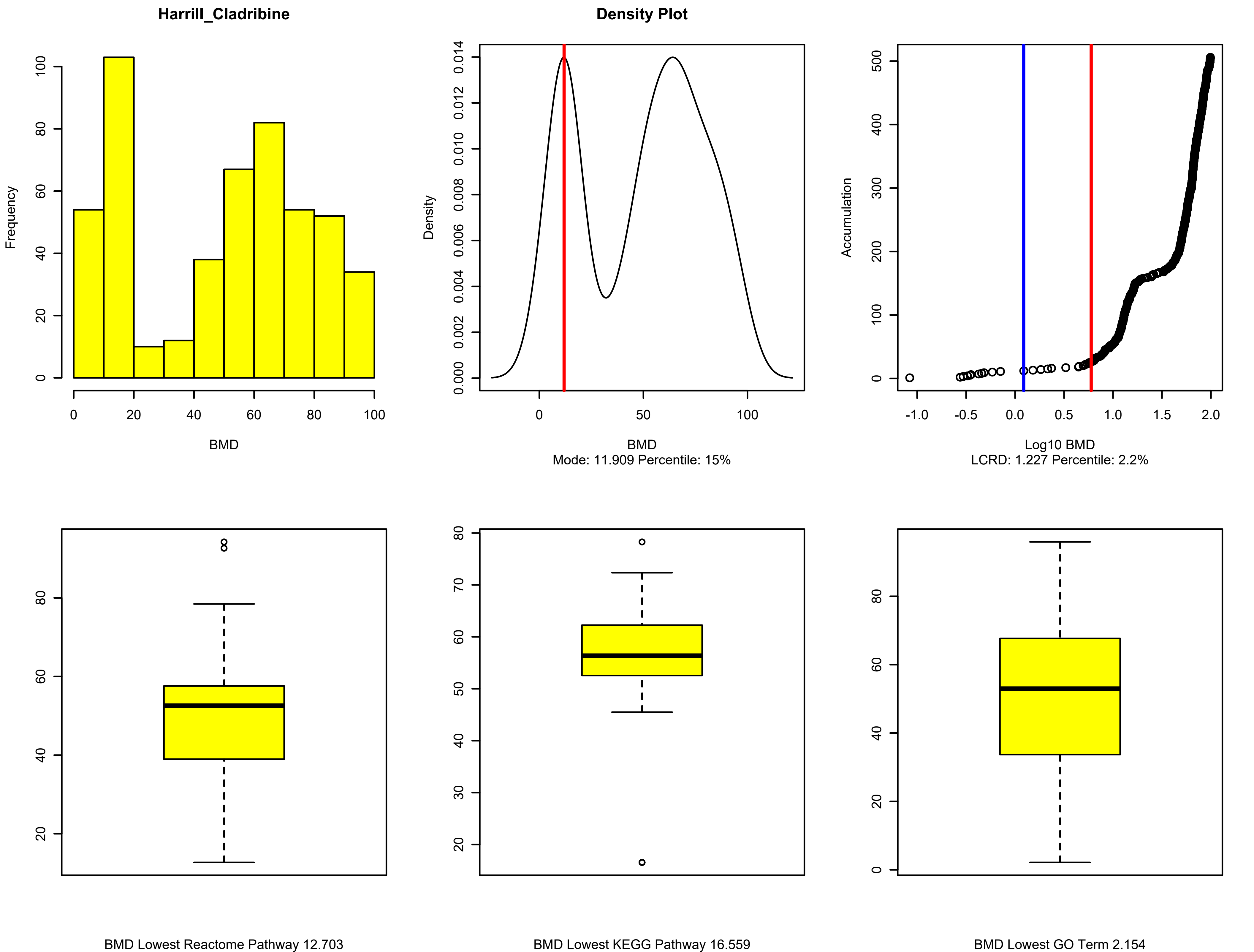

Harrill_Cladribine
Frequency
BMD
Density Plot
Density
BMD
Mode: 11.909 Percentile: 15%
Accumulation
Log10 BMD
LCRD: 1.227 Percentile: 2.2%
BMD Lowest Reactome Pathway 12.703
BMD Lowest KEGG Pathway 16.559
BMD Lowest GO Term 2.154

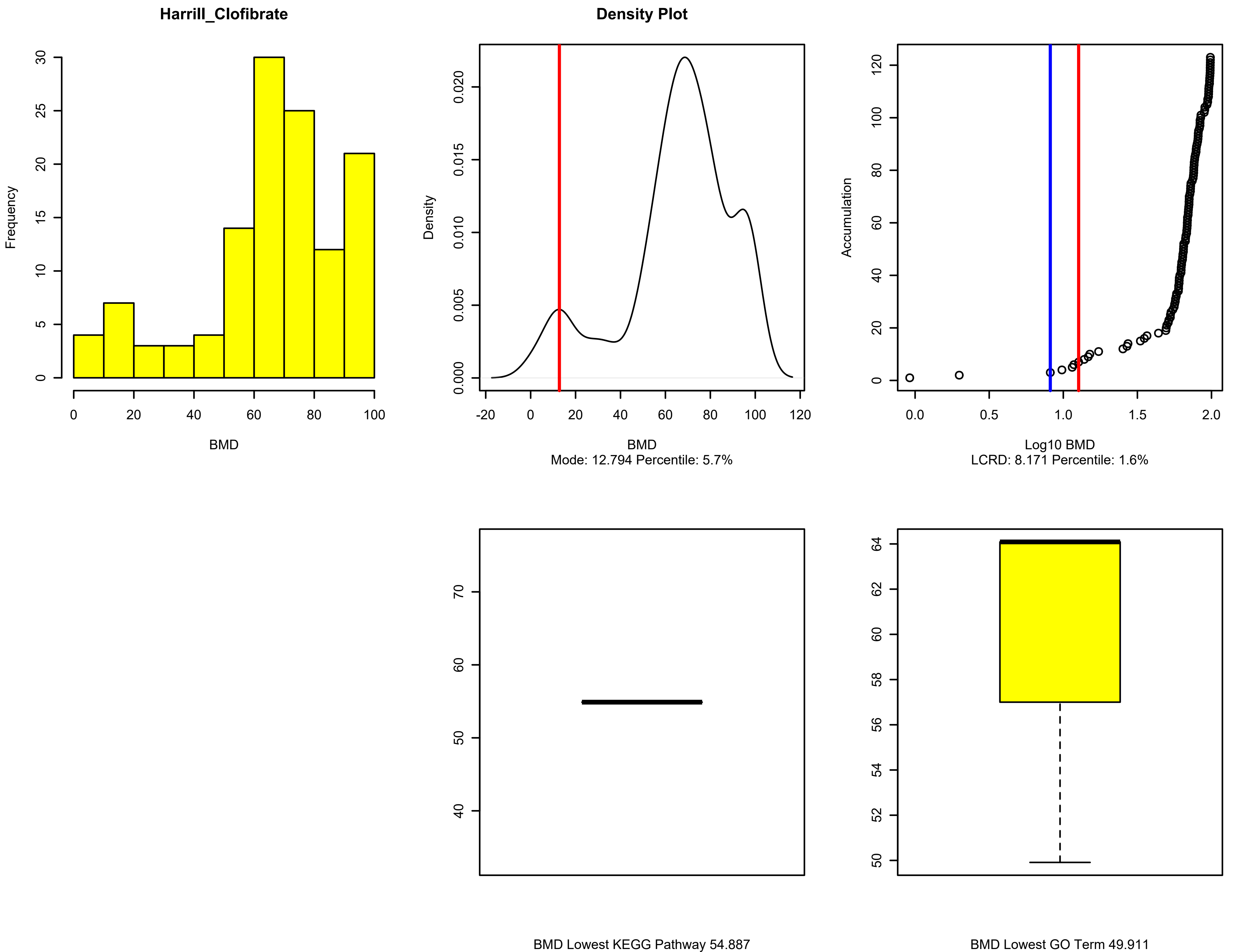

Harrill_Clofibrate
Frequency
BMD
Density Plot
Density
BMD
Mode: 12.794 Percentile: 5.7%
Accumulation
Log10 BMD
LCRD: 8.171 Percentile: 1.6%
BMD Lowest KEGG Pathway 54.887
BMD Lowest GO Term 49.911

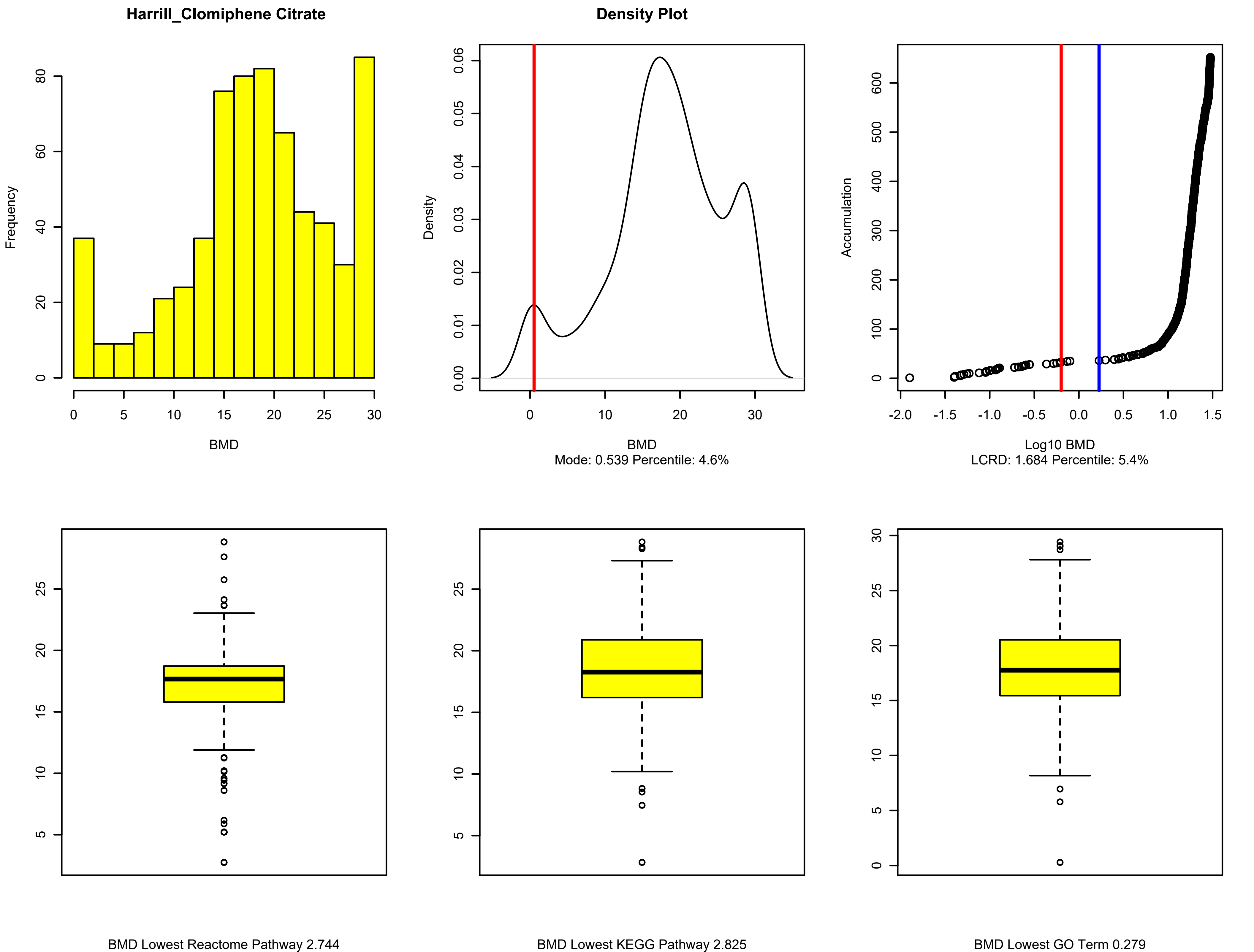

Harrill_Clomiphene Citrate
Frequency
BMD
Density Plot
Density
BMD
Mode: 0.539 Percentile: 4.6%
Accumulation
Log10 BMD
LCRD: 1.684 Percentile: 5.4%
BMD Lowest Reactome Pathway 2.744
BMD Lowest KEGG Pathway 2.825
BMD Lowest GO Term 0.279

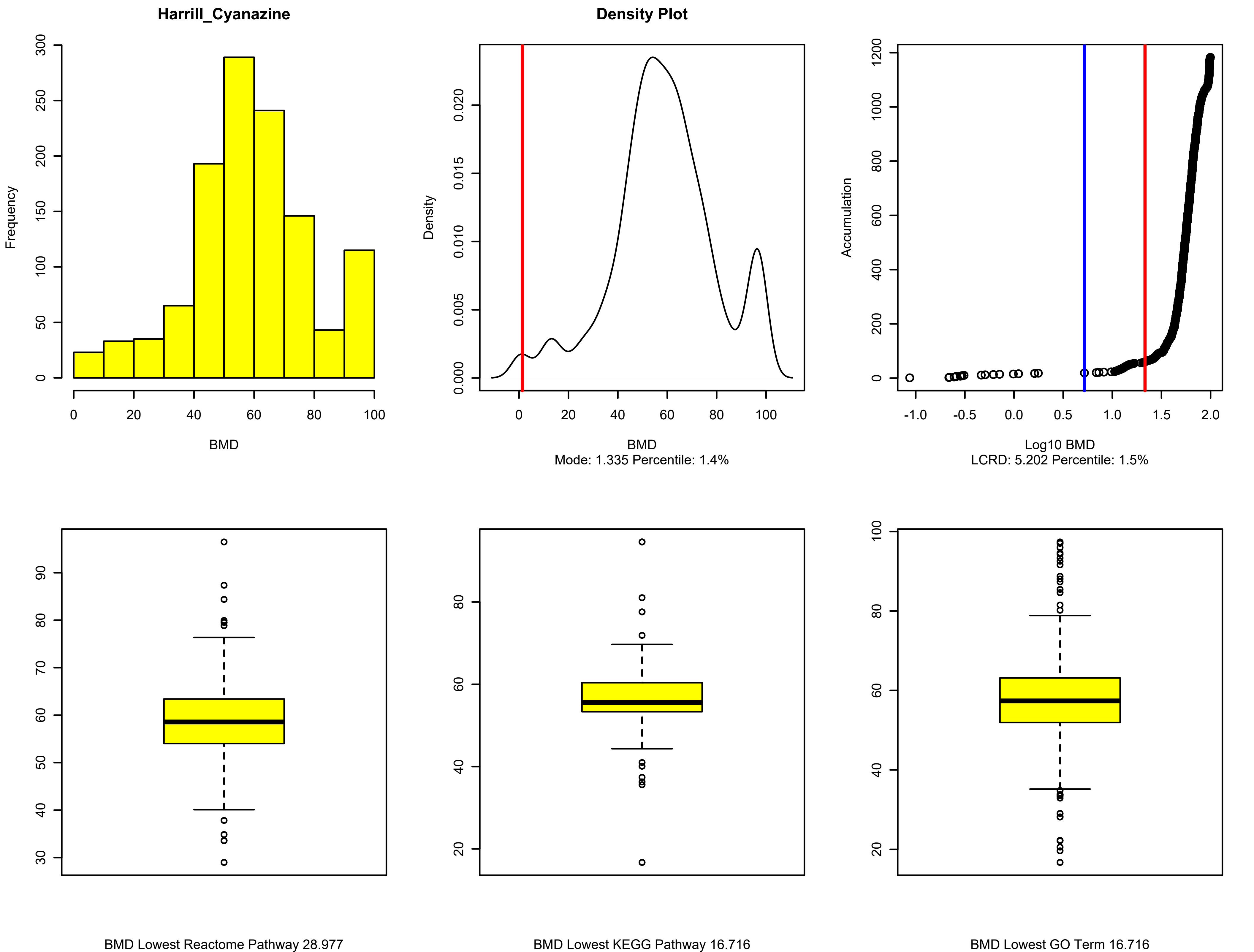

Harrill_Cyanazine
Frequency
BMD
Density Plot
Density
BMD
Mode: 1.335 Percentile: 1.4%
Accumulation
Log10 BMD
LCRD: 5.202 Percentile: 1.5%
BMD Lowest Reactome Pathway 28.977
BMD Lowest KEGG Pathway 16.716
BMD Lowest GO Term 16.716

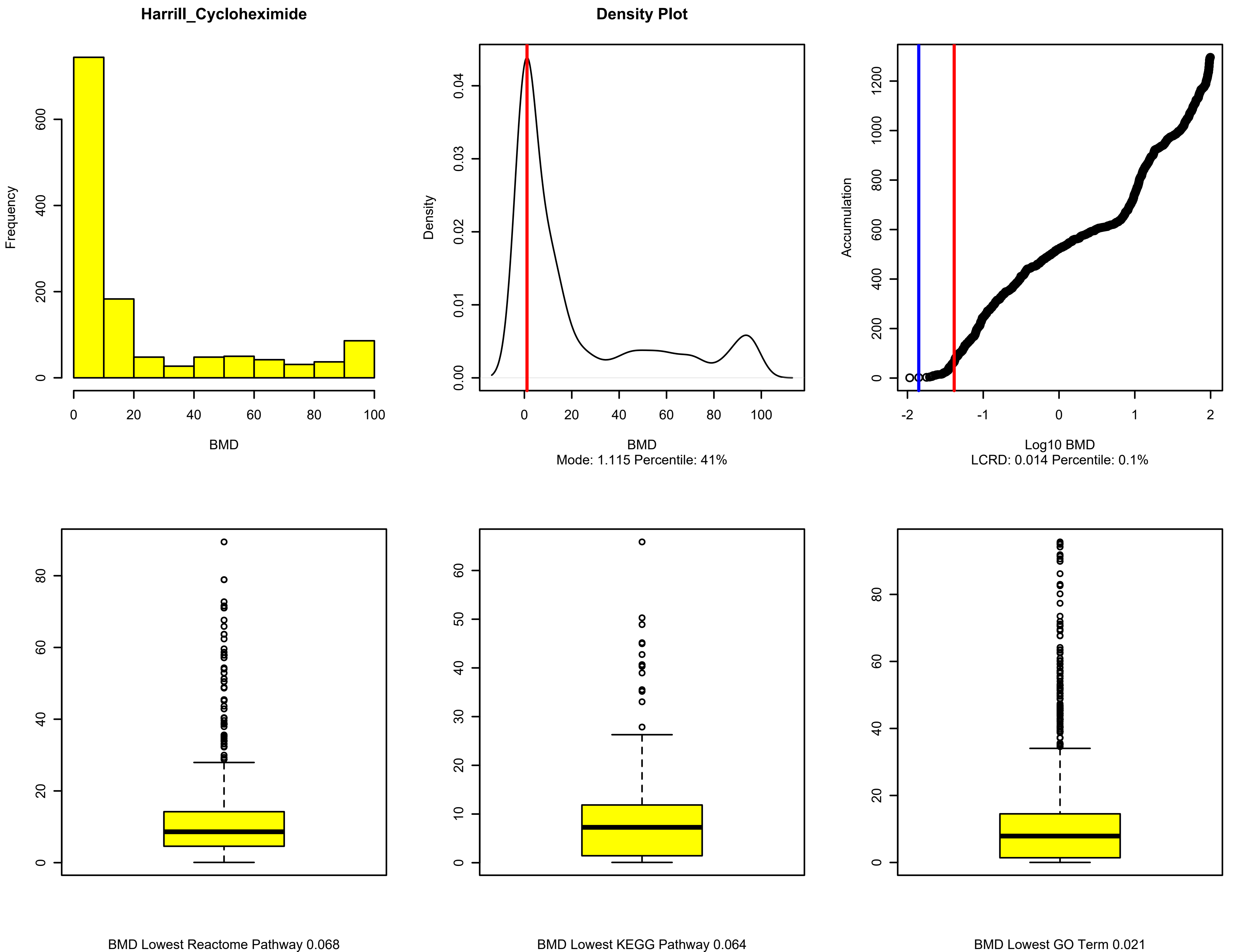
Harrill_Cycloheximide
Frequency
BMD
Density Plot
Density
BMD
Mode: 1.115 Percentile: 41%
Accumulation
Log10 BMD
LCRD: 0.014 Percentile: 0.1%
BMD Lowest Reactome Pathway 0.068
BMD Lowest KEGG Pathway 0.064
BMD Lowest GO Term 0.021

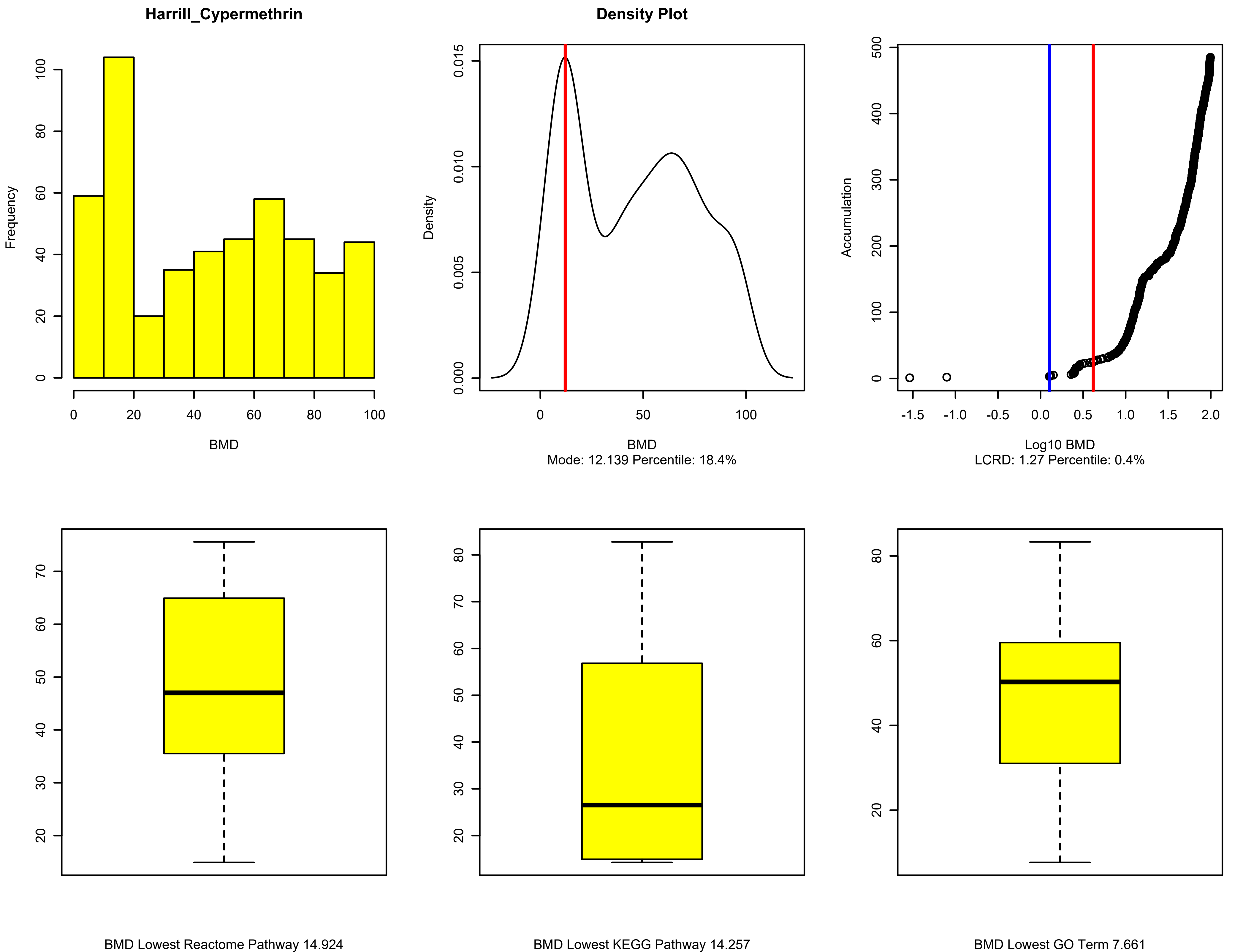

Harrill_Cypermethrin
Frequency
BMD
Density Plot
Density
BMD
Mode: 12.139 Percentile: 18.4%
Accumulation
Log10 BMD
LCRD: 1.27 Percentile: 0.4%
BMD Lowest Reactome Pathway 14.924
BMD Lowest KEGG Pathway 14.257
BMD Lowest GO Term 7.661

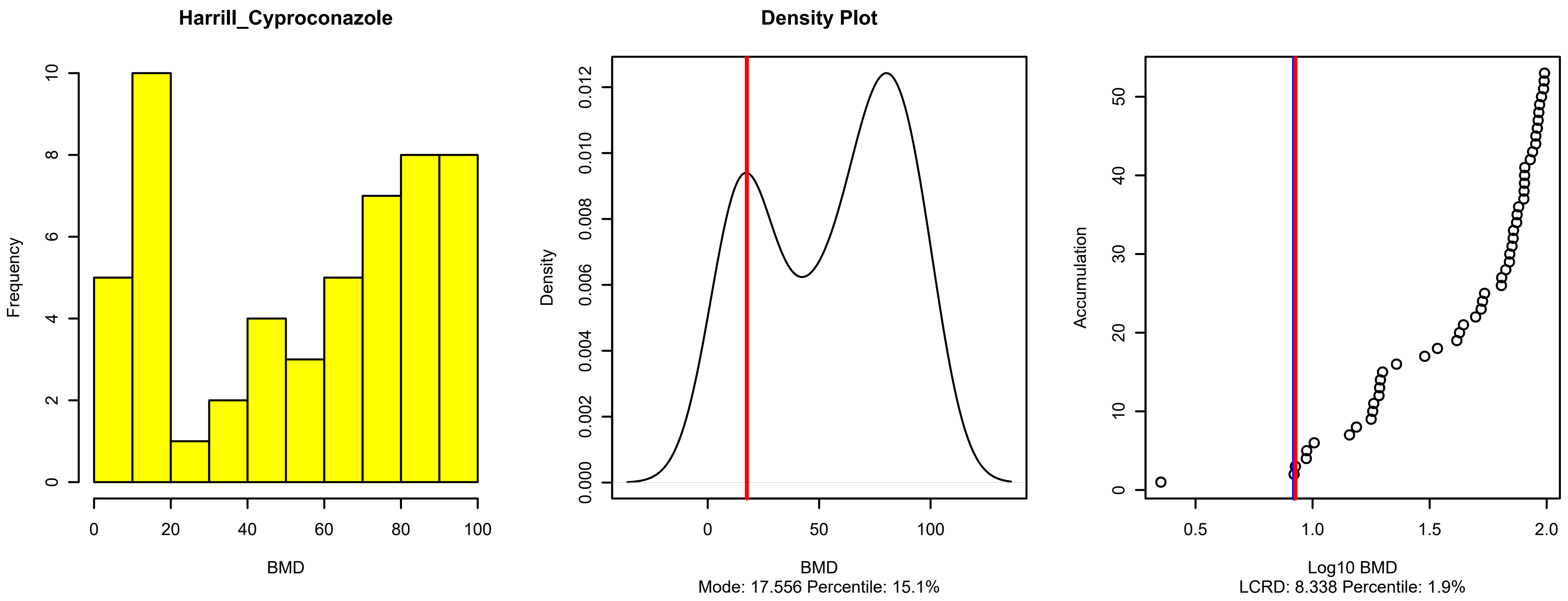

Harrill_Cyproconazole
Frequency
BMD
Density Plot
Density
BMD
Mode: 17.556 Percentile: 15.1%
Accumulation
Log10 BMD
LCRD: 8.338 Percentile: 1.9%

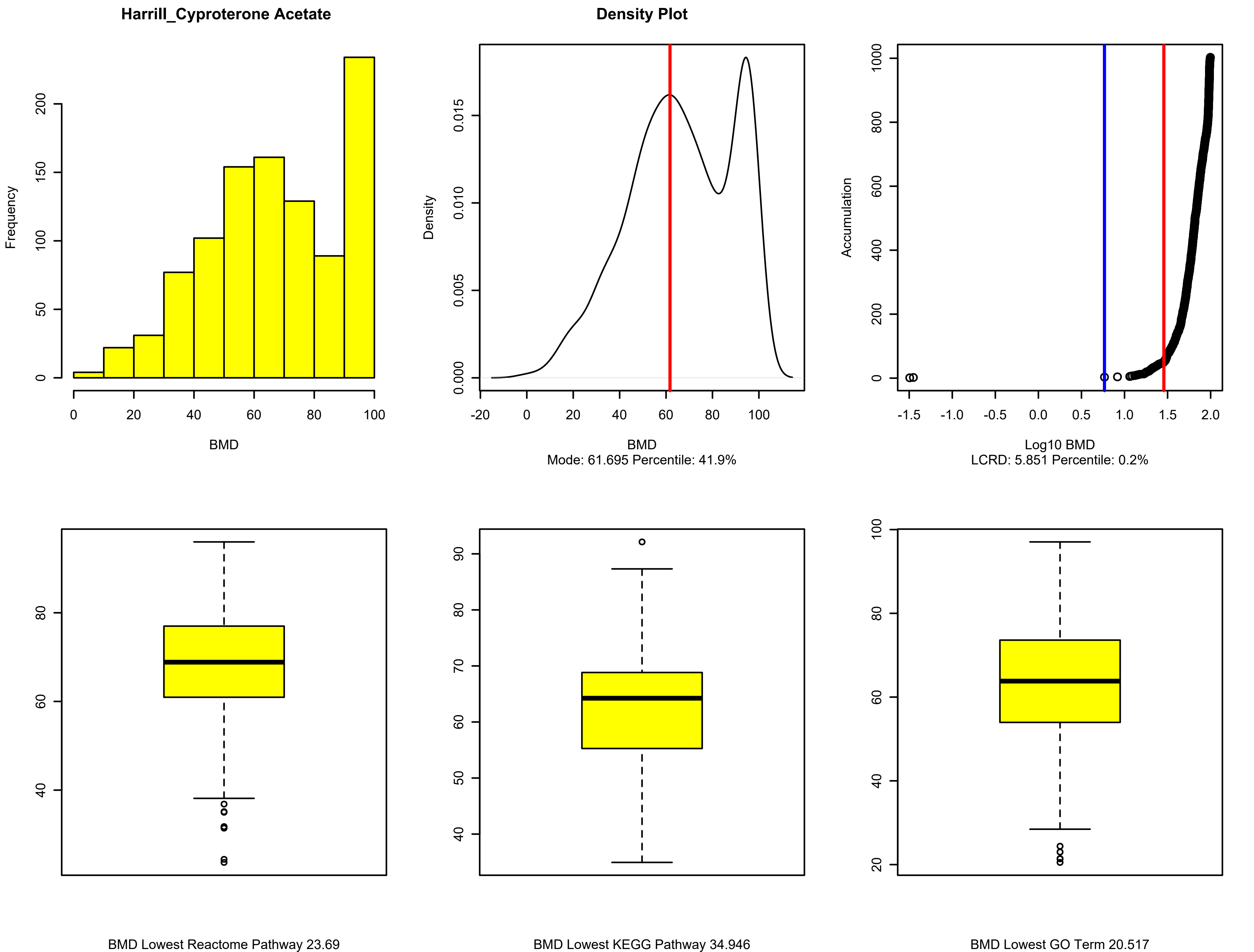
Harrill_Cyproterone Acetate
Frequency
BMD
Density Plot
Density
BMD
Mode: 61.695 Percentile: 41.9%
Accumulation
Log10 BMD
LCRD: 5.851 Percentile: 0.2%
BMD Lowest Reactome Pathway 23.69
BMD Lowest KEGG Pathway 34.946
BMD Lowest GO Term 20.517

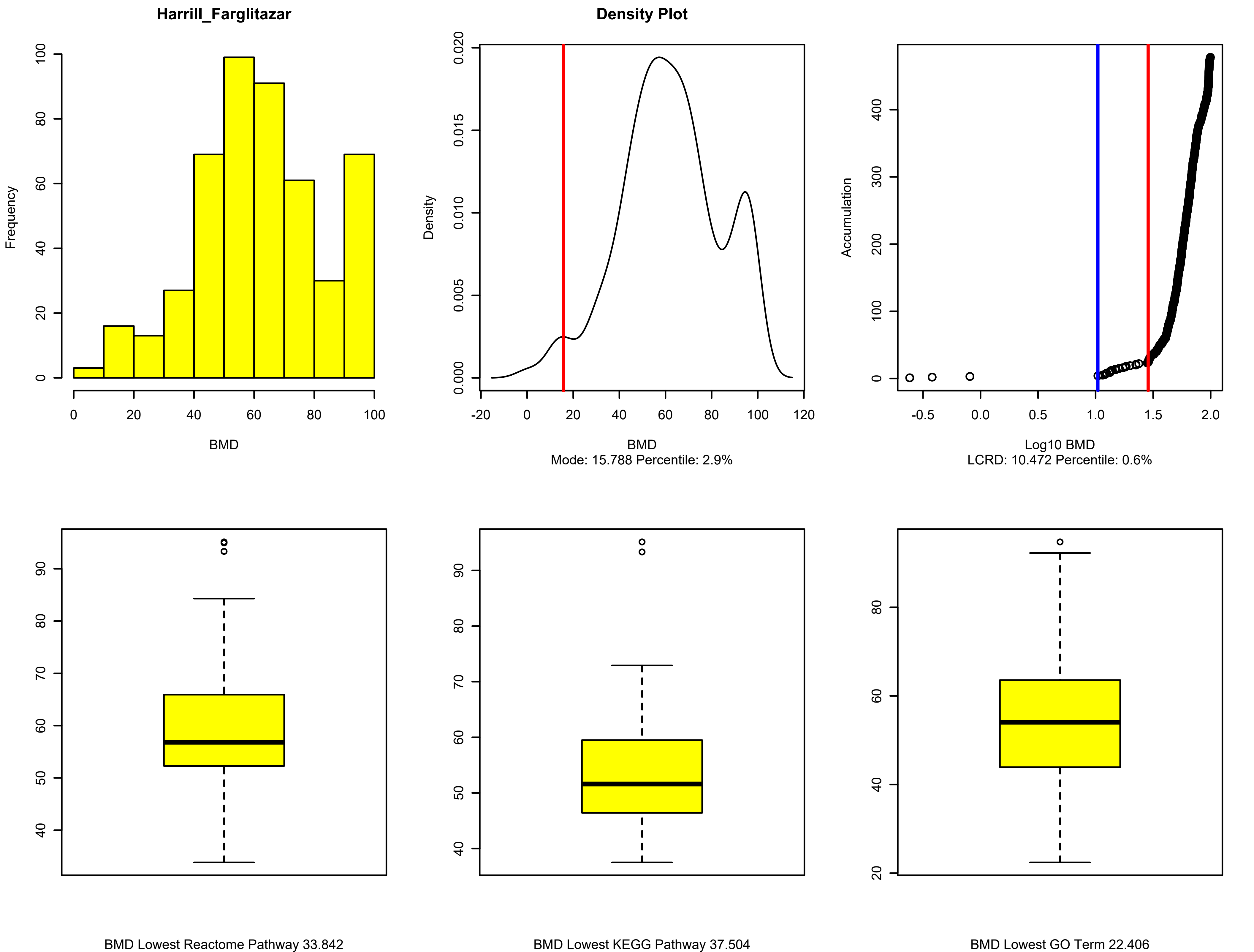
Harrill_Farglitazar
Frequency
BMD
Density Plot
Density
BMD
Mode: 15.788 Percentile: 2.9%
Accumulation
Log10 BMD
LCRD: 10.472 Percentile: 0.6%
BMD Lowest Reactome Pathway 33.842
BMD Lowest KEGG Pathway 37.504
BMD Lowest GO Term 22.406

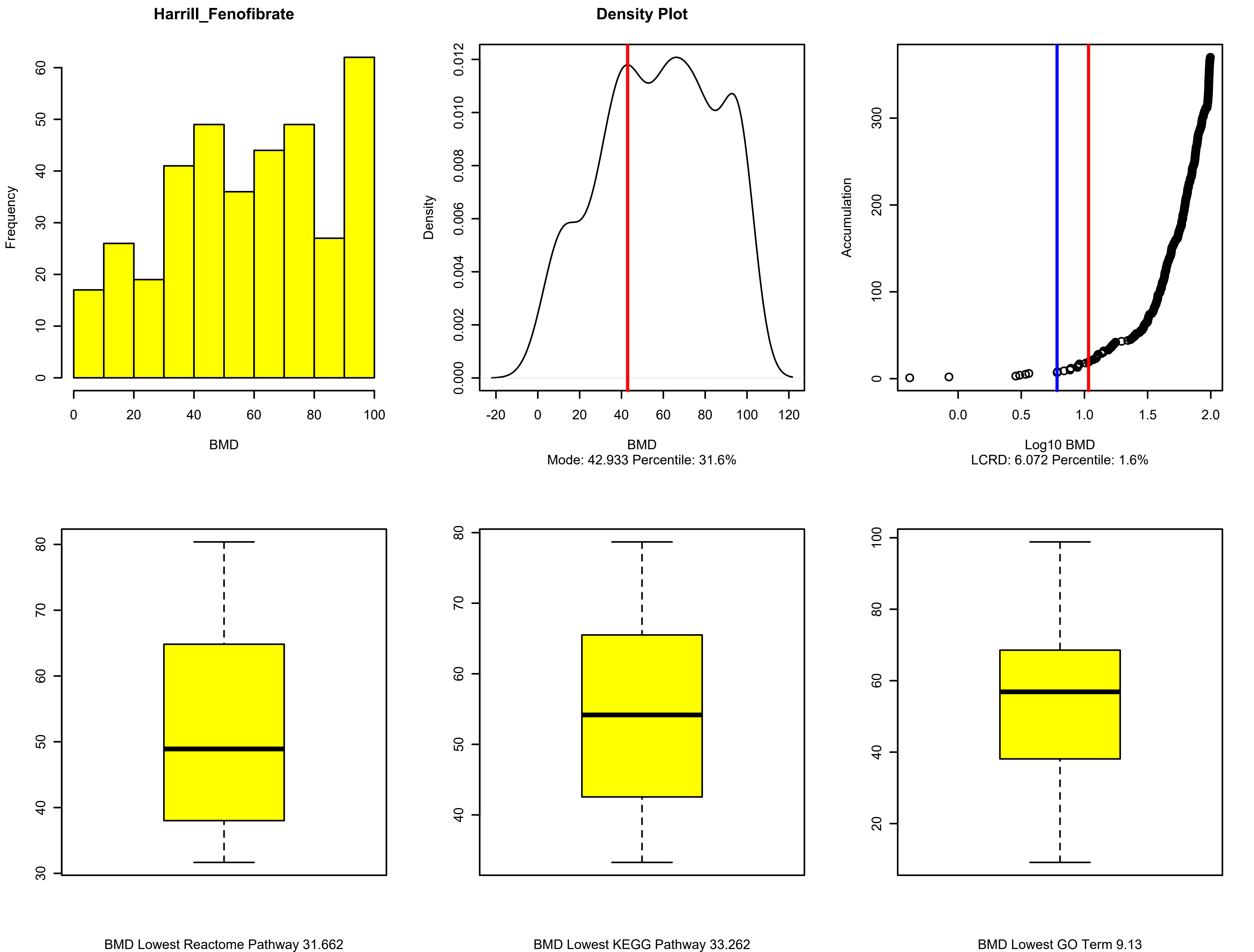

Harrill_Fenofibrate
Frequency
BMD
Density Plot
Density
BMD
Mode: 42.933 Percentile: 31.6%
Accumulation
Log10 BMD
LCRD: 6.072 Percentile: 1.6%
BMD Lowest Reactome Pathway 31.662
BMD Lowest KEGG Pathway 33.262
BMD Lowest GO Term 9.13

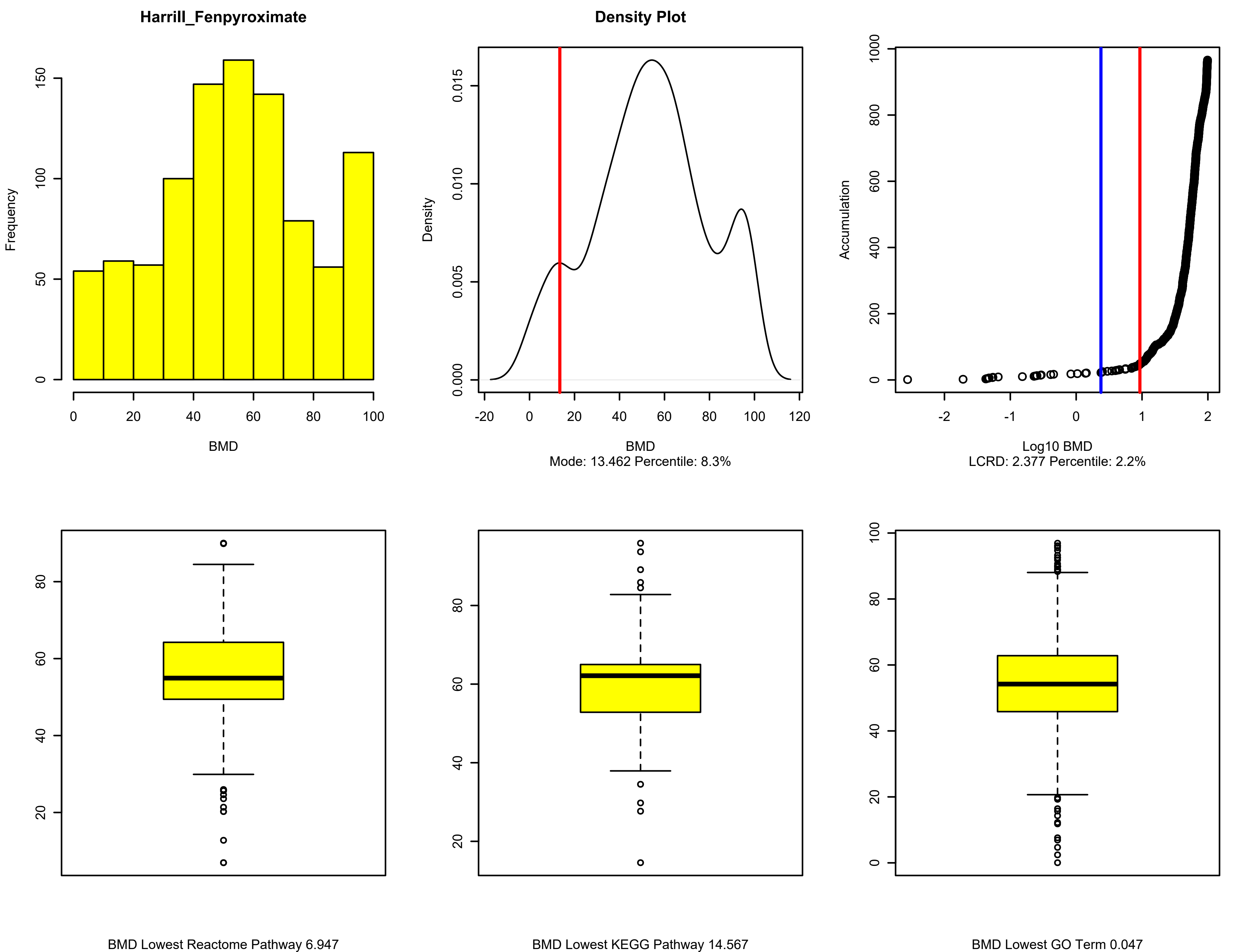
Harrill_Fenpyroximate
Frequency
BMD
Density Plot
Density
BMD
Mode: 13.462 Percentile: 8.3%
Accumulation
Log10 BMD
LCRD: 2.377 Percentile: 2.2%
BMD Lowest Reactome Pathway 6.947
BMD Lowest KEGG Pathway 14.567
BMD Lowest GO Term 0.047

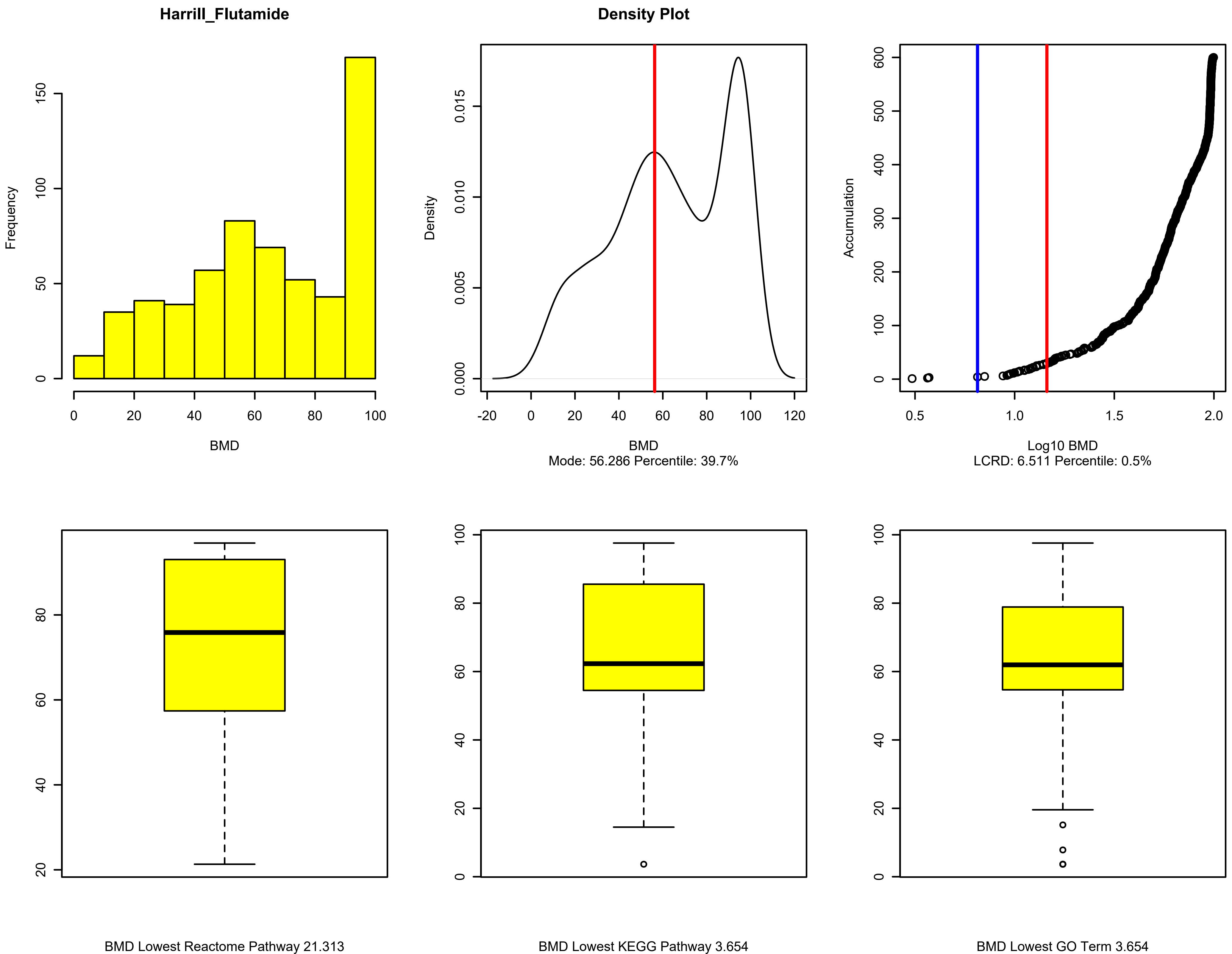

Harrill_Flutamide
Frequency
BMD
Density Plot
Density
BMD
Mode: 56.286 Percentile: 39.7%
Accumulation
Log10 BMD
LCRD: 6.511 Percentile: 0.5%
BMD Lowest Reactome Pathway 21.313
BMD Lowest KEGG Pathway 3.654
BMD Lowest GO Term 3.654

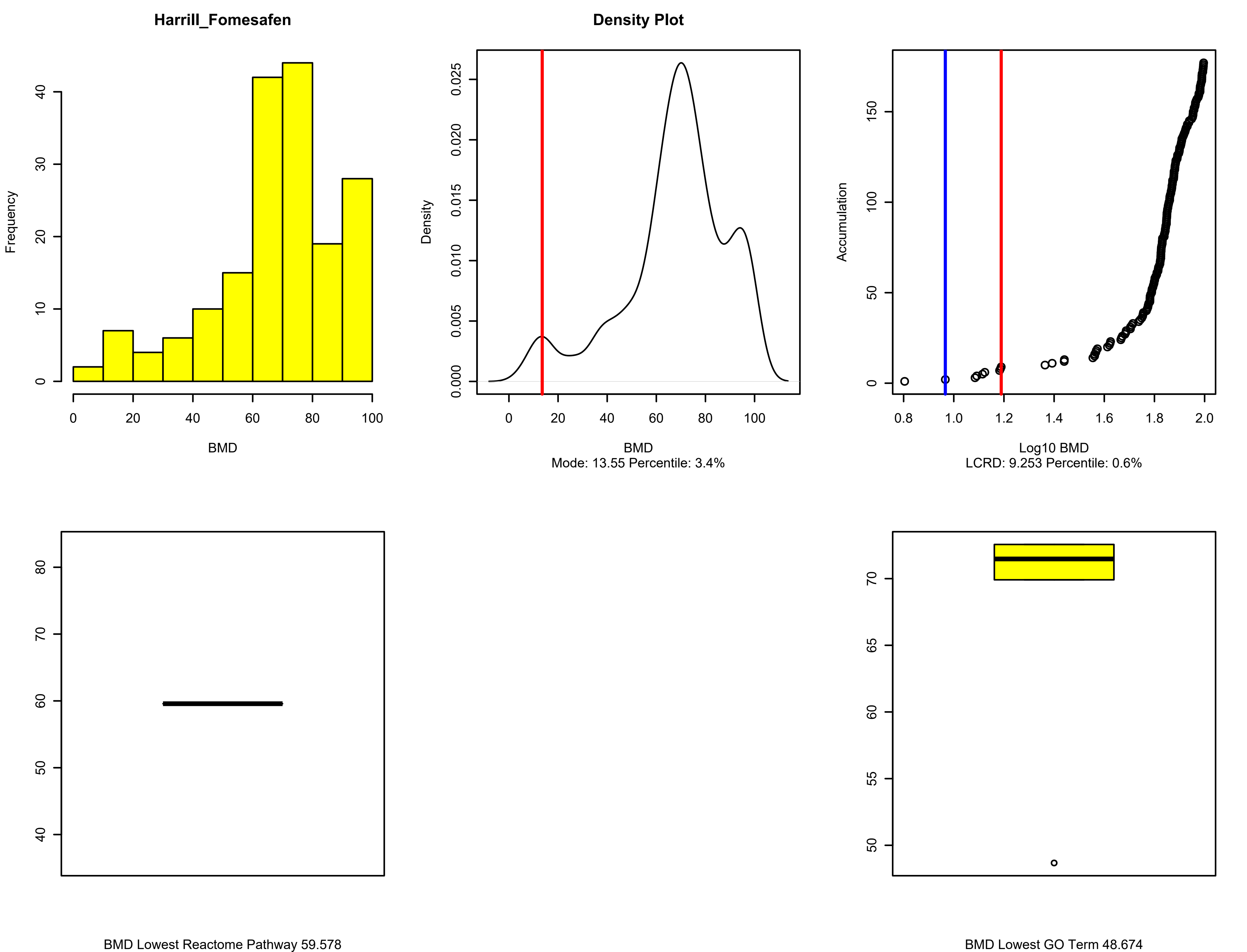

Harrill_Fomesafen
Frequency
BMD
Density Plot
Density
BMD
Mode: 13.55 Percentile: 3.4%
Accumulation
Log10 BMD
LCRD: 9.253 Percentile: 0.6%
BMD Lowest Reactome Pathway 59.578
BMD Lowest GO Term 48.674

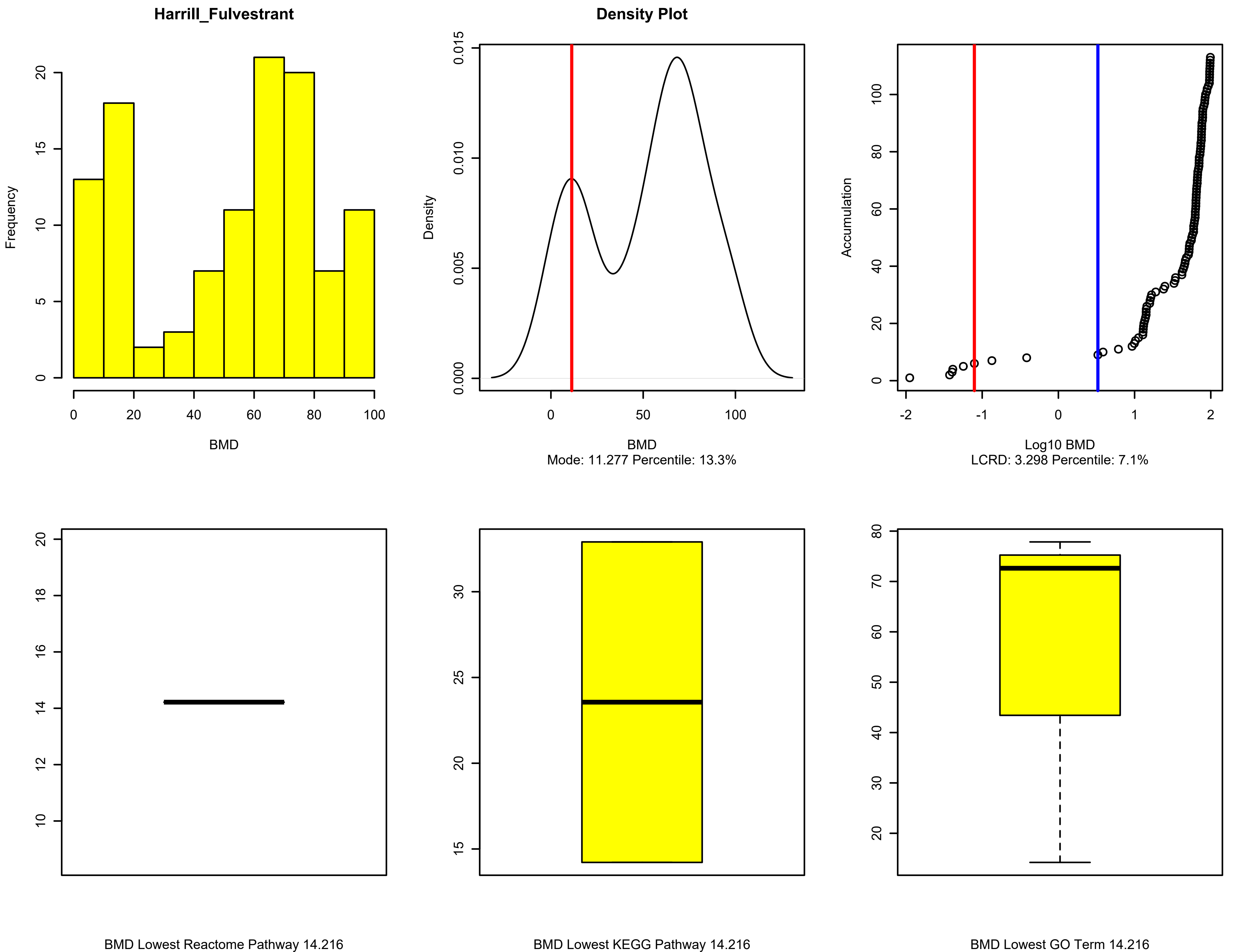

Harrill_Fulvestrant
Frequency
BMD
Density Plot
Density
BMD
Mode: 11.277 Percentile: 13.3%
Accumulation
Log10 BMD
LCRD: 3.298 Percentile: 7.1%
BMD Lowest Reactome Pathway 14.216
BMD Lowest KEGG Pathway 14.216
BMD Lowest GO Term 14.216

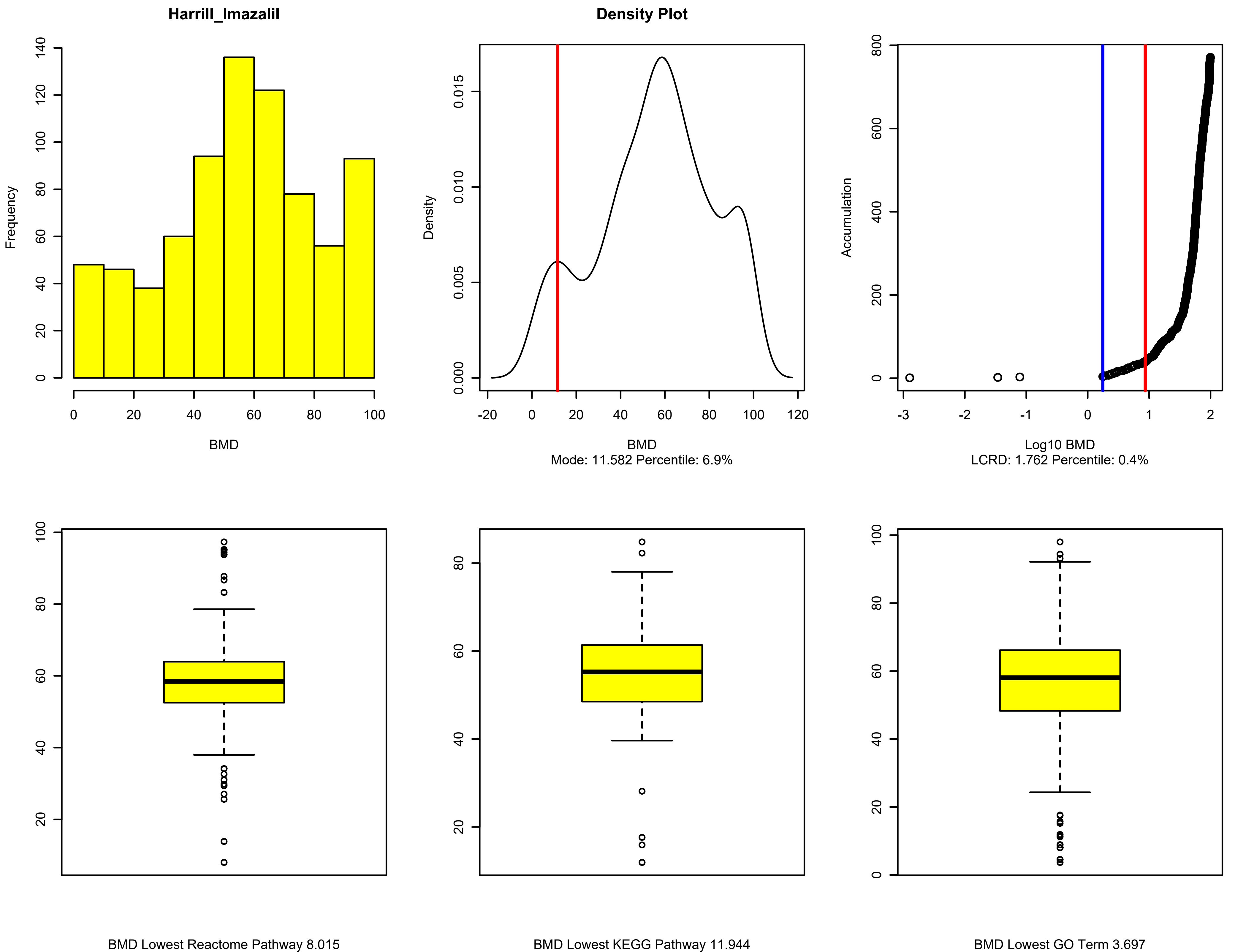
Harrill_Imazalil
Frequency
BMD
Density Plot
Density
BMD
Mode: 11.582 Percentile: 6.9%
Accumulation
Log10 BMD
LCRD: 1.762 Percentile: 0.4%
BMD Lowest Reactome Pathway 8.015
BMD Lowest KEGG Pathway 11.944
BMD Lowest GO Term 3.697

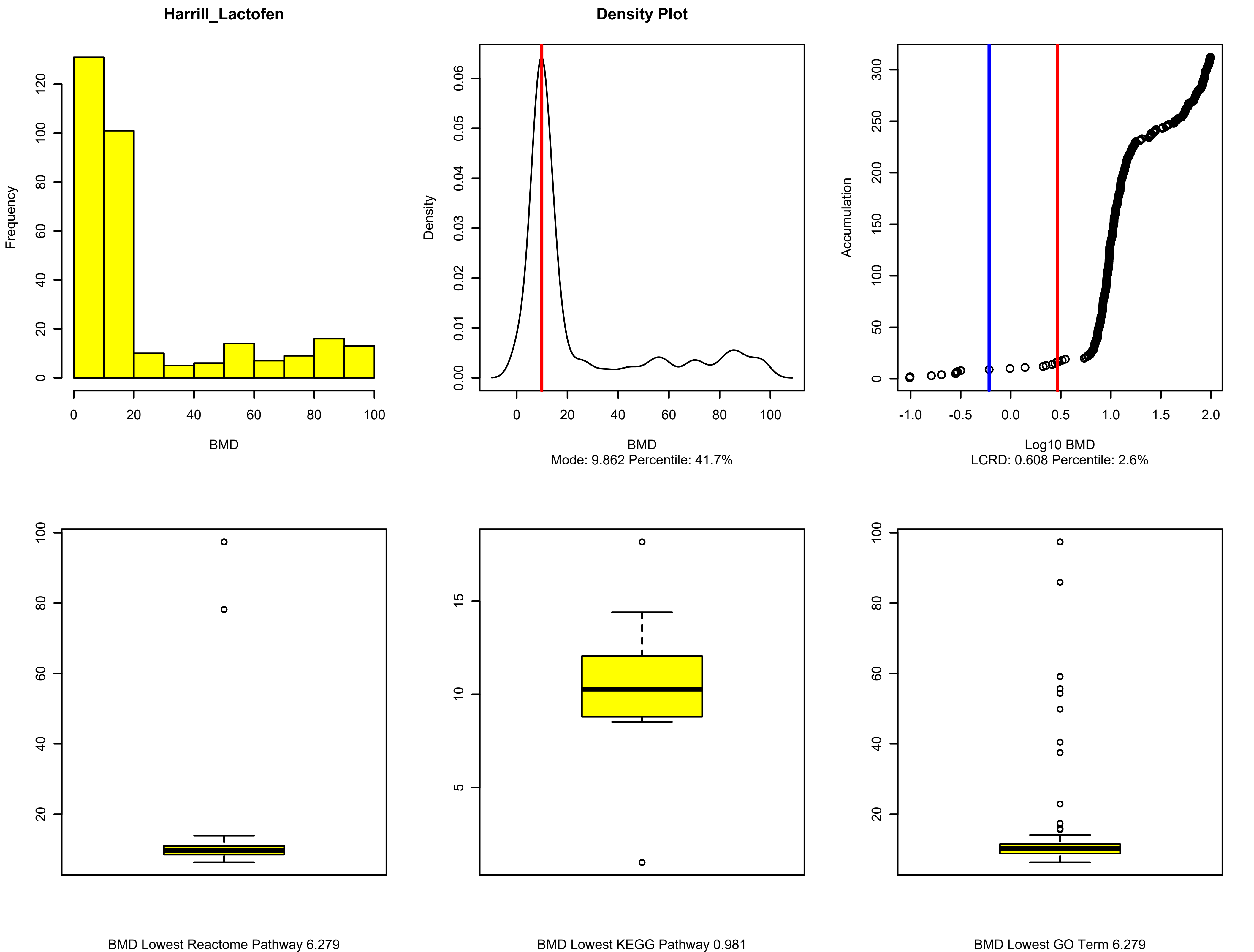

Harrill_Lactofen
Frequency
BMD
Density Plot
Density
BMD
Mode: 9.862 Percentile: 41.7%
Accumulation
Log10 BMD
LCRD: 0.608 Percentile: 2.6%
BMD Lowest Reactome Pathway 6.279
BMD Lowest KEGG Pathway 0.981
BMD Lowest GO Term 6.279

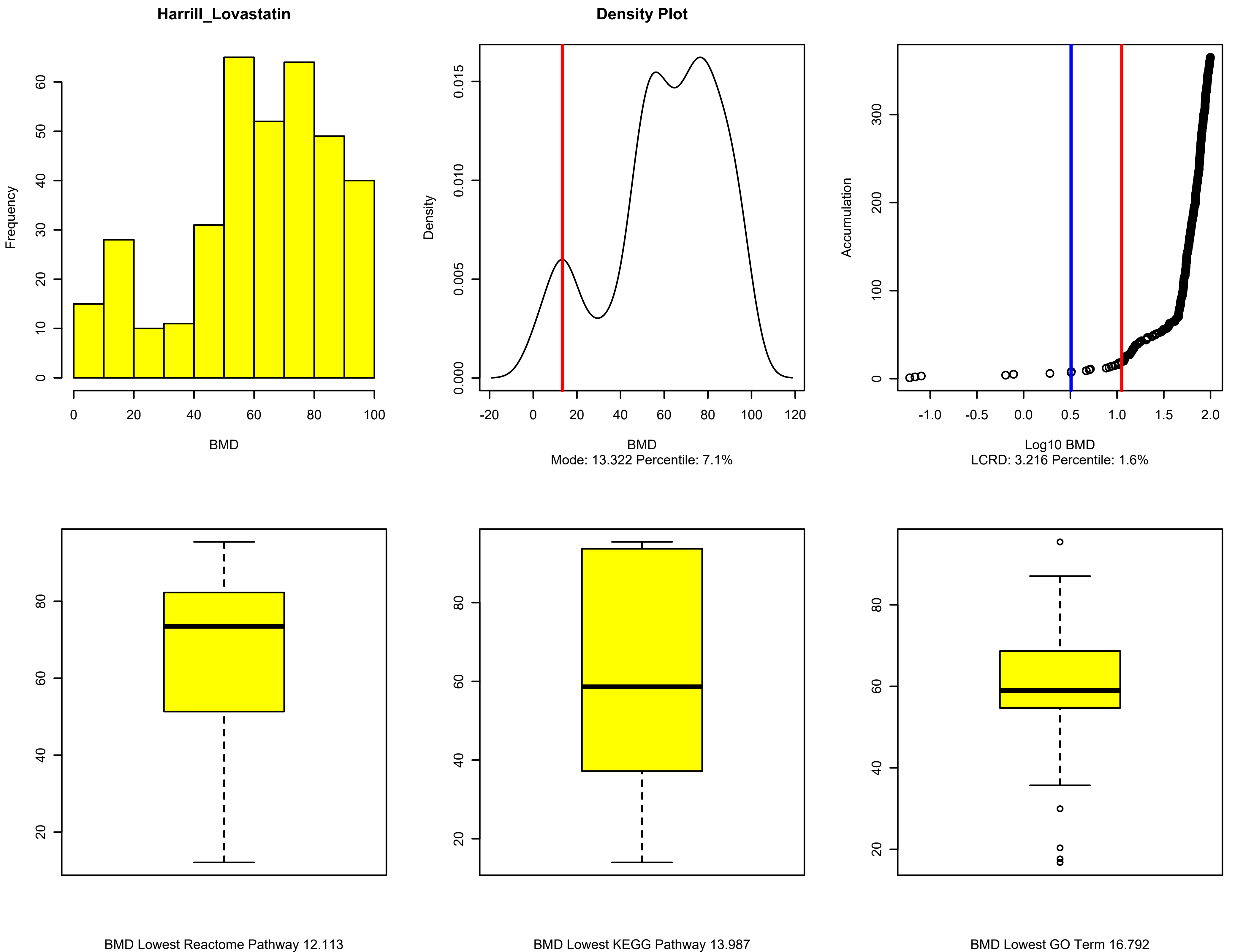

Harrill_Lovastatin
Frequency
BMD
Density Plot
Density
BMD
Mode: 13.322 Percentile: 7.1%
Accumulation
Log10 BMD
LCRD: 3.216 Percentile: 1.6%
BMD Lowest Reactome Pathway 12.113
BMD Lowest KEGG Pathway 13.987
BMD Lowest GO Term 16.792

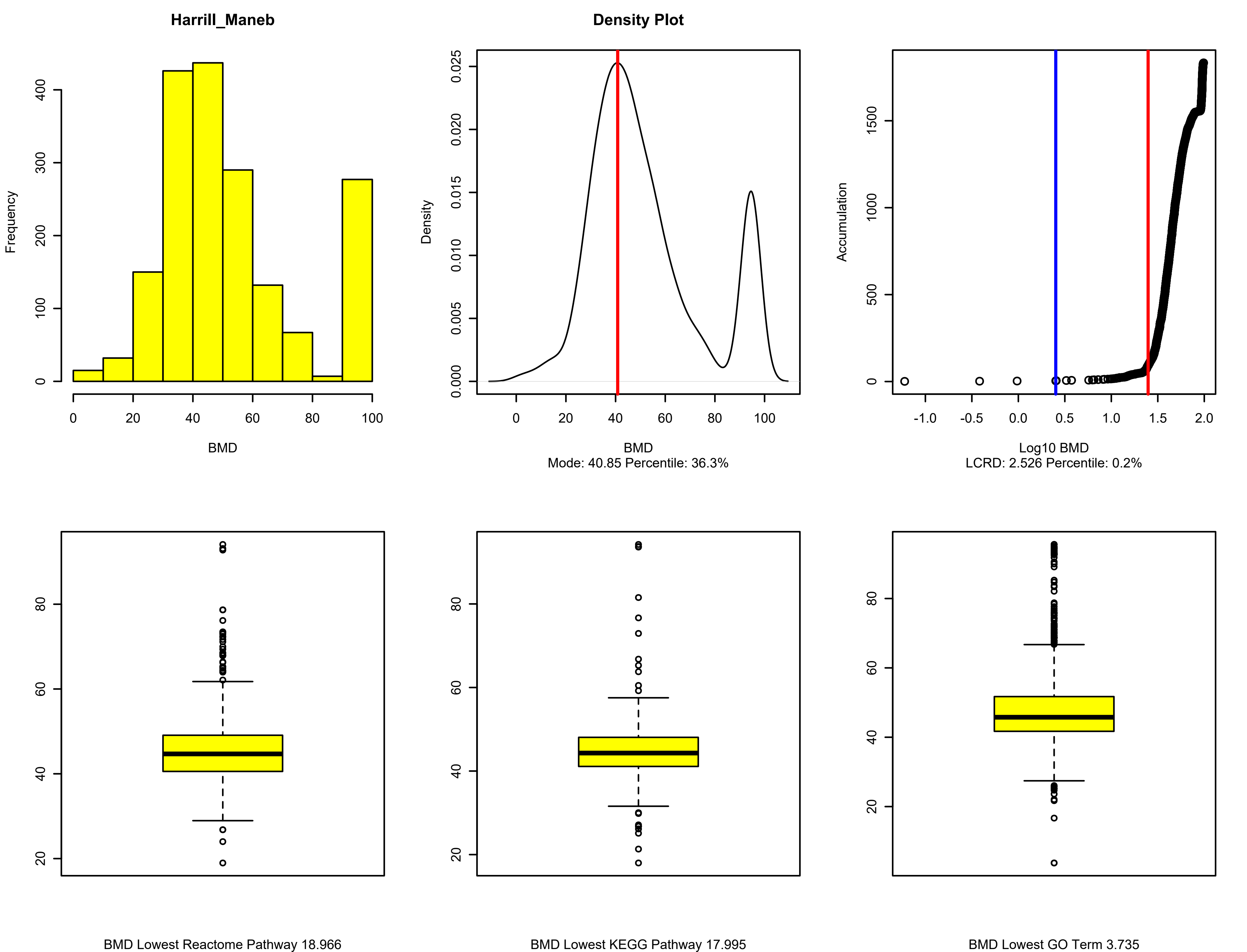
Harrill_Maneb
Frequency
BMD
Density Plot
Density
BMD
Mode: 40.85 Percentile: 36.3%
Accumulation
Log10 BMD
LCRD: 2.526 Percentile: 0.2%
BMD Lowest Reactome Pathway 18.966
BMD Lowest KEGG Pathway 17.995
BMD Lowest GO Term 3.735

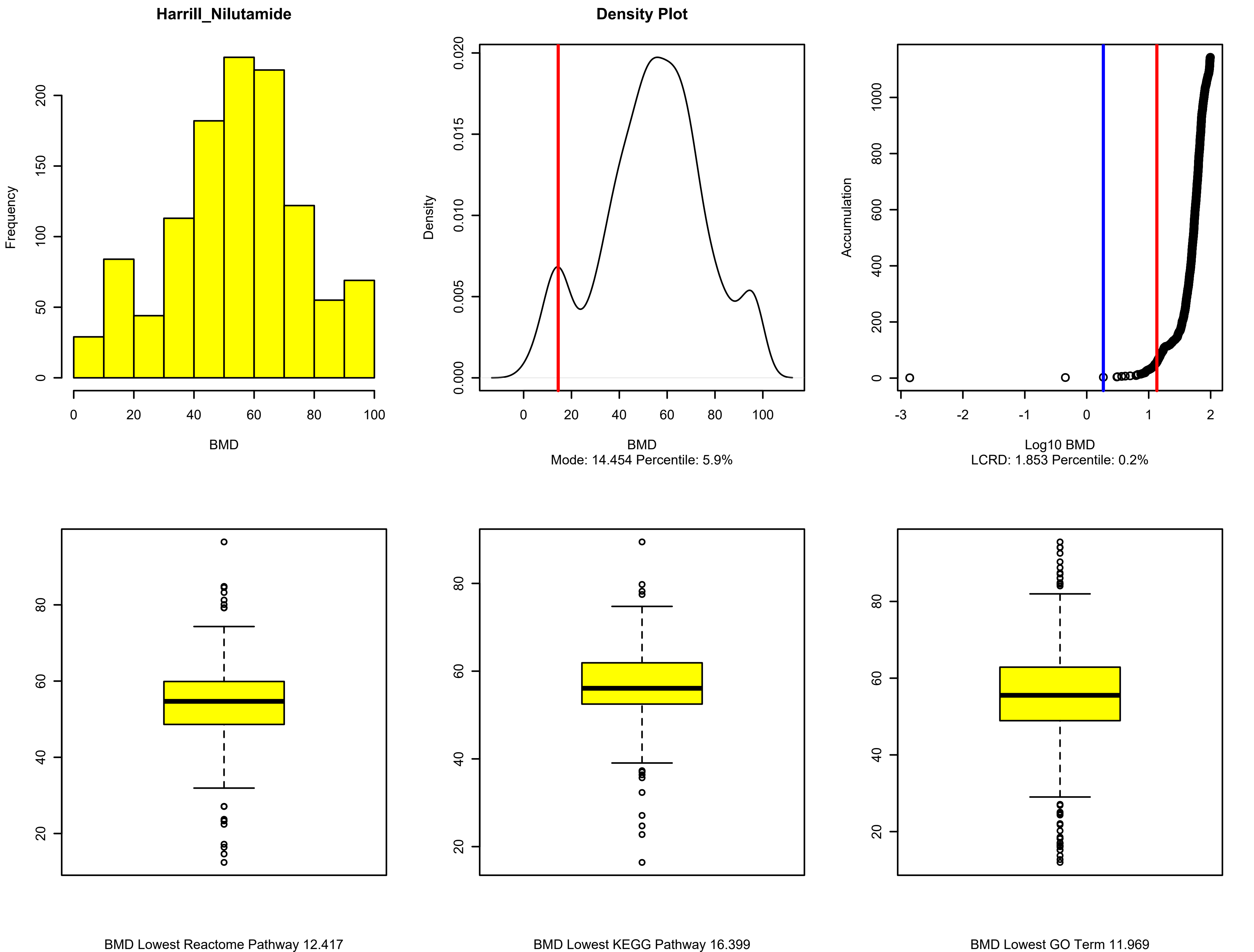
Harrill_Nilutamide
Frequency
BMD
Density Plot
Density
BMD
Mode: 14.454 Percentile: 5.9%
Accumulation
Log10 BMD
LCRD: 1.853 Percentile: 0.2%
BMD Lowest Reactome Pathway 12.417
BMD Lowest KEGG Pathway 16.399
BMD Lowest GO Term 11.969

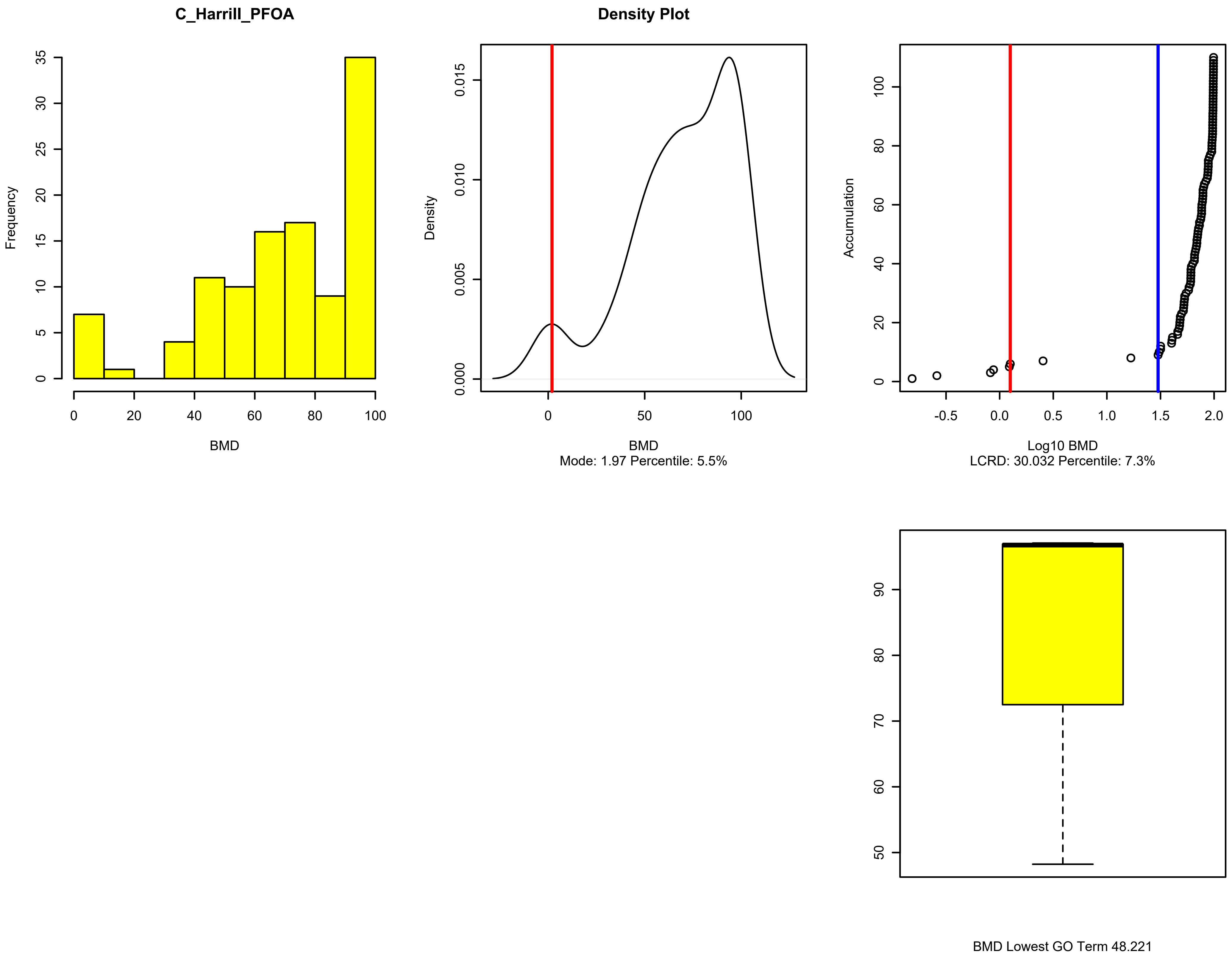

C_Harrill_PFOA
Frequency
BMD
Density Plot
Density
BMD
Mode: 1.97 Percentile: 5.5%
Accumulation
Log10 BMD
LCRD: 30.032 Percentile: 7.3%
BMD Lowest GO Term 48.221

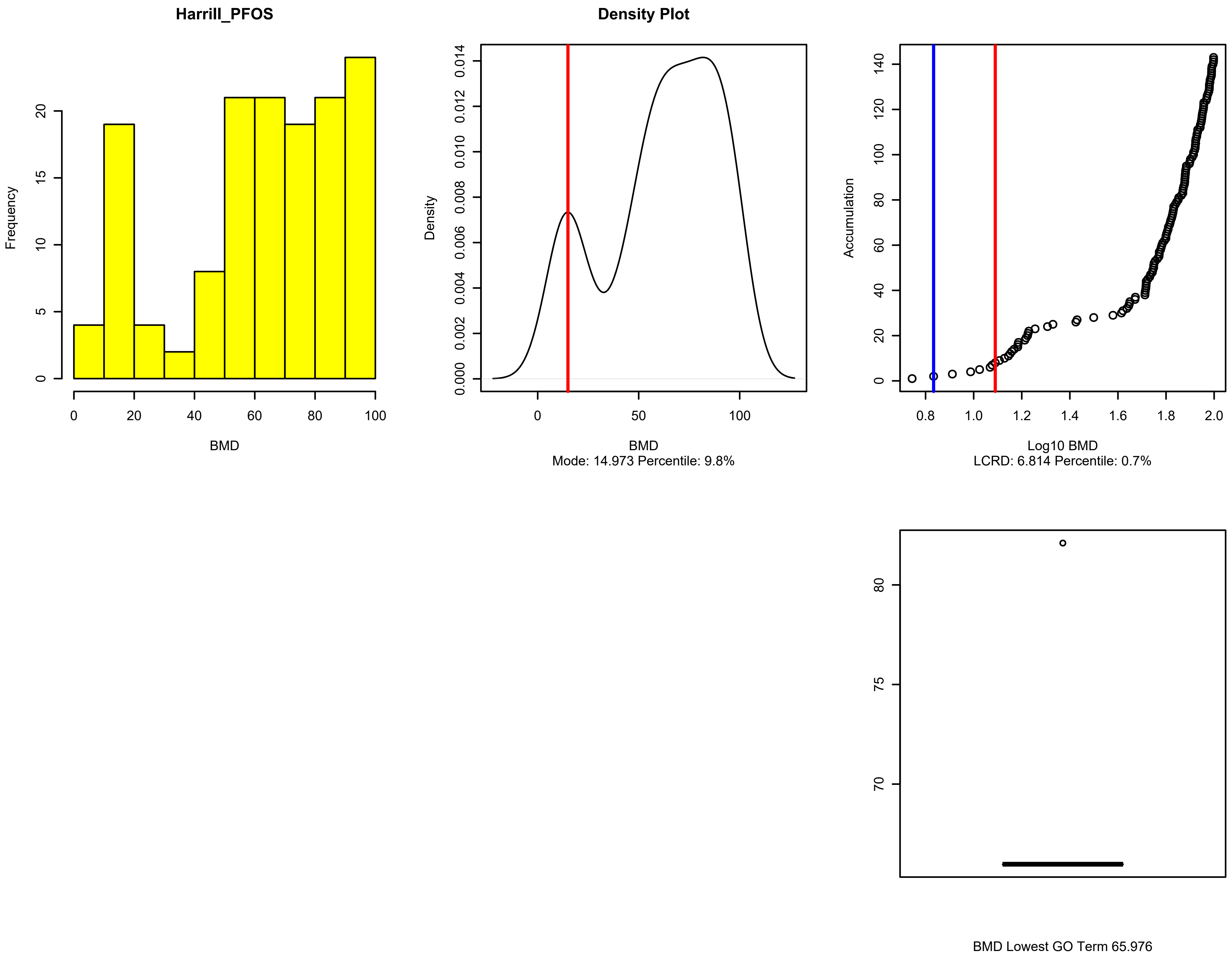
Harrill_PFOS
Frequency
BMD
Density Plot
Density
BMD
Mode: 14.973 Percentile: 9.8%
Accumulation
Log10 BMD
LCRD: 6.814 Percentile: 0.7%
BMD Lowest GO Term 65.976

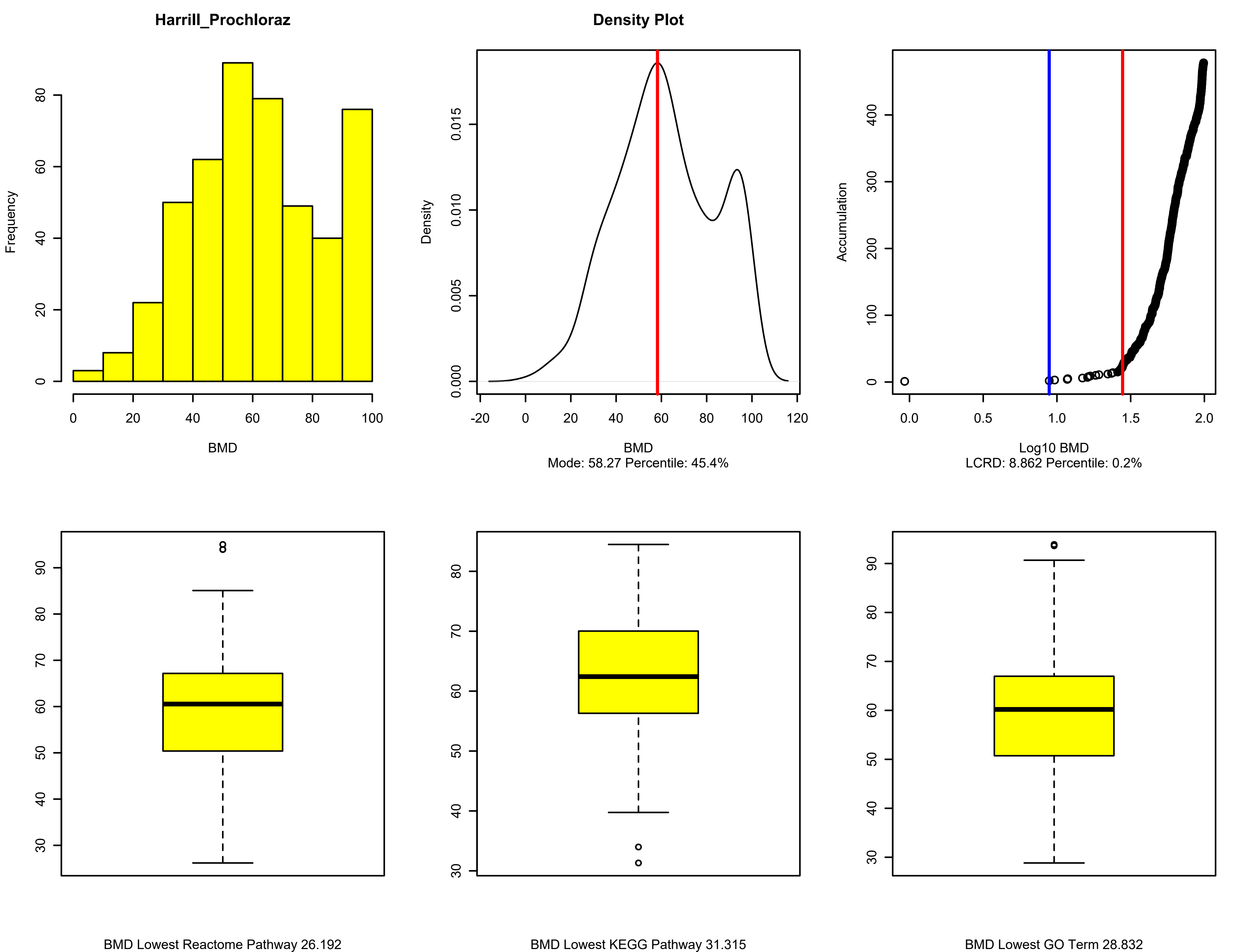
Harrill_Prochloraz
Frequency
BMD
Density Plot
Density
BMD
Mode: 58.27 Percentile: 45.4%
Accumulation
Log10 BMD
LCRD: 8.862 Percentile: 0.2%
BMD Lowest Reactome Pathway 26.192
BMD Lowest KEGG Pathway 31.315
BMD Lowest GO Term 28.832

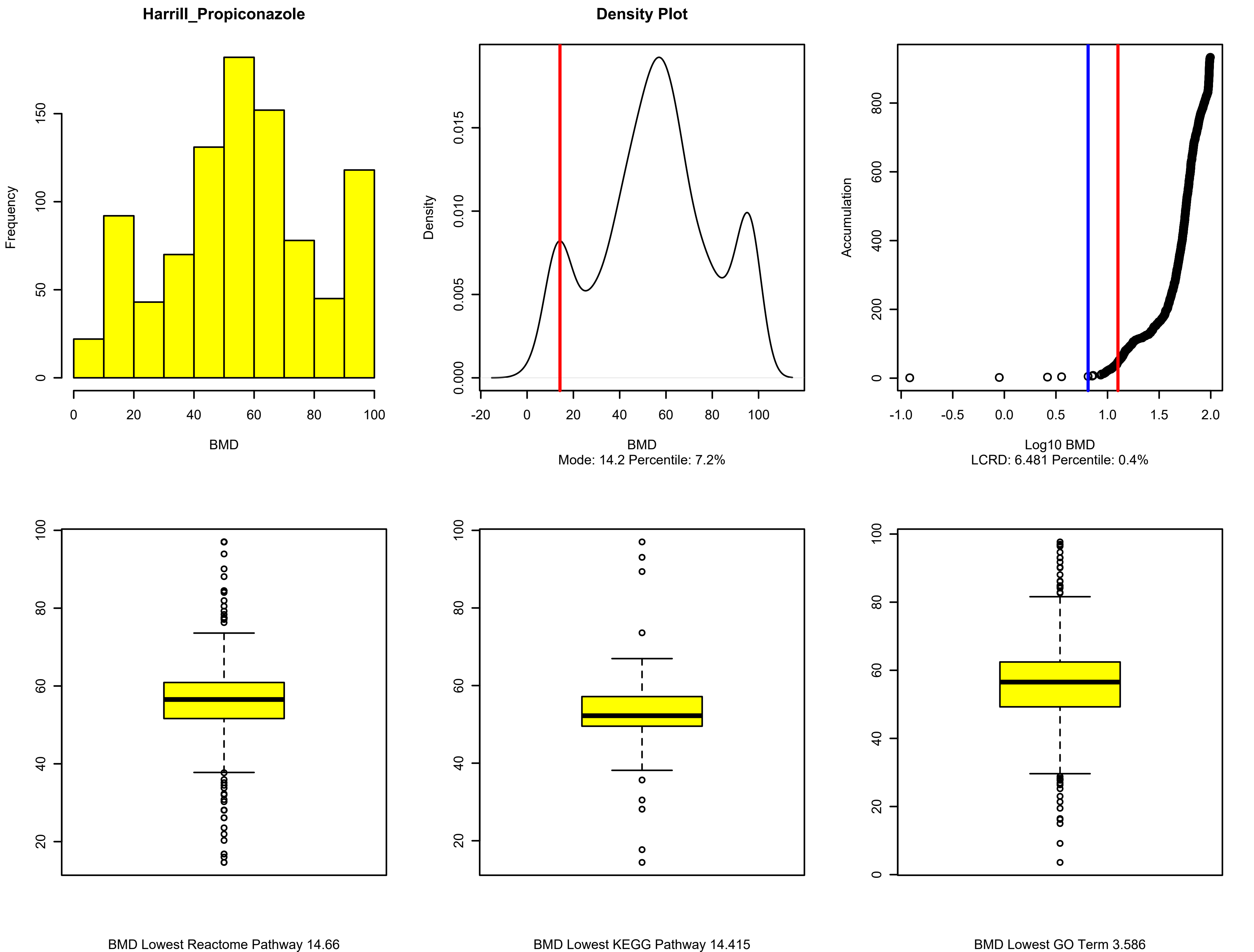
Harrill_Propiconazole
Frequency
BMD
Density Plot
Density
BMD
Mode: 14.2 Percentile: 7.2%
Accumulation
Log10 BMD
LCRD: 6.481 Percentile: 0.4%
BMD Lowest Reactome Pathway 14.66
BMD Lowest KEGG Pathway 14.415
BMD Lowest GO Term 3.586

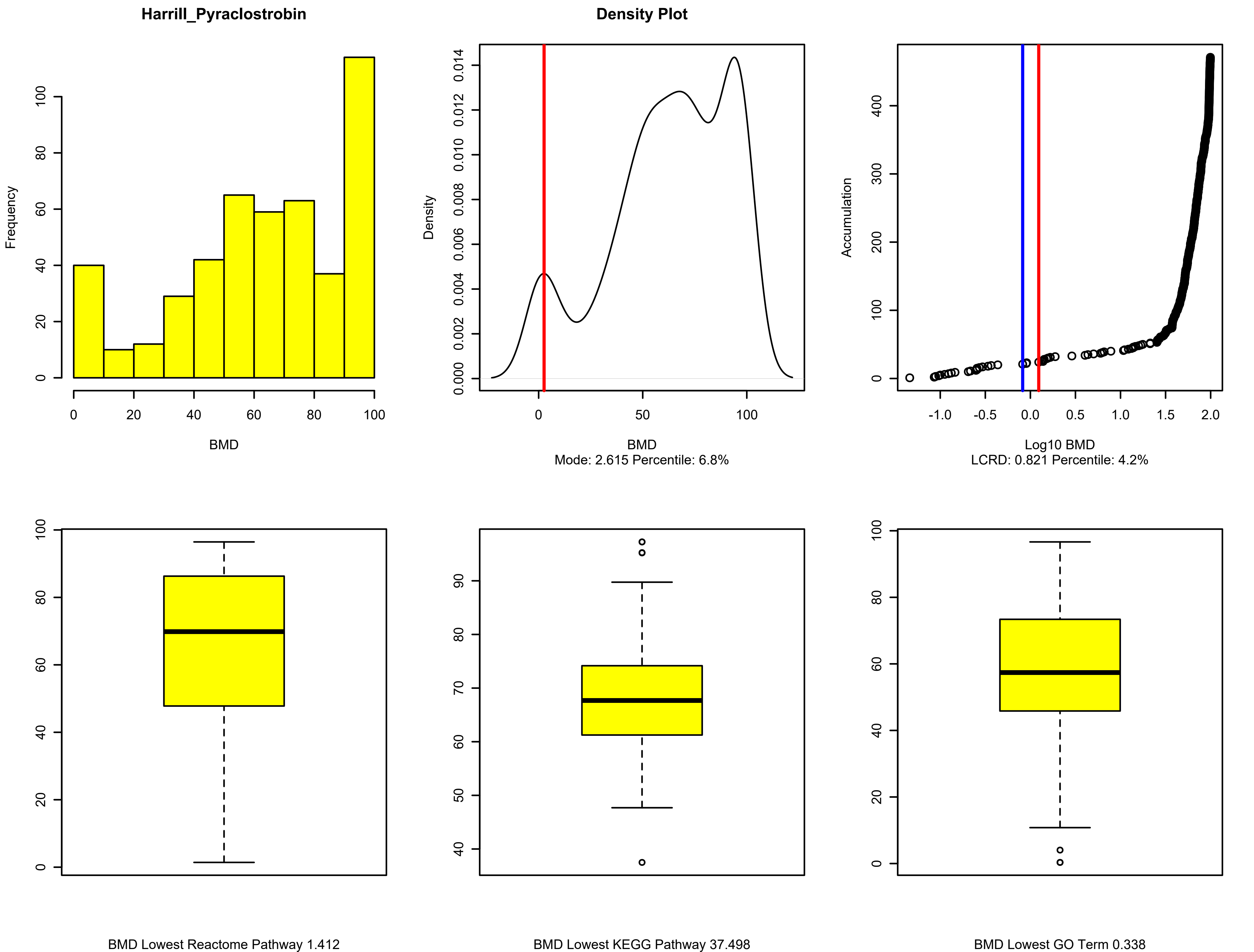

Harrill_Pyraclostrobin
Frequency
BMD
Density Plot
Density
BMD
Mode: 2.615 Percentile: 6.8%
Accumulation
Log10 BMD
LCRD: 0.821 Percentile: 4.2%
BMD Lowest Reactome Pathway 1.412
BMD Lowest KEGG Pathway 37.498
BMD Lowest GO Term 0.338

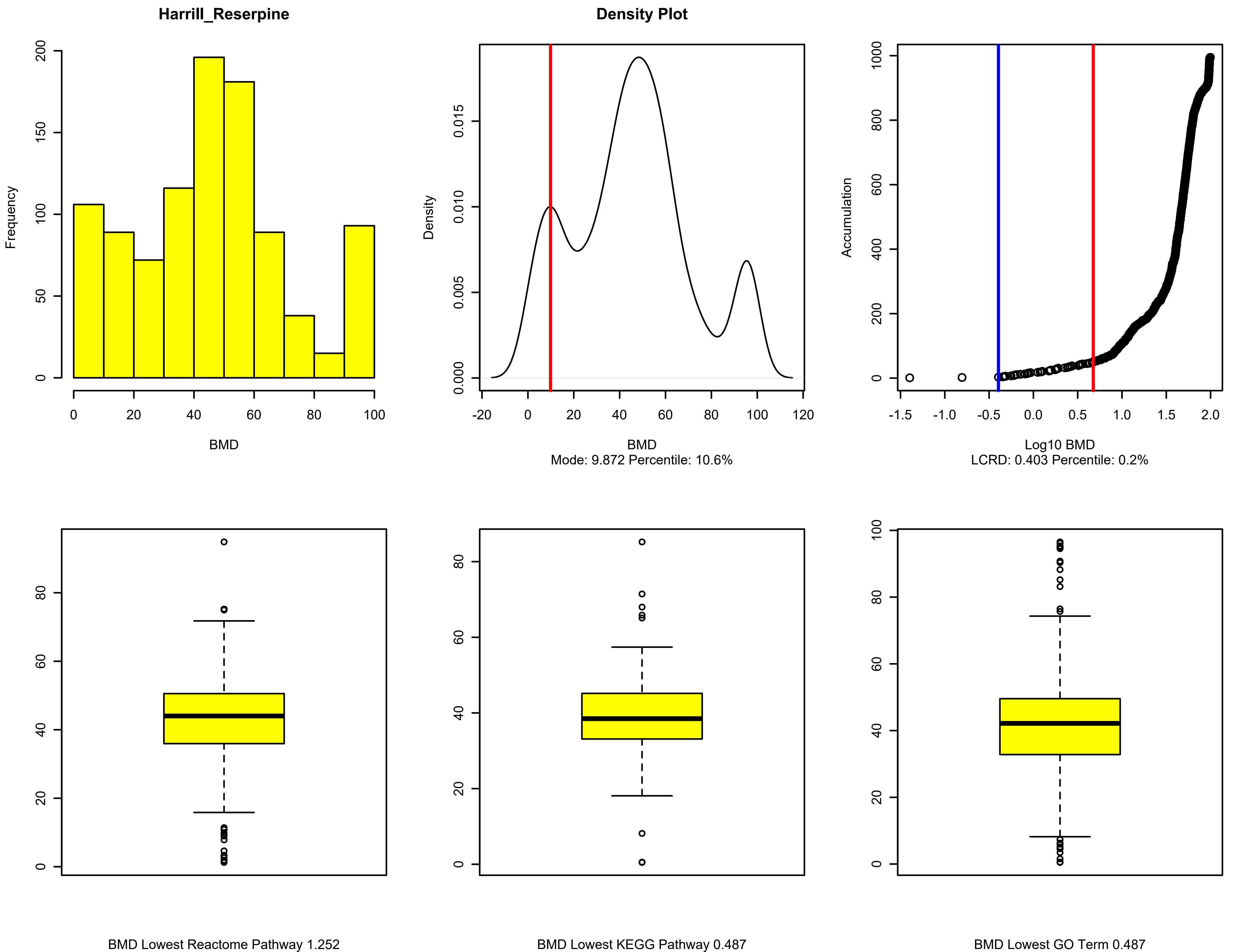

Harrill_Reserpine
Frequency
BMD
Density Plot
Density
BMD
Mode: 9.872 Percentile: 10.6%
Accumulation
Log10 BMD
LCRD: 0.403 Percentile: 0.2%
BMD Lowest Reactome Pathway 1.252
BMD Lowest KEGG Pathway 0.487
BMD Lowest GO Term 0.487

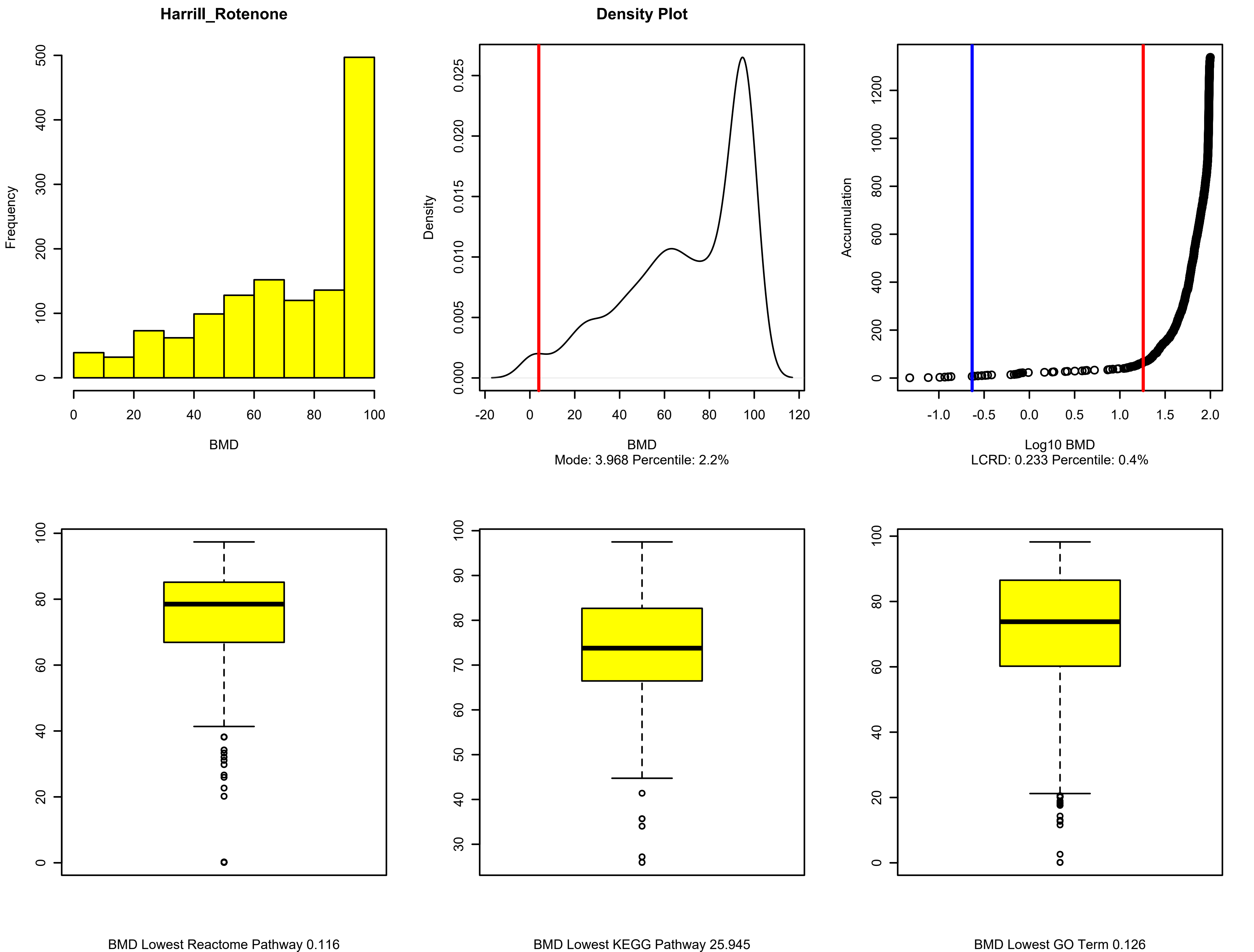

Harrill_Rotenone
Frequency
BMD
Density Plot
Density
BMD
Mode: 3.968 Percentile: 2.2%
Accumulation
Log10 BMD
LCRD: 0.233 Percentile: 0.4%
BMD Lowest Reactome Pathway 0.116
BMD Lowest KEGG Pathway 25.945
BMD Lowest GO Term 0.126

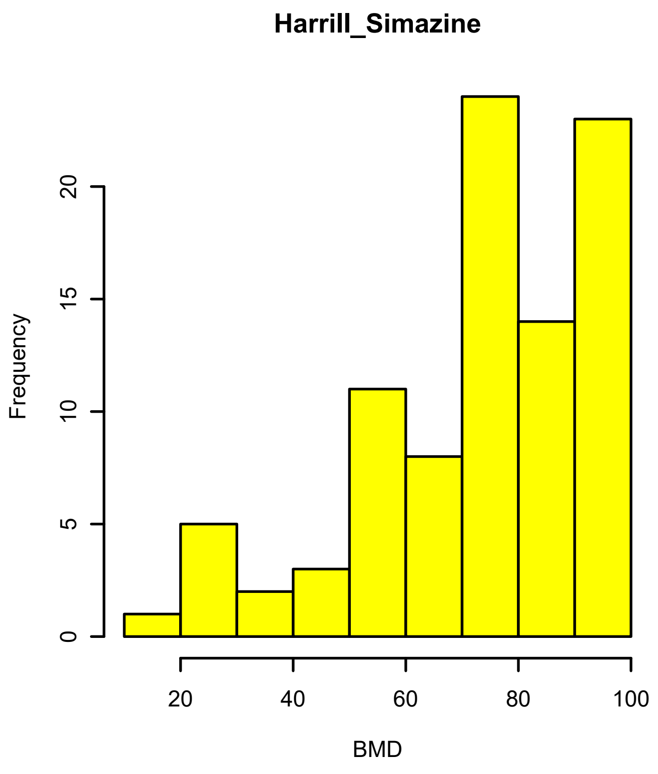
Harrill_Simazine
Frequency
0
5
10
15
20
20
40
60
80
100
BMD

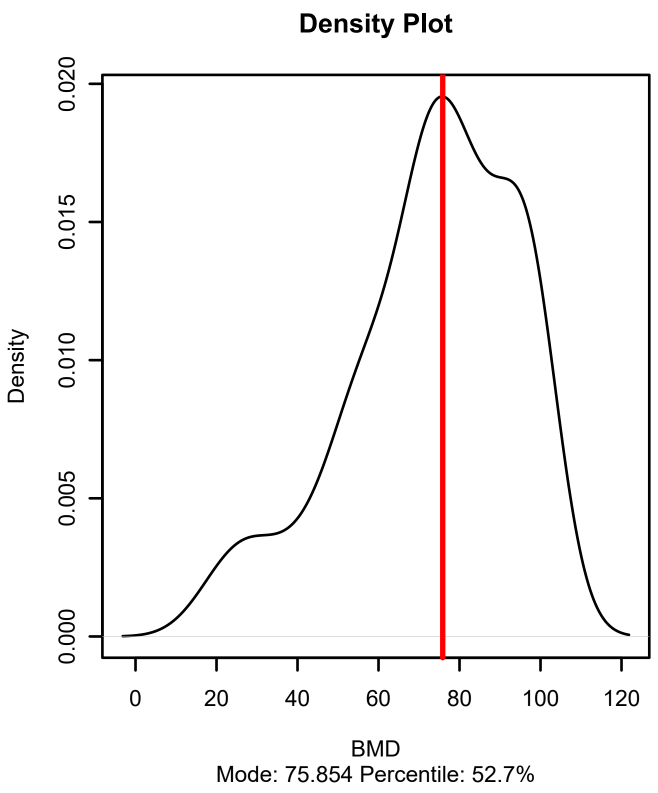
Density Plot
Density
0.000
0.005
0.010
0.015
0.020
0
20
40
60
80
100
120
BMD
Mode: 75.854 Percentile: 52.7%

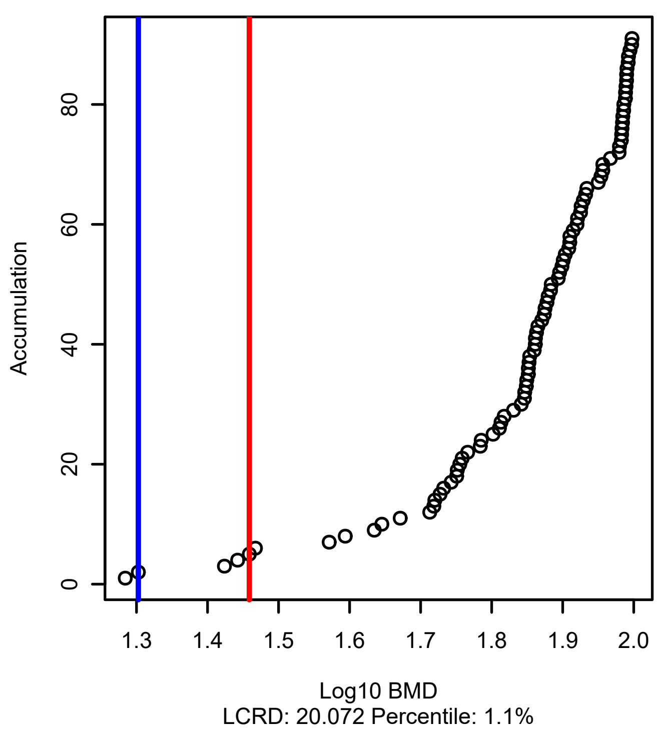
Accumulation
0
20
40
60
80
1.3
1.4
1.5
1.6
1.7
1.8
1.9
2.0
Log10 BMD
LCRD: 20.072 Percentile: 1.1%

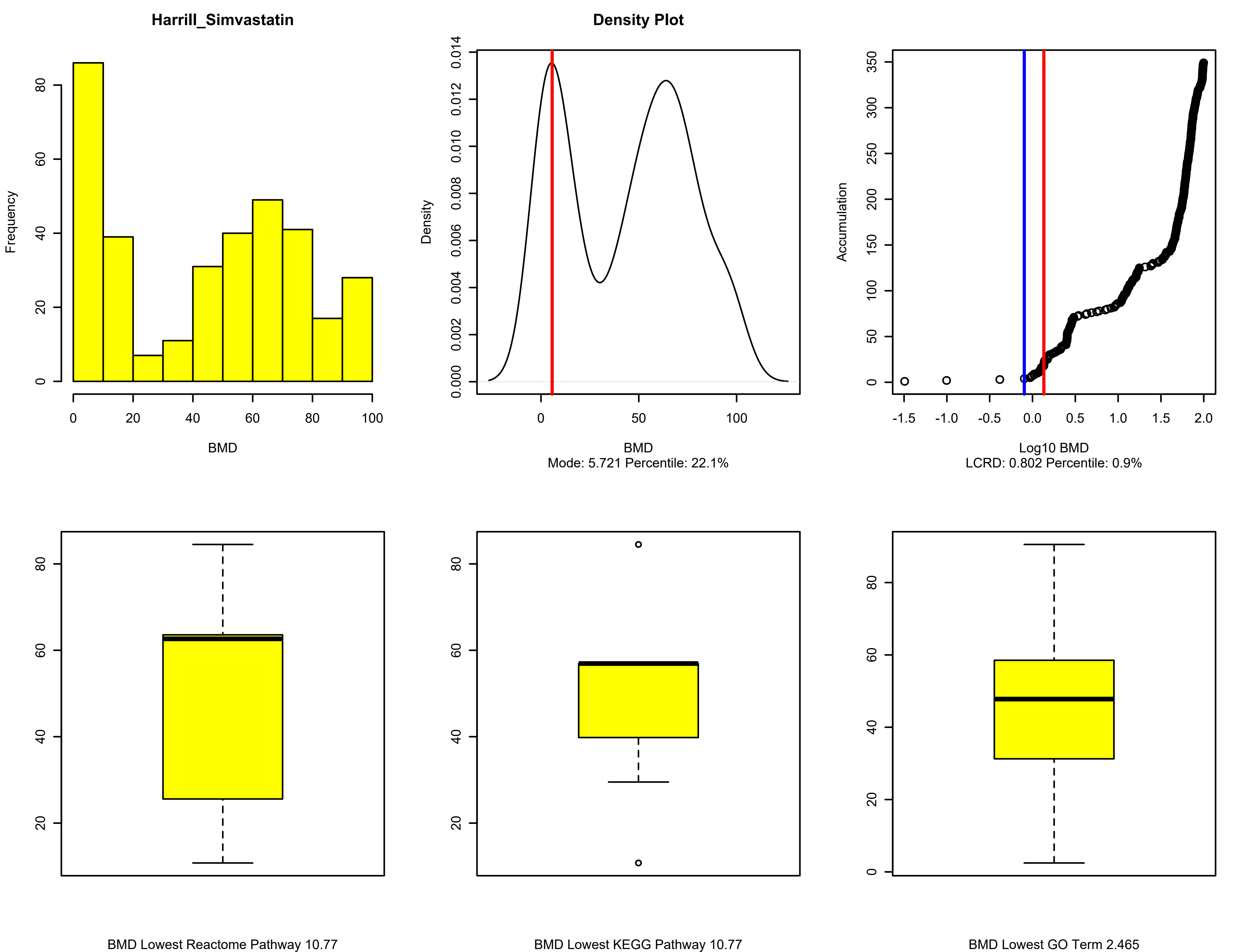

Harrill_Simvastatin
Frequency
BMD
Density Plot
Density
BMD
Mode: 5.721 Percentile: 22.1%
Accumulation
Log10 BMD
LCRD: 0.802 Percentile: 0.9%
BMD Lowest Reactome Pathway 10.77
BMD Lowest KEGG Pathway 10.77
BMD Lowest GO Term 2.465

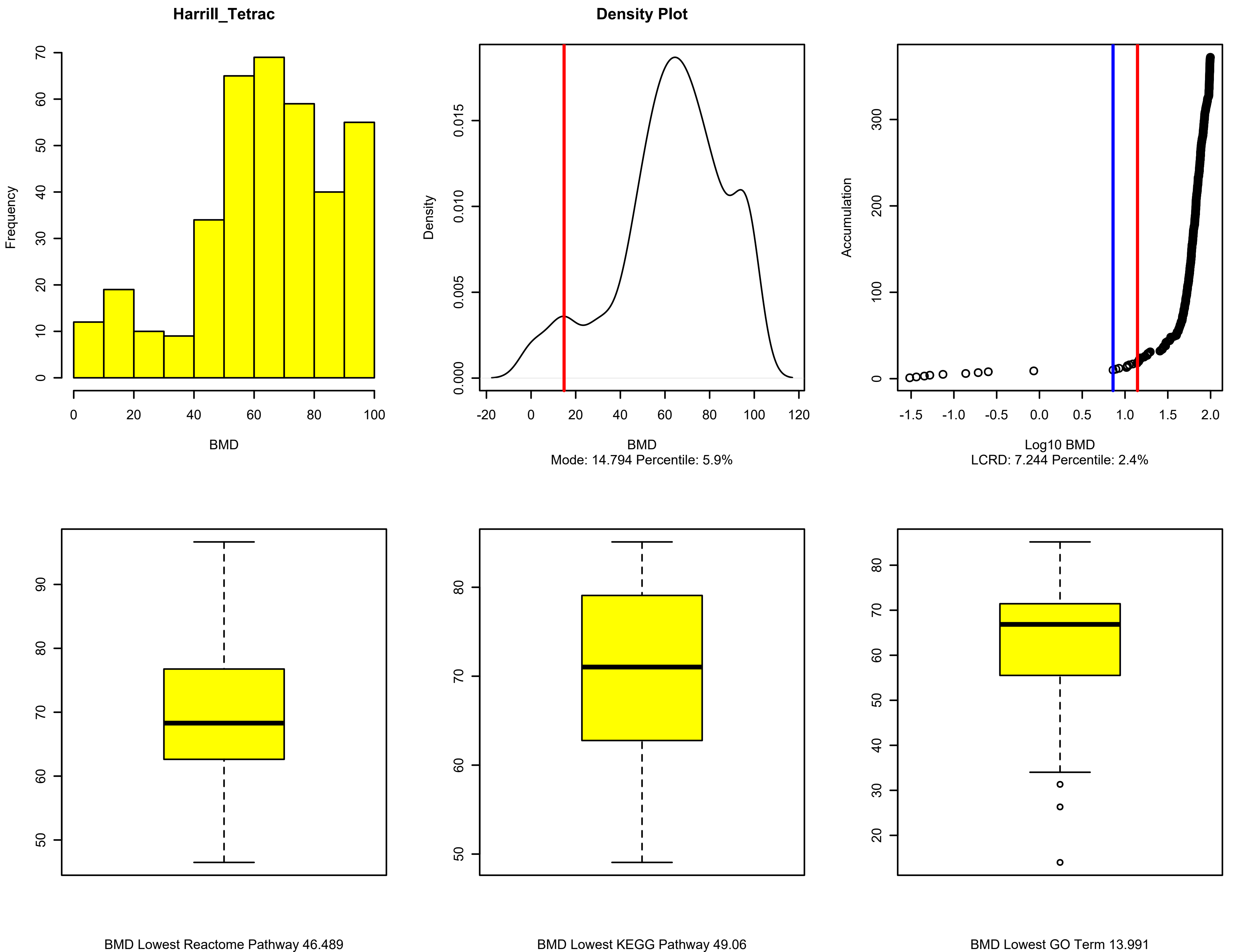
Harrill_Tetrac
Frequency
BMD
Density Plot
Density
BMD
Mode: 14.794 Percentile: 5.9%
Accumulation
Log10 BMD
LCRD: 7.244 Percentile: 2.4%
BMD Lowest Reactome Pathway 46.489
BMD Lowest KEGG Pathway 49.06
BMD Lowest GO Term 13.991

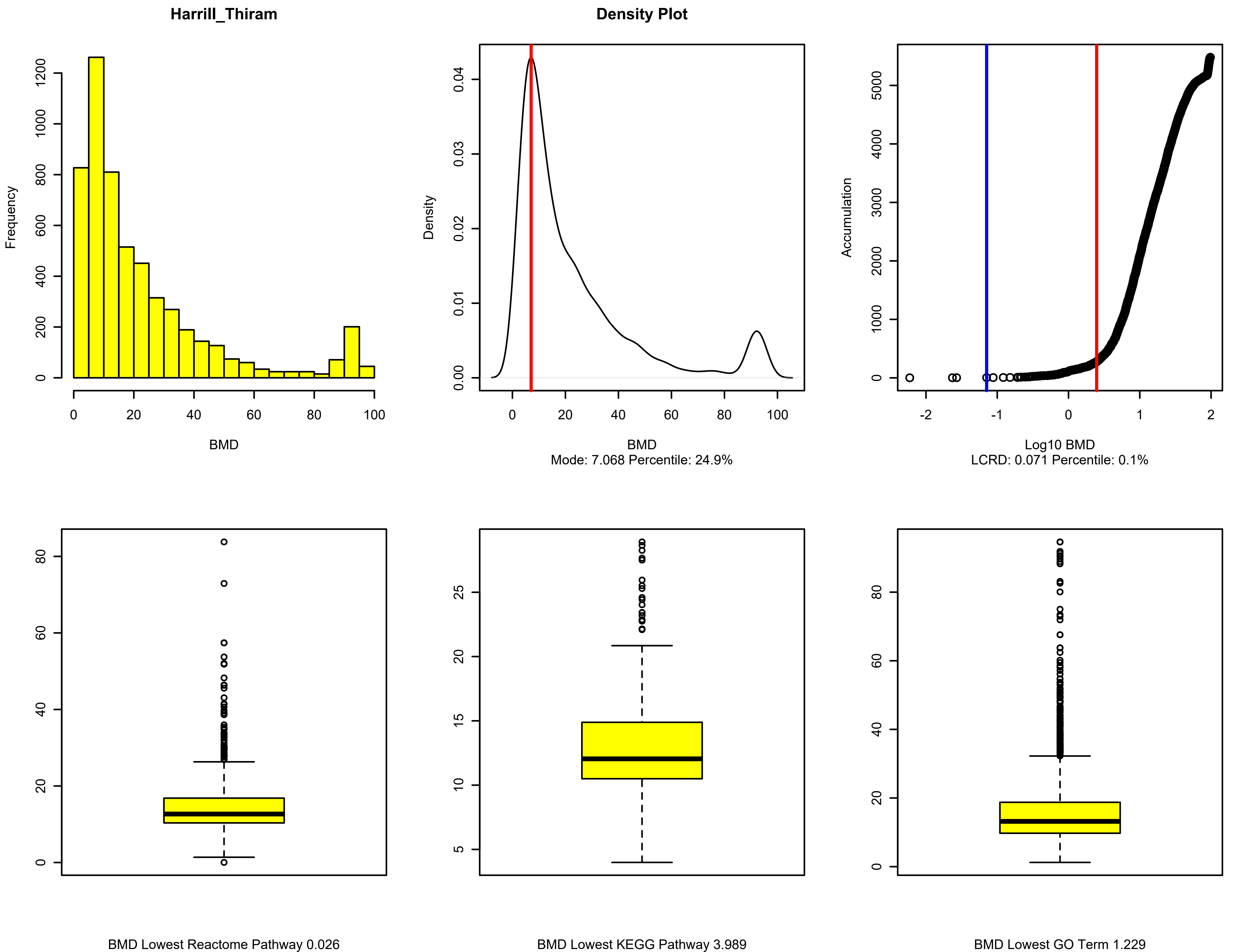
Harrill_Thiram
Frequency
BMD
Density Plot
Density
BMD
Mode: 7.068 Percentile: 24.9%
Accumulation
Log10 BMD
LCRD: 0.071 Percentile: 0.1%
BMD Lowest Reactome Pathway 0.026
BMD Lowest KEGG Pathway 3.989
BMD Lowest GO Term 1.229

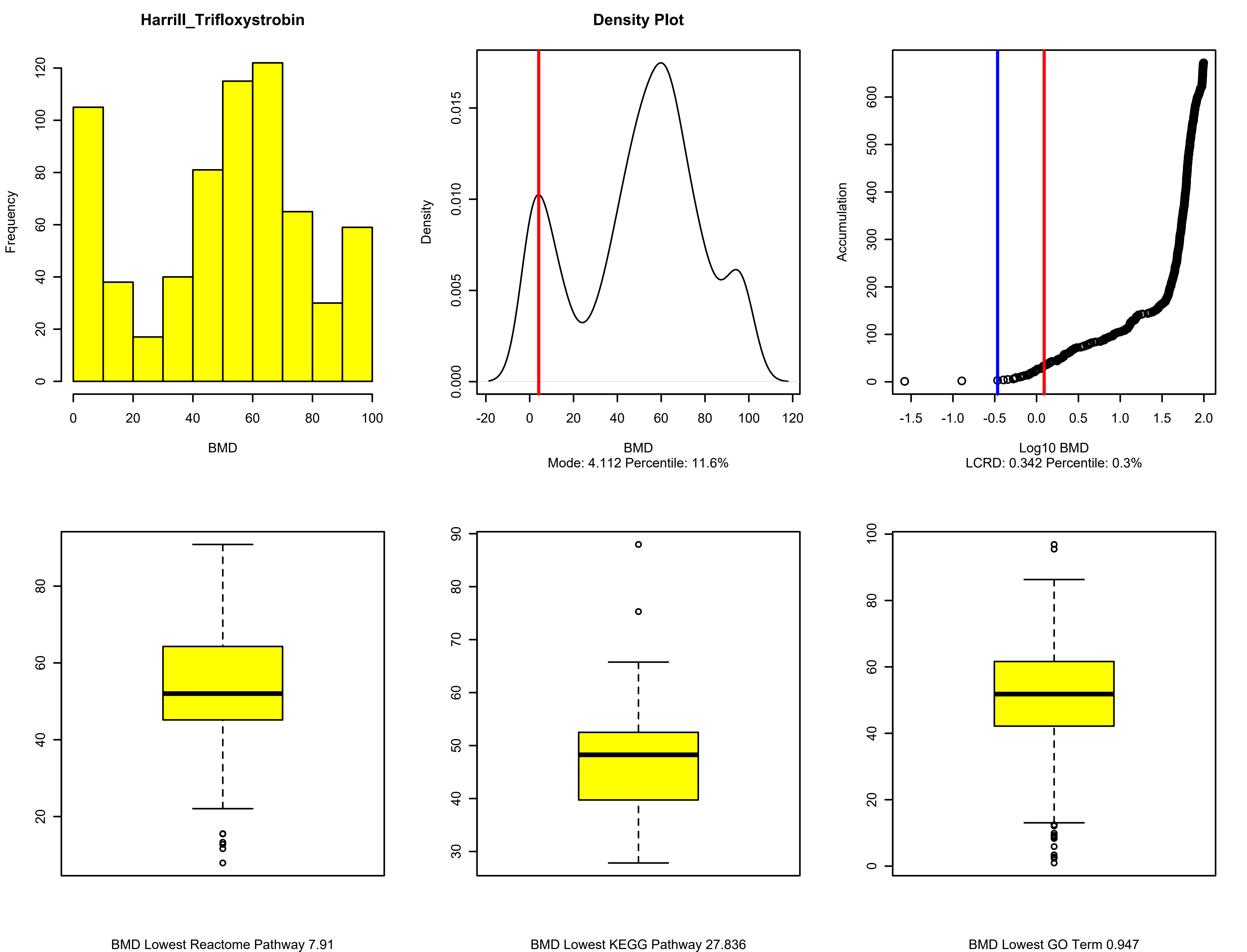
Harrill_Trifloxystrobin
Frequency
BMD
Density Plot
Density
BMD
Mode: 4.112 Percentile: 11.6%
Accumulation
Log10 BMD
LCRD: 0.342 Percentile: 0.3%
BMD Lowest Reactome Pathway 7.91
BMD Lowest KEGG Pathway 27.836
BMD Lowest GO Term 0.947

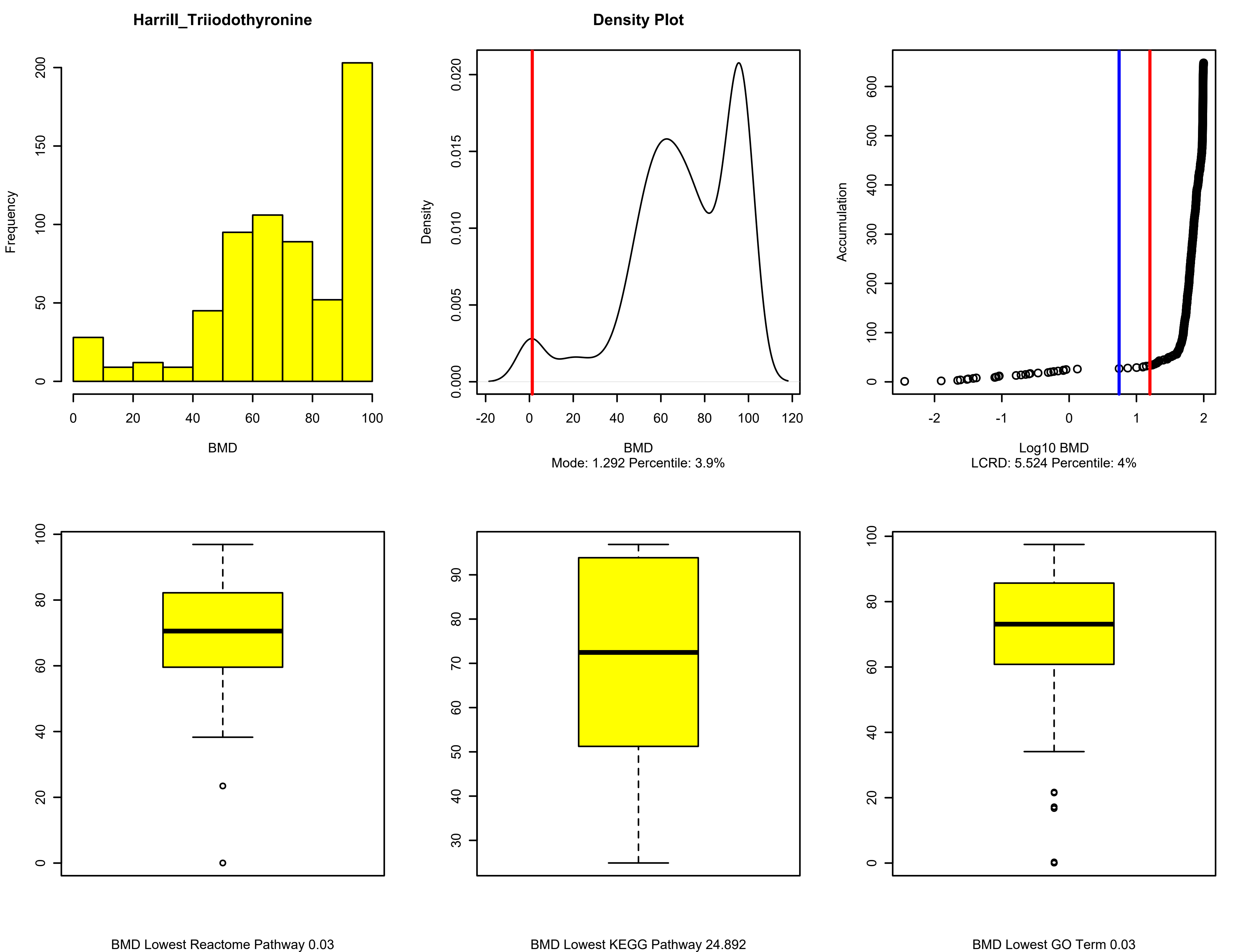
Harrill_Triiodothyronine
Frequency
BMD
Density Plot
Density
BMD
Mode: 1.292 Percentile: 3.9%
Accumulation
Log10 BMD
LCRD: 5.524 Percentile: 4%
BMD Lowest Reactome Pathway 0.03
BMD Lowest KEGG Pathway 24.892
BMD Lowest GO Term 0.03

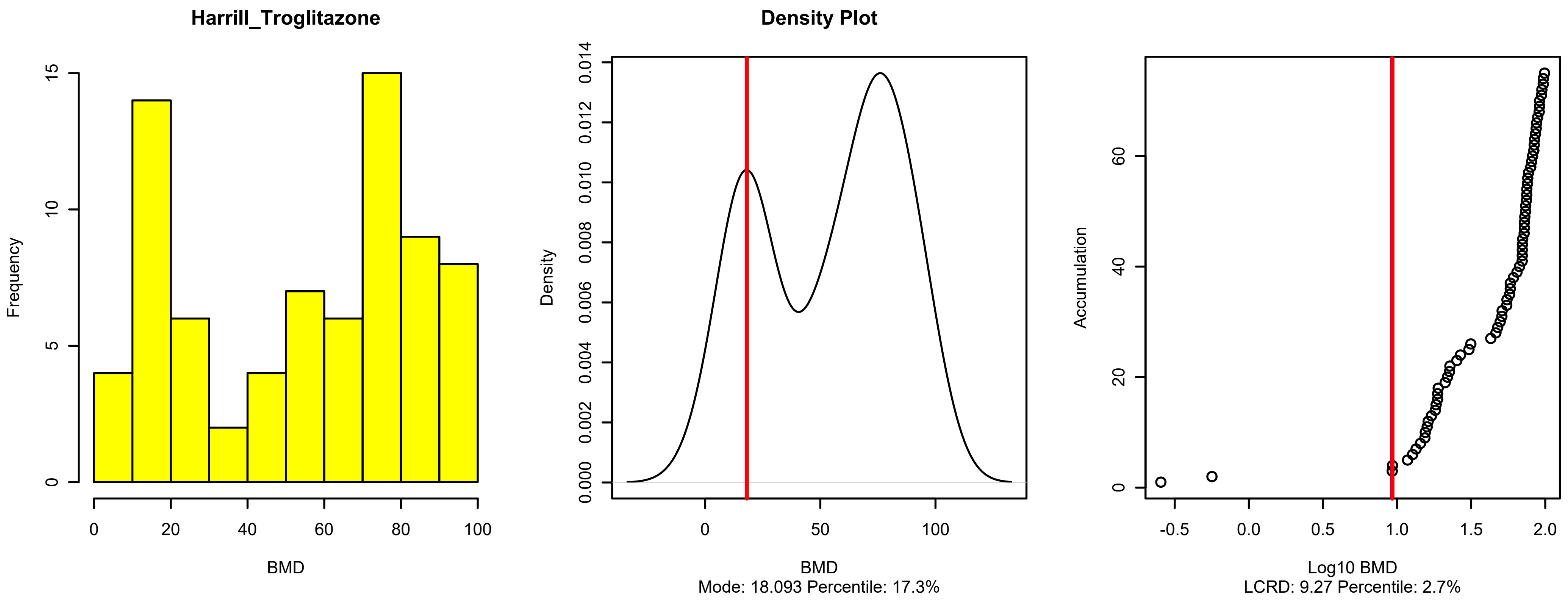
Harrill_Troglitazone
Frequency
BMD
Density Plot
Density
BMD
Mode: 18.093 Percentile: 17.3%
Accumulation
Log10 BMD
LCRD: 9.27 Percentile: 2.7%

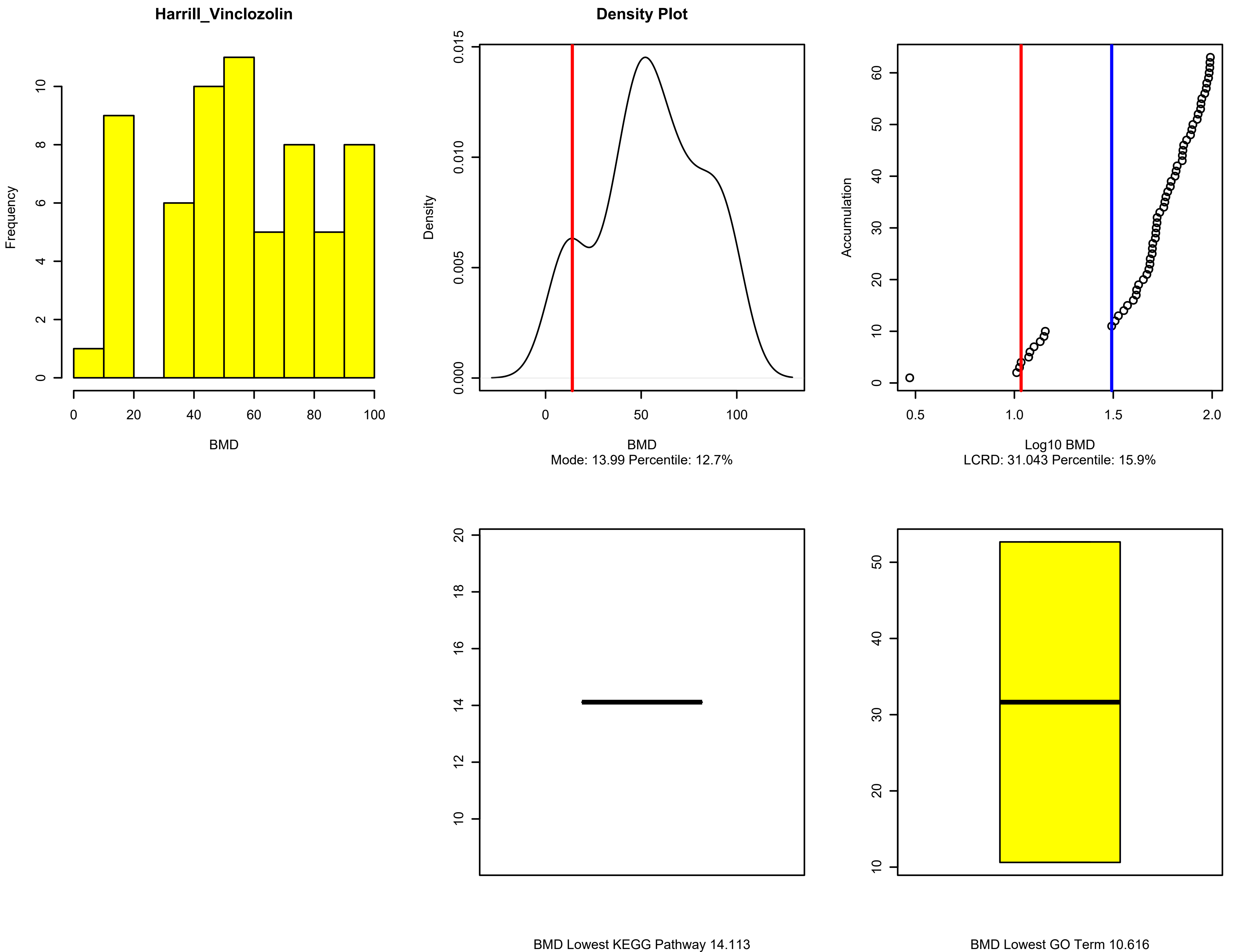
Harrill_Vinclozolin
Frequency
BMD
Density Plot
Density
BMD
Mode: 13.99 Percentile: 12.7%
Accumulation
Log10 BMD
LCRD: 31.043 Percentile: 15.9%
BMD Lowest KEGG Pathway 14.113
BMD Lowest GO Term 10.616

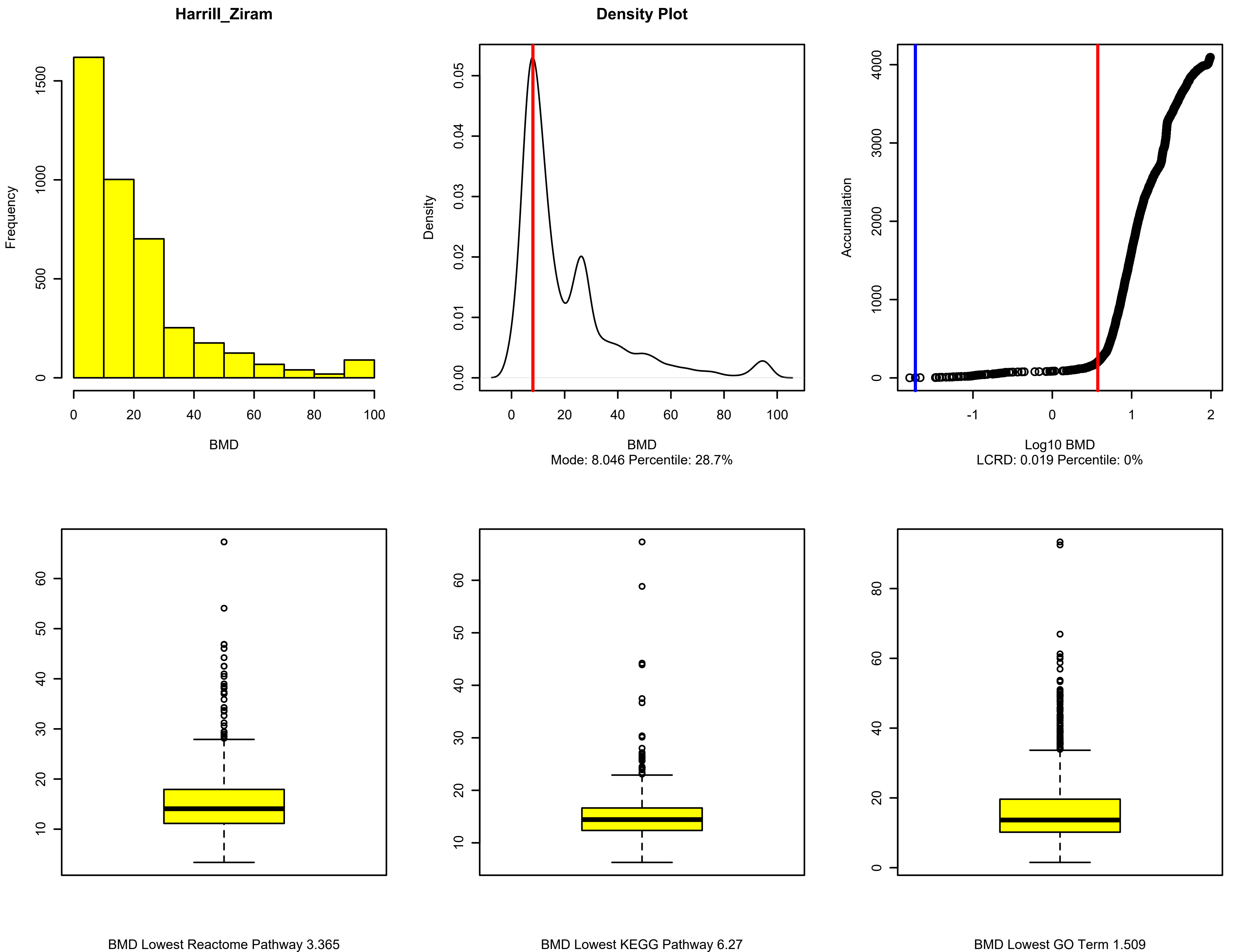
Harrill_Ziram
Frequency
BMD
Density Plot
Density
BMD
Mode: 8.046 Percentile: 28.7%
Accumulation
Log10 BMD
LCRD: 0.019 Percentile: 0%
BMD Lowest Reactome Pathway 3.365
BMD Lowest KEGG Pathway 6.27
BMD Lowest GO Term 1.509

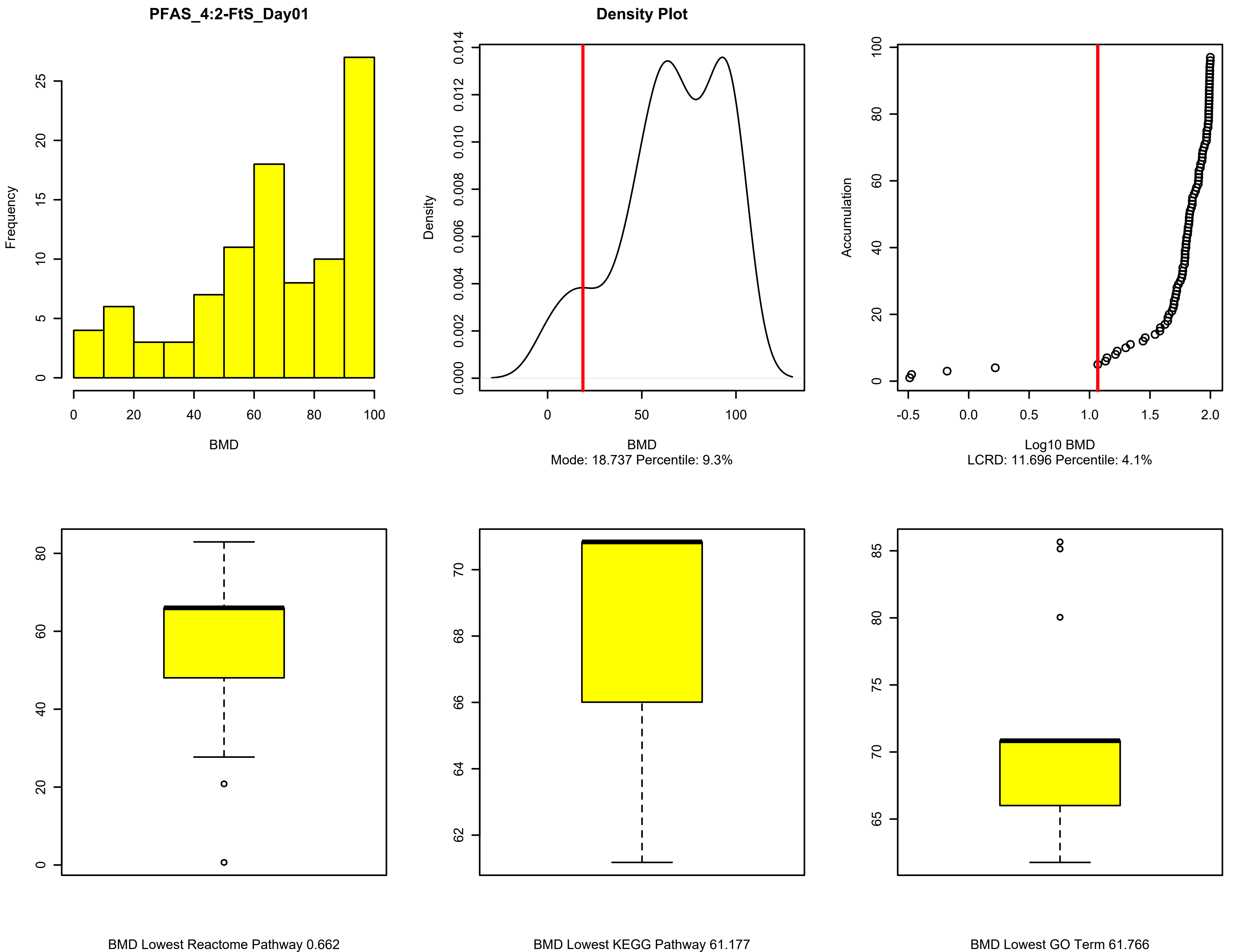
PFAS_4:2-FtS_Day01
Frequency
BMD
Density Plot
Density
BMD
Mode: 18.737 Percentile: 9.3%
Accumulation
Log10 BMD
LCRD: 11.696 Percentile: 4.1%
BMD Lowest Reactome Pathway 0.662
BMD Lowest KEGG Pathway 61.177
BMD Lowest GO Term 61.766

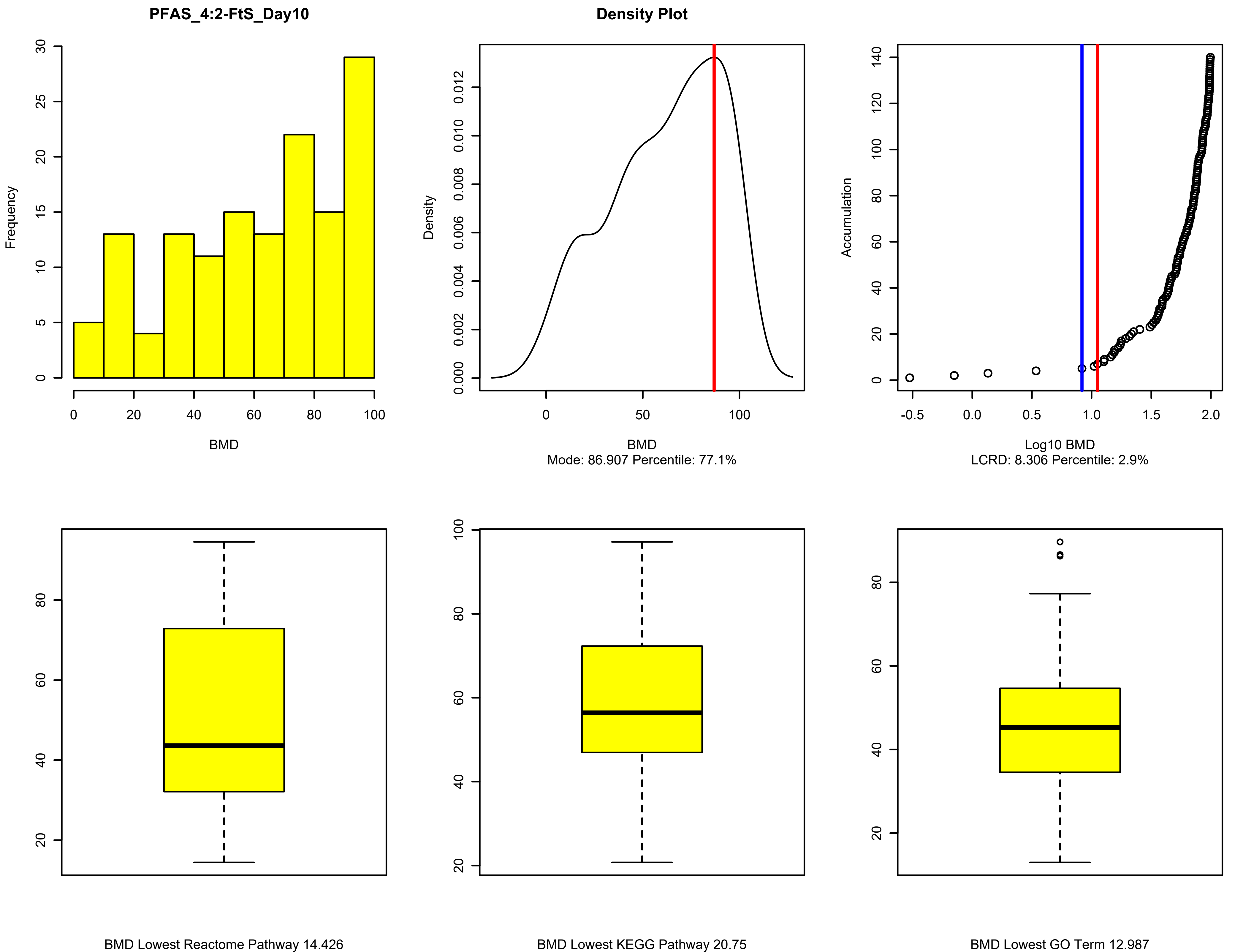
PFAS_4:2-FtS_Day10
Frequency
BMD
Density Plot
Density
BMD
Mode: 86.907 Percentile: 77.1%
Accumulation
Log10 BMD
LCRD: 8.306 Percentile: 2.9%
BMD Lowest Reactome Pathway 14.426
BMD Lowest KEGG Pathway 20.75
BMD Lowest GO Term 12.987

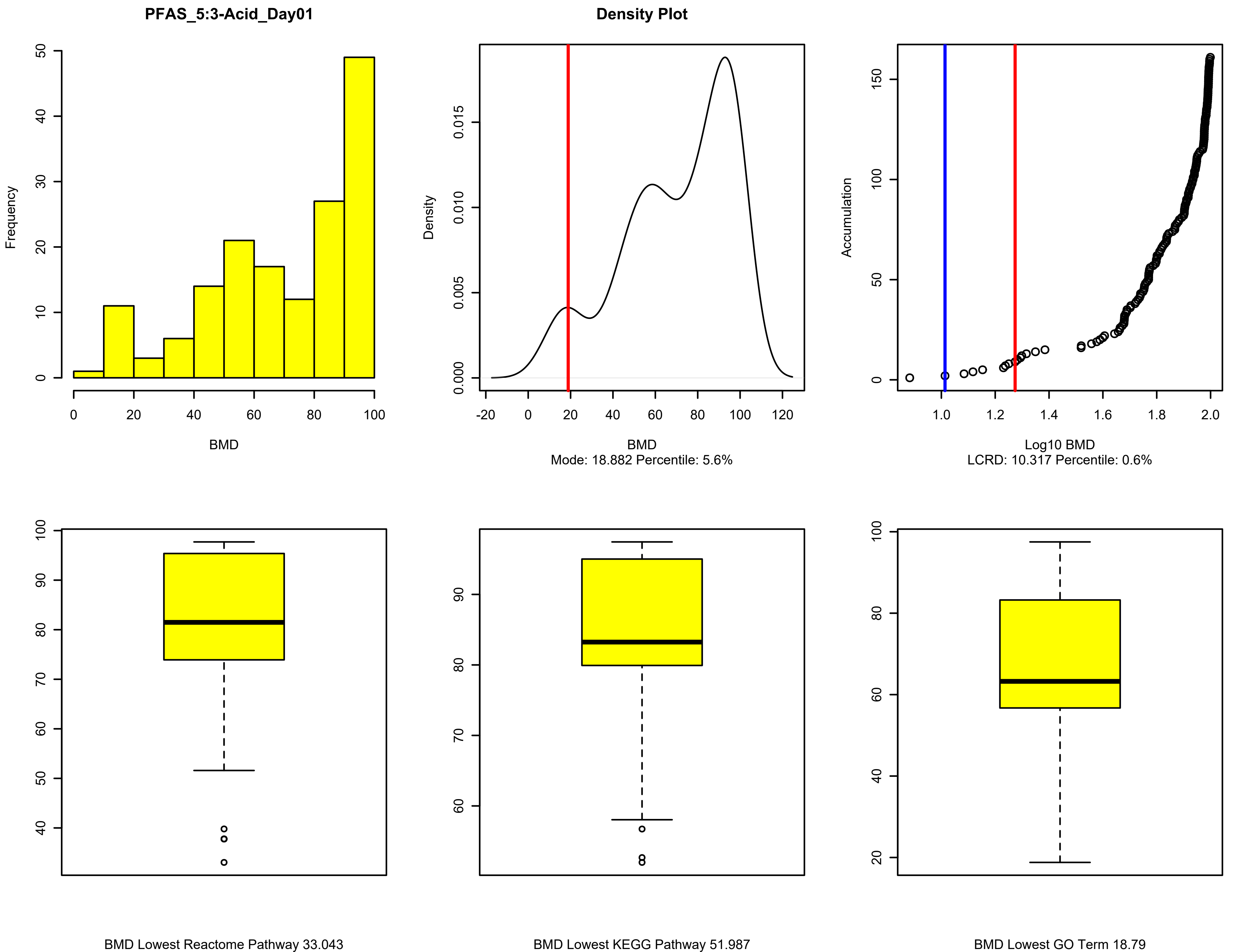

PFAS_5:3-Acid_Day01
Frequency
BMD
Density Plot
Density
BMD
Mode: 18.882 Percentile: 5.6%
Accumulation
Log10 BMD
LCRD: 10.317 Percentile: 0.6%
BMD Lowest Reactome Pathway 33.043
BMD Lowest KEGG Pathway 51.987
BMD Lowest GO Term 18.79

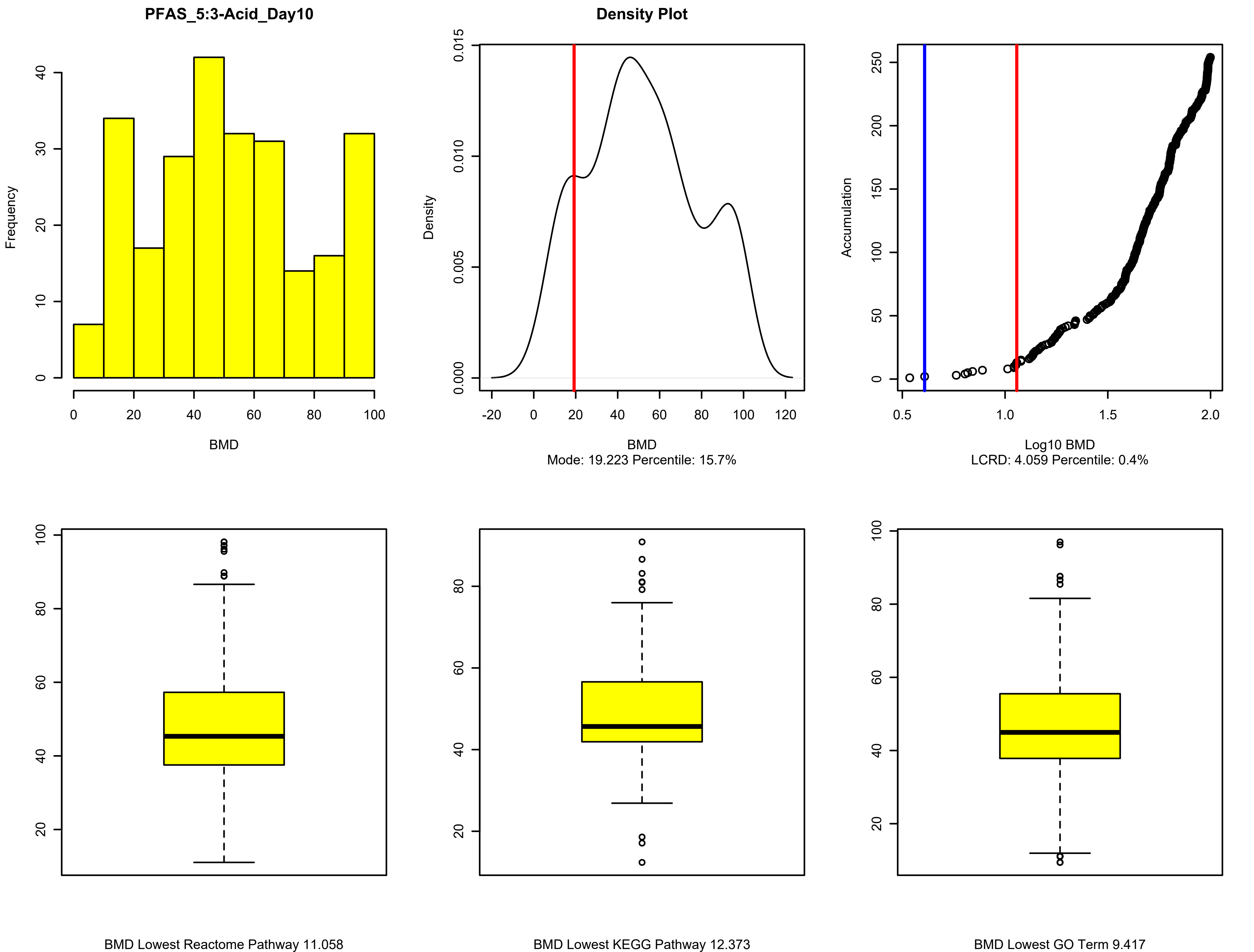

PFAS_5:3-Acid_Day10
Frequency
BMD
Density Plot
Density
BMD
Mode: 19.223 Percentile: 15.7%
Accumulation
Log10 BMD
LCRD: 4.059 Percentile: 0.4%
BMD Lowest Reactome Pathway 11.058
BMD Lowest KEGG Pathway 12.373
BMD Lowest GO Term 9.417

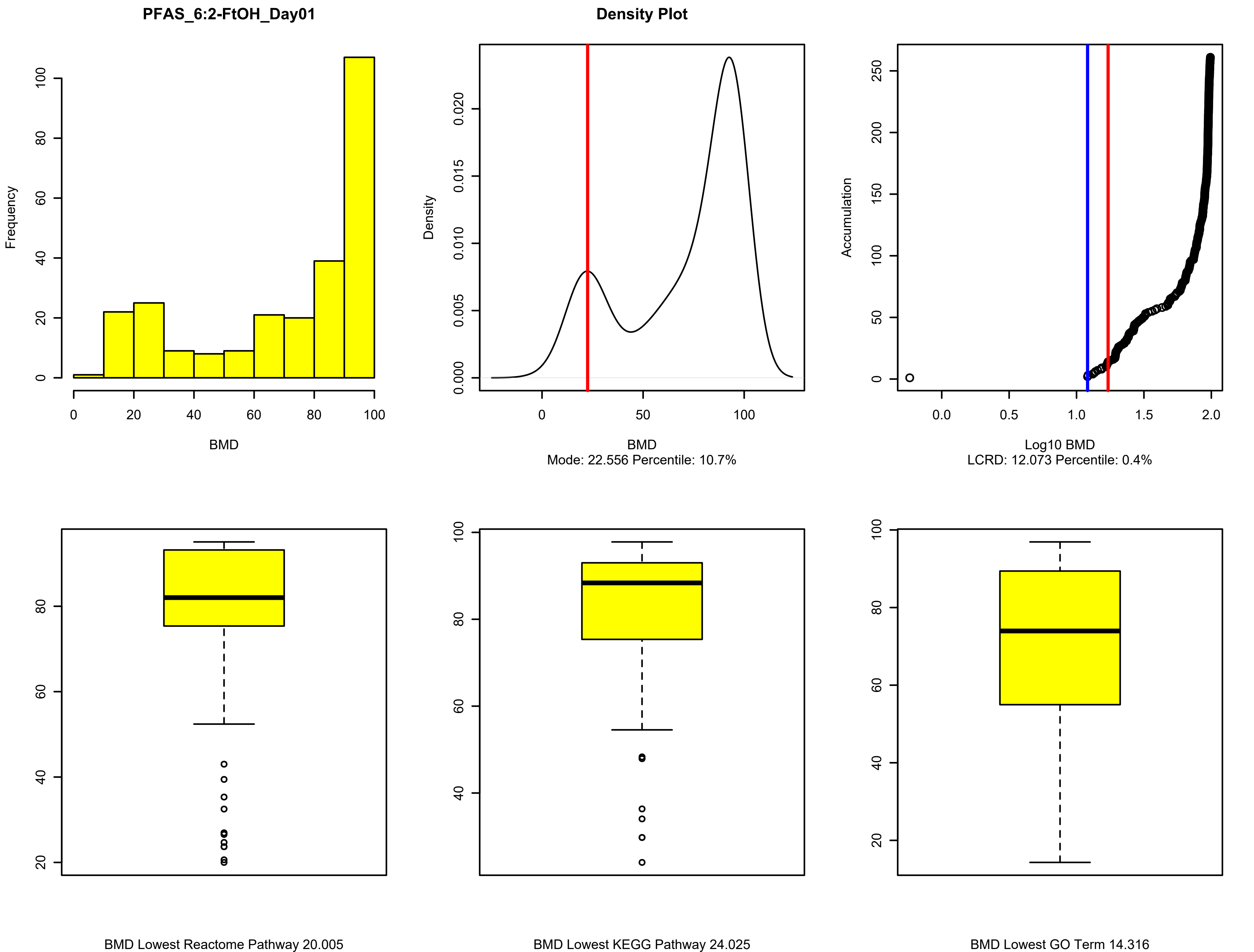
PFAS_6:2-FtOH_Day01
Frequency
BMD
Density Plot
Density
BMD
Mode: 22.556 Percentile: 10.7%
Accumulation
Log10 BMD
LCRD: 12.073 Percentile: 0.4%
BMD Lowest Reactome Pathway 20.005
BMD Lowest KEGG Pathway 24.025
BMD Lowest GO Term 14.316

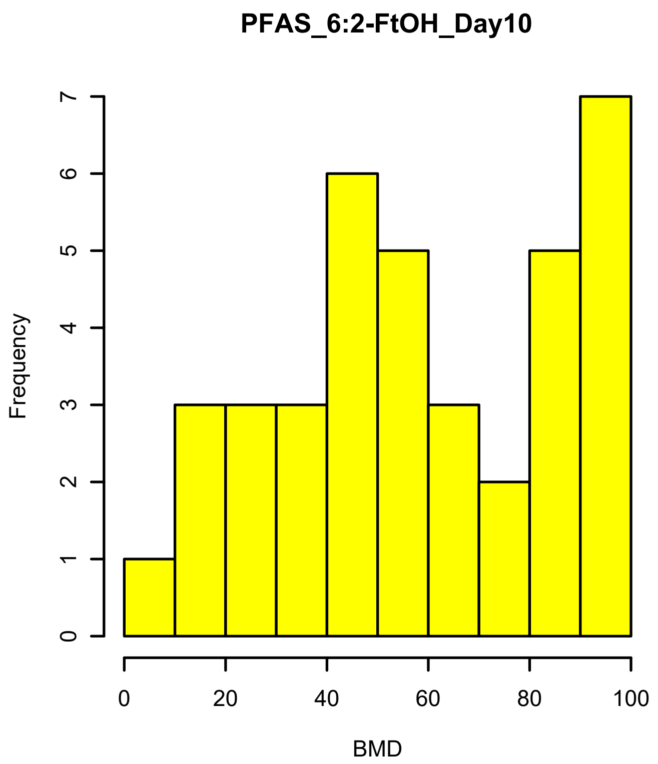

PFAS_6:2-FtOH_Day10
Frequency
BMD

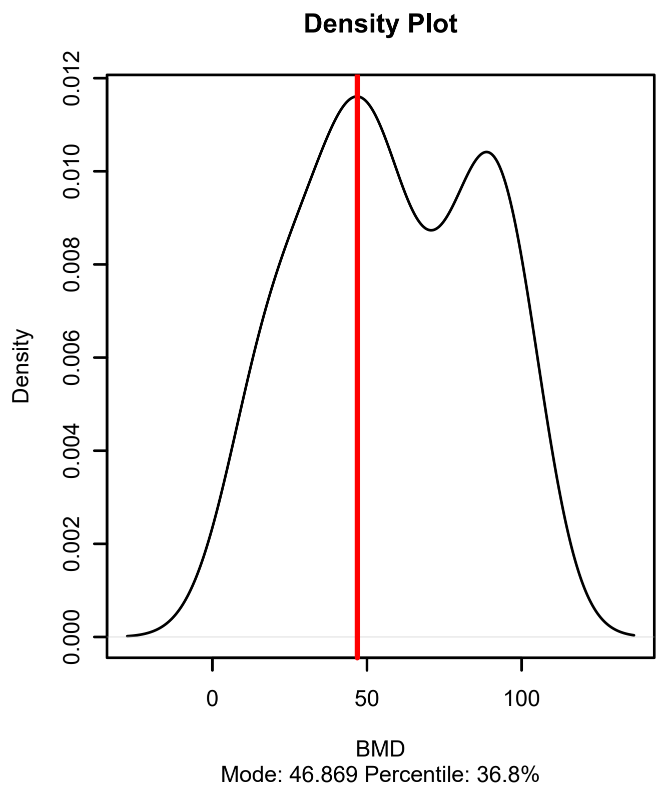

Density Plot
Density
BMD
Mode: 46.869 Percentile: 36.8%

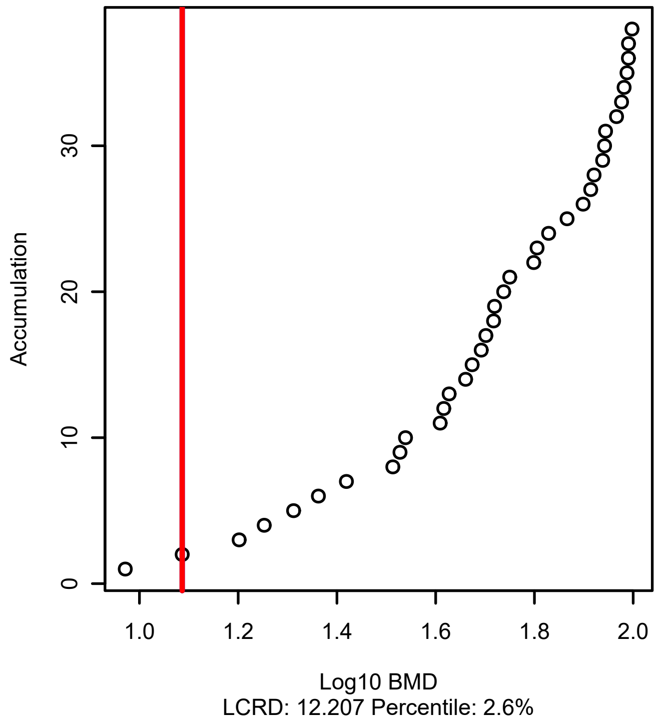

Accumulation
Log10 BMD
LCRD: 12.207 Percentile: 2.6%

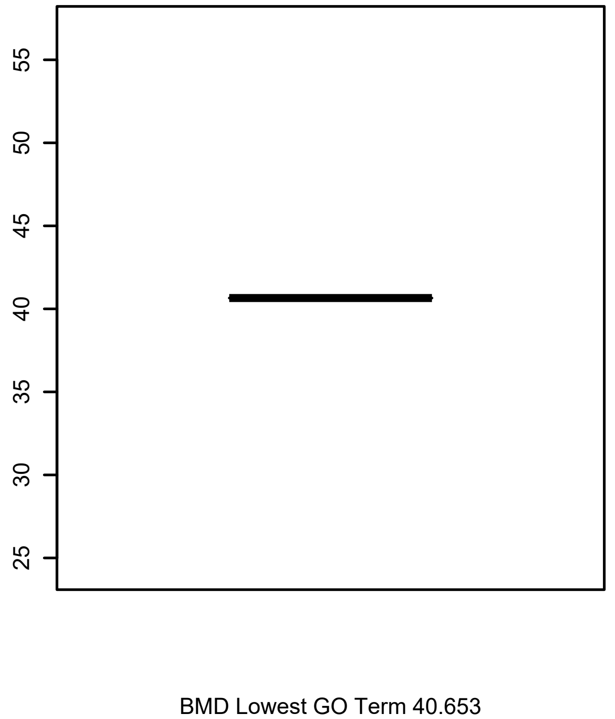

BMD Lowest GO Term 40.653

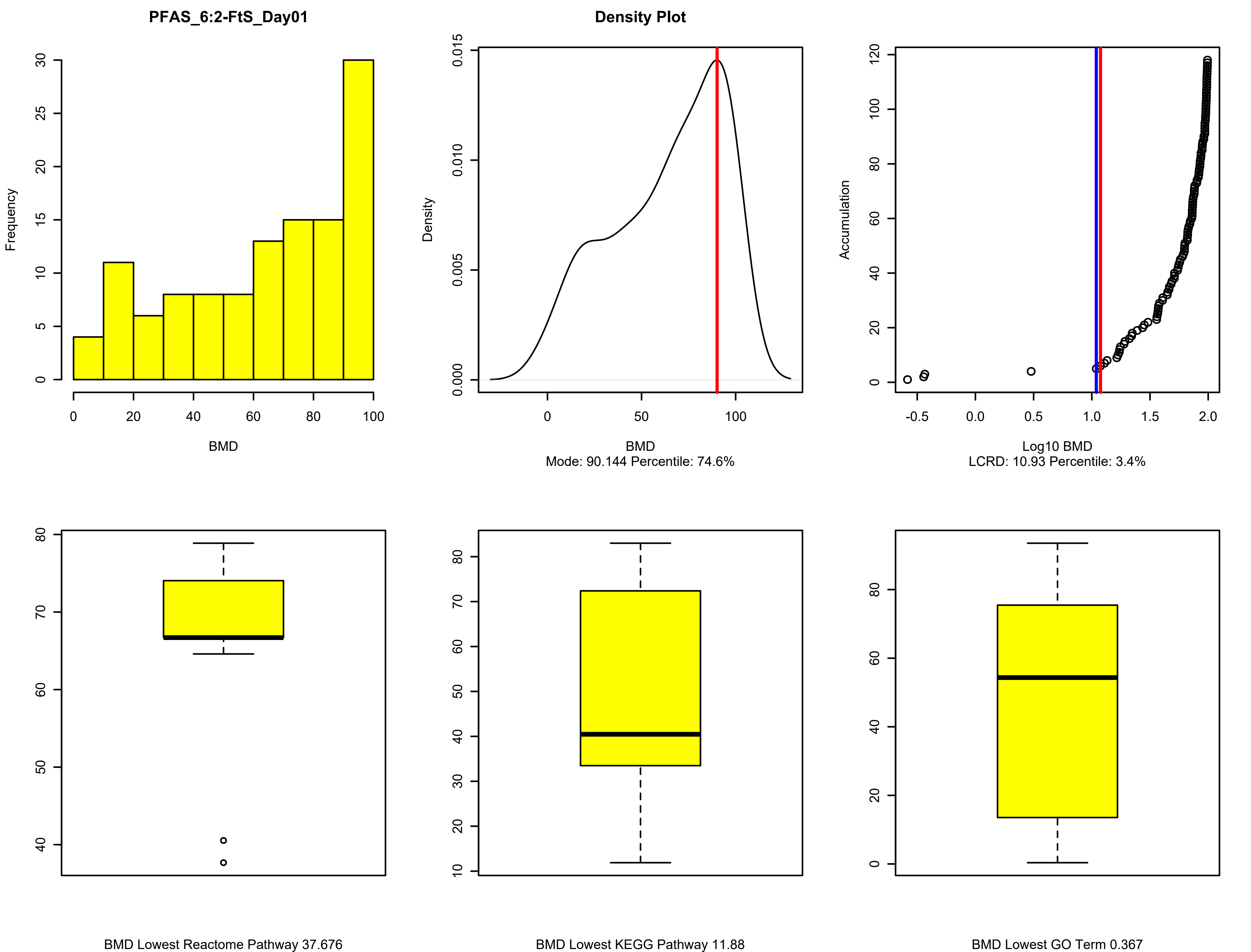
PFAS_6:2-FtS_Day01
Frequency
BMD
Density Plot
Density
BMD
Mode: 90.144 Percentile: 74.6%
Accumulation
Log10 BMD
LCRD: 10.93 Percentile: 3.4%
BMD Lowest Reactome Pathway 37.676
BMD Lowest KEGG Pathway 11.88
BMD Lowest GO Term 0.367

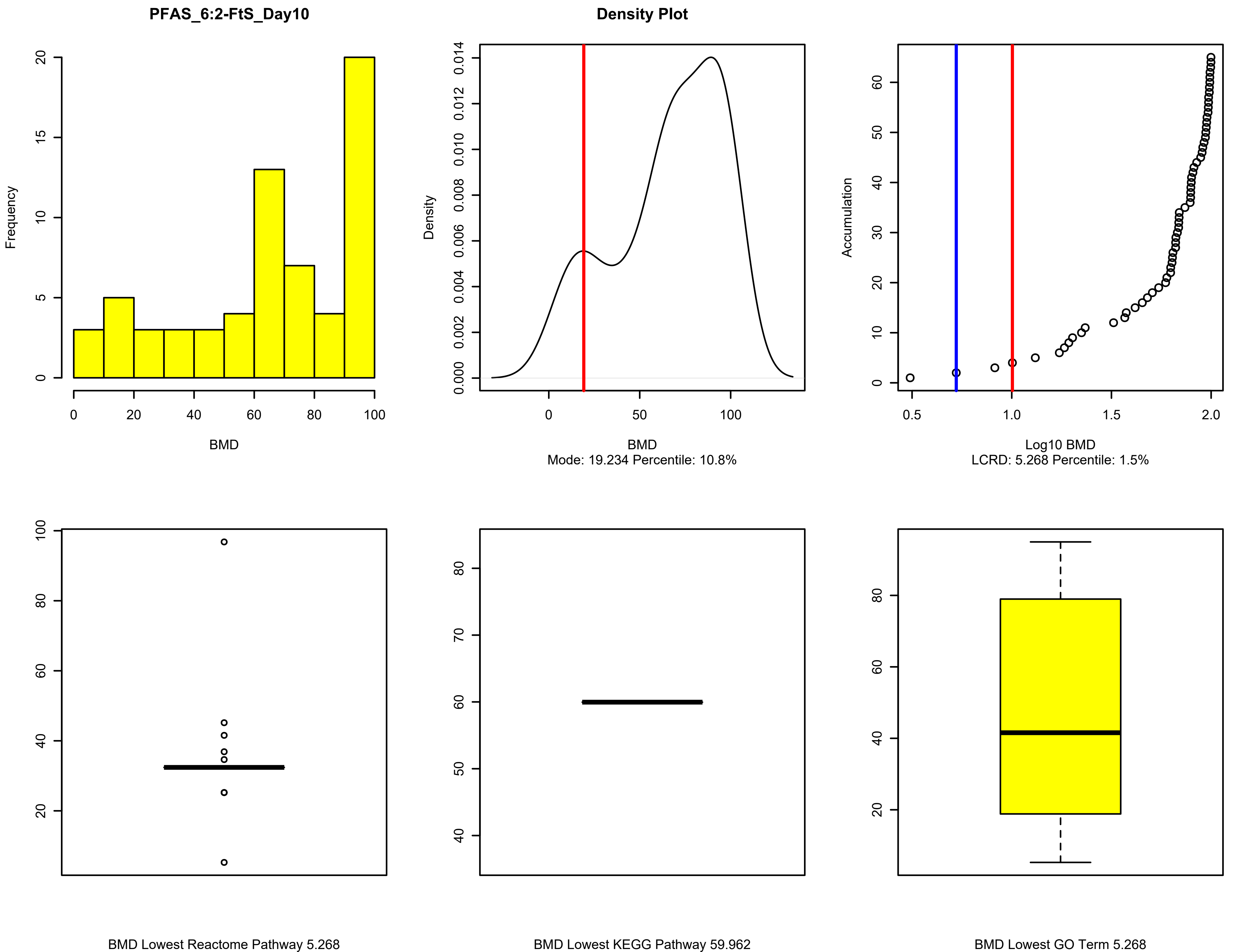
PFAS_6:2-FtS_Day10
Frequency
BMD
Density Plot
Density
BMD
Mode: 19.234 Percentile: 10.8%
Accumulation
Log10 BMD
LCRD: 5.268 Percentile: 1.5%
BMD Lowest Reactome Pathway 5.268
BMD Lowest KEGG Pathway 59.962
BMD Lowest GO Term 5.268

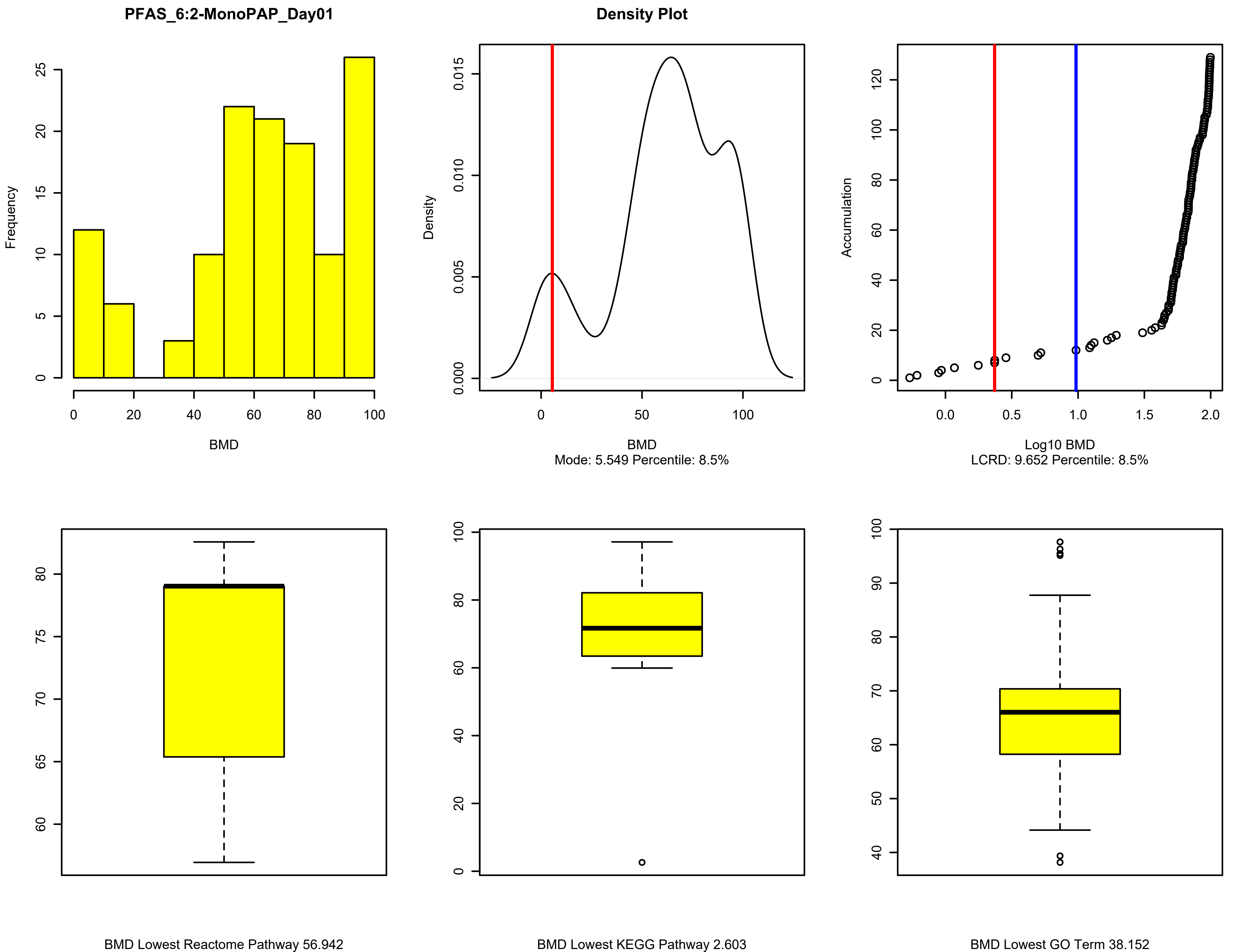
PFAS_6:2-MonoPAP_Day01
Frequency
BMD
Density Plot
Density
BMD
Mode: 5.549 Percentile: 8.5%
Accumulation
Log10 BMD
LCRD: 9.652 Percentile: 8.5%
BMD Lowest Reactome Pathway 56.942
BMD Lowest KEGG Pathway 2.603
BMD Lowest GO Term 38.152

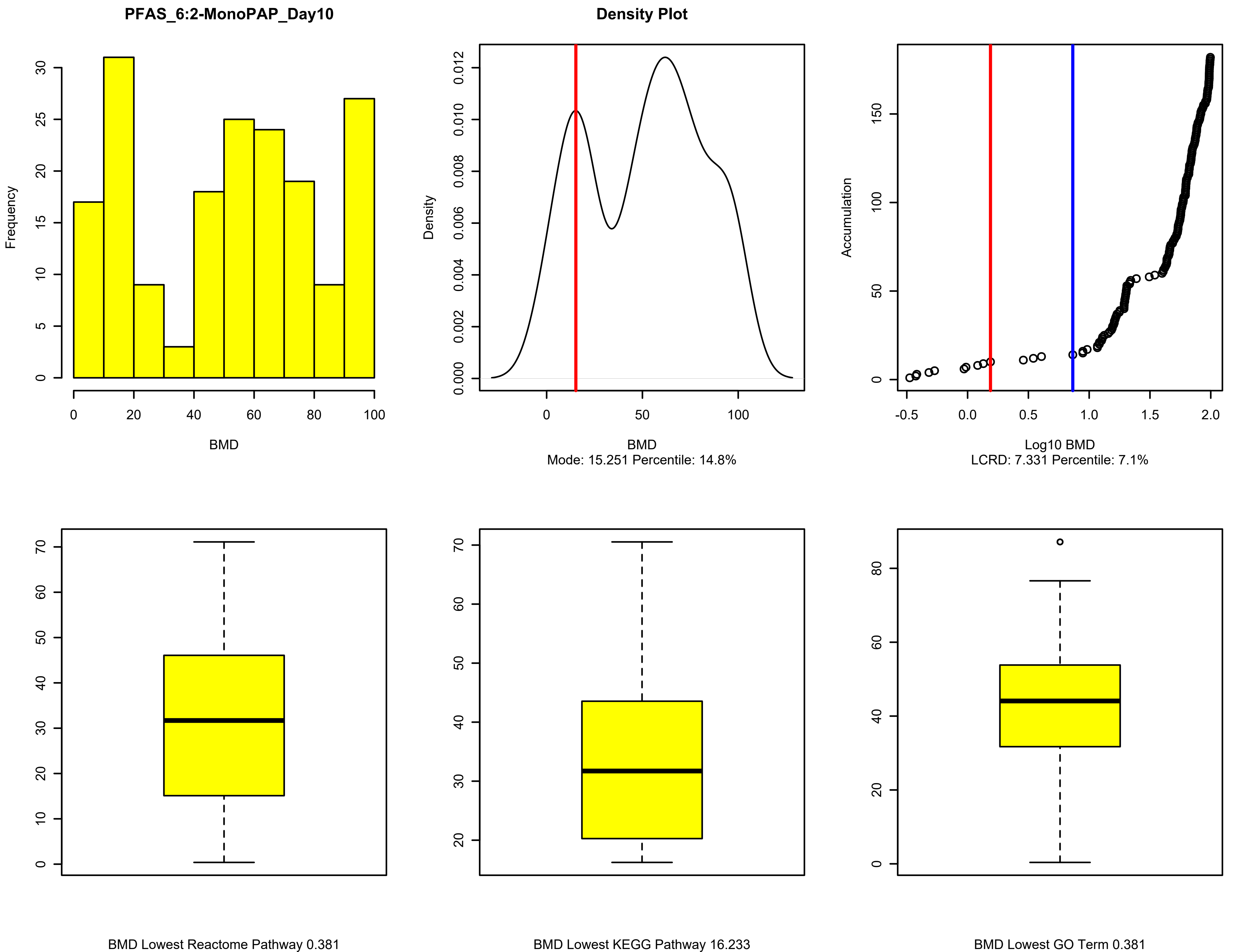

PFAS_6:2-MonoPAP_Day10
Frequency
BMD
Density Plot
Density
BMD
Mode: 15.251 Percentile: 14.8%
Accumulation
Log10 BMD
LCRD: 7.331 Percentile: 7.1%
BMD Lowest Reactome Pathway 0.381
BMD Lowest KEGG Pathway 16.233
BMD Lowest GO Term 0.381

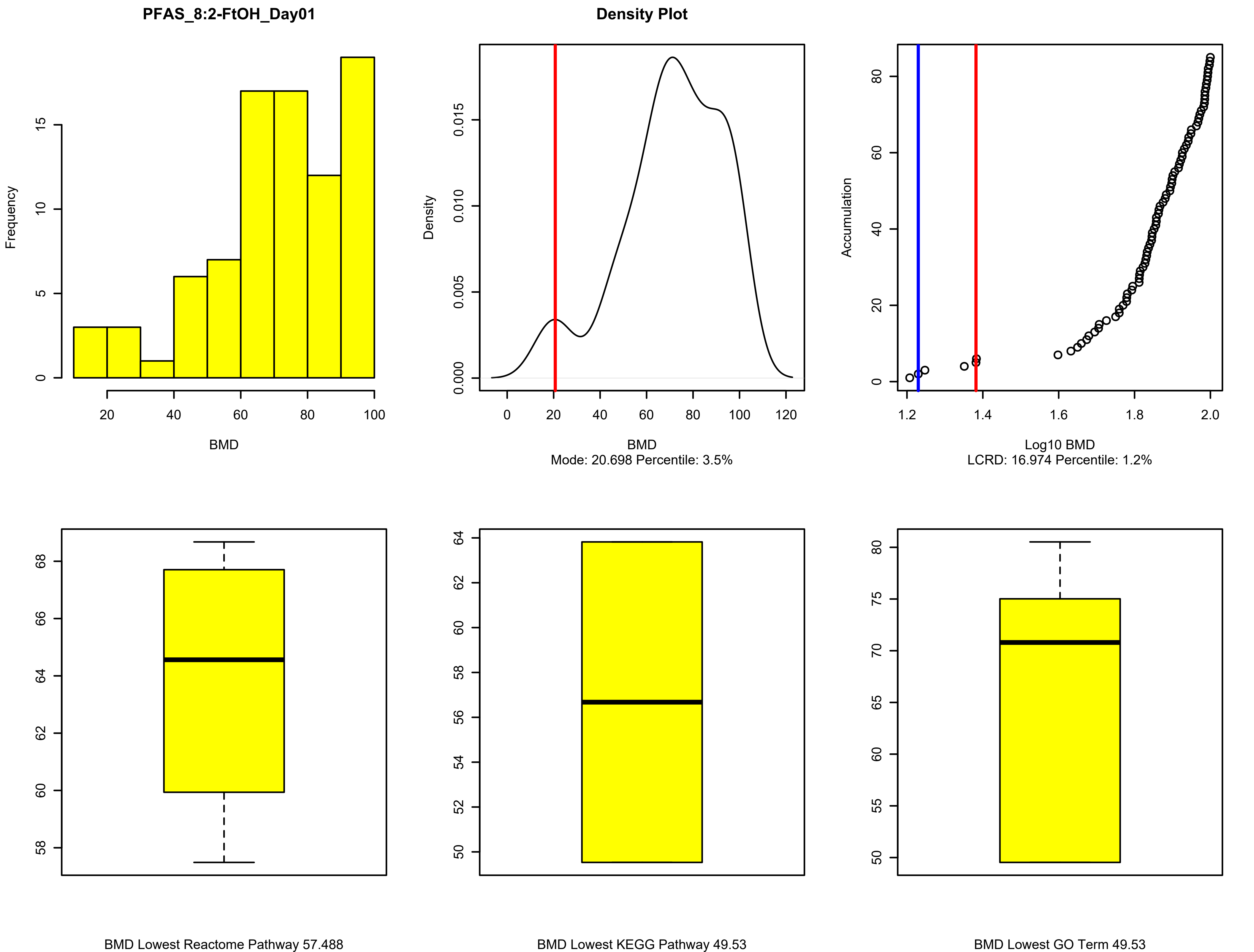

PFAS_8:2-FtOH_Day01
Frequency
BMD
Density Plot
Density
BMD
Mode: 20.698 Percentile: 3.5%
Accumulation
Log10 BMD
LCRD: 16.974 Percentile: 1.2%
BMD Lowest Reactome Pathway 57.488
BMD Lowest KEGG Pathway 49.53
BMD Lowest GO Term 49.53

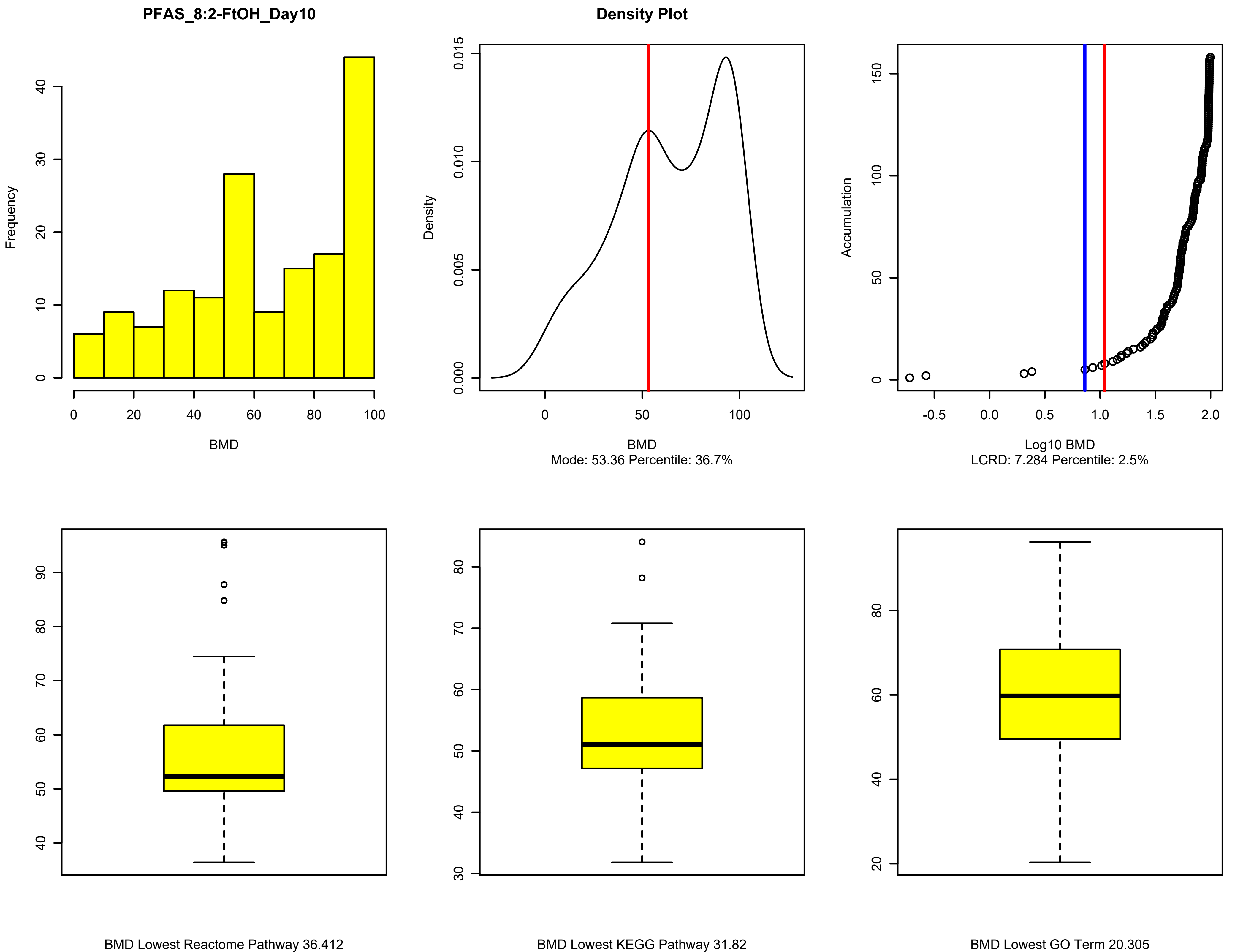
PFAS_8:2-FtOH_Day10
Frequency
BMD
Density Plot
Density
BMD
Mode: 53.36 Percentile: 36.7%
Accumulation
Log10 BMD
LCRD: 7.284 Percentile: 2.5%
BMD Lowest Reactome Pathway 36.412
BMD Lowest KEGG Pathway 31.82
BMD Lowest GO Term 20.305

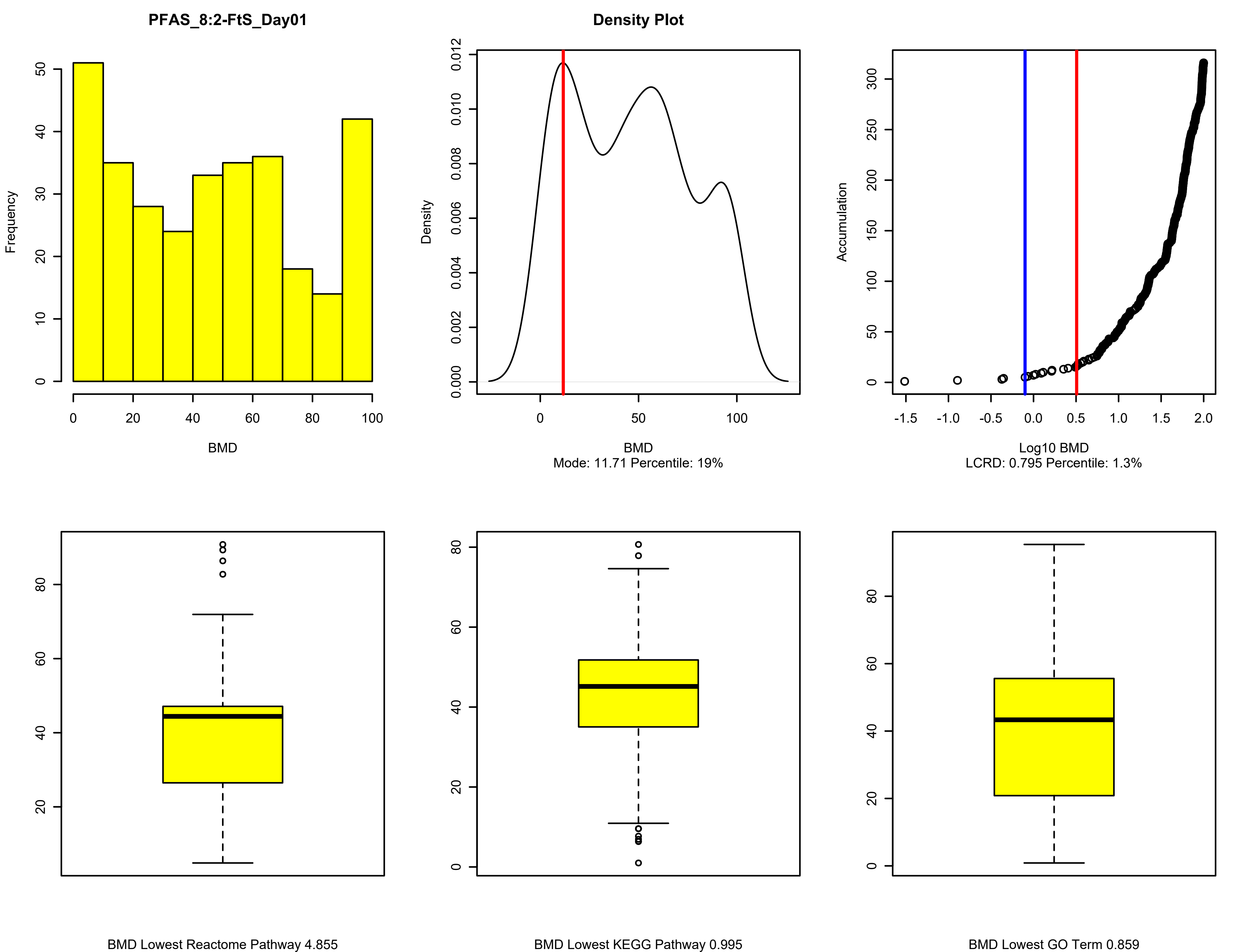

PFAS_8:2-FtS_Day01
Frequency
BMD
Density Plot
Density
BMD
Mode: 11.71 Percentile: 19%
Accumulation
Log10 BMD
LCRD: 0.795 Percentile: 1.3%
BMD Lowest Reactome Pathway 4.855
BMD Lowest KEGG Pathway 0.995
BMD Lowest GO Term 0.859

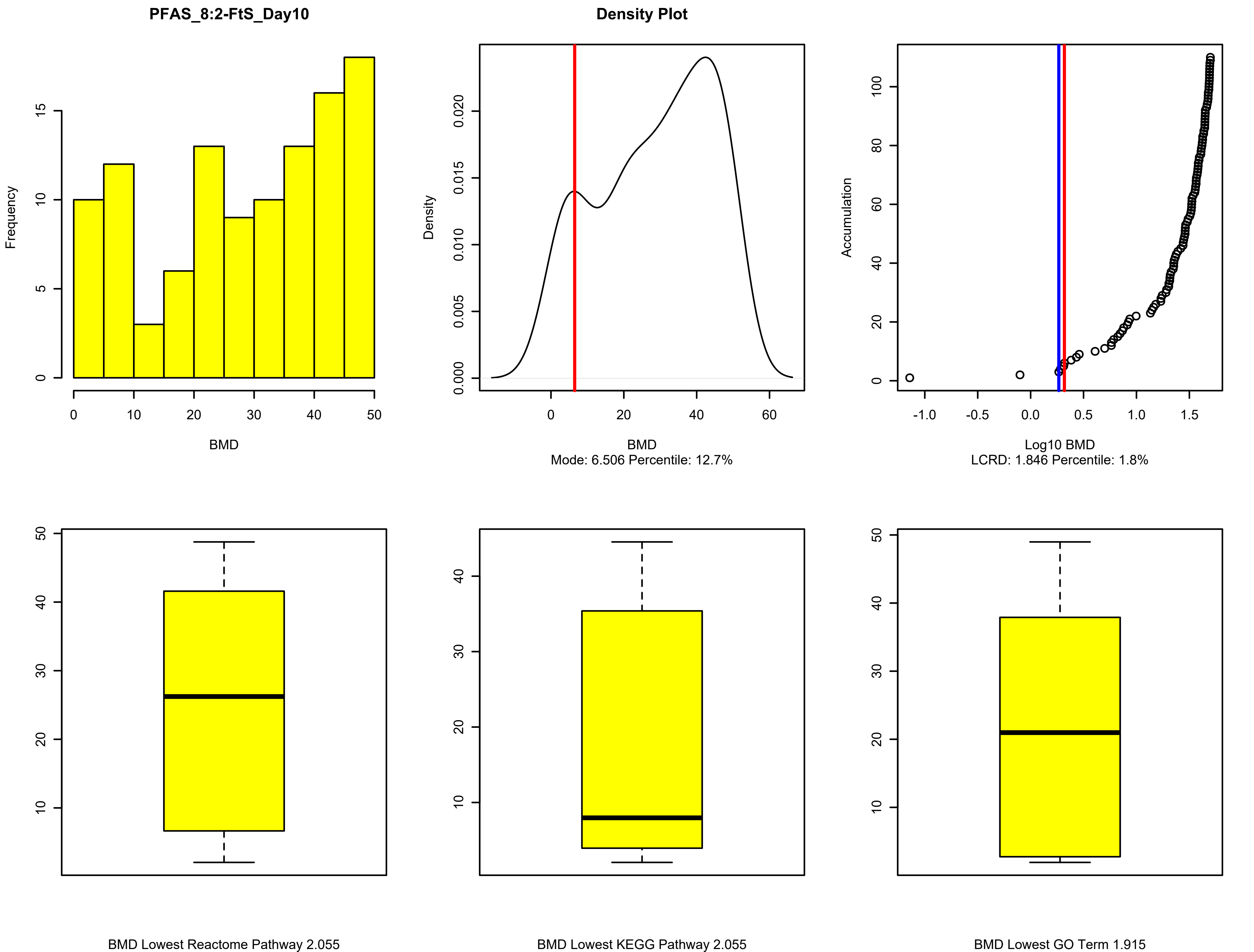

PFAS_8:2-FtS_Day10
Frequency
BMD
Density Plot
Density
BMD
Mode: 6.506 Percentile: 12.7%
Accumulation
Log10 BMD
LCRD: 1.846 Percentile: 1.8%
BMD Lowest Reactome Pathway 2.055
BMD Lowest KEGG Pathway 2.055
BMD Lowest GO Term 1.915

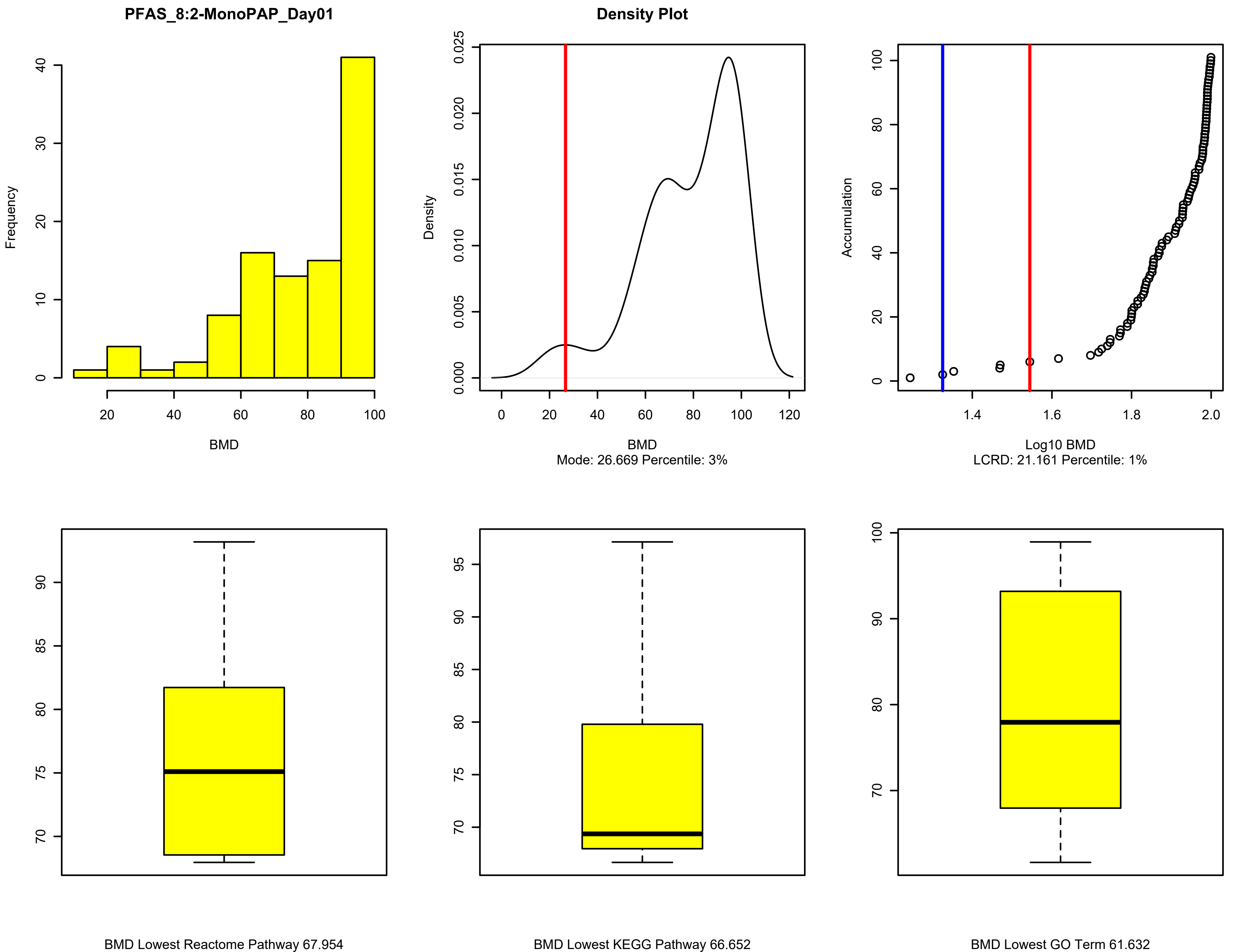
PFAS_8:2-MonoPAP_Day01
Frequency
BMD
Density Plot
Density
BMD
Mode: 26.669 Percentile: 3%
Accumulation
Log10 BMD
LCRD: 21.161 Percentile: 1%
BMD Lowest Reactome Pathway 67.954
BMD Lowest KEGG Pathway 66.652
BMD Lowest GO Term 61.632

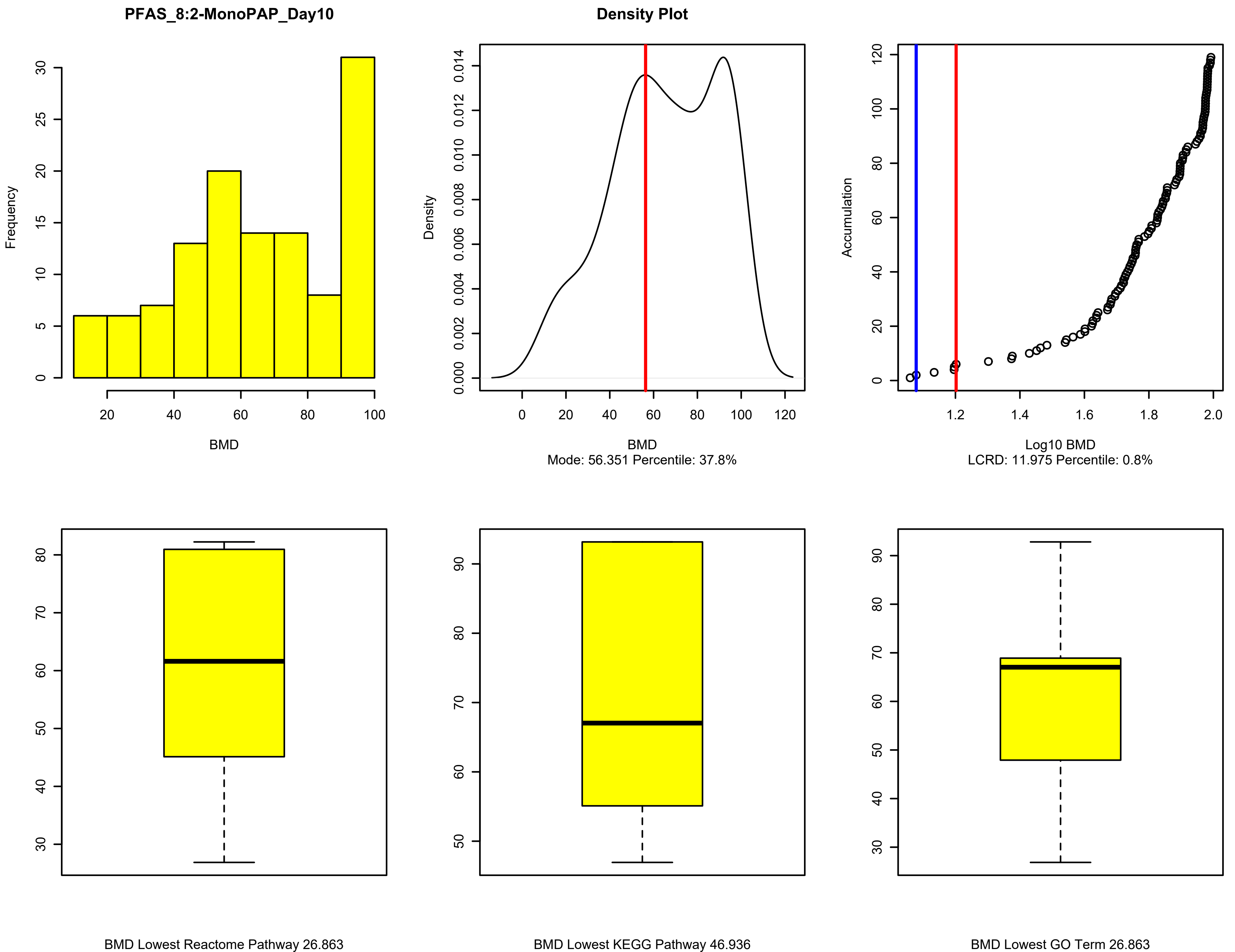
PFAS_8:2-MonoPAP_Day10
Frequency
BMD
Density Plot
Density
BMD
Mode: 56.351 Percentile: 37.8%
Accumulation
Log10 BMD
LCRD: 11.975 Percentile: 0.8%
BMD Lowest Reactome Pathway 26.863
BMD Lowest KEGG Pathway 46.936
BMD Lowest GO Term 26.863

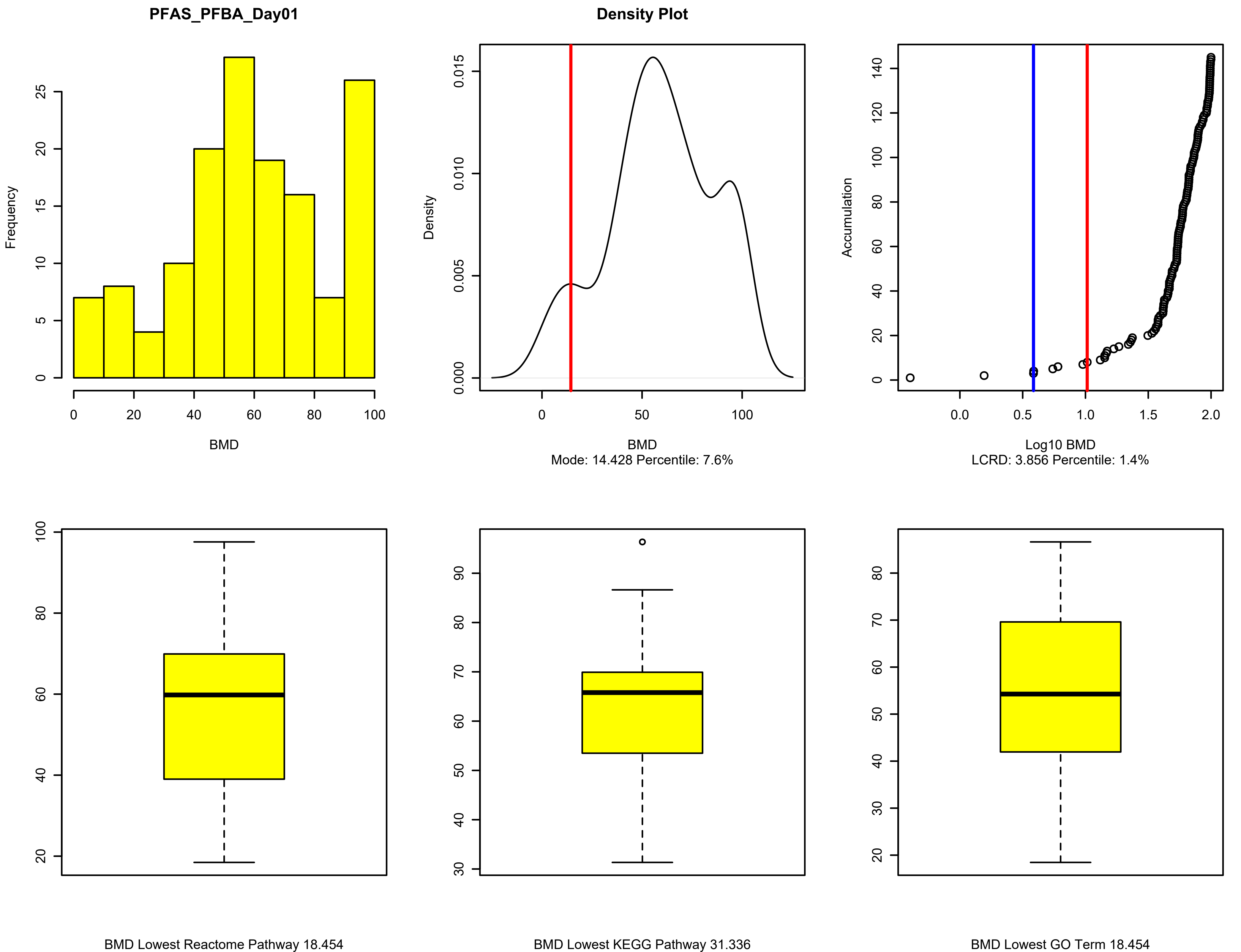
PFAS_PFBA_Day01
Frequency
BMD
Density Plot
Density
BMD
Mode: 14.428 Percentile: 7.6%
Accumulation
Log10 BMD
LCRD: 3.856 Percentile: 1.4%
BMD Lowest Reactome Pathway 18.454
BMD Lowest KEGG Pathway 31.336
BMD Lowest GO Term 18.454

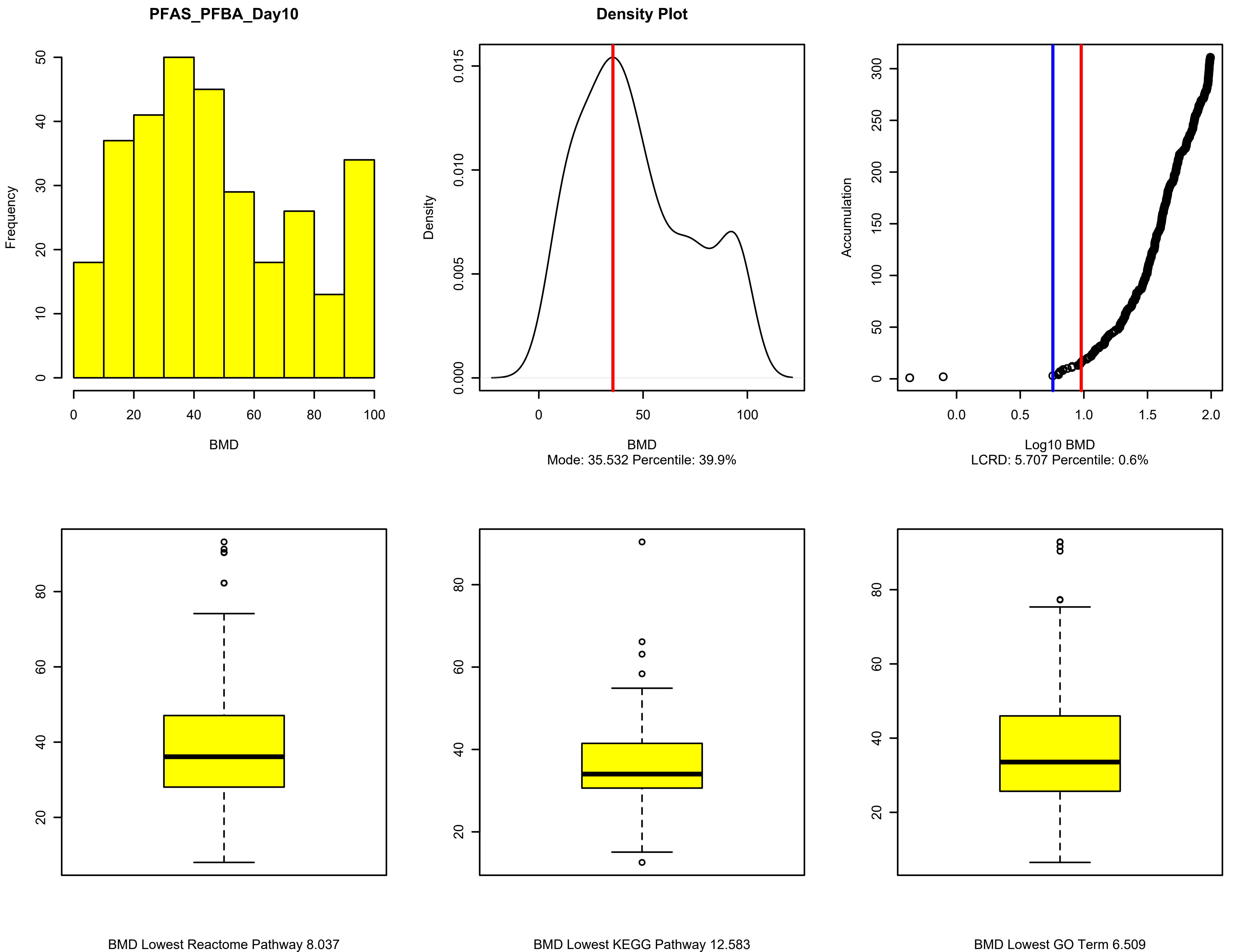

PFAS_PFBA_Day10
Frequency
BMD
Density Plot
Density
BMD
Mode: 35.532 Percentile: 39.9%
Accumulation
Log10 BMD
LCRD: 5.707 Percentile: 0.6%
BMD Lowest Reactome Pathway 8.037
BMD Lowest KEGG Pathway 12.583
BMD Lowest GO Term 6.509

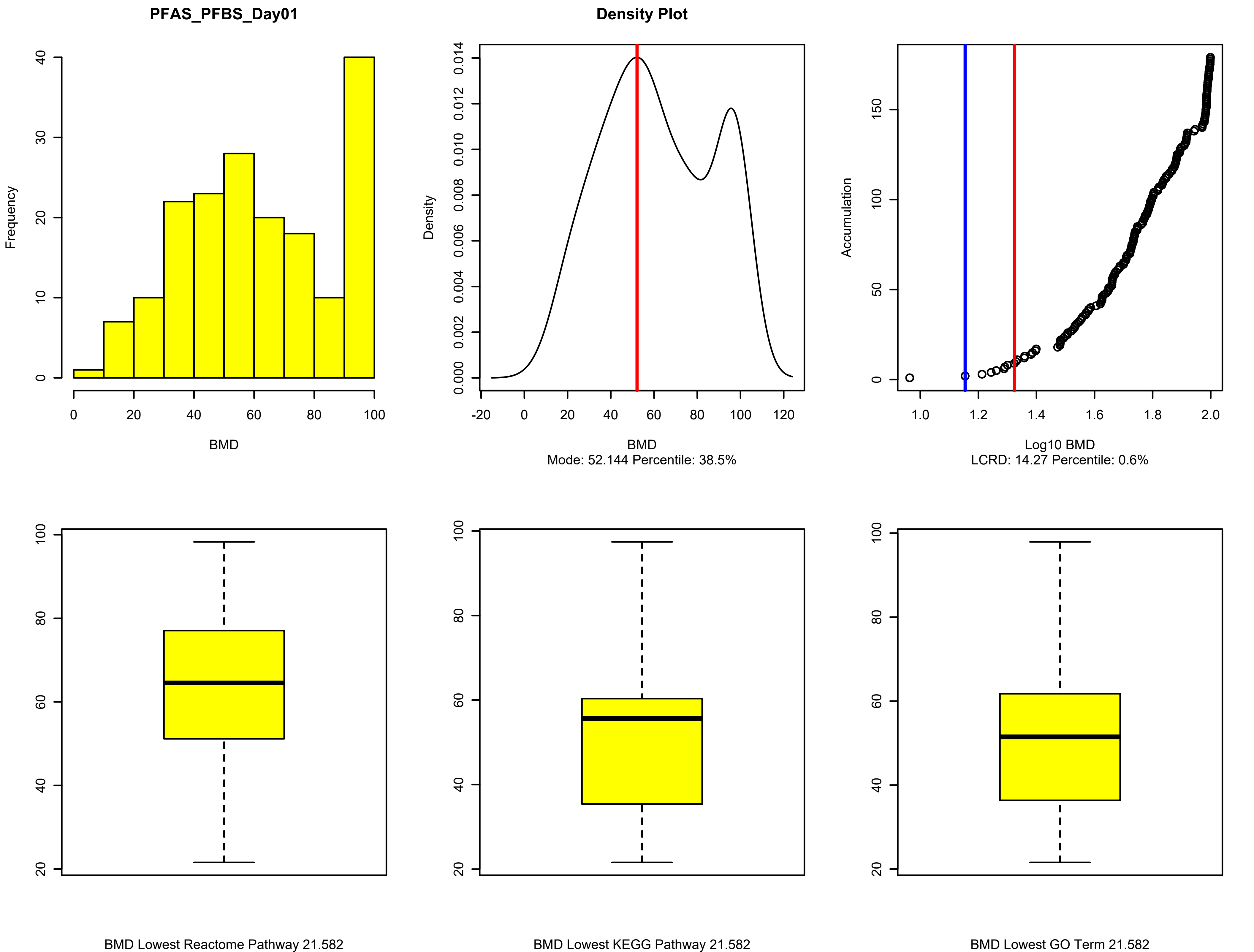
PFAS_PFBS_Day01
Frequency
BMD
Density Plot
Density
BMD
Mode: 52.144 Percentile: 38.5%
Accumulation
Log10 BMD
LCRD: 14.27 Percentile: 0.6%
BMD Lowest Reactome Pathway 21.582
BMD Lowest KEGG Pathway 21.582
BMD Lowest GO Term 21.582

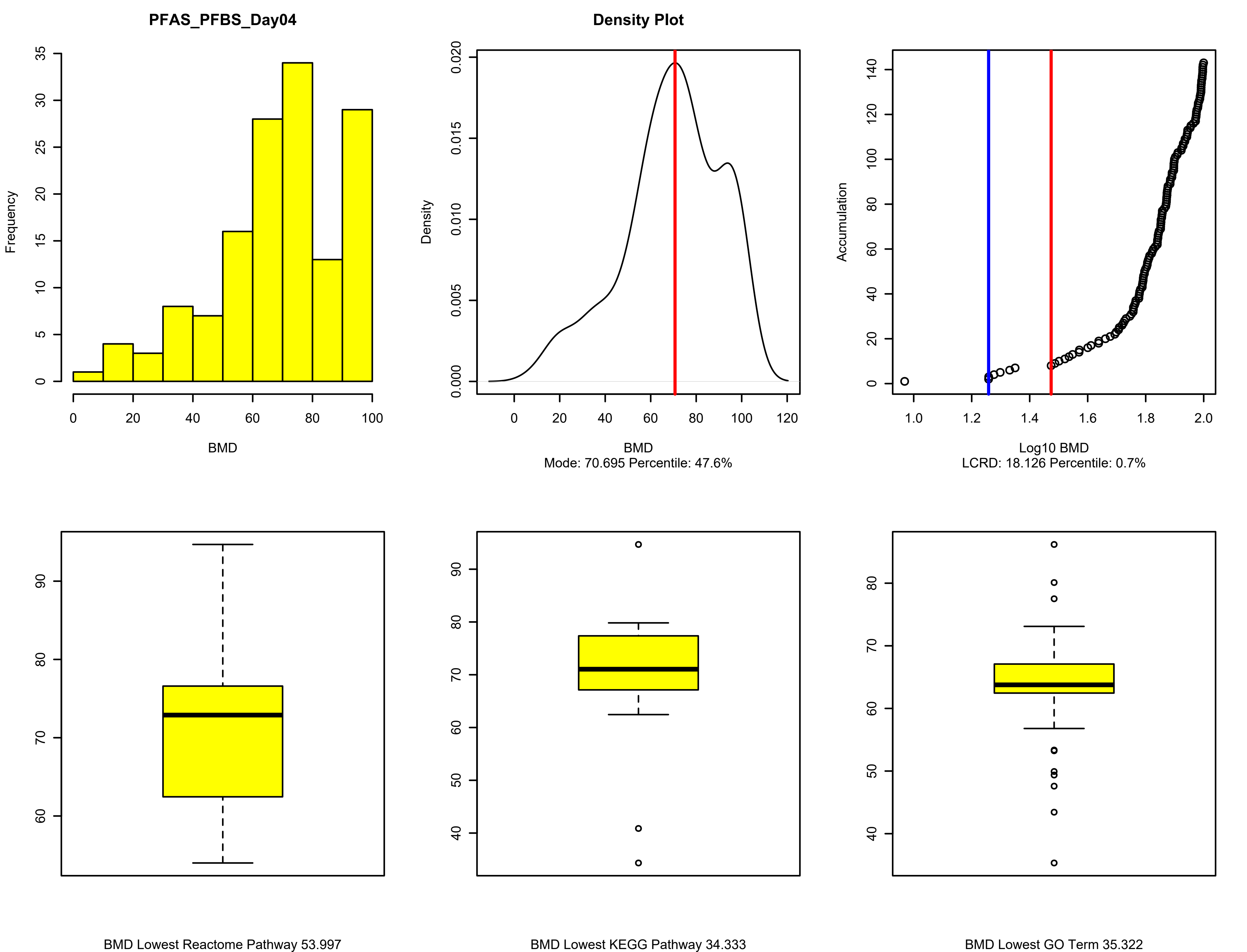

PFAS_PFBS_Day04
Frequency
BMD
Density Plot
Density
BMD
Mode: 70.695 Percentile: 47.6%
Accumulation
Log10 BMD
LCRD: 18.126 Percentile: 0.7%
BMD Lowest Reactome Pathway 53.997
BMD Lowest KEGG Pathway 34.333
BMD Lowest GO Term 35.322

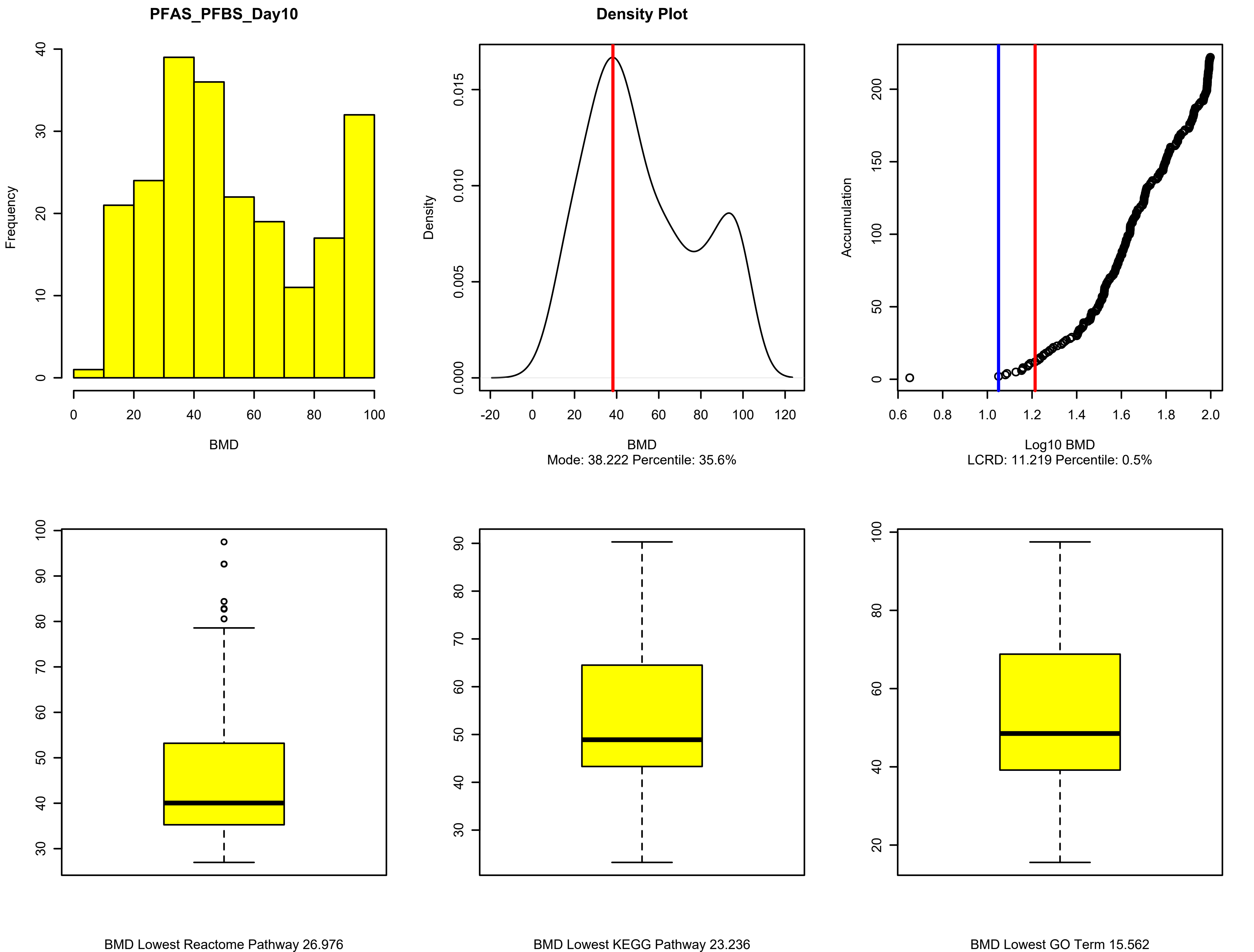
PFAS_PFBS_Day10
Frequency
BMD
Density Plot
Density
BMD
Mode: 38.222 Percentile: 35.6%
Accumulation
Log10 BMD
LCRD: 11.219 Percentile: 0.5%
BMD Lowest Reactome Pathway 26.976
BMD Lowest KEGG Pathway 23.236
BMD Lowest GO Term 15.562

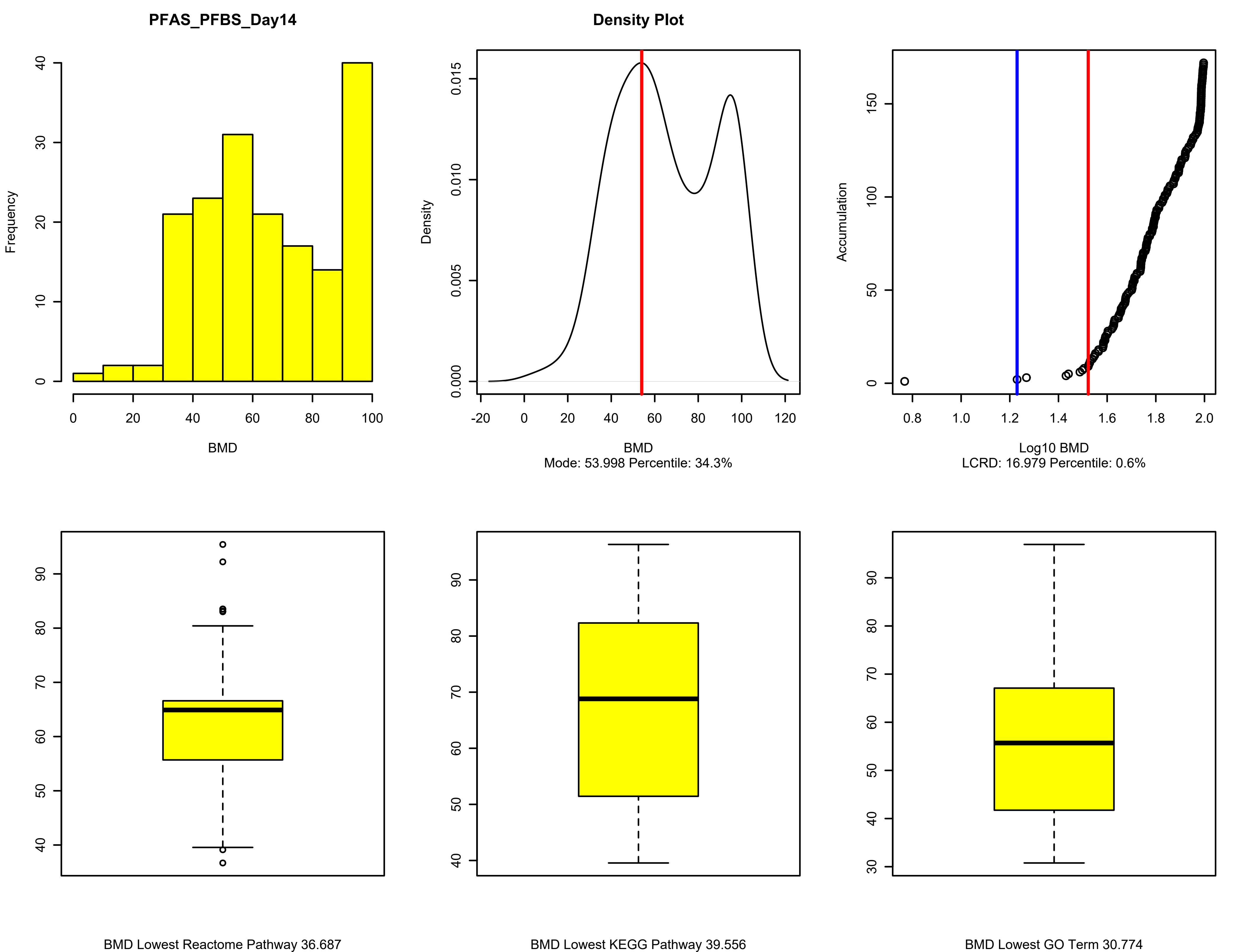

PFAS_PFBS_Day14
Frequency
BMD
Density Plot
Density
BMD
Mode: 53.998 Percentile: 34.3%
Accumulation
Log10 BMD
LCRD: 16.979 Percentile: 0.6%
BMD Lowest Reactome Pathway 36.687
BMD Lowest KEGG Pathway 39.556
BMD Lowest GO Term 30.774

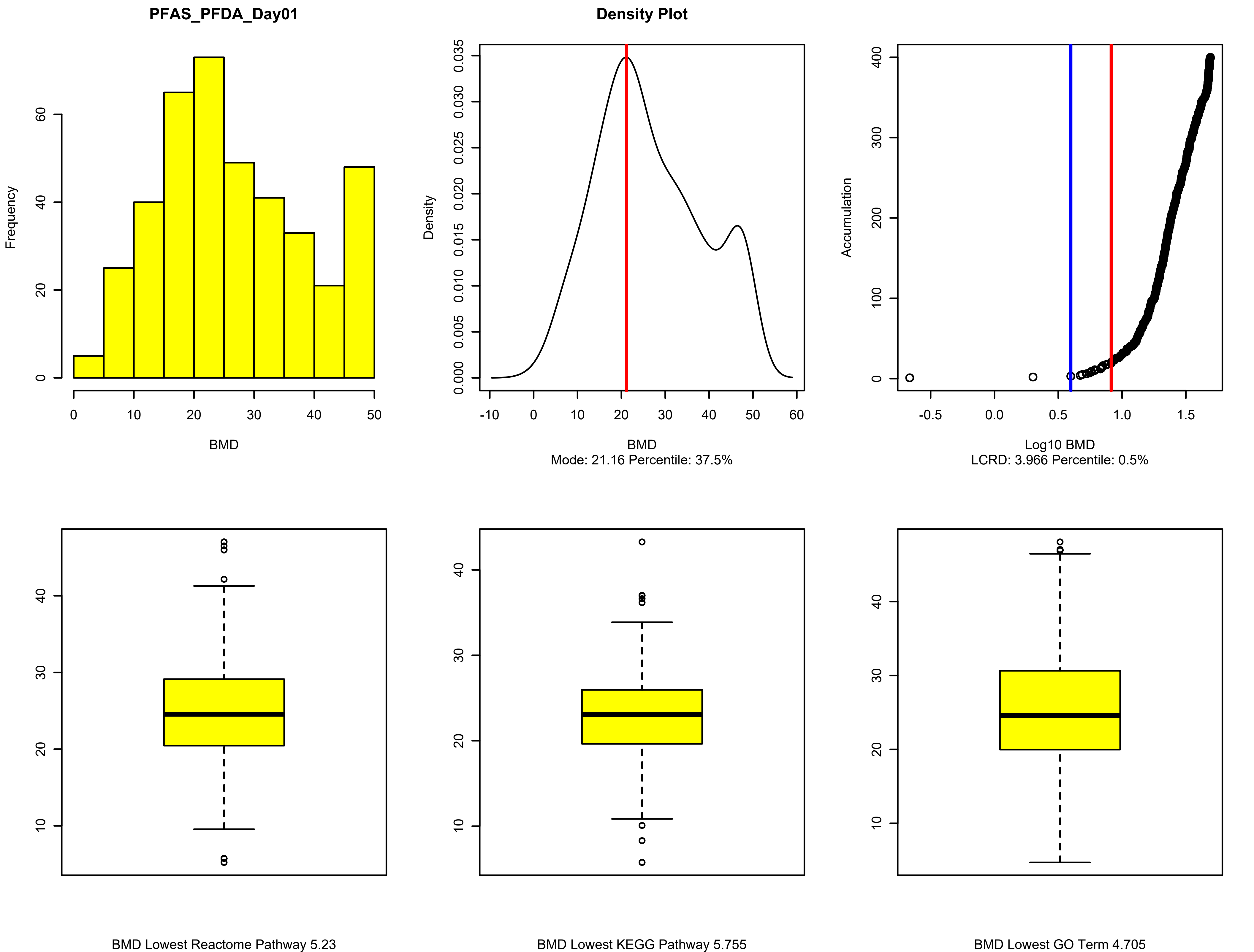

PFAS_PFDA_Day01
Frequency
BMD
Density Plot
Density
BMD
Mode: 21.16 Percentile: 37.5%
Accumulation
Log10 BMD
LCRD: 3.966 Percentile: 0.5%
BMD Lowest Reactome Pathway 5.23
BMD Lowest KEGG Pathway 5.755
BMD Lowest GO Term 4.705

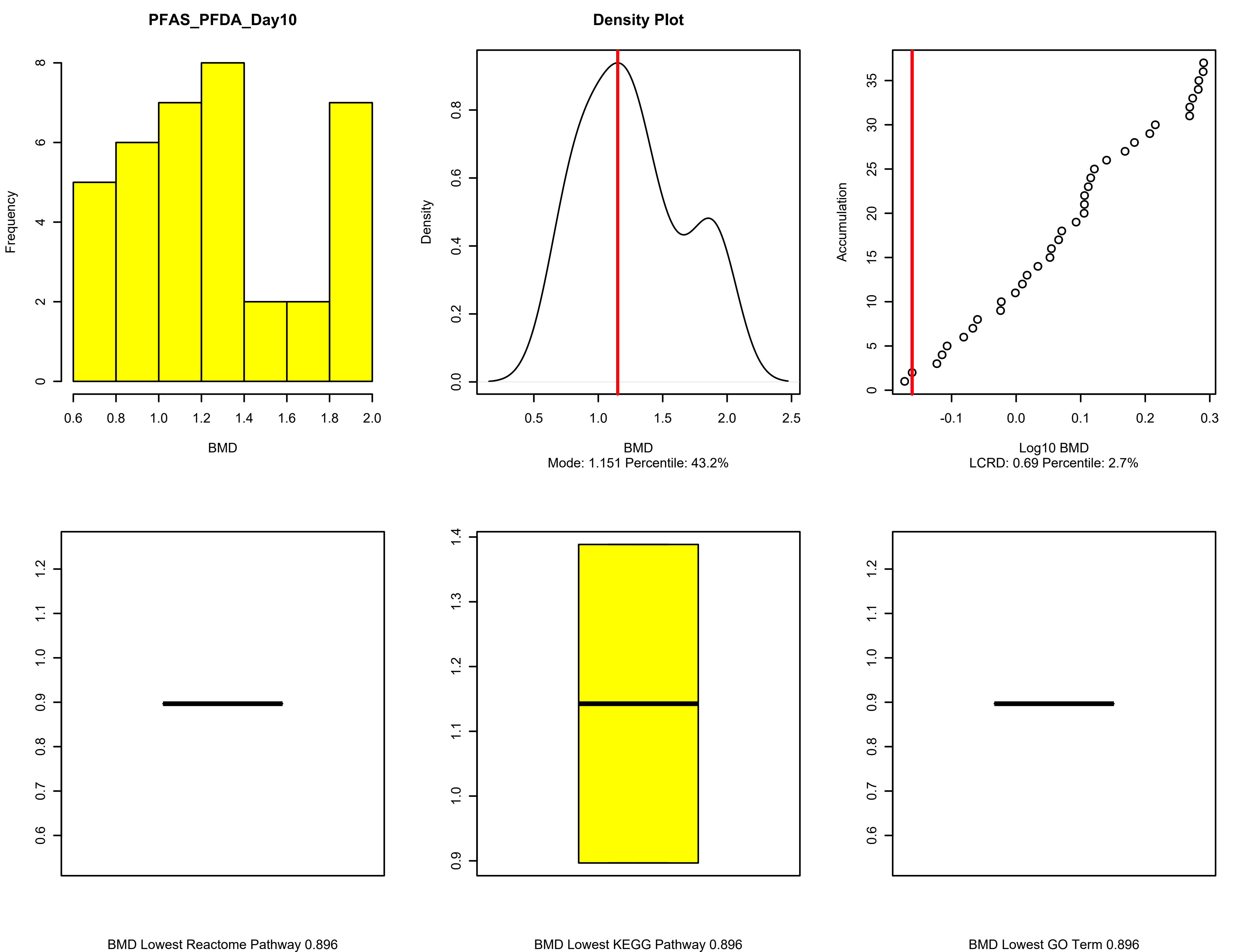
PFAS_PFDA_Day10
Frequency
BMD
Density Plot
Density
BMD
Mode: 1.151 Percentile: 43.2%
Accumulation
Log10 BMD
LCRD: 0.69 Percentile: 2.7%
BMD Lowest Reactome Pathway 0.896
BMD Lowest KEGG Pathway 0.896
BMD Lowest GO Term 0.896

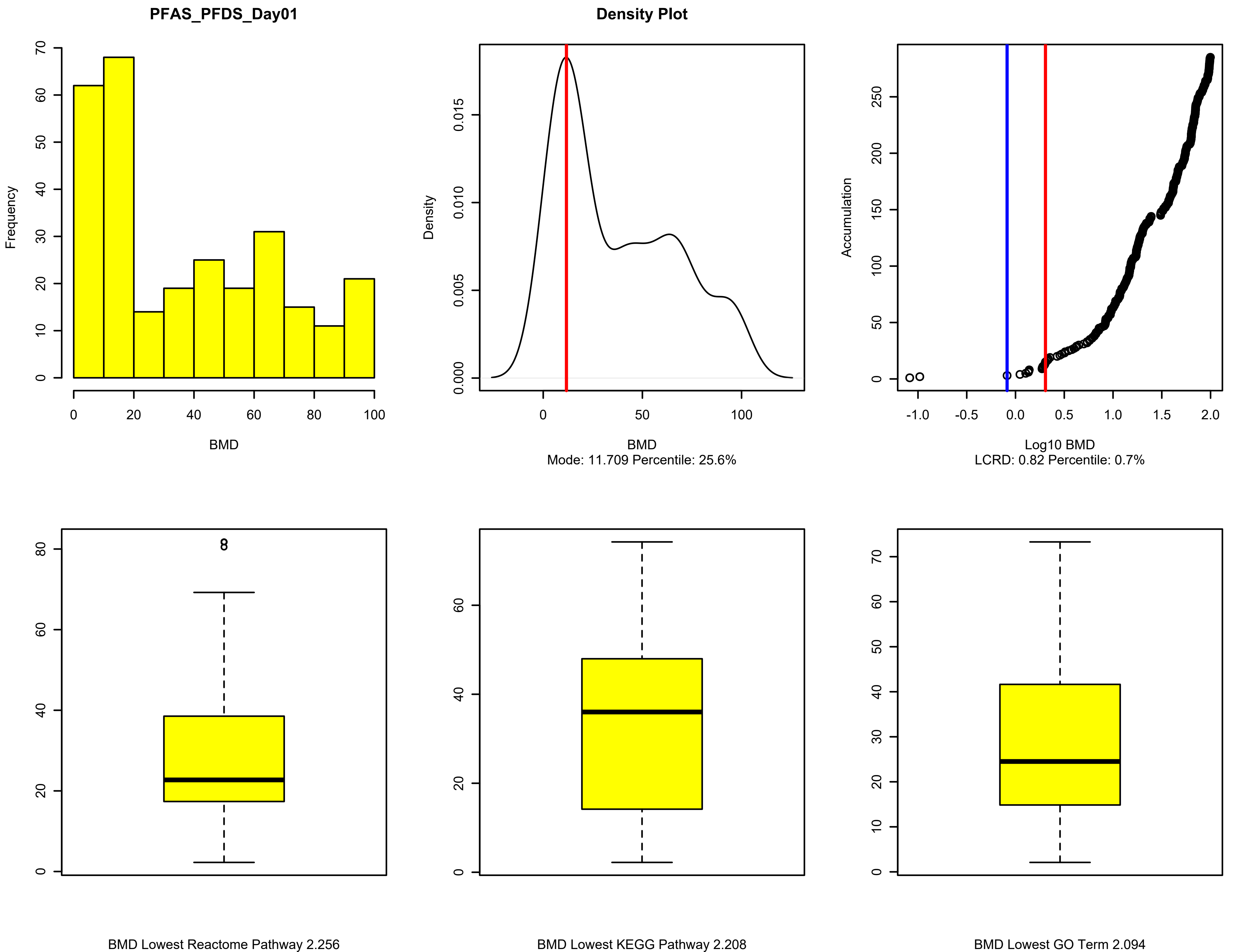

PFAS_PFDS_Day01
Frequency
BMD
Density Plot
Density
BMD
Mode: 11.709 Percentile: 25.6%
Accumulation
Log10 BMD
LCRD: 0.82 Percentile: 0.7%
BMD Lowest Reactome Pathway 2.256
BMD Lowest KEGG Pathway 2.208
BMD Lowest GO Term 2.094

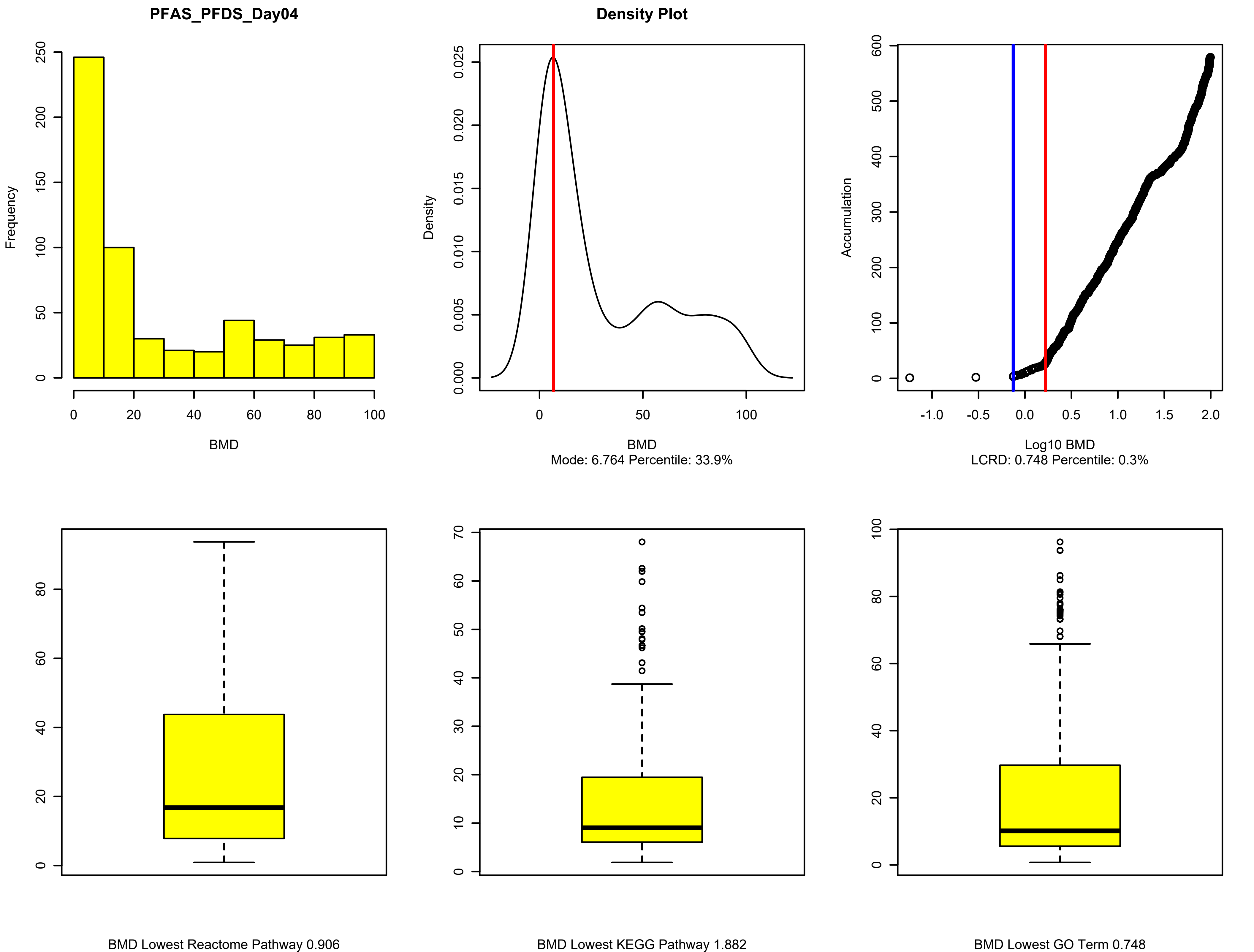
PFAS_PFDS_Day04
Frequency
BMD
Density Plot
Density
BMD
Mode: 6.764 Percentile: 33.9%
Accumulation
Log10 BMD
LCRD: 0.748 Percentile: 0.3%
BMD Lowest Reactome Pathway 0.906
BMD Lowest KEGG Pathway 1.882
BMD Lowest GO Term 0.748

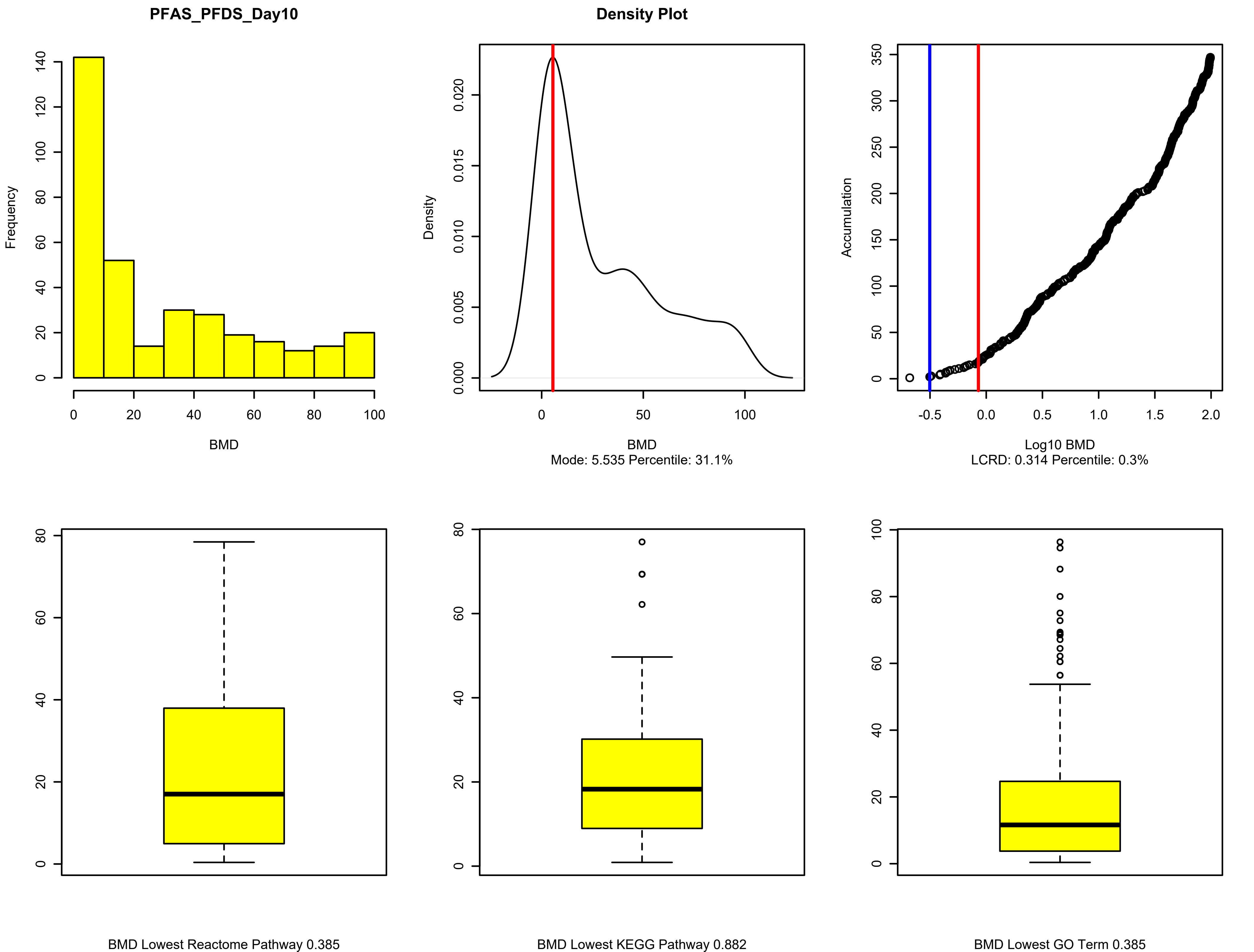

PFAS_PFDS_Day10
Frequency
BMD
Density Plot
Density
BMD
Mode: 5.535 Percentile: 31.1%
Accumulation
Log10 BMD
LCRD: 0.314 Percentile: 0.3%
BMD Lowest Reactome Pathway 0.385
BMD Lowest KEGG Pathway 0.882
BMD Lowest GO Term 0.385

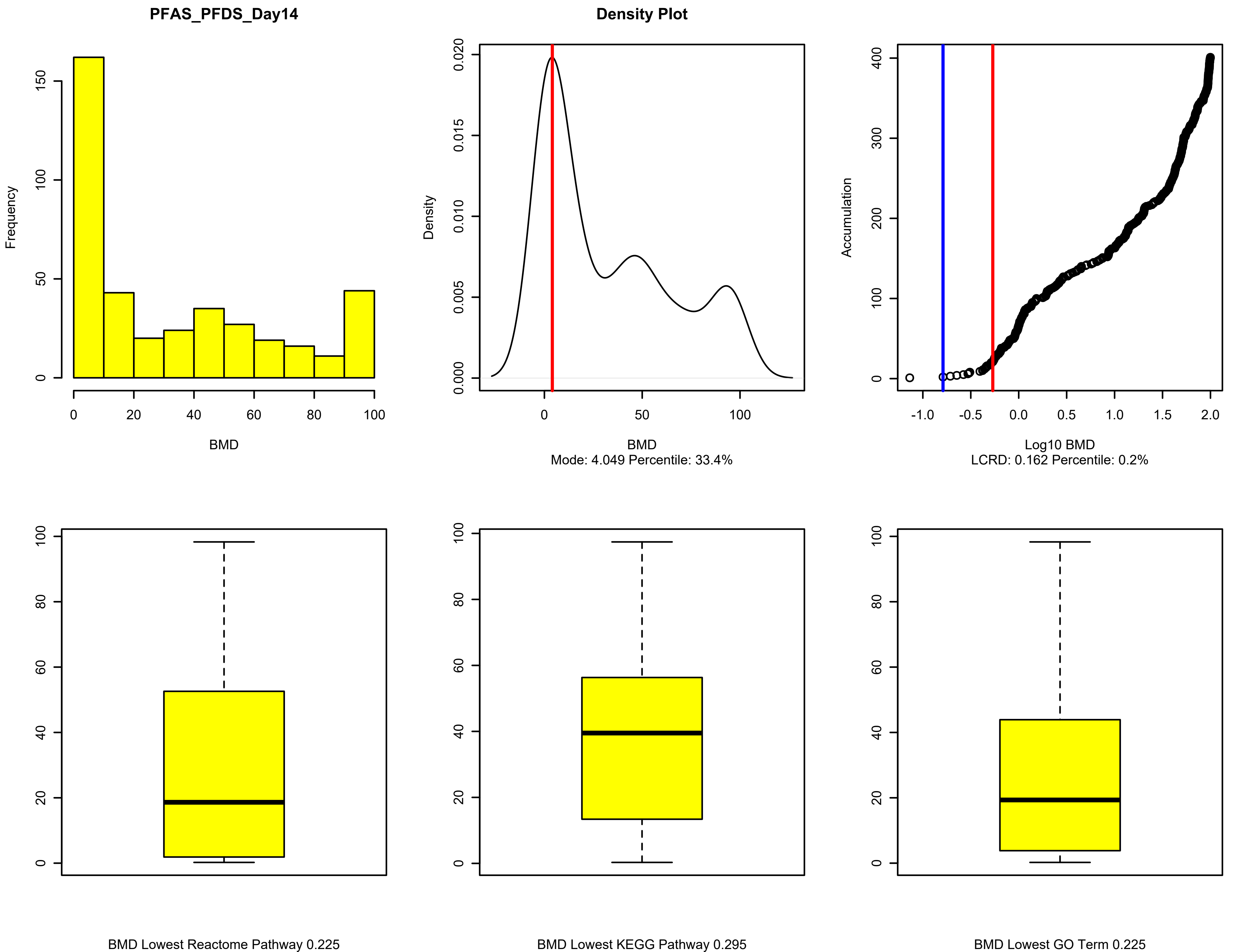

PFAS_PFDS_Day14
Frequency
BMD
Density Plot
Density
BMD
Mode: 4.049 Percentile: 33.4%
Accumulation
Log10 BMD
LCRD: 0.162 Percentile: 0.2%
BMD Lowest Reactome Pathway 0.225
BMD Lowest KEGG Pathway 0.295
BMD Lowest GO Term 0.225

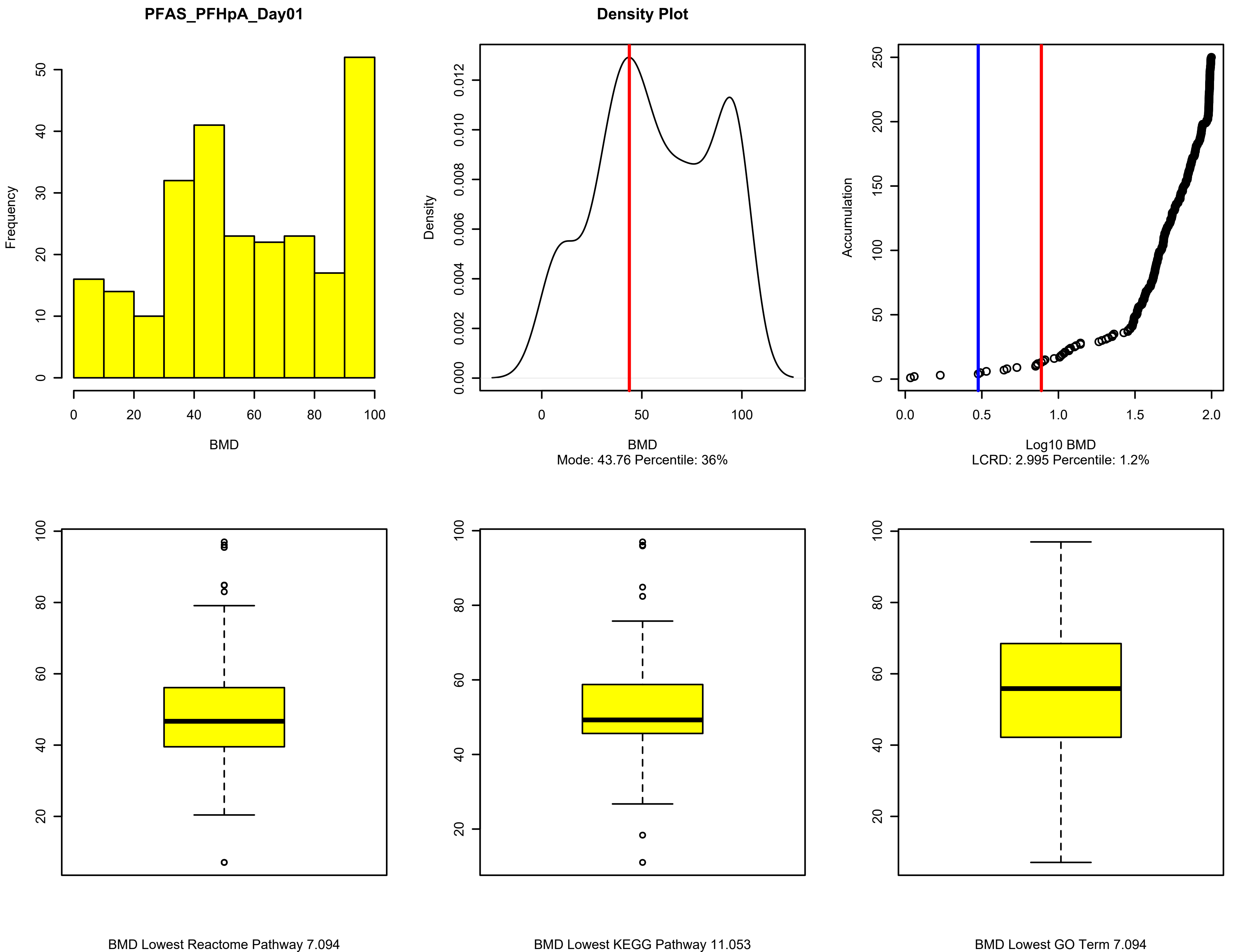
PFAS_PFHpA_Day01
Frequency
BMD
Density Plot
Density
BMD
Mode: 43.76 Percentile: 36%
Accumulation
Log10 BMD
LCRD: 2.995 Percentile: 1.2%
BMD Lowest Reactome Pathway 7.094
BMD Lowest KEGG Pathway 11.053
BMD Lowest GO Term 7.094

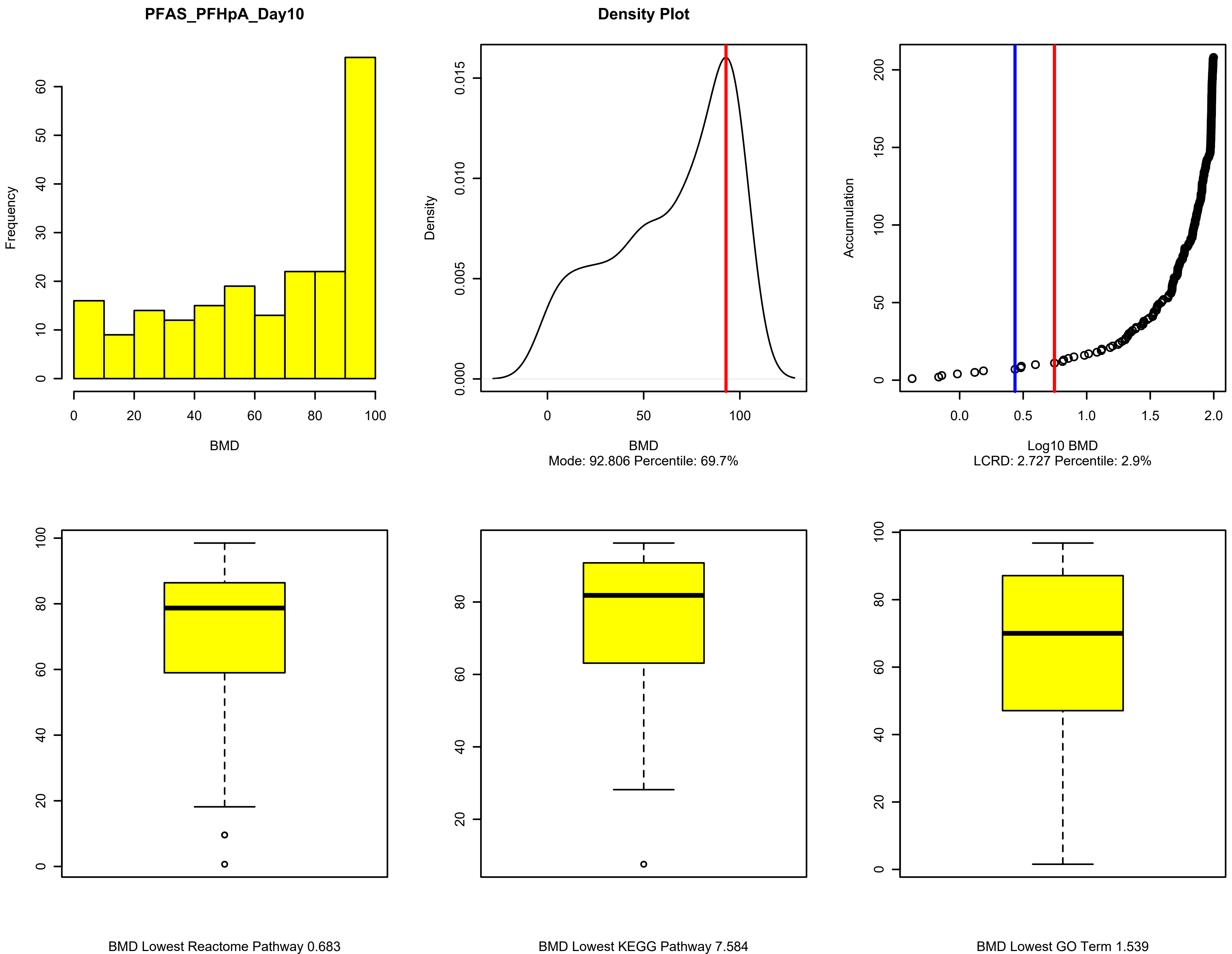

PFAS_PFHpA_Day10
Frequency
BMD
Density Plot
Density
BMD
Mode: 92.806 Percentile: 69.7%
Accumulation
Log10 BMD
LCRD: 2.727 Percentile: 2.9%
BMD Lowest Reactome Pathway 0.683
BMD Lowest KEGG Pathway 7.584
BMD Lowest GO Term 1.539

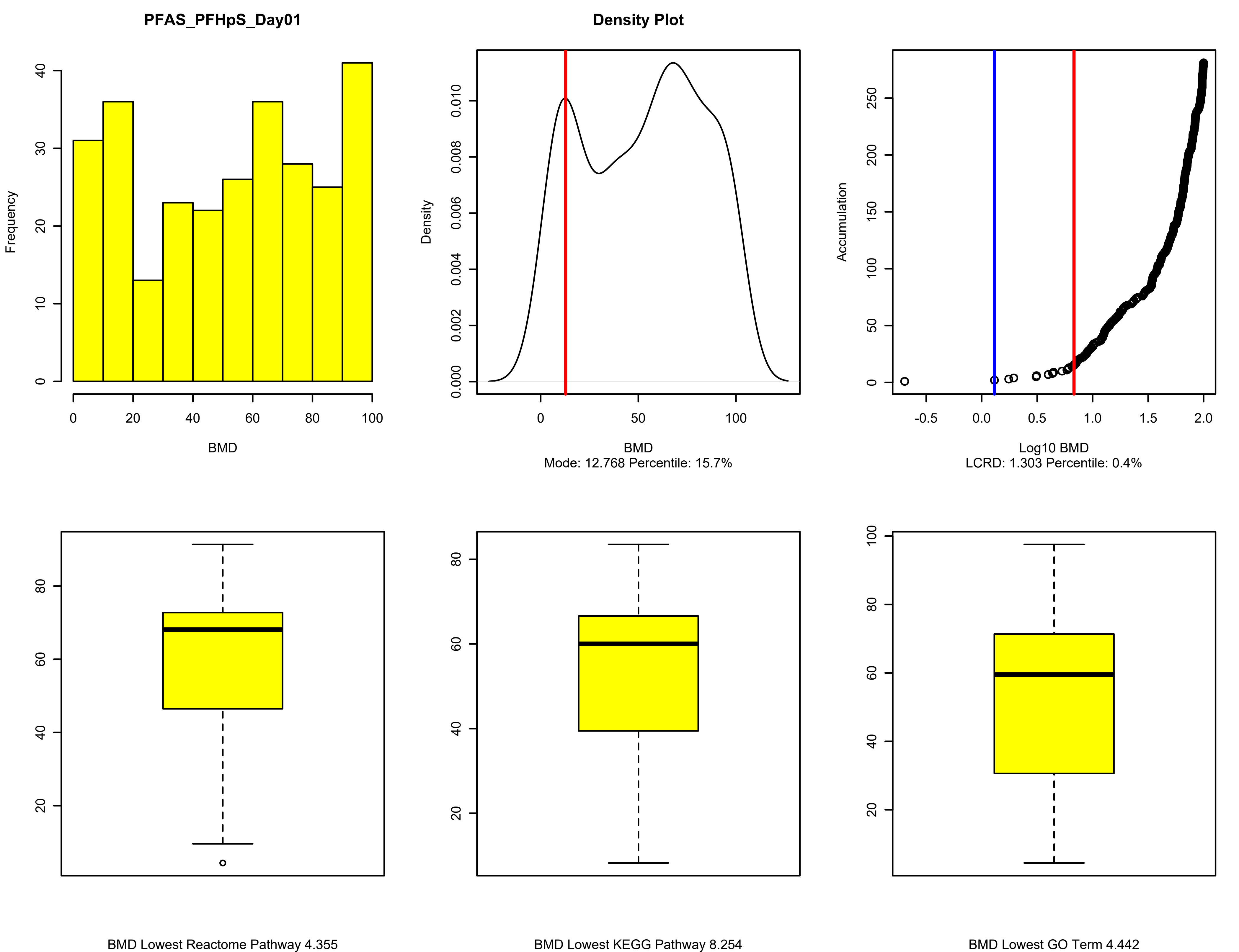

PFAS_PFHpS_Day01
Frequency
BMD
Density Plot
Density
BMD
Mode: 12.768 Percentile: 15.7%
Accumulation
Log10 BMD
LCRD: 1.303 Percentile: 0.4%
BMD Lowest Reactome Pathway 4.355
BMD Lowest KEGG Pathway 8.254
BMD Lowest GO Term 4.442

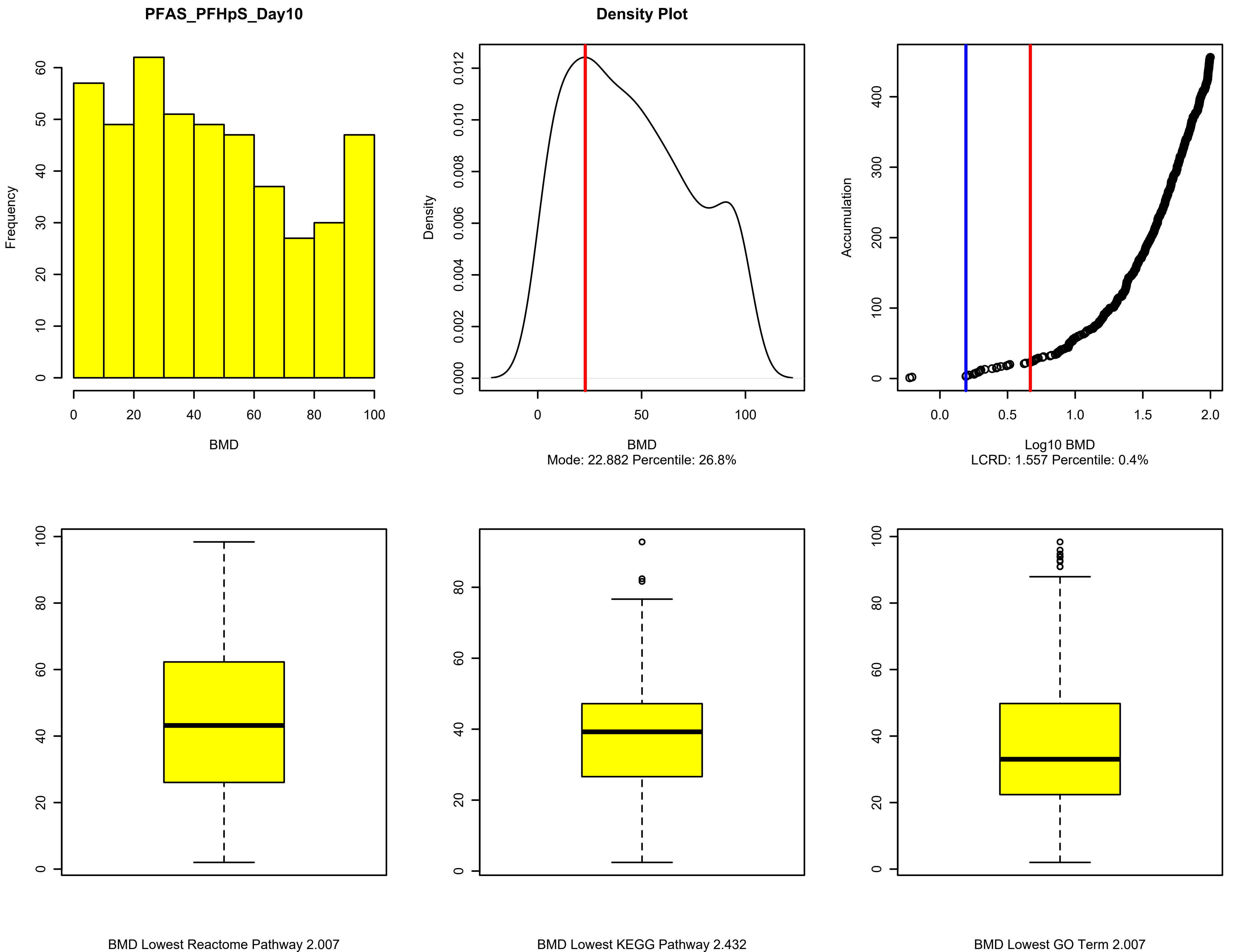
PFAS_PFHpS_Day10
Frequency
BMD
Density Plot
Density
BMD
Mode: 22.882 Percentile: 26.8%
Accumulation
Log10 BMD
LCRD: 1.557 Percentile: 0.4%
BMD Lowest Reactome Pathway 2.007
BMD Lowest KEGG Pathway 2.432
BMD Lowest GO Term 2.007

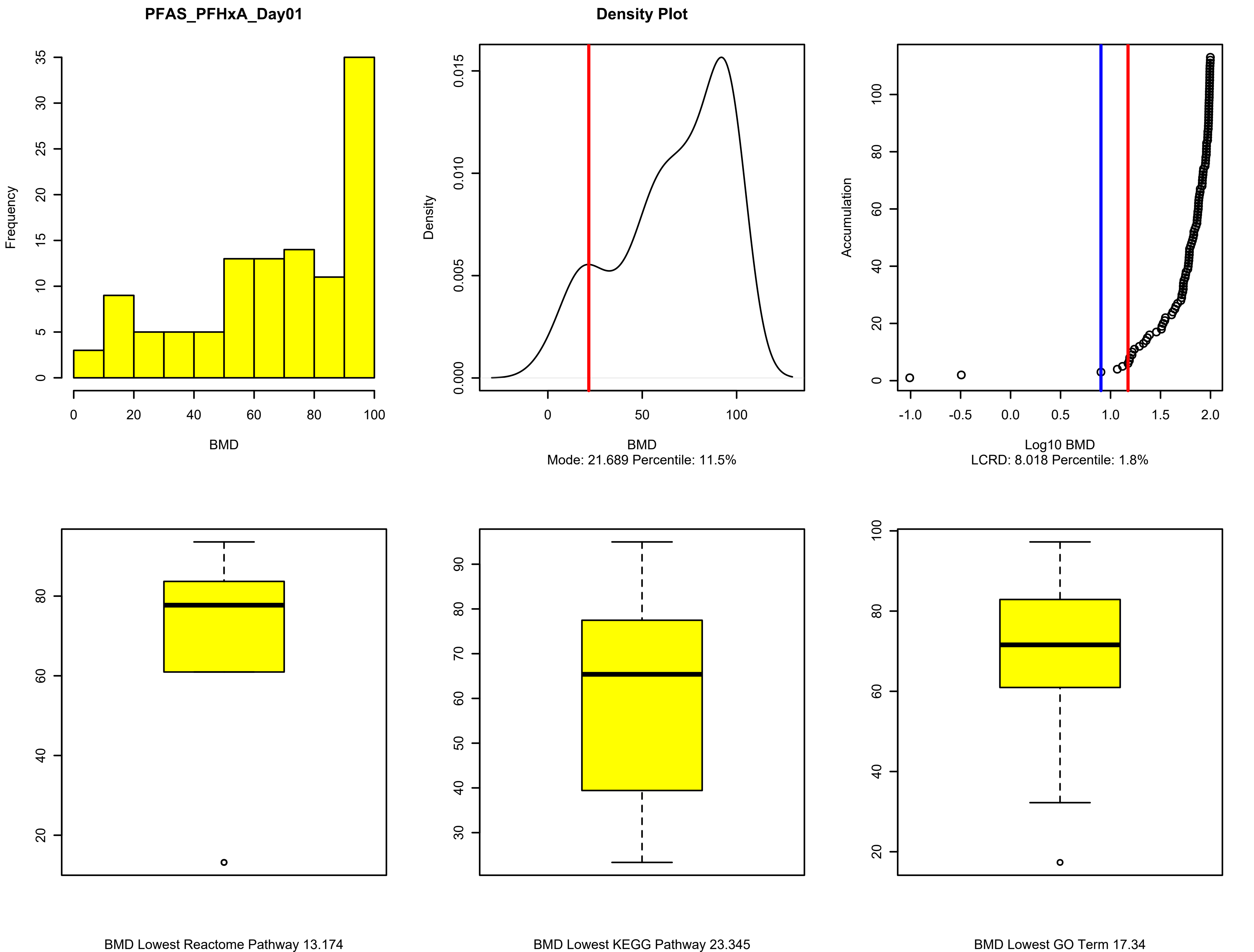

PFAS_PFHxA_Day01
Frequency
BMD
Density Plot
Density
BMD
Mode: 21.689 Percentile: 11.5%
Accumulation
Log10 BMD
LCRD: 8.018 Percentile: 1.8%
BMD Lowest Reactome Pathway 13.174
BMD Lowest KEGG Pathway 23.345
BMD Lowest GO Term 17.34

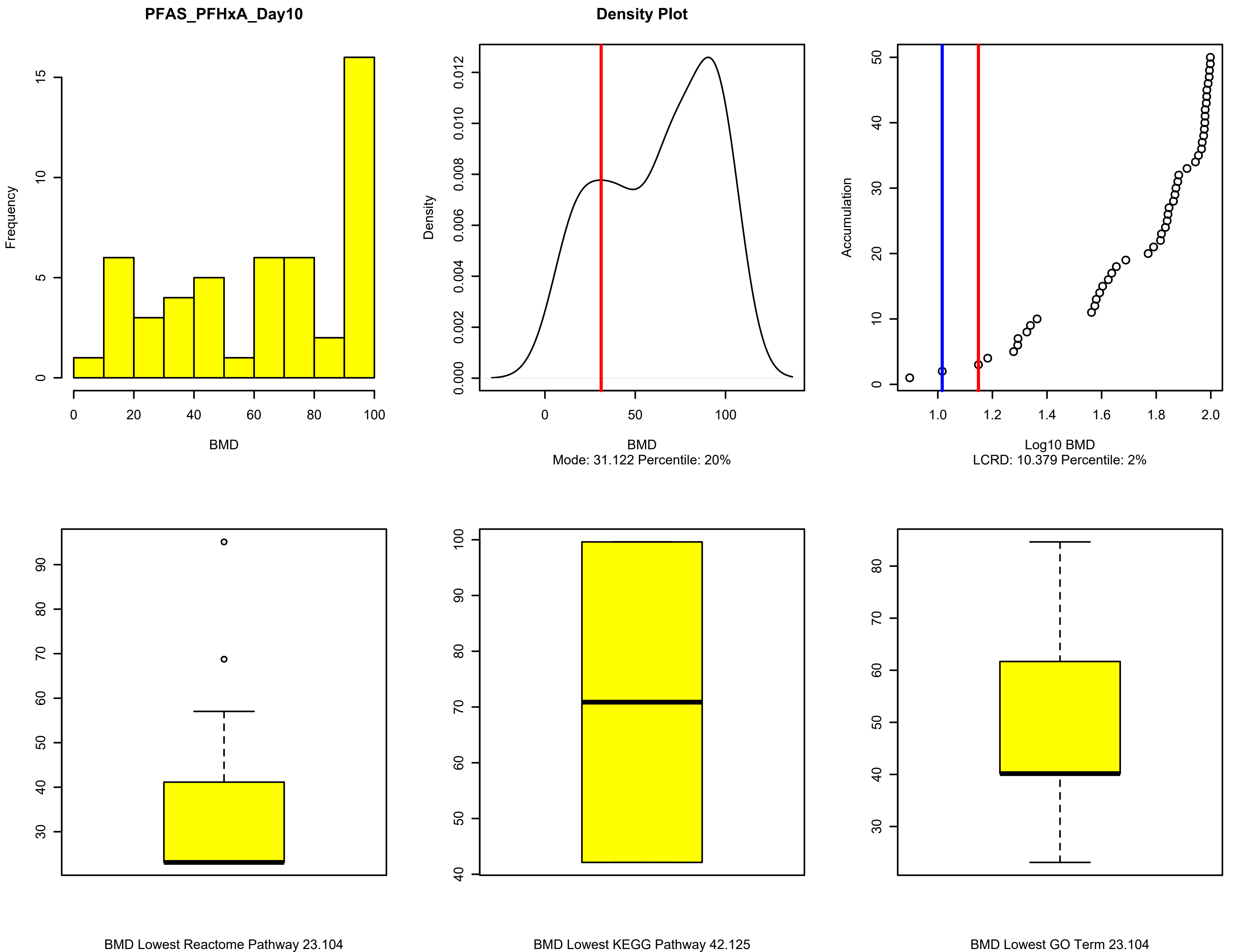

PFAS_PFHxA_Day10
Frequency
BMD
Density Plot
Density
BMD
Mode: 31.122 Percentile: 20%
Accumulation
Log10 BMD
LCRD: 10.379 Percentile: 2%
BMD Lowest Reactome Pathway 23.104
BMD Lowest KEGG Pathway 42.125
BMD Lowest GO Term 23.104

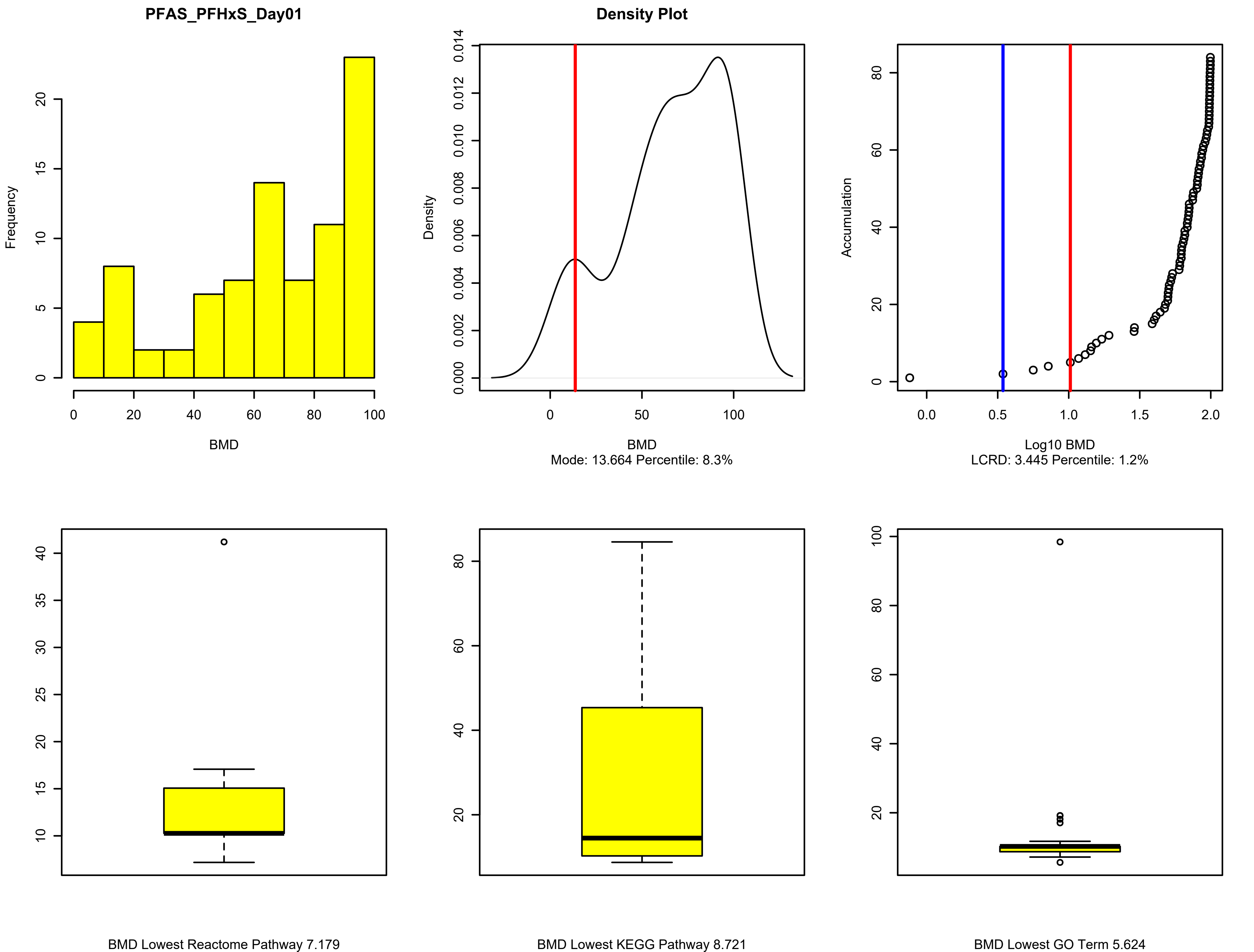

PFAS_PFHxS_Day01
Frequency
BMD
Density Plot
Density
BMD
Mode: 13.664 Percentile: 8.3%
Accumulation
Log10 BMD
LCRD: 3.445 Percentile: 1.2%
BMD Lowest Reactome Pathway 7.179
BMD Lowest KEGG Pathway 8.721
BMD Lowest GO Term 5.624

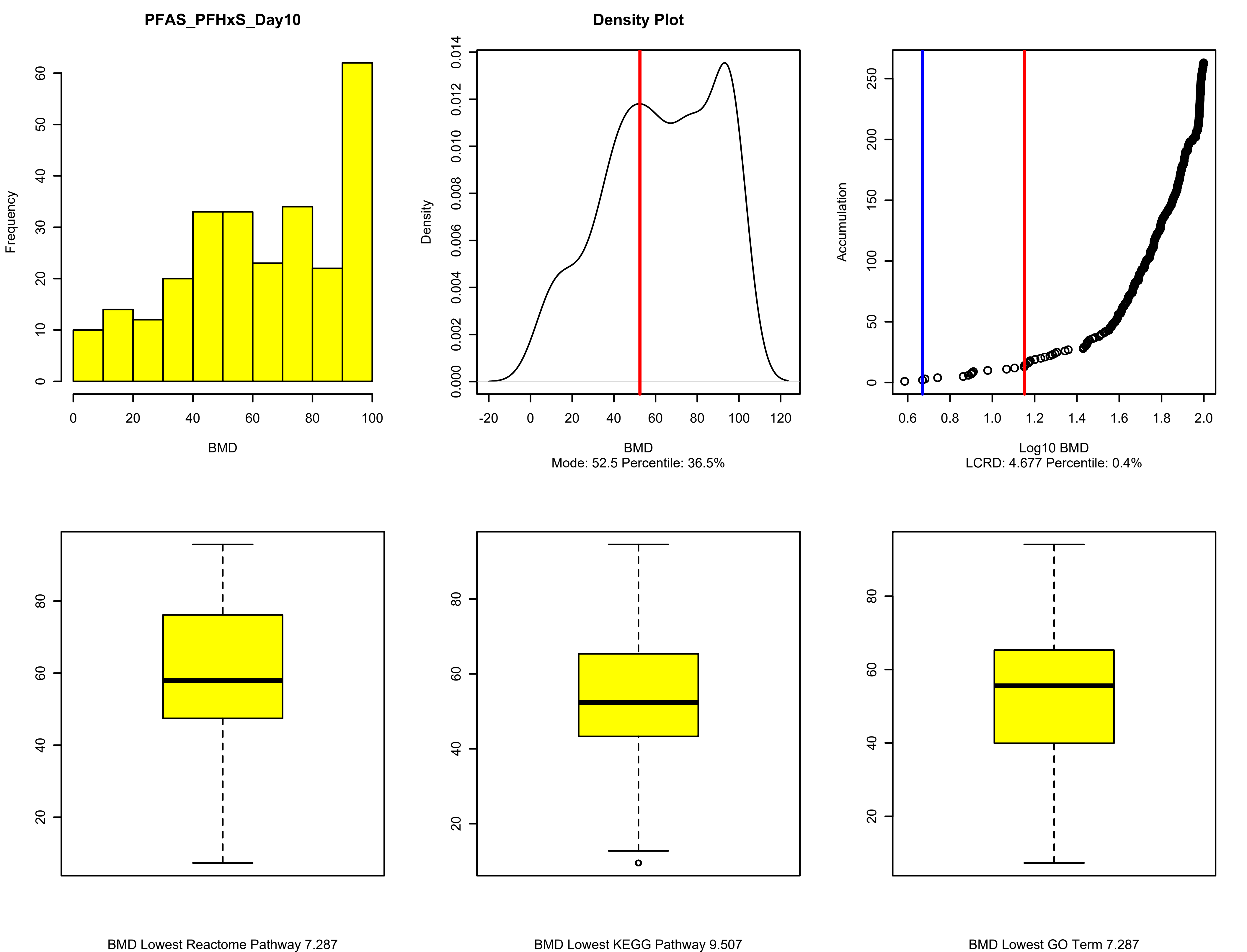

PFAS_PFHxS_Day10
Frequency
BMD
Density Plot
Density
BMD
Mode: 52.5 Percentile: 36.5%
Accumulation
Log10 BMD
LCRD: 4.677 Percentile: 0.4%
BMD Lowest Reactome Pathway 7.287
BMD Lowest KEGG Pathway 9.507
BMD Lowest GO Term 7.287

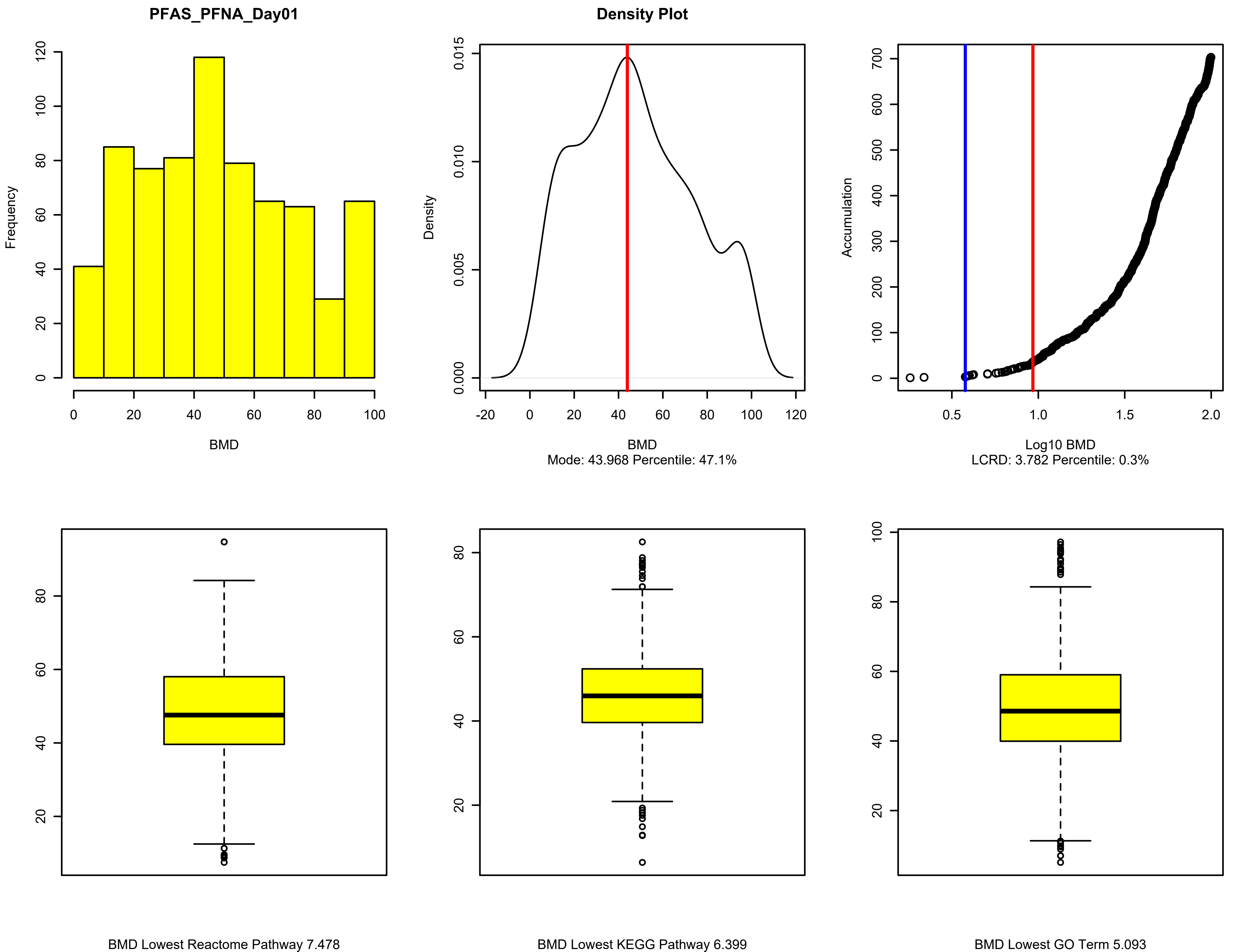
PFAS_PFNA_Day01
Frequency
BMD
Density Plot
Density
BMD
Mode: 43.968 Percentile: 47.1%
Accumulation
Log10 BMD
LCRD: 3.782 Percentile: 0.3%
BMD Lowest Reactome Pathway 7.478
BMD Lowest KEGG Pathway 6.399
BMD Lowest GO Term 5.093

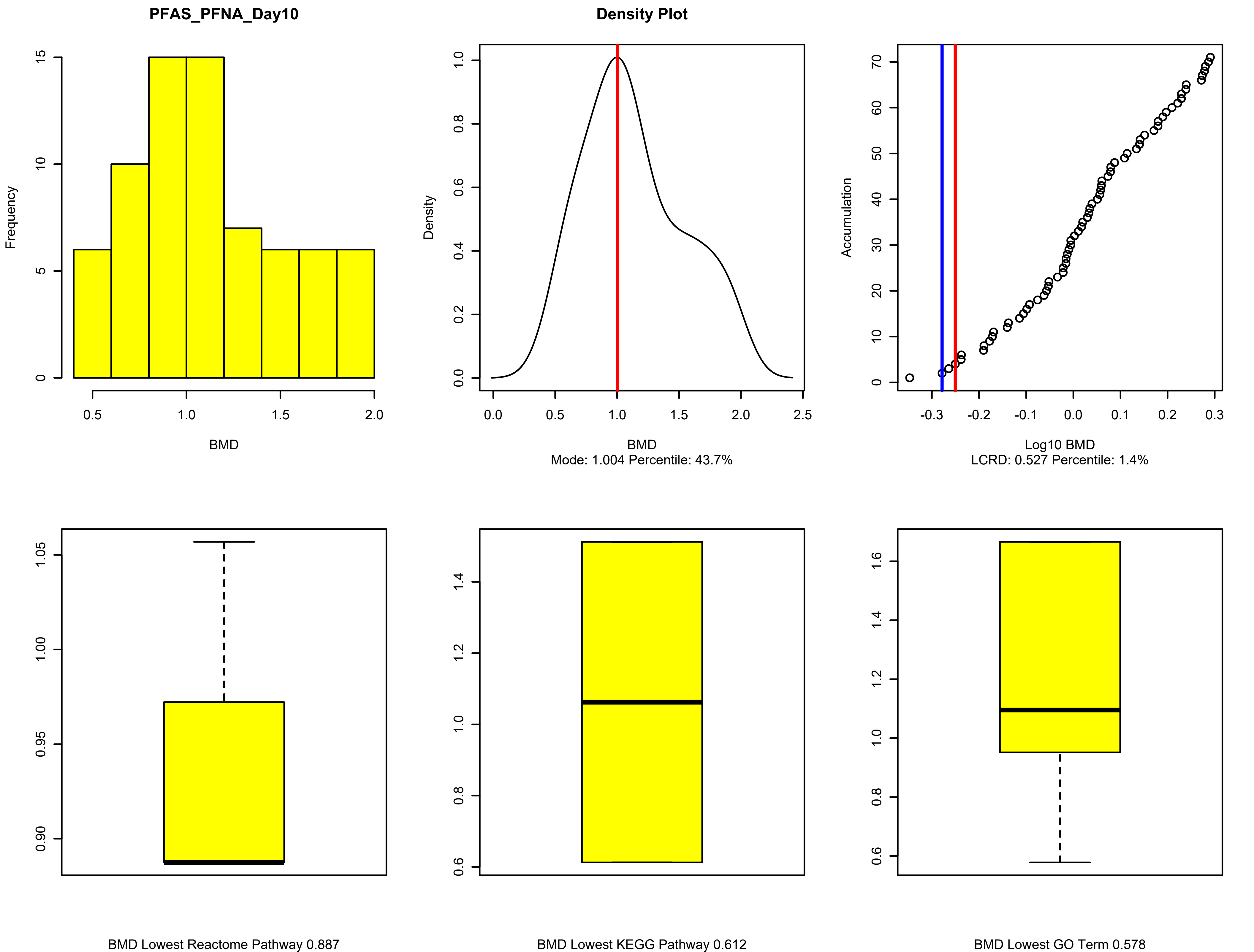

PFAS_PFNA_Day10
Frequency
BMD
Density Plot
Density
BMD
Mode: 1.004 Percentile: 43.7%
Accumulation
Log10 BMD
LCRD: 0.527 Percentile: 1.4%
BMD Lowest Reactome Pathway 0.887
BMD Lowest KEGG Pathway 0.612
BMD Lowest GO Term 0.578

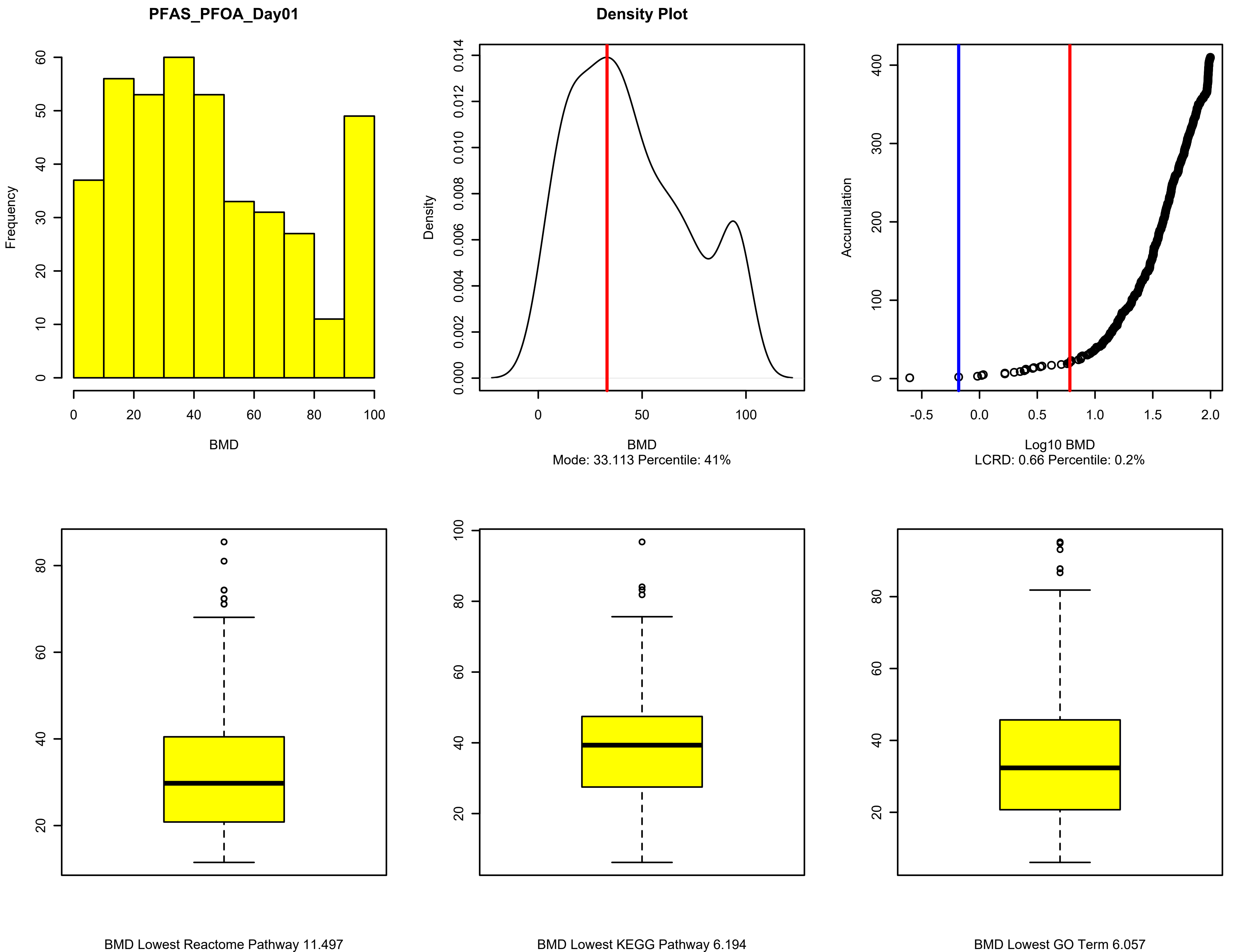

PFAS_PFOA_Day01
Frequency
BMD
Density Plot
Density
BMD
Mode: 33.113 Percentile: 41%
Accumulation
Log10 BMD
LCRD: 0.66 Percentile: 0.2%
BMD Lowest Reactome Pathway 11.497
BMD Lowest KEGG Pathway 6.194
BMD Lowest GO Term 6.057

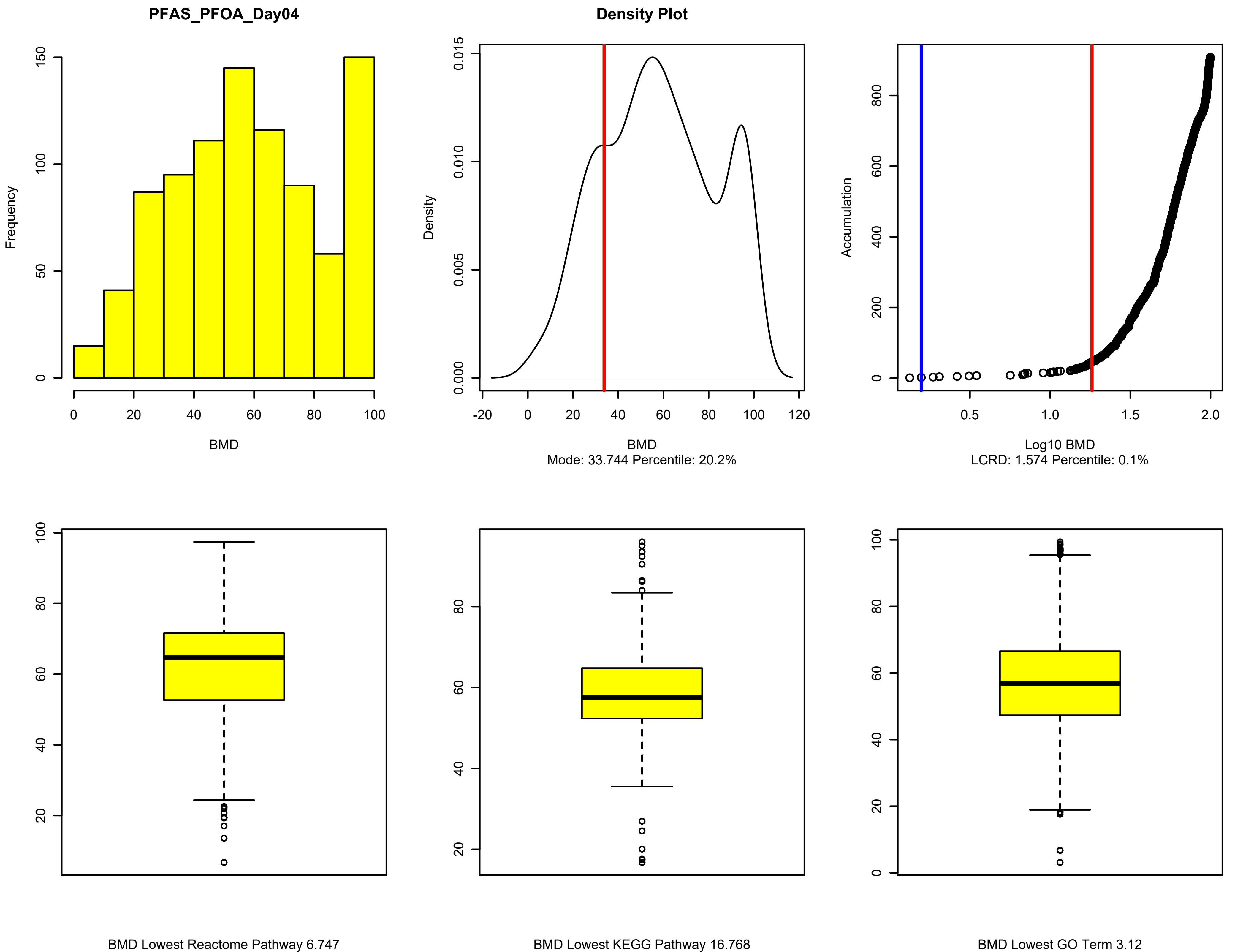

PFAS_PFOA_Day04
Frequency
BMD
Density Plot
Density
BMD
Mode: 33.744 Percentile: 20.2%
Accumulation
Log10 BMD
LCRD: 1.574 Percentile: 0.1%
BMD Lowest Reactome Pathway 6.747
BMD Lowest KEGG Pathway 16.768
BMD Lowest GO Term 3.12

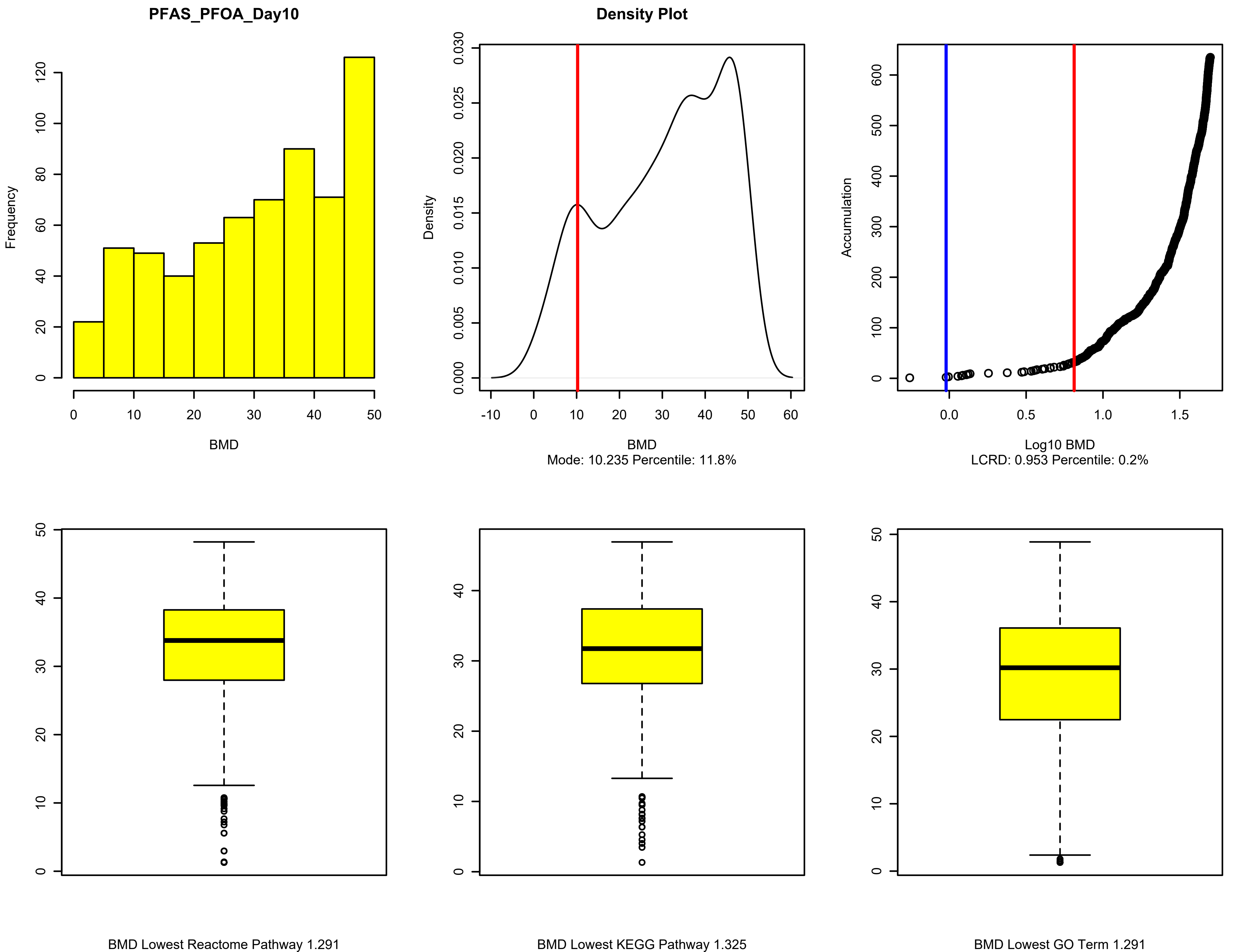
PFAS_PFOA_Day10
Frequency
BMD
Density Plot
Density
BMD
Mode: 10.235 Percentile: 11.8%
Accumulation
Log10 BMD
LCRD: 0.953 Percentile: 0.2%
BMD Lowest Reactome Pathway 1.291
BMD Lowest KEGG Pathway 1.325
BMD Lowest GO Term 1.291

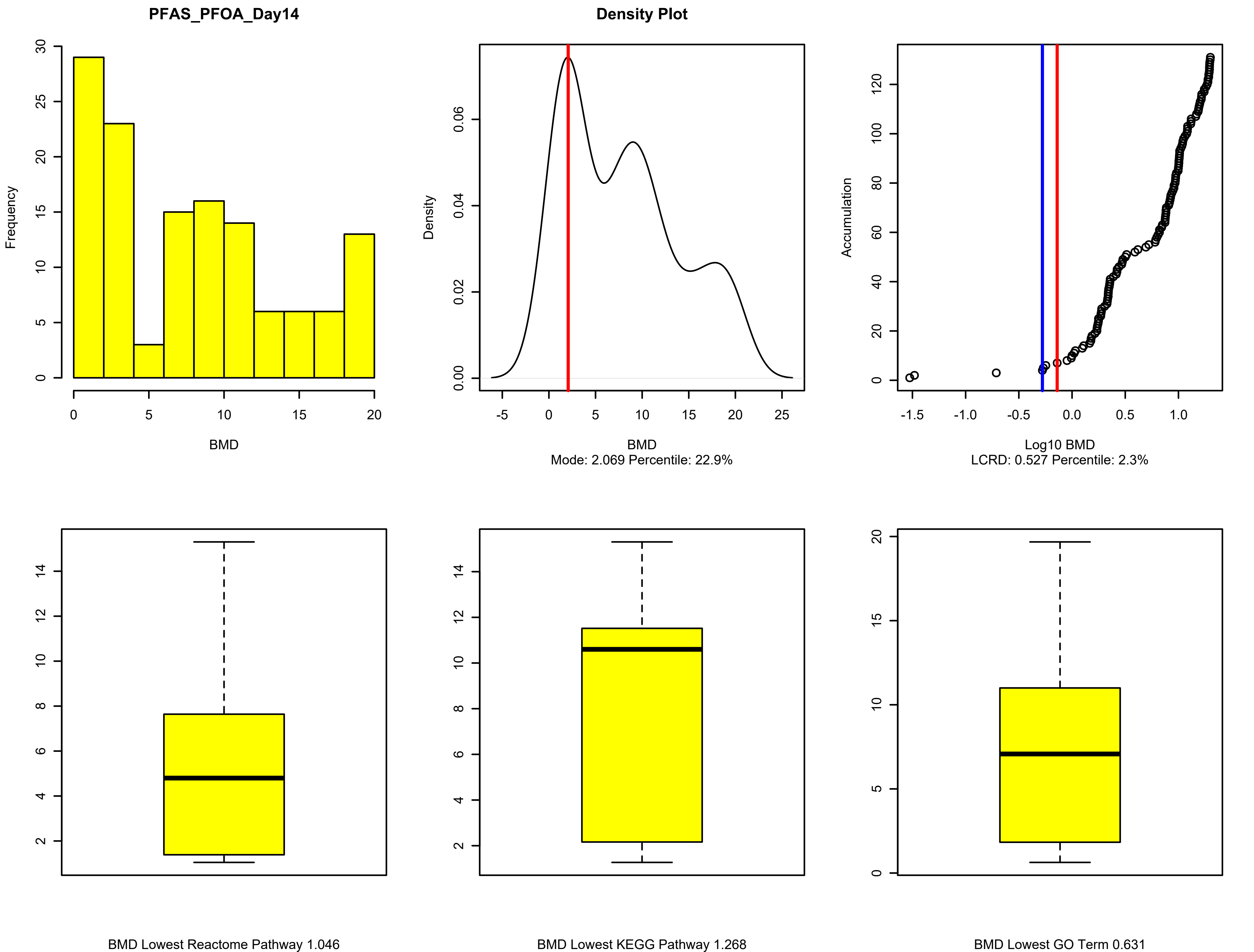
PFAS_PFOA_Day14
Frequency
BMD
Density Plot
Density
BMD
Mode: 2.069 Percentile: 22.9%
Accumulation
Log10 BMD
LCRD: 0.527 Percentile: 2.3%
BMD Lowest Reactome Pathway 1.046
BMD Lowest KEGG Pathway 1.268
BMD Lowest GO Term 0.631

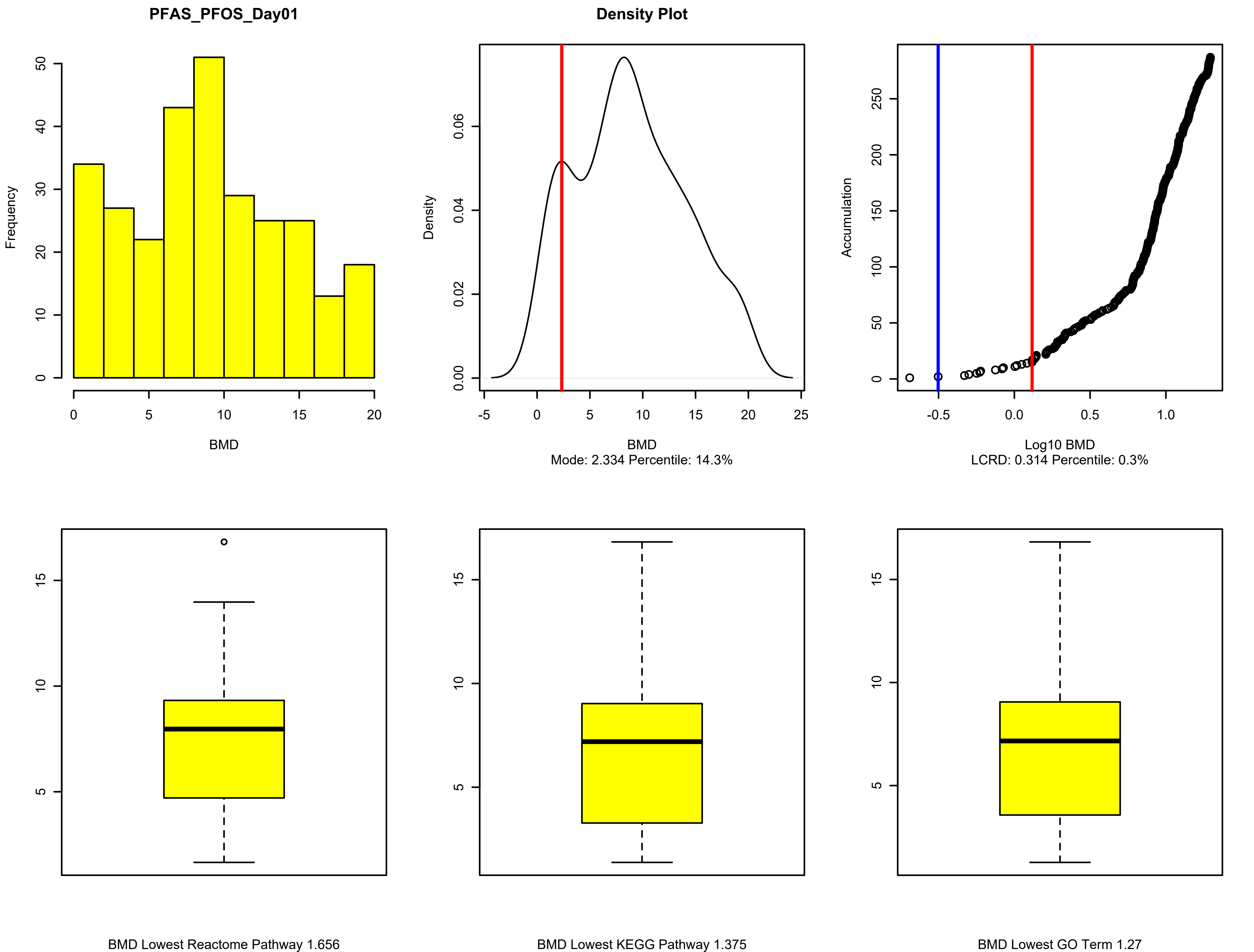

PFAS_PFOS_Day01
Frequency
BMD
Density Plot
Density
BMD
Mode: 2.334 Percentile: 14.3%
Accumulation
Log10 BMD
LCRD: 0.314 Percentile: 0.3%
BMD Lowest Reactome Pathway 1.656
BMD Lowest KEGG Pathway 1.375
BMD Lowest GO Term 1.27

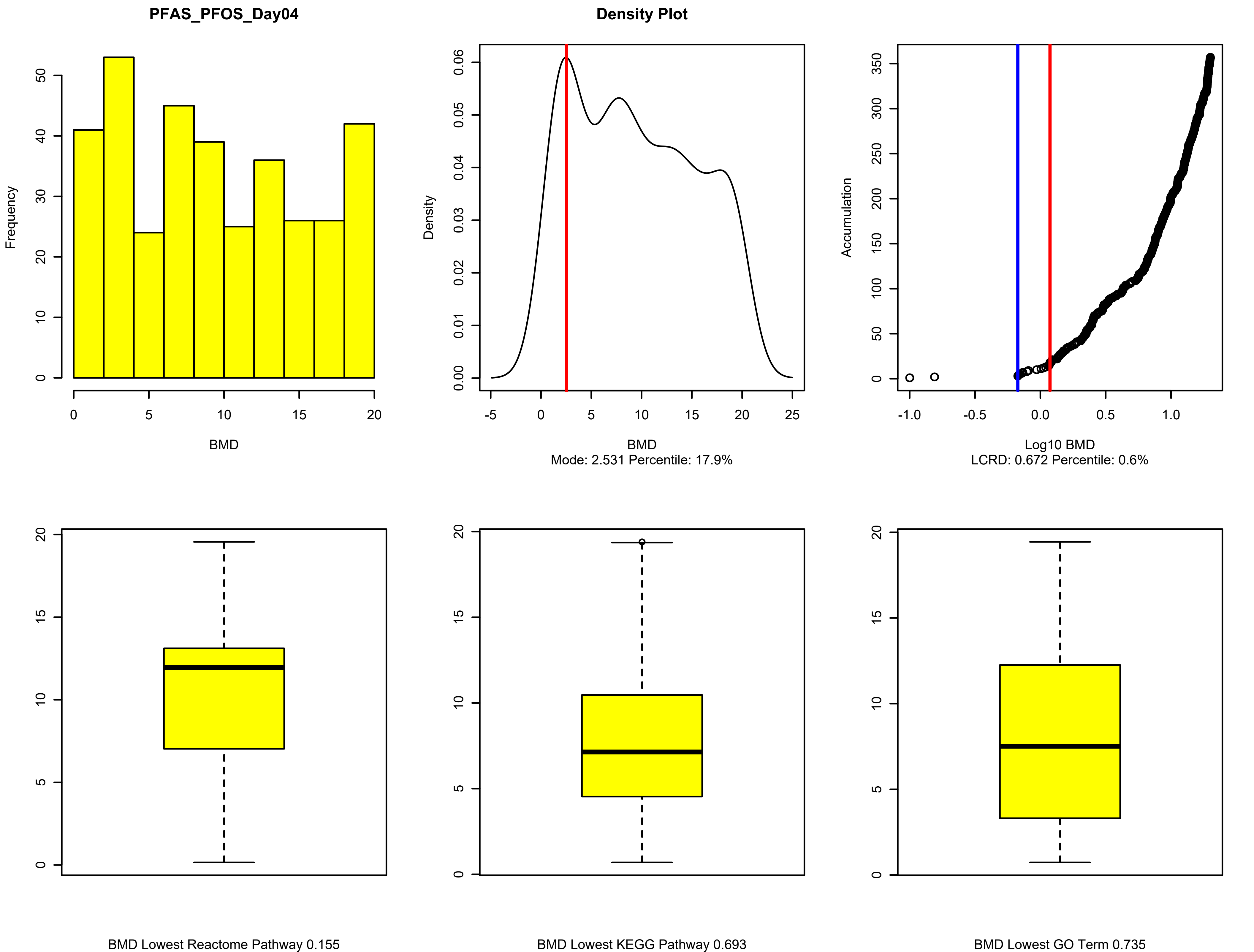

PFAS_PFOS_Day04
Frequency
BMD
Density Plot
Density
BMD
Mode: 2.531 Percentile: 17.9%
Accumulation
Log10 BMD
LCRD: 0.672 Percentile: 0.6%
BMD Lowest Reactome Pathway 0.155
BMD Lowest KEGG Pathway 0.693
BMD Lowest GO Term 0.735

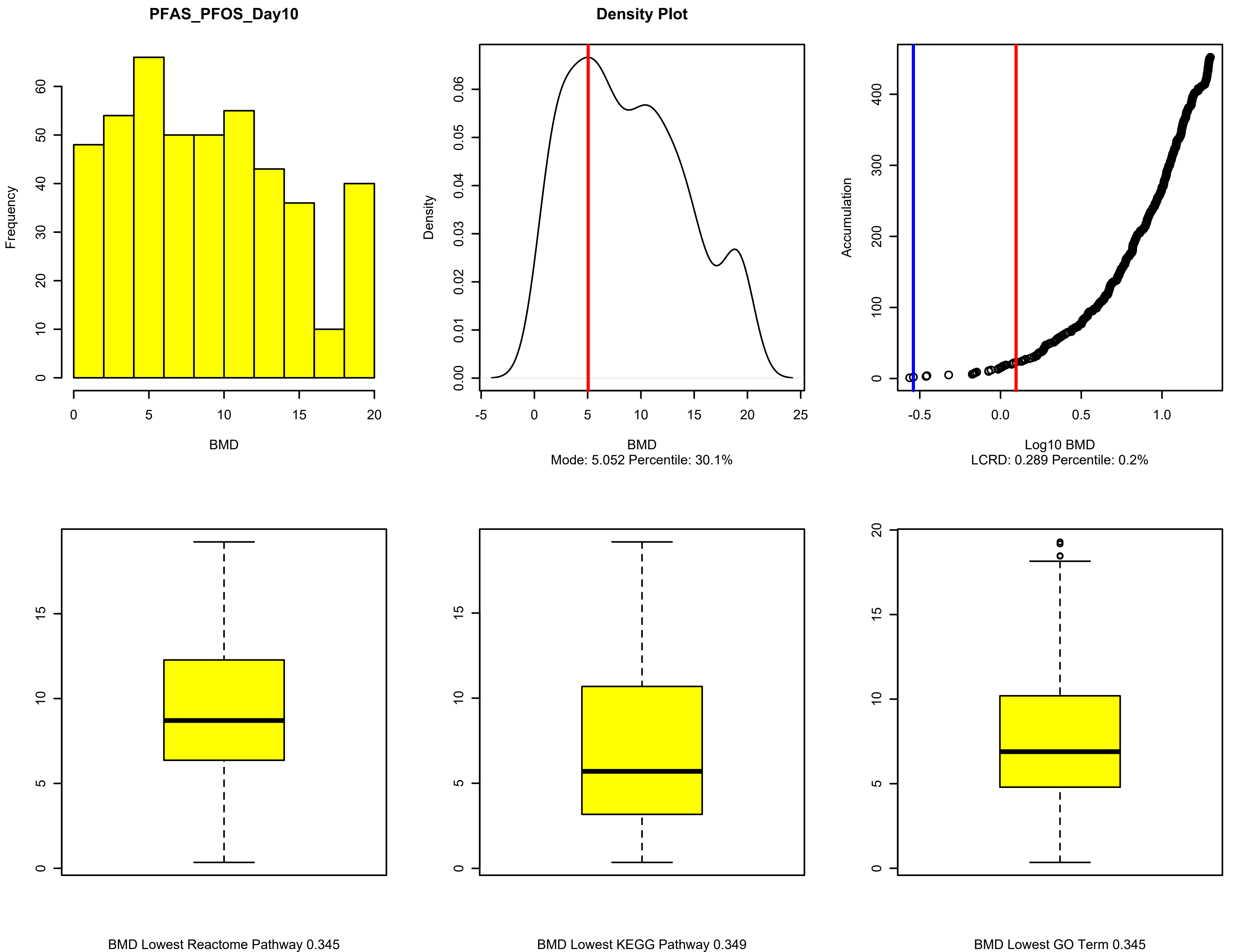

PFAS_PFOS_Day10
Frequency
BMD
Density Plot
Density
BMD
Mode: 5.052 Percentile: 30.1%
Accumulation
Log10 BMD
LCRD: 0.289 Percentile: 0.2%
BMD Lowest Reactome Pathway 0.345
BMD Lowest KEGG Pathway 0.349
BMD Lowest GO Term 0.345

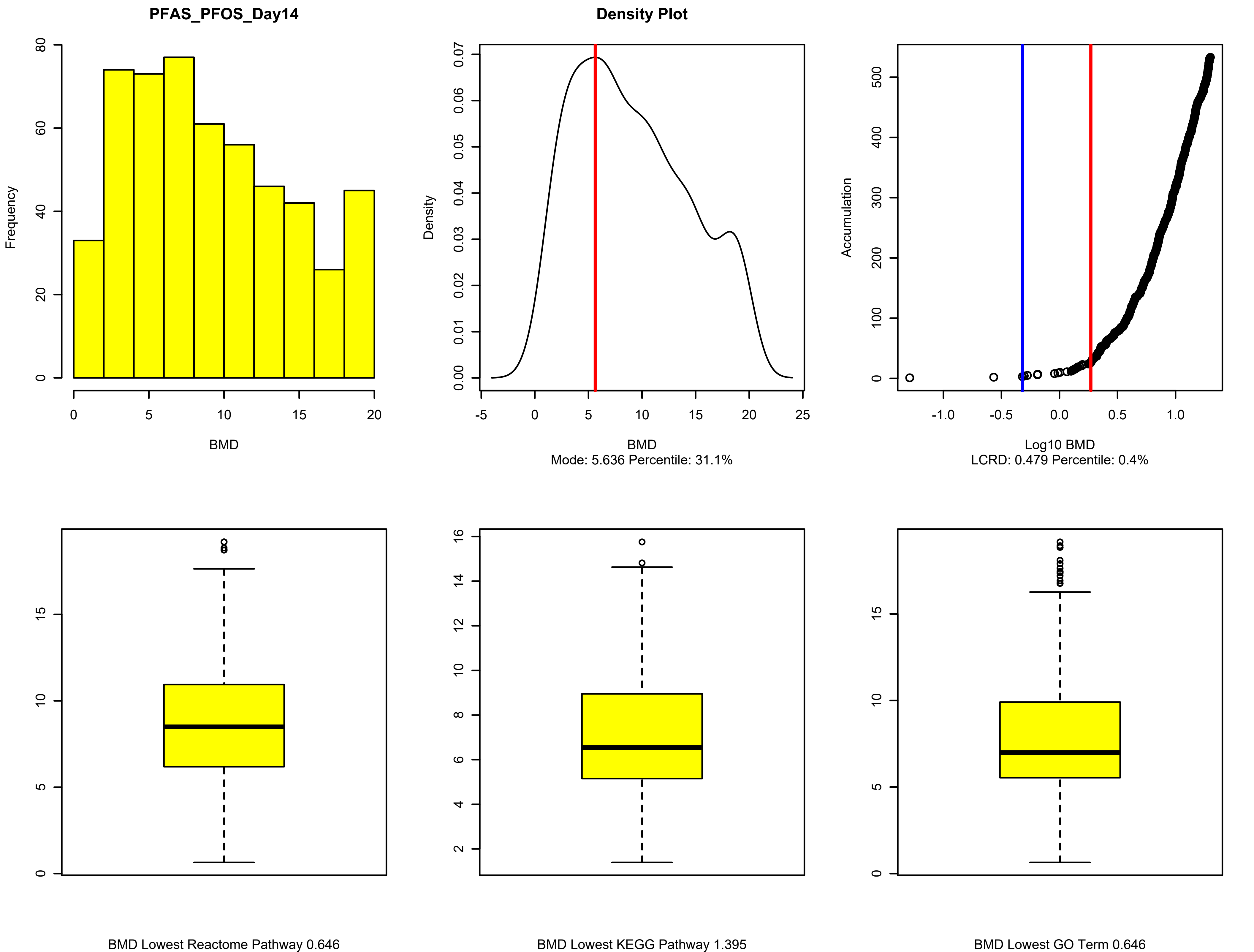

PFAS_PFOS_Day14
Frequency
BMD
Density Plot
Density
BMD
Mode: 5.636 Percentile: 31.1%
Accumulation
Log10 BMD
LCRD: 0.479 Percentile: 0.4%
BMD Lowest Reactome Pathway 0.646
BMD Lowest KEGG Pathway 1.395
BMD Lowest GO Term 0.646

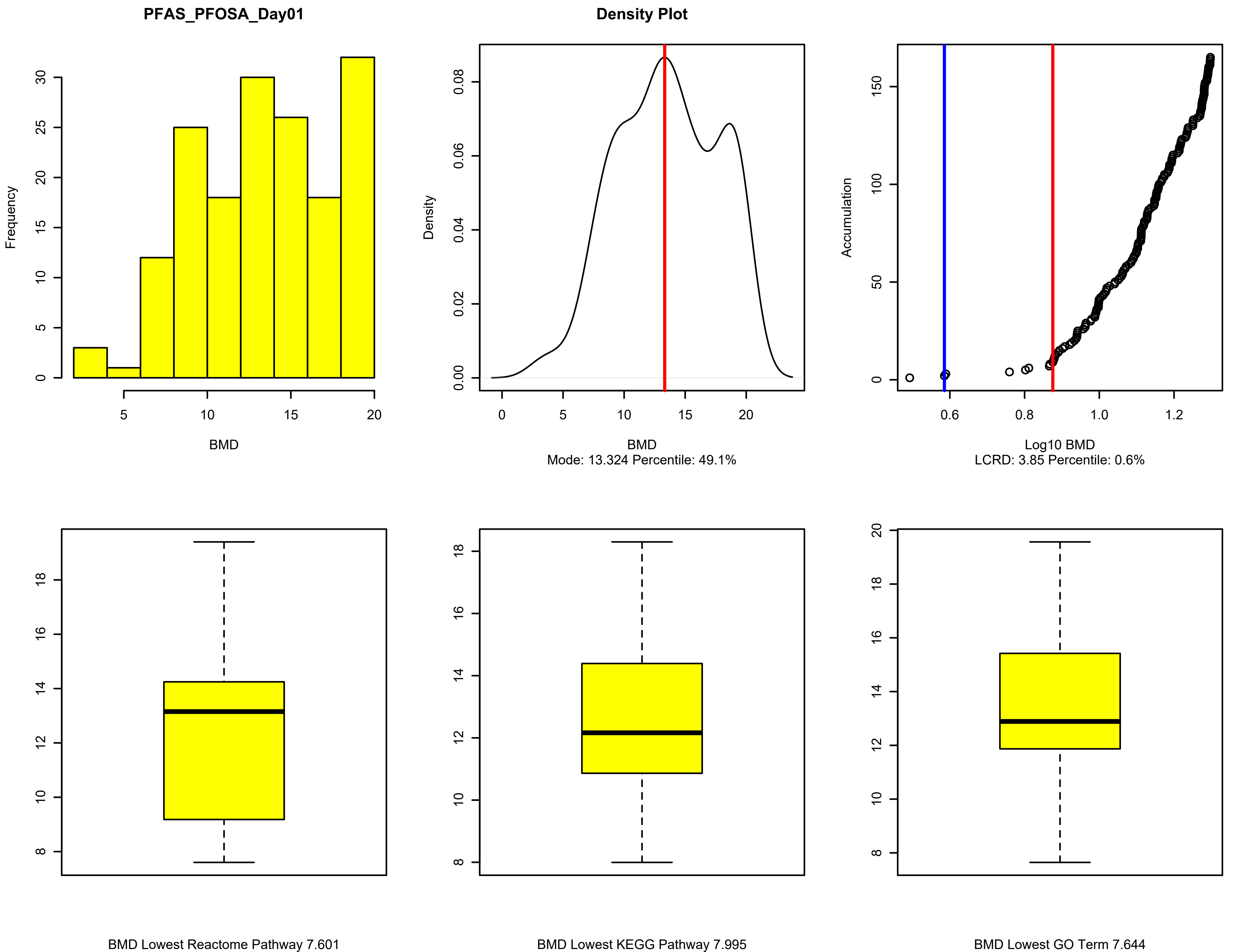
PFAS_PFOSA_Day01
Frequency
BMD
Density Plot
Density
BMD
Mode: 13.324 Percentile: 49.1%
Accumulation
Log10 BMD
LCRD: 3.85 Percentile: 0.6%
BMD Lowest Reactome Pathway 7.601
BMD Lowest KEGG Pathway 7.995
BMD Lowest GO Term 7.644

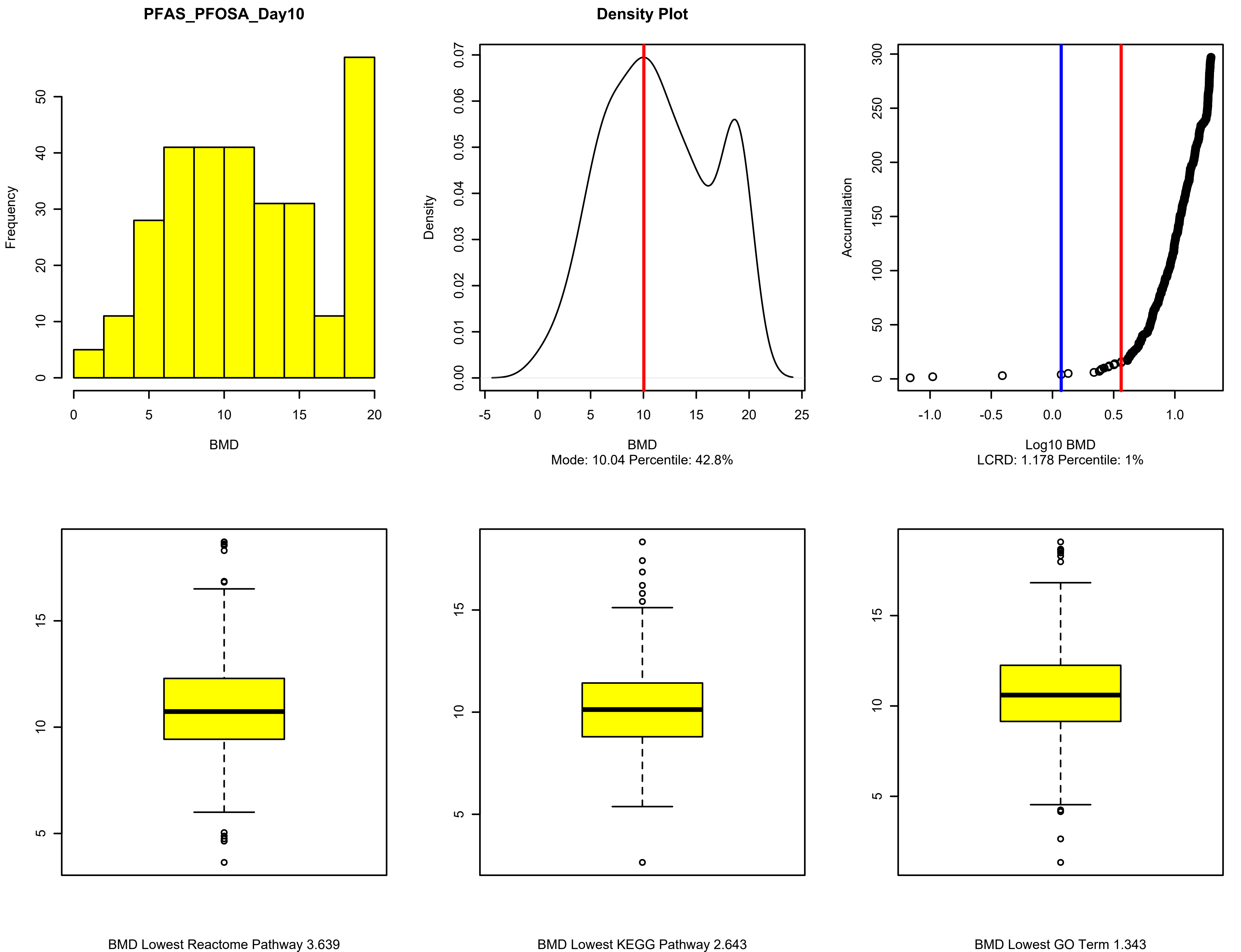
PFAS_PFOSA_Day10
Frequency
BMD
Density Plot
Density
BMD
Mode: 10.04 Percentile: 42.8%
Accumulation
Log10 BMD
LCRD: 1.178 Percentile: 1%
BMD Lowest Reactome Pathway 3.639
BMD Lowest KEGG Pathway 2.643
BMD Lowest GO Term 1.343

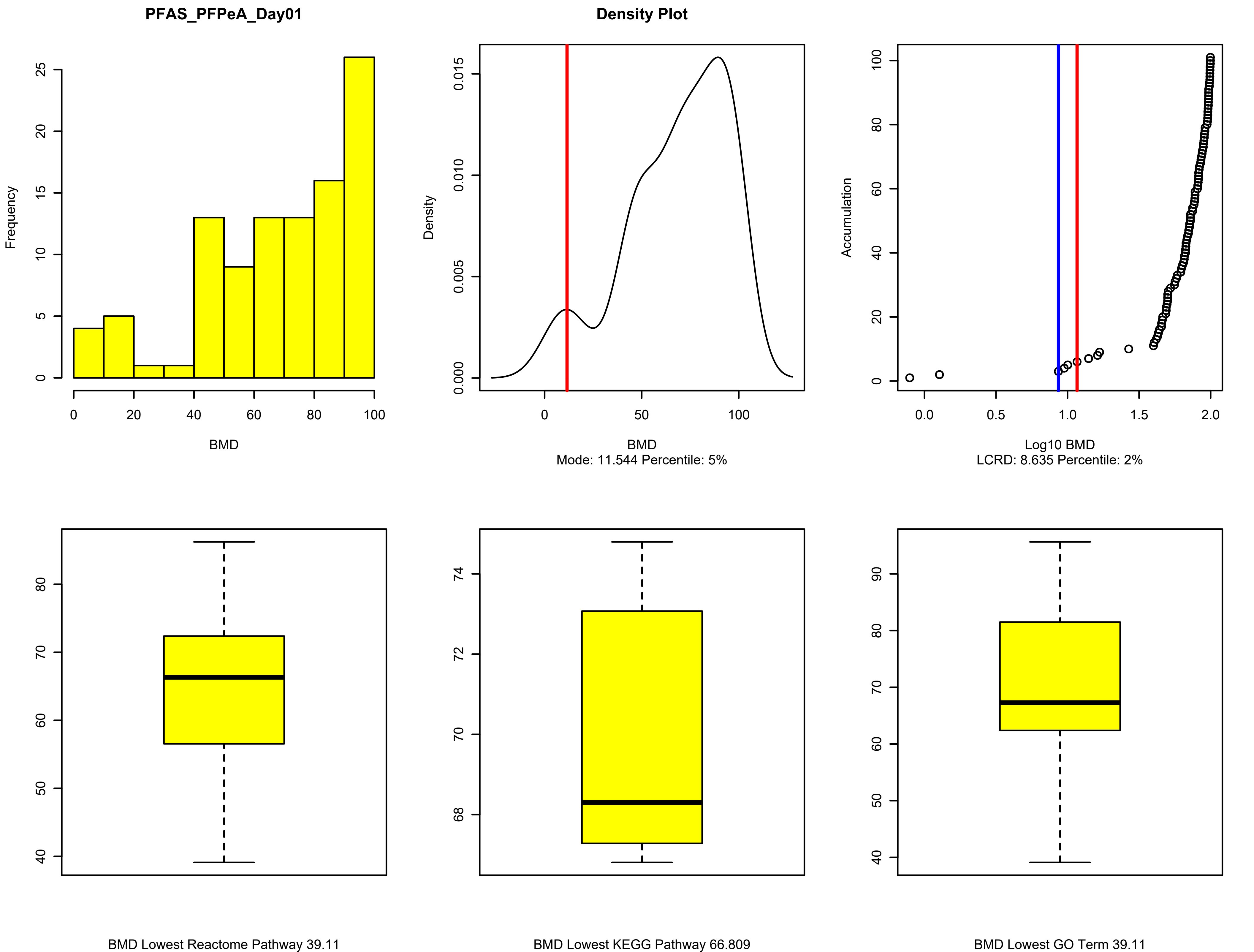

PFAS_PFPeA_Day01
Frequency
BMD
Density Plot
Density
BMD
Mode: 11.544 Percentile: 5%
Accumulation
Log10 BMD
LCRD: 8.635 Percentile: 2%
BMD Lowest Reactome Pathway 39.11
BMD Lowest KEGG Pathway 66.809
BMD Lowest GO Term 39.11

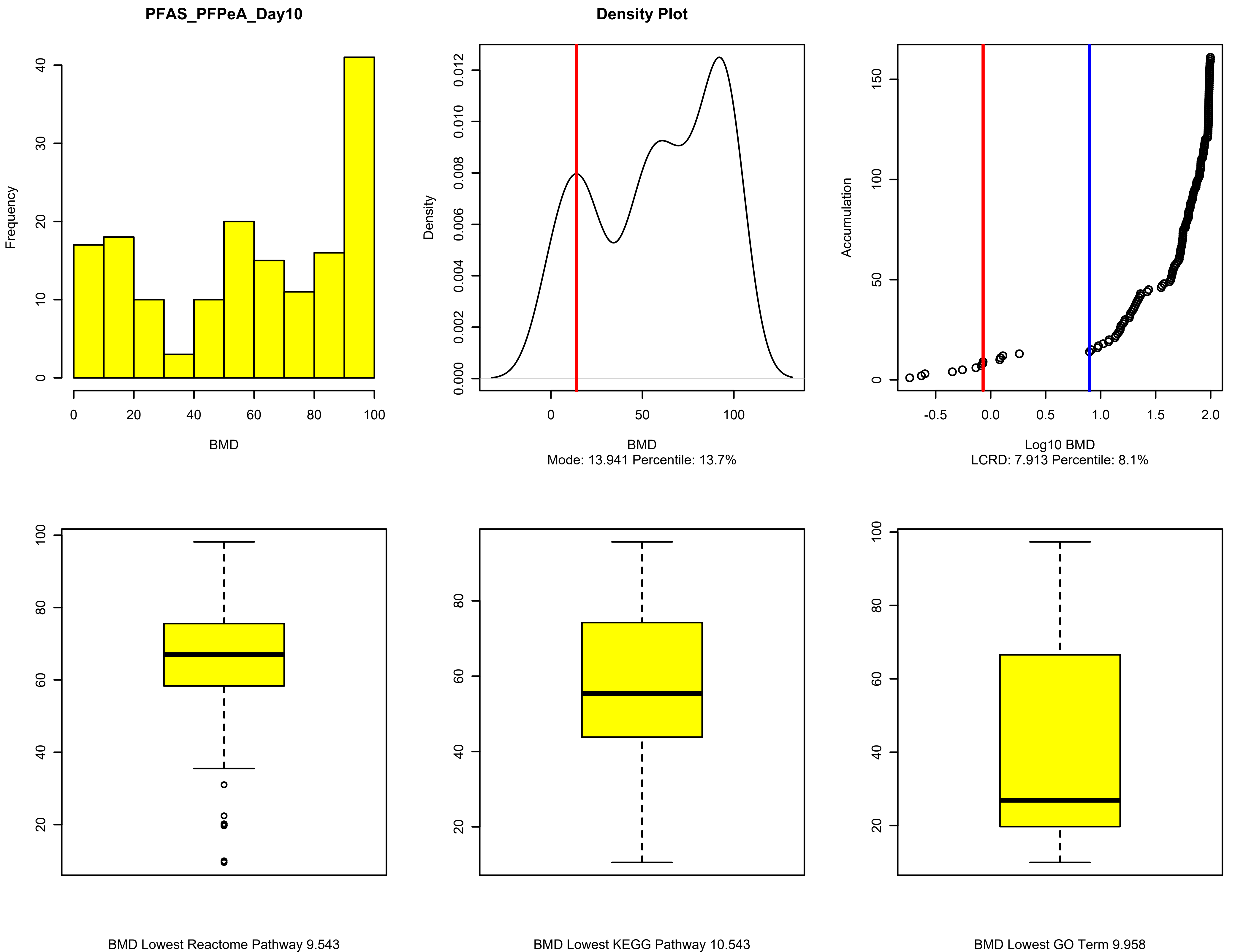

PFAS_PFPeA_Day10
Frequency
BMD
Density Plot
Density
BMD
Mode: 13.941 Percentile: 13.7%
Accumulation
Log10 BMD
LCRD: 7.913 Percentile: 8.1%
BMD Lowest Reactome Pathway 9.543
BMD Lowest KEGG Pathway 10.543
BMD Lowest GO Term 9.958

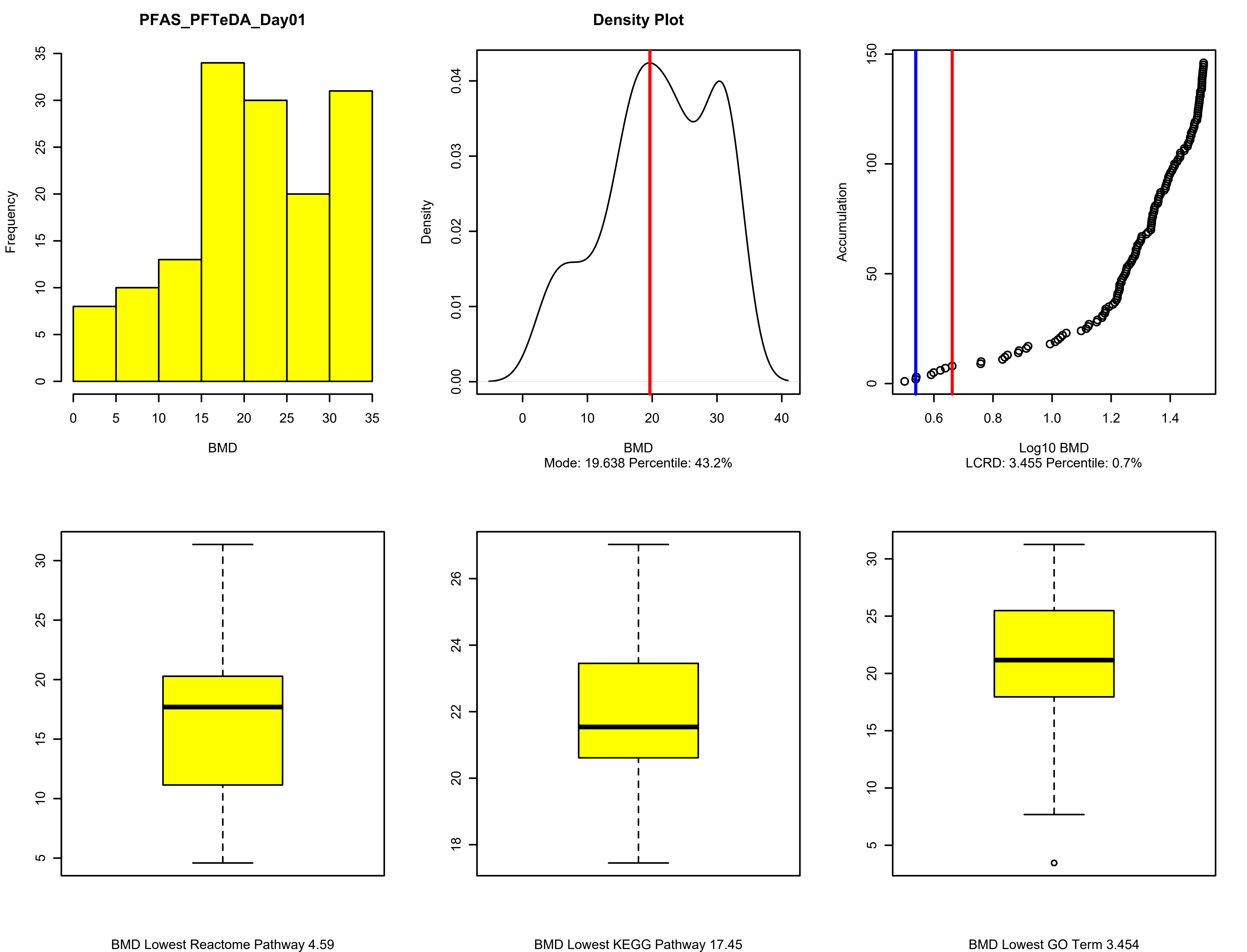

PFAS_PFTeDA_Day01
Frequency
BMD
Density Plot
Density
BMD
Mode: 19.638 Percentile: 43.2%
Accumulation
Log10 BMD
LCRD: 3.455 Percentile: 0.7%
BMD Lowest Reactome Pathway 4.59
BMD Lowest KEGG Pathway 17.45
BMD Lowest GO Term 3.454

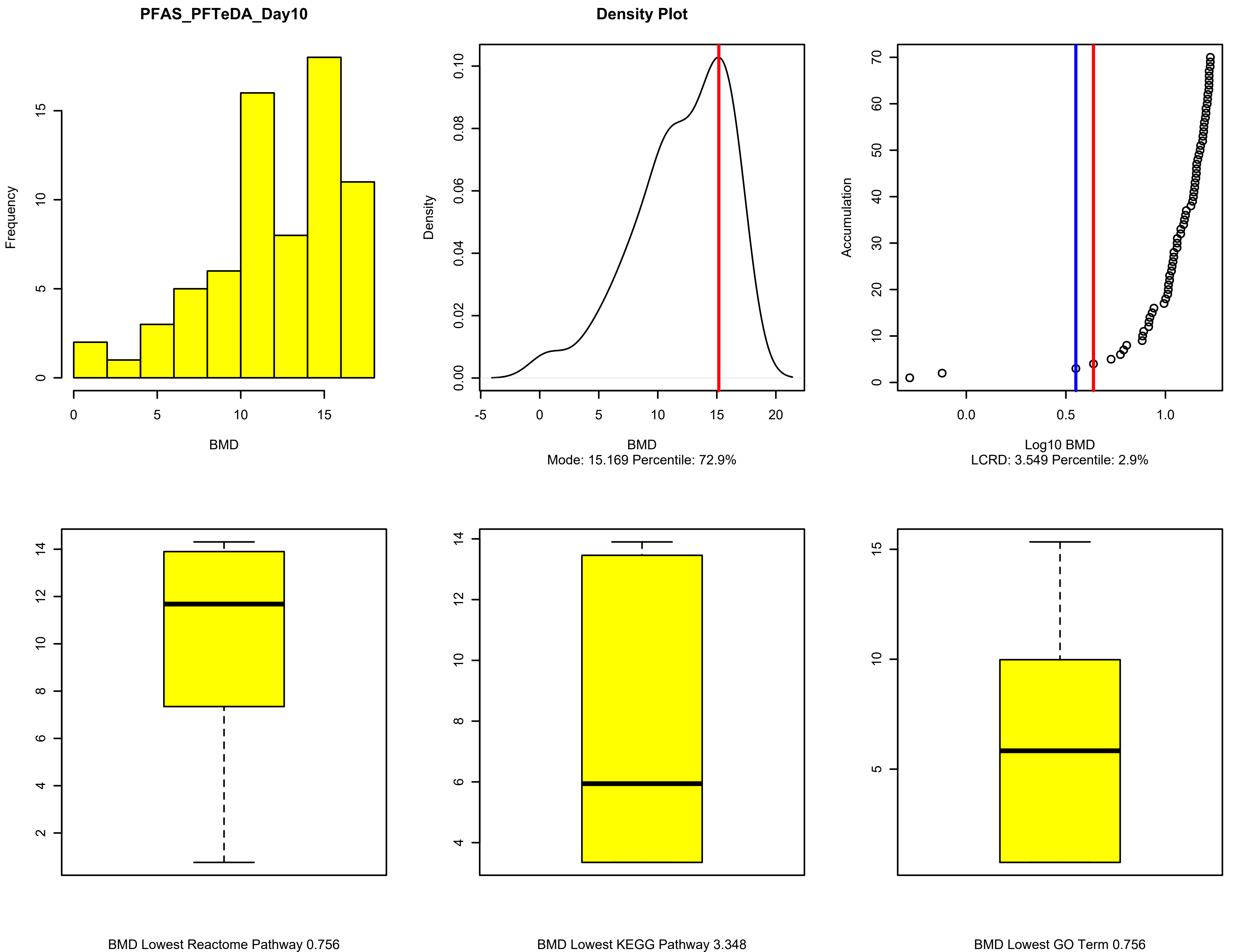

PFAS_PFTeDA_Day10
Frequency
BMD
Density Plot
Density
BMD
Mode: 15.169 Percentile: 72.9%
Accumulation
Log10 BMD
LCRD: 3.549 Percentile: 2.9%
BMD Lowest Reactome Pathway 0.756
BMD Lowest KEGG Pathway 3.348
BMD Lowest GO Term 0.756

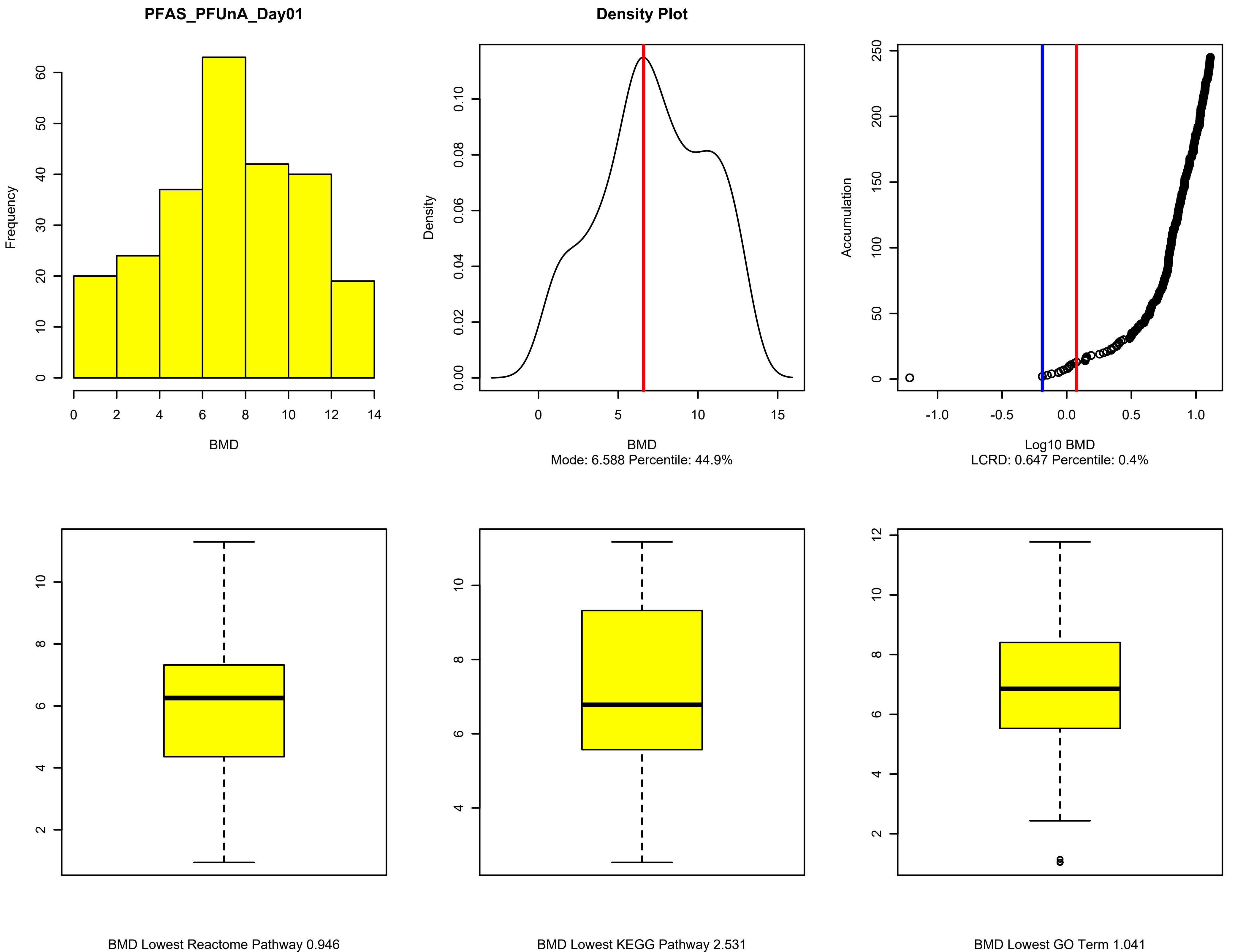
PFAS_PFUnA_Day01
Frequency
BMD
Density Plot
Density
BMD
Mode: 6.588 Percentile: 44.9%
Accumulation
Log10 BMD
LCRD: 0.647 Percentile: 0.4%
BMD Lowest Reactome Pathway 0.946
BMD Lowest KEGG Pathway 2.531
BMD Lowest GO Term 1.041

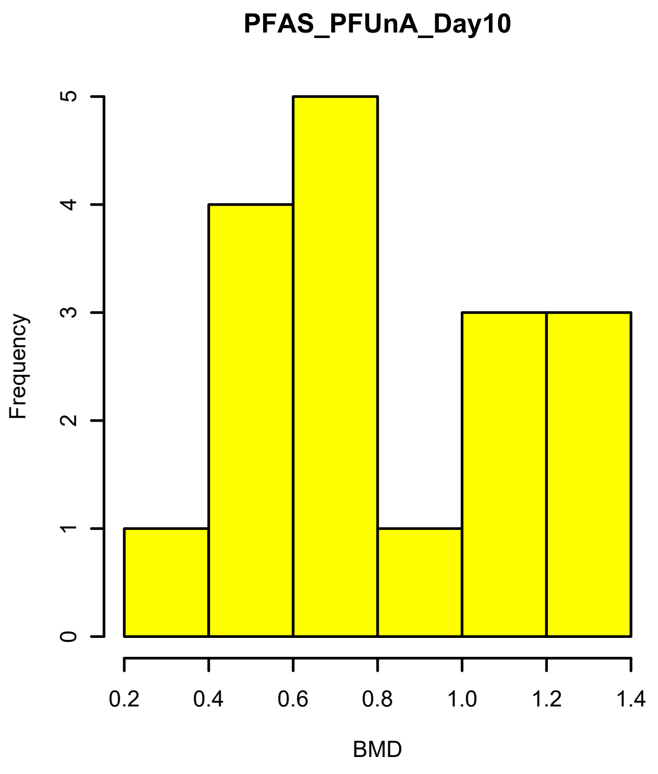
PFAS_PFUnA_Day10
Frequency
BMD

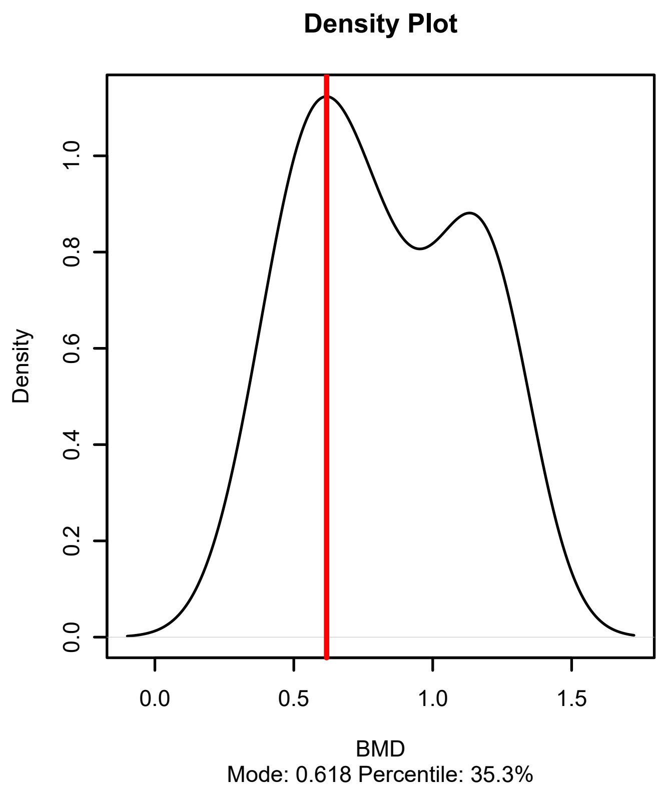
Density Plot
Density
BMD
Mode: 0.618 Percentile: 35.3%

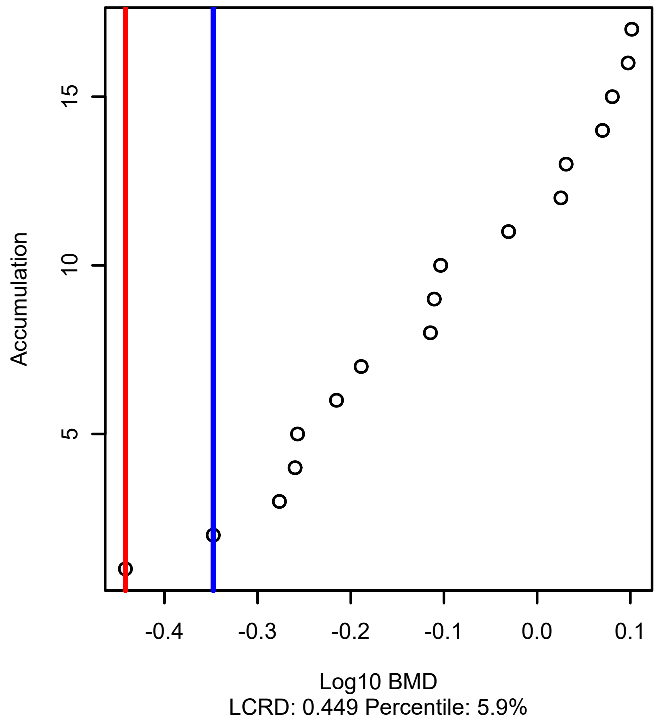
Accumulation
Log10 BMD
LCRD: 0.449 Percentile: 5.9%

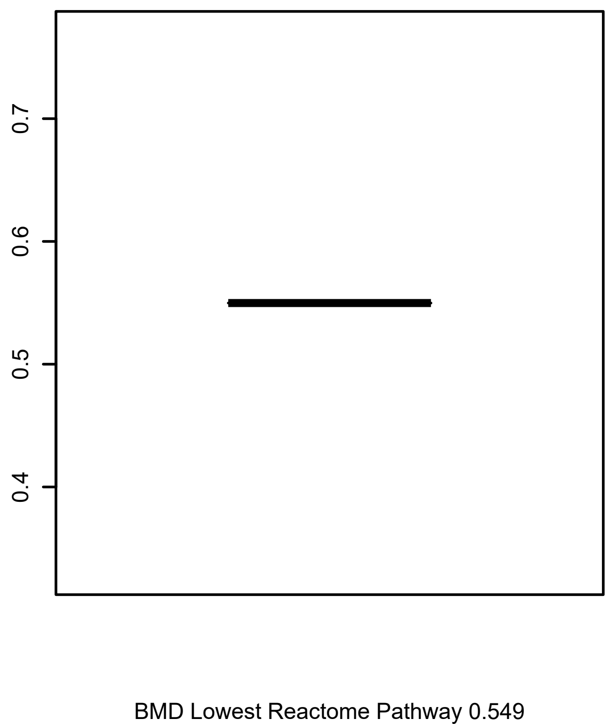
BMD Lowest Reactome Pathway 0.549

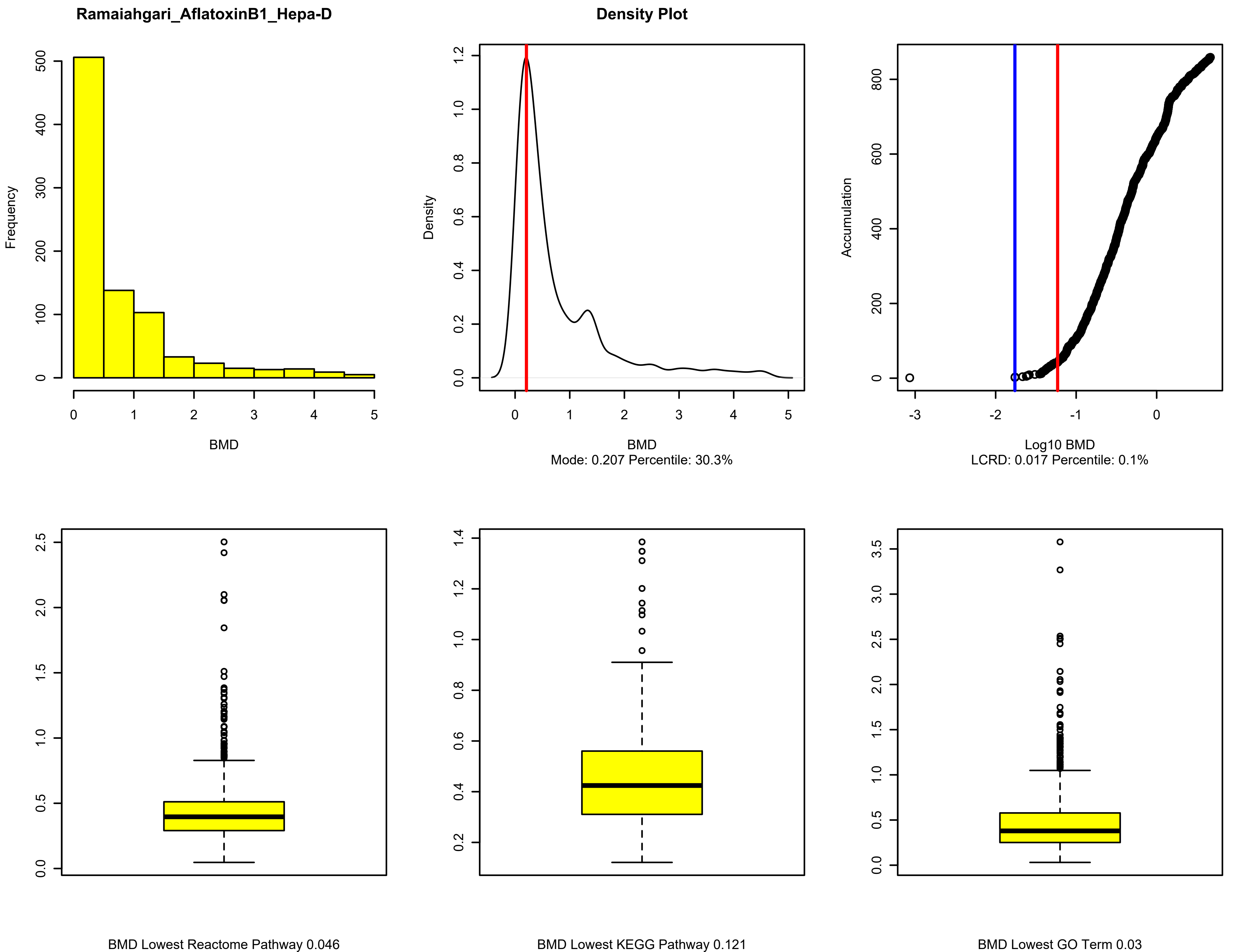
Ramaiahgari_AflatoxinB1_Hepa-D
Frequency
BMD
Density Plot
Density
BMD
Mode: 0.207 Percentile: 30.3%
Accumulation
Log10 BMD
LCRD: 0.017 Percentile: 0.1%
BMD Lowest Reactome Pathway 0.046
BMD Lowest KEGG Pathway 0.121
BMD Lowest GO Term 0.03

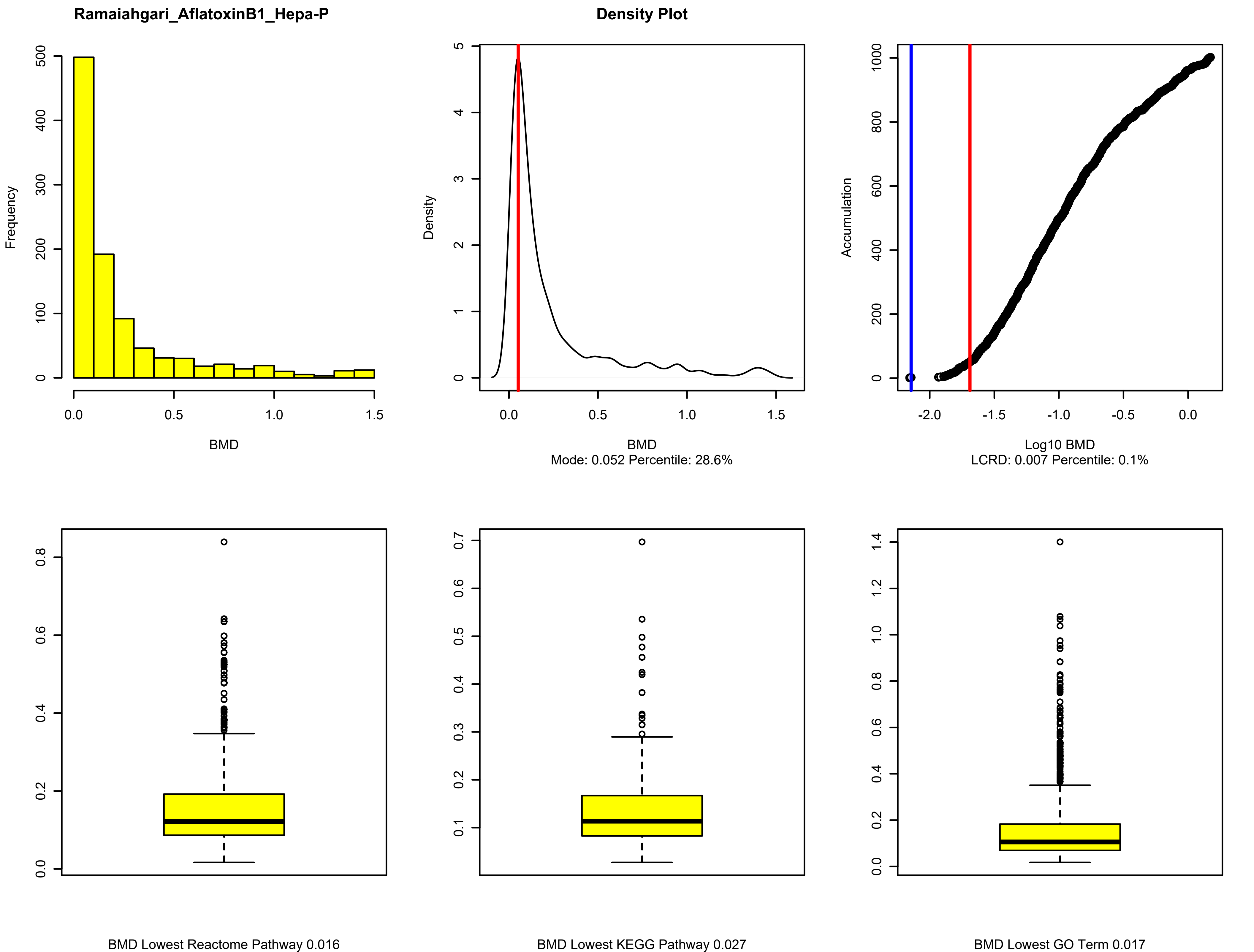

Ramaiahgari_AflatoxinB1_Hepa-P
Frequency
BMD
Density Plot
Density
BMD
Mode: 0.052 Percentile: 28.6%
Accumulation
Log10 BMD
LCRD: 0.007 Percentile: 0.1%
BMD Lowest Reactome Pathway 0.016
BMD Lowest KEGG Pathway 0.027
BMD Lowest GO Term 0.017

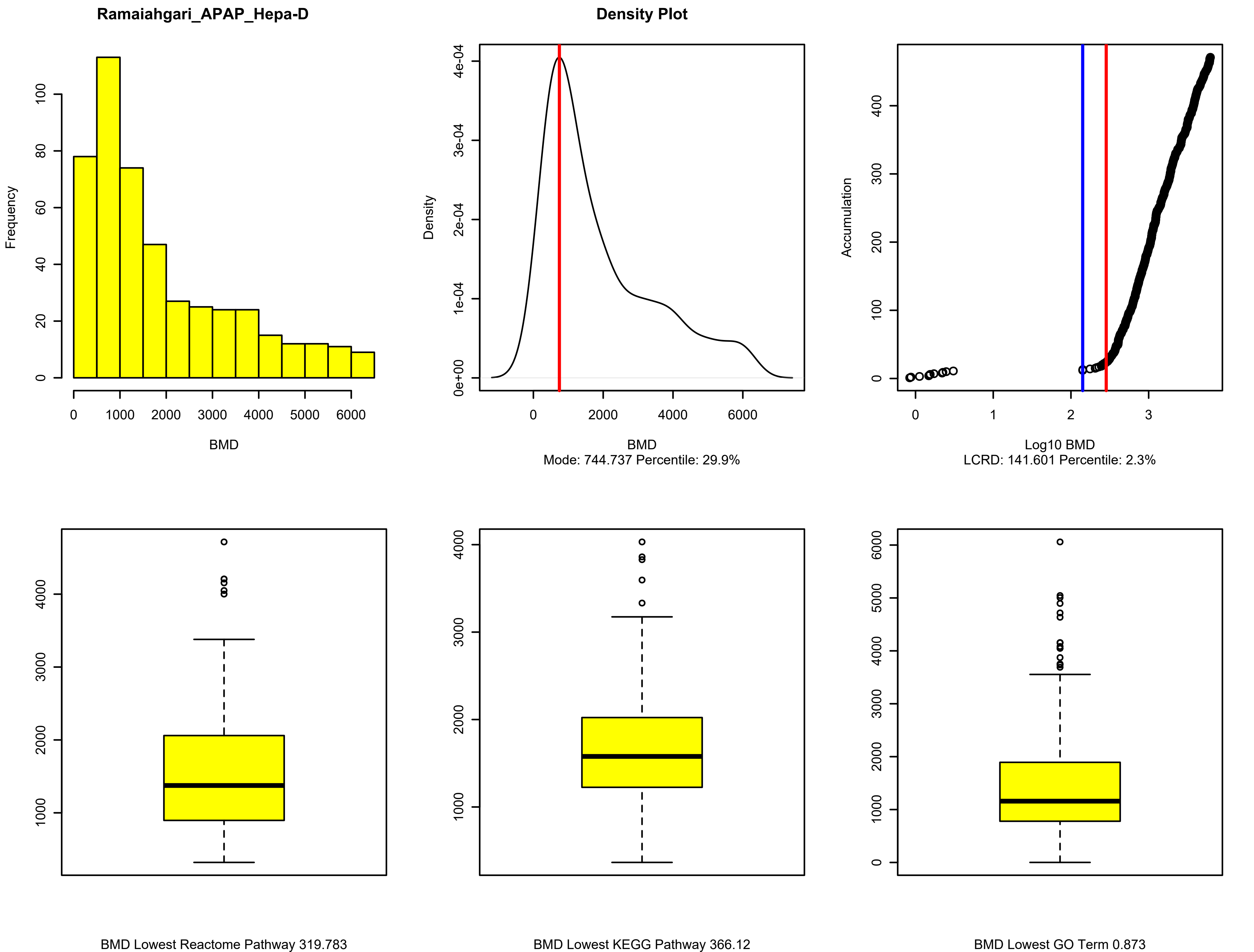

Ramaiahgari_APAP_Hepa-D
Frequency
BMD
0
20
40
60
80
100
1000
2000
3000
4000
5000
6000
Density Plot
Density
0e+00
1e-04
2e-04
3e-04
4e-04
BMD
Mode: 744.737 Percentile: 29.9%
Accumulation
Log10 BMD
LCRD: 141.601 Percentile: 2.3%
BMD Lowest Reactome Pathway 319.783
BMD Lowest KEGG Pathway 366.12
BMD Lowest GO Term 0.873

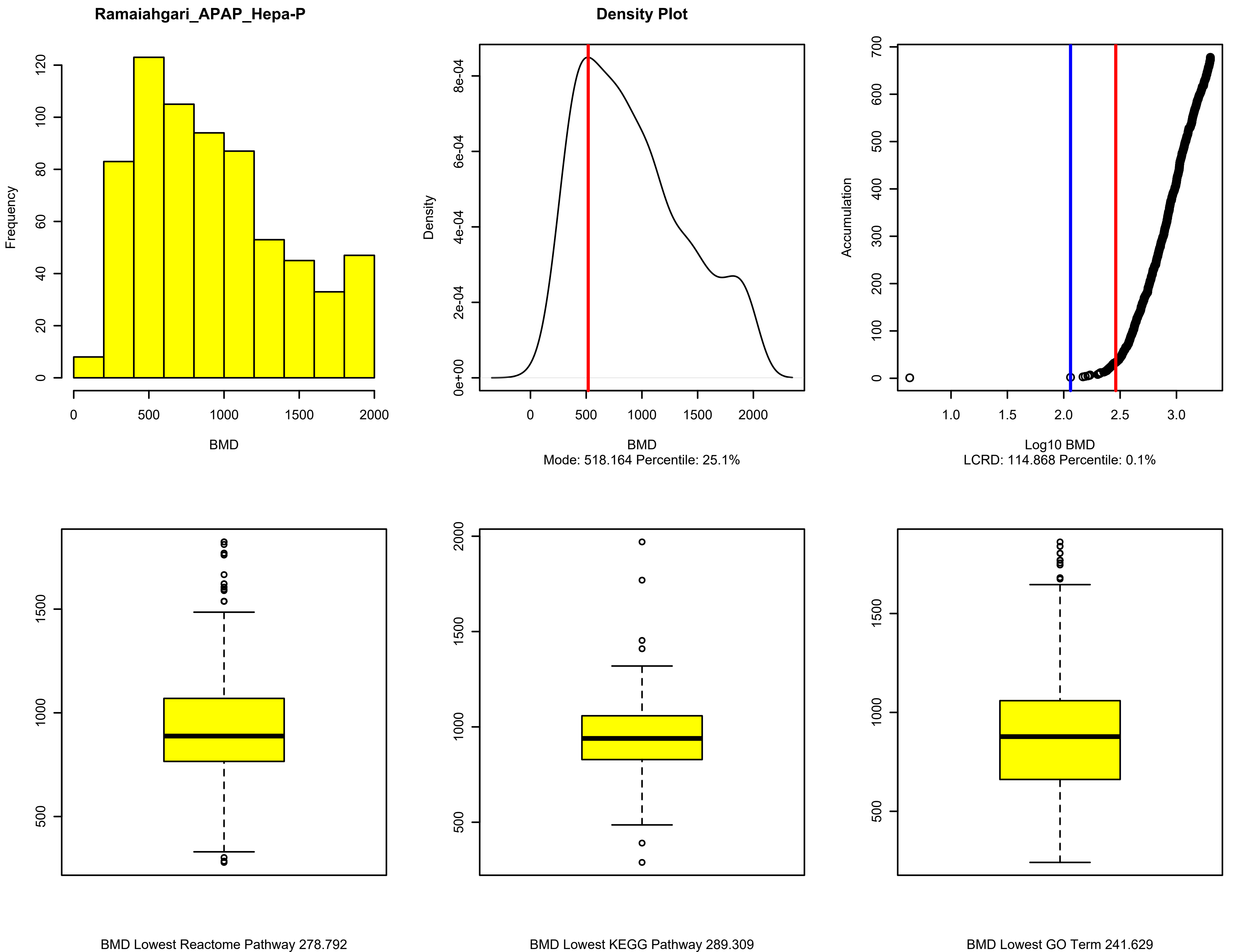

Ramaiahgari_APAP_Hepa-P
Frequency
BMD
Density Plot
Density
BMD
Mode: 518.164 Percentile: 25.1%
Accumulation
Log10 BMD
LCRD: 114.868 Percentile: 0.1%
BMD Lowest Reactome Pathway 278.792
BMD Lowest KEGG Pathway 289.309
BMD Lowest GO Term 241.629

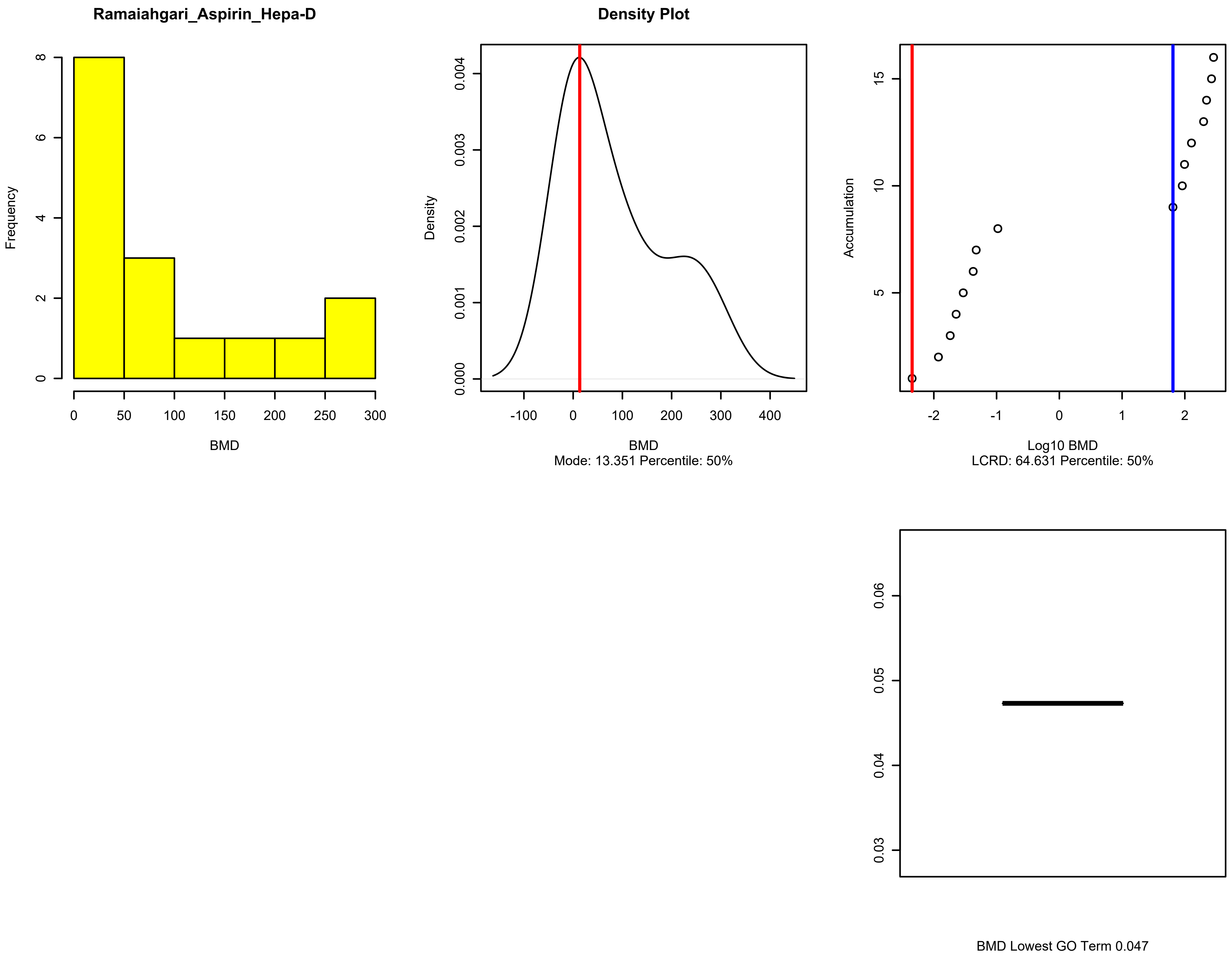
Ramaiahgari_Aspirin_Hepa-D
Frequency
BMD
Density Plot
Density
BMD
Mode: 13.351 Percentile: 50%
Accumulation
Log10 BMD
LCRD: 64.631 Percentile: 50%
BMD Lowest GO Term 0.047

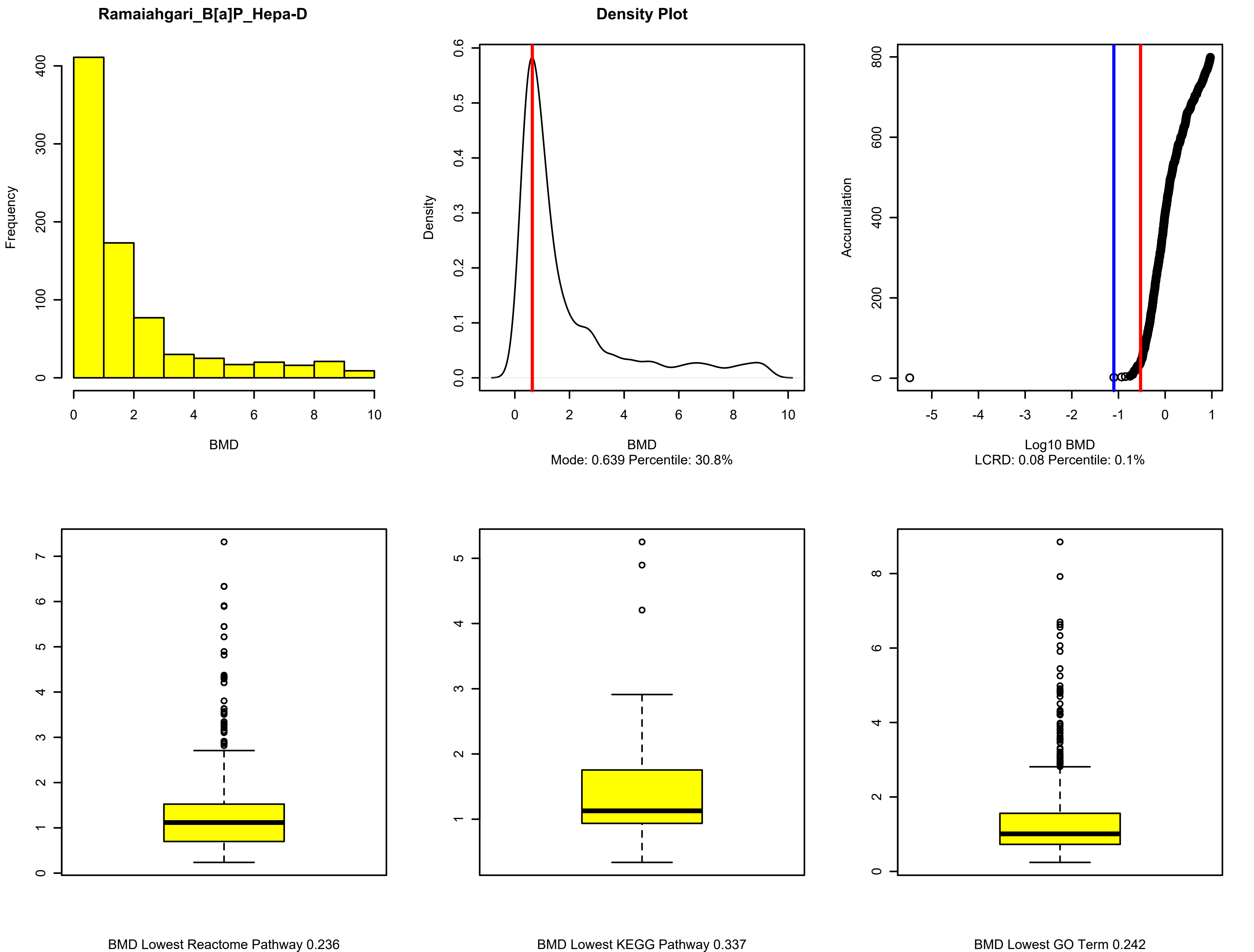
Ramaiahgari_B[a]P_Hepa-D
Frequency
BMD
Density Plot
Density
BMD
Mode: 0.639 Percentile: 30.8%
Accumulation
Log10 BMD
LCRD: 0.08 Percentile: 0.1%
BMD Lowest Reactome Pathway 0.236
BMD Lowest KEGG Pathway 0.337
BMD Lowest GO Term 0.242

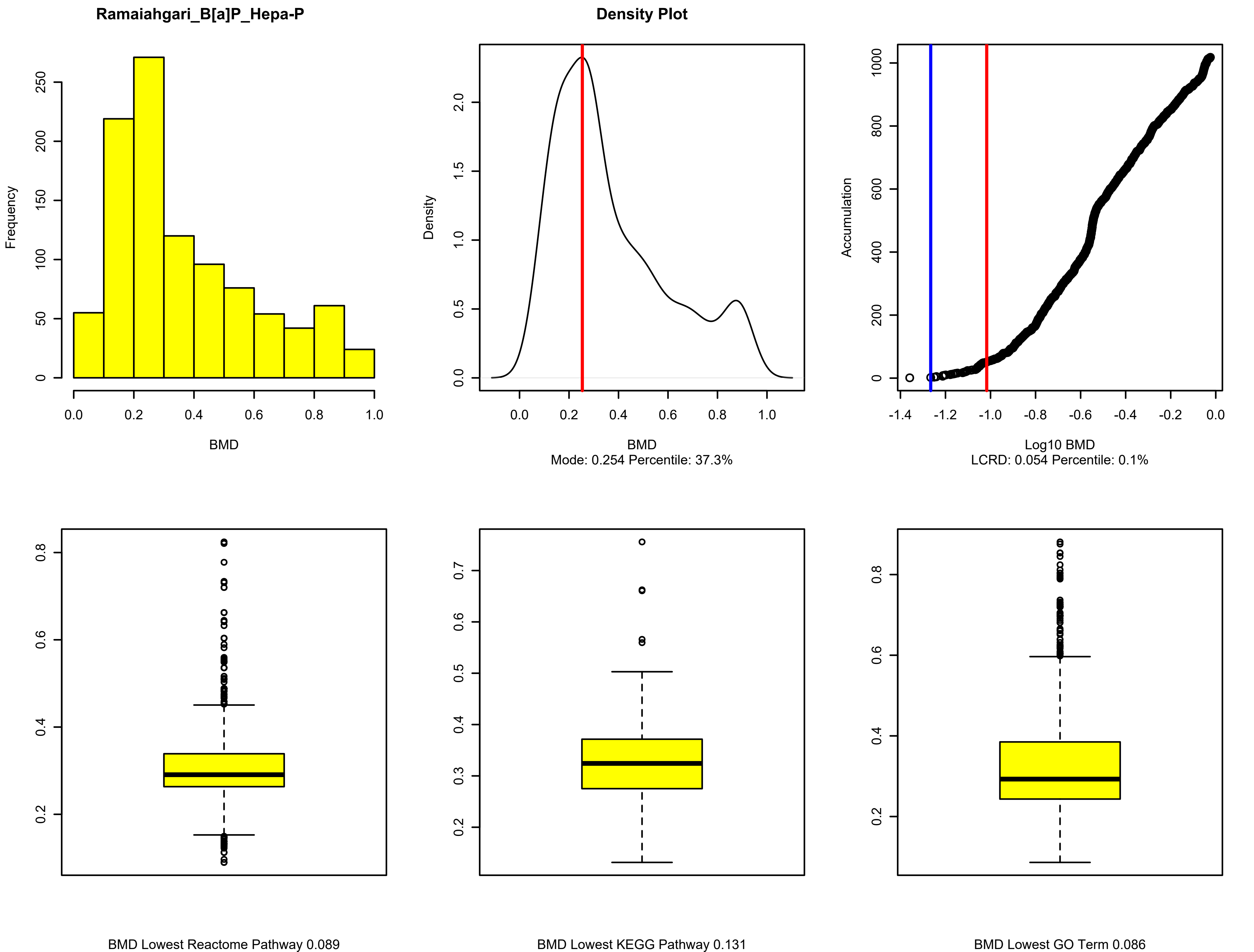

Ramaiahgari_B[a]P_Hepa-P
Frequency
BMD
Density Plot
Density
BMD
Mode: 0.254 Percentile: 37.3%
Accumulation
Log10 BMD
LCRD: 0.054 Percentile: 0.1%
BMD Lowest Reactome Pathway 0.089
BMD Lowest KEGG Pathway 0.131
BMD Lowest GO Term 0.086

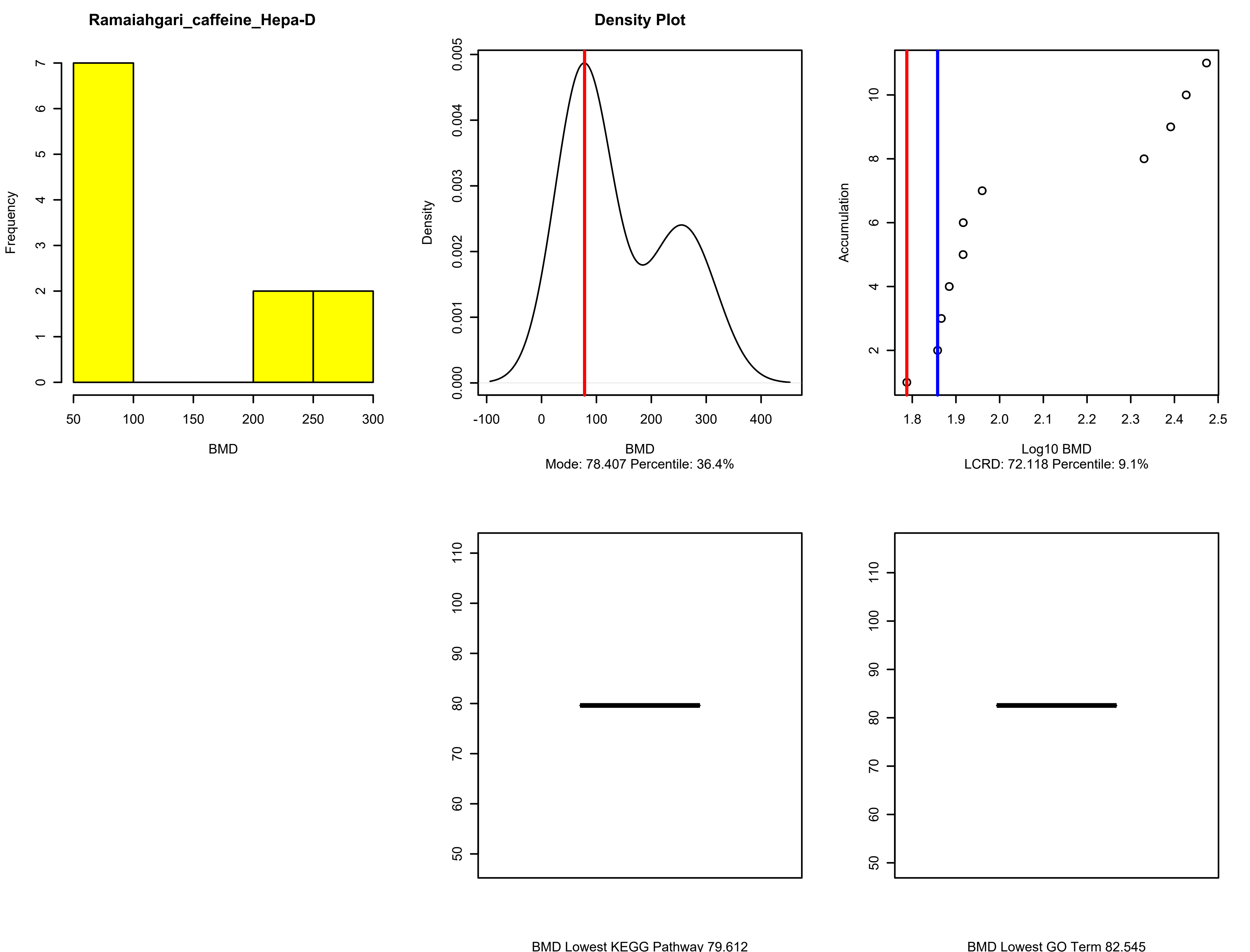

Ramaiahgari_caffeine_Hepa-D
Frequency
BMD
Density Plot
Density
BMD
Mode: 78.407 Percentile: 36.4%
Accumulation
Log10 BMD
LCRD: 72.118 Percentile: 9.1%
BMD Lowest KEGG Pathway 79.612
BMD Lowest GO Term 82.545

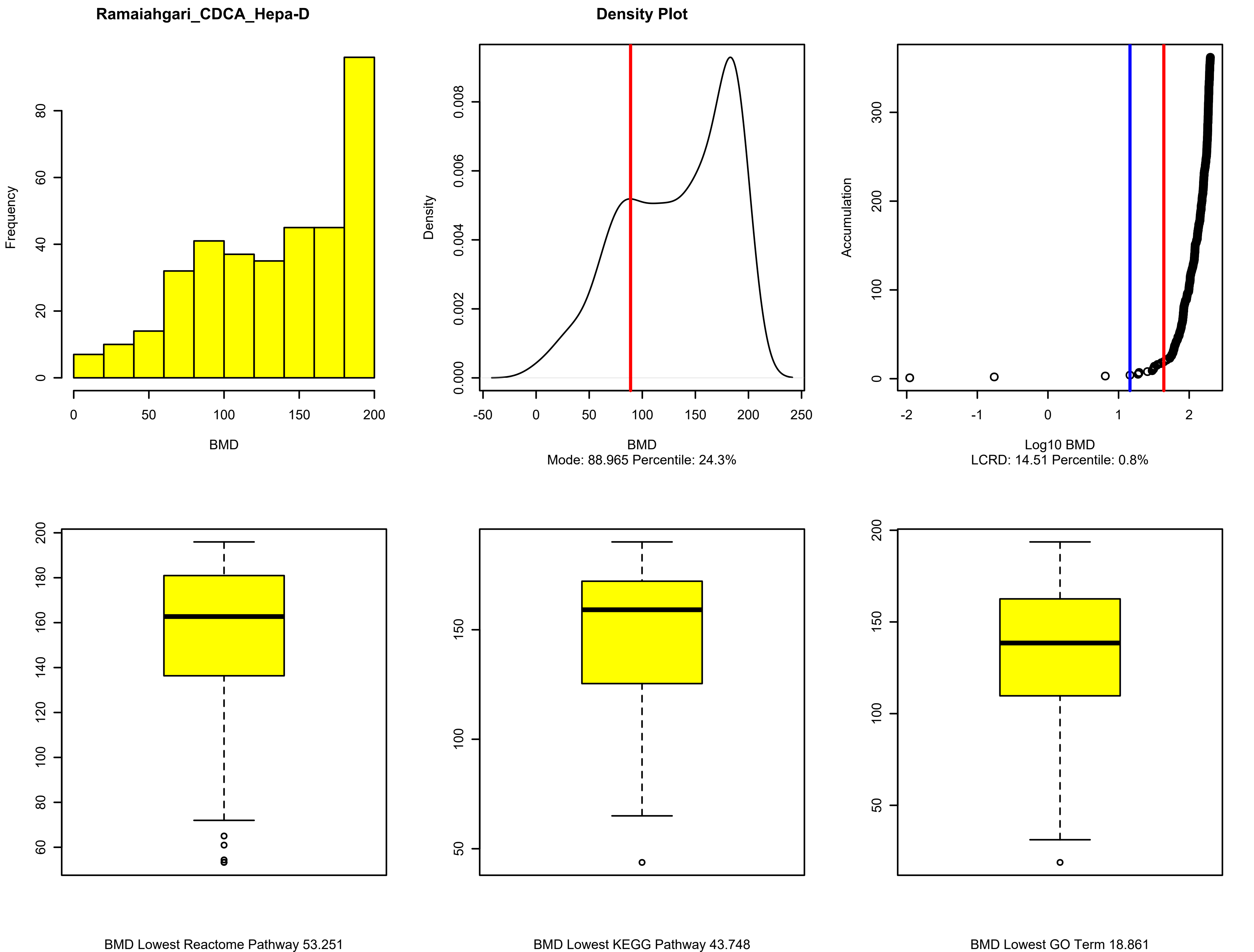
Ramaiahgari_CDCA_Hepa-D
Frequency
BMD
Density Plot
Density
BMD
Mode: 88.965 Percentile: 24.3%
Accumulation
Log10 BMD
LCRD: 14.51 Percentile: 0.8%
BMD Lowest Reactome Pathway 53.251
BMD Lowest KEGG Pathway 43.748
BMD Lowest GO Term 18.861

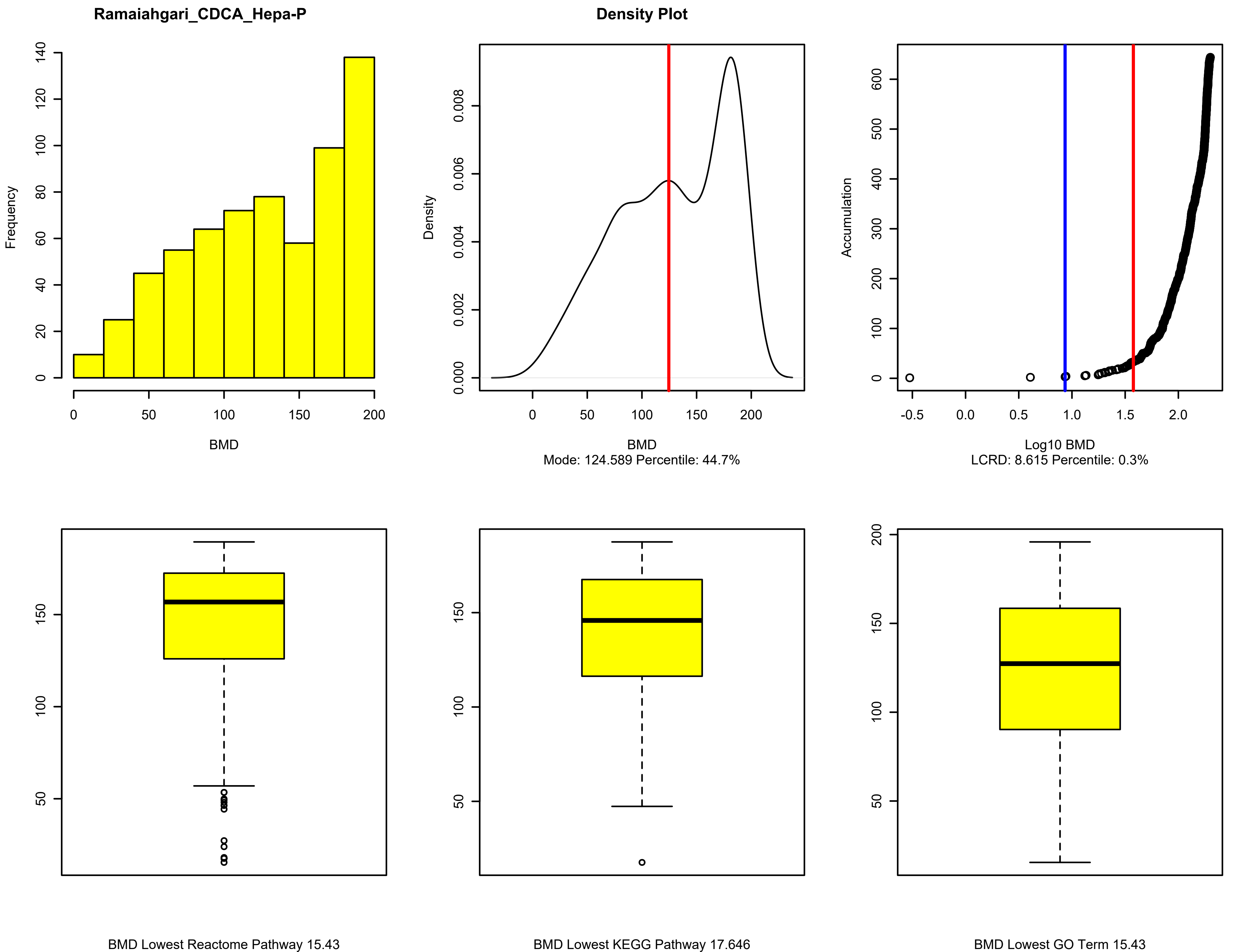
Ramaiahgari_CDCA_Hepa-P
Frequency
BMD
Density Plot
Density
BMD
Mode: 124.589 Percentile: 44.7%
Accumulation
Log10 BMD
LCRD: 8.615 Percentile: 0.3%
BMD Lowest Reactome Pathway 15.43
BMD Lowest KEGG Pathway 17.646
BMD Lowest GO Term 15.43

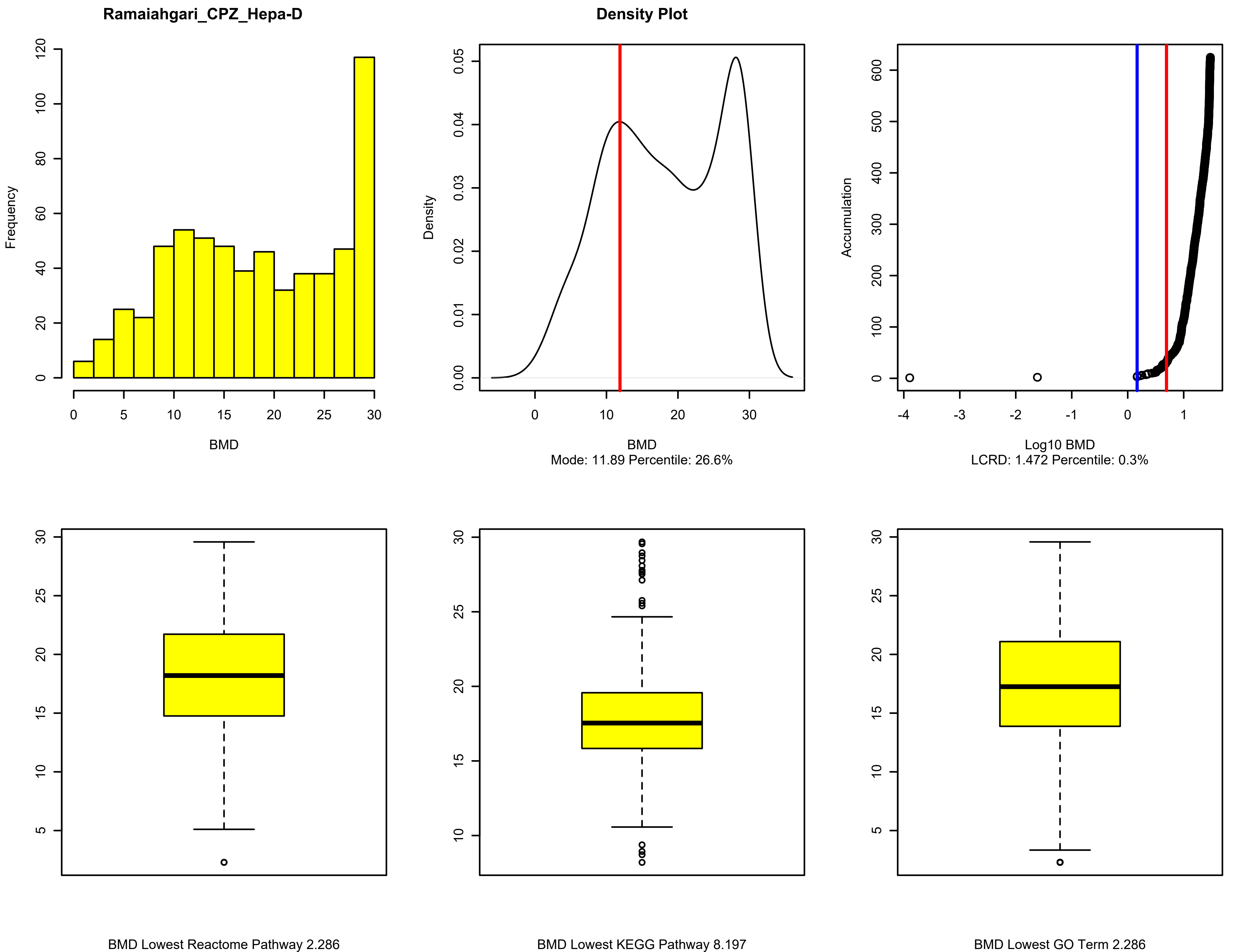

Ramaiahgari_CPZ_Hepa-D
Frequency
BMD
Density Plot
Density
BMD
Mode: 11.89 Percentile: 26.6%
Accumulation
Log10 BMD
LCRD: 1.472 Percentile: 0.3%
BMD Lowest Reactome Pathway 2.286
BMD Lowest KEGG Pathway 8.197
BMD Lowest GO Term 2.286

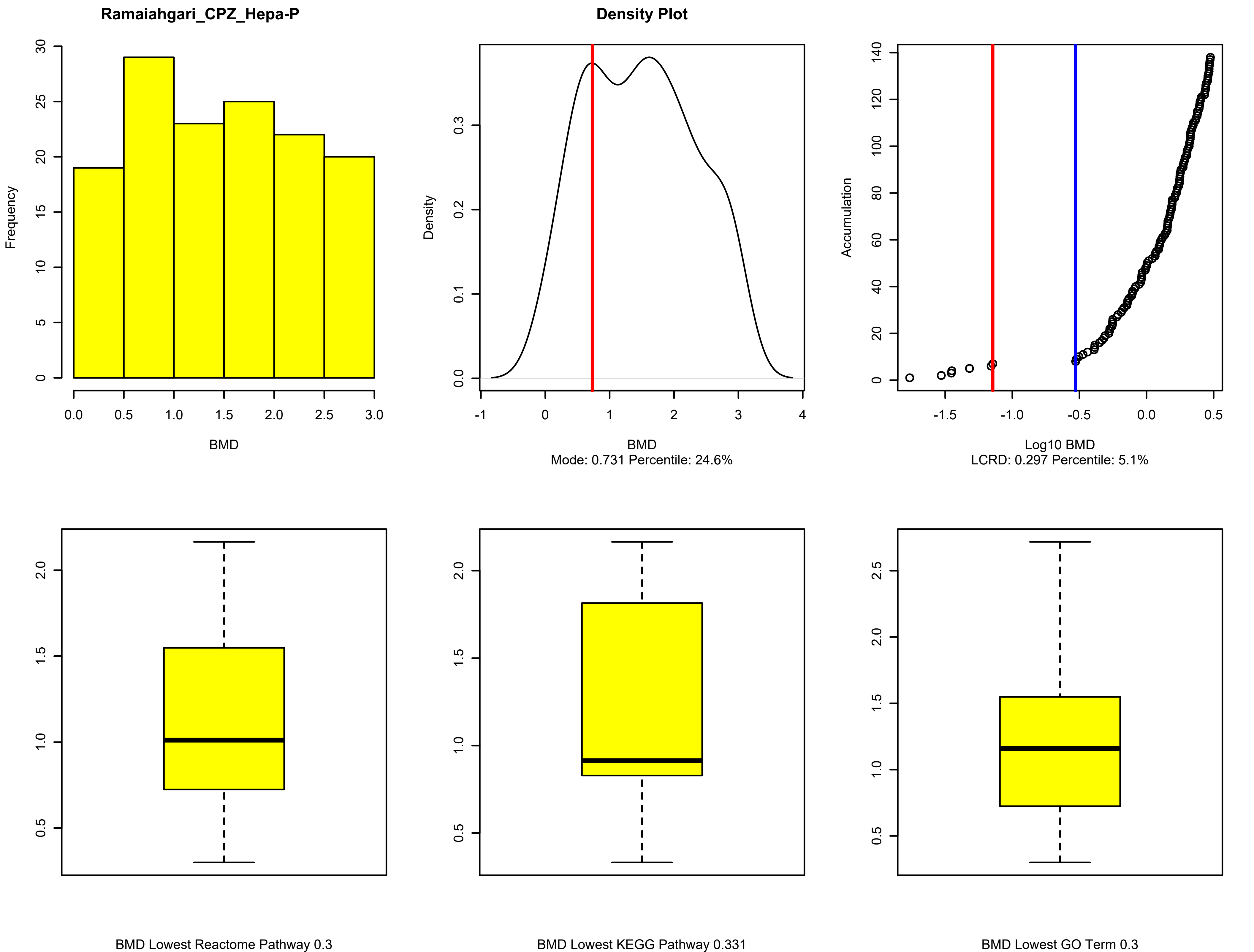

Ramaiahgari_CPZ_Hepa-P
Frequency
BMD
Density Plot
Density
BMD
Mode: 0.731 Percentile: 24.6%
Accumulation
Log10 BMD
LCRD: 0.297 Percentile: 5.1%
BMD Lowest Reactome Pathway 0.3
BMD Lowest KEGG Pathway 0.331
BMD Lowest GO Term 0.3

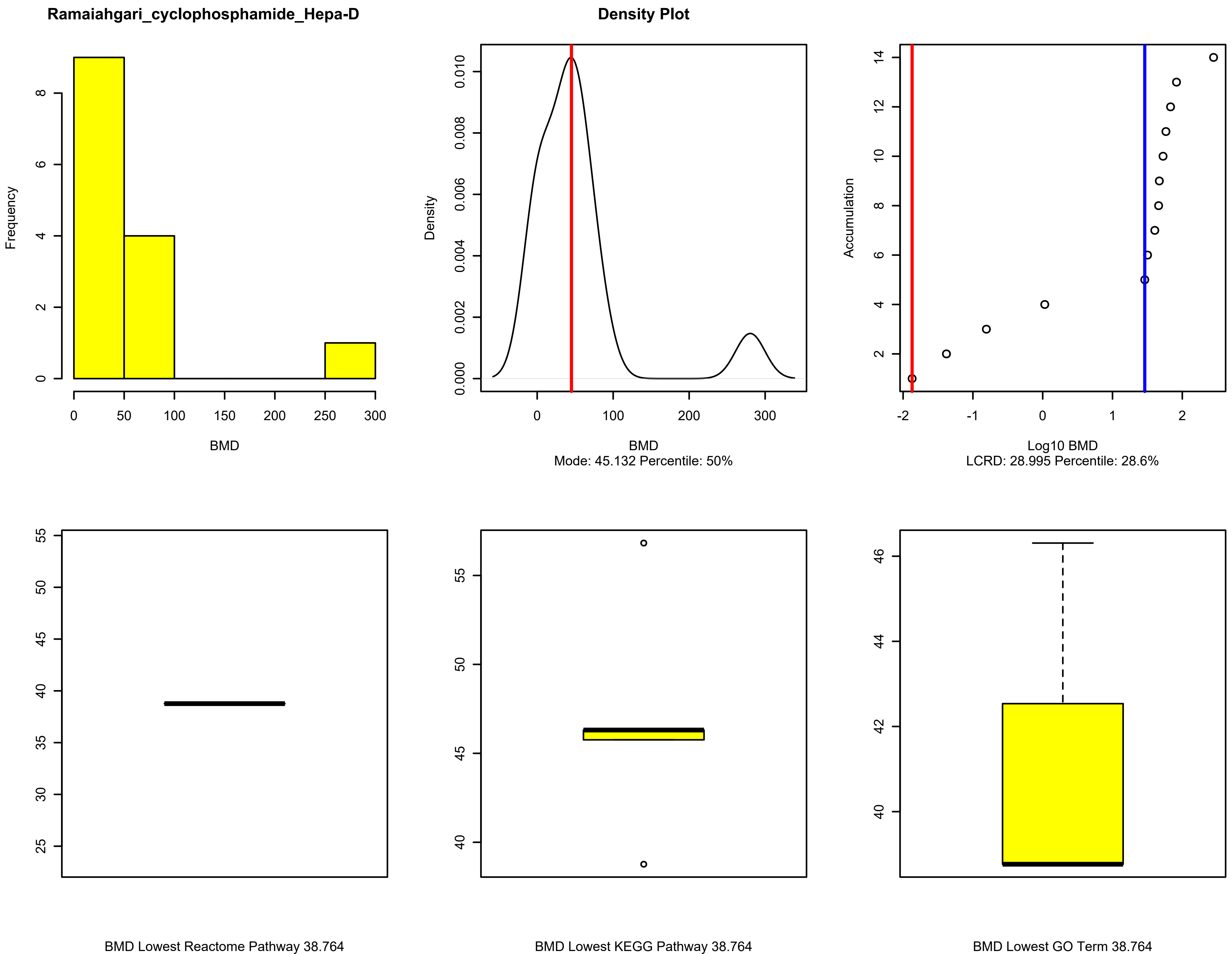

Ramaiahgari_cyclophosphamide_Hepa-D
Frequency
BMD
Density Plot
Density
BMD
Mode: 45.132 Percentile: 50%
Accumulation
Log10 BMD
LCRD: 28.995 Percentile: 28.6%
BMD Lowest Reactome Pathway 38.764
BMD Lowest KEGG Pathway 38.764
BMD Lowest GO Term 38.764

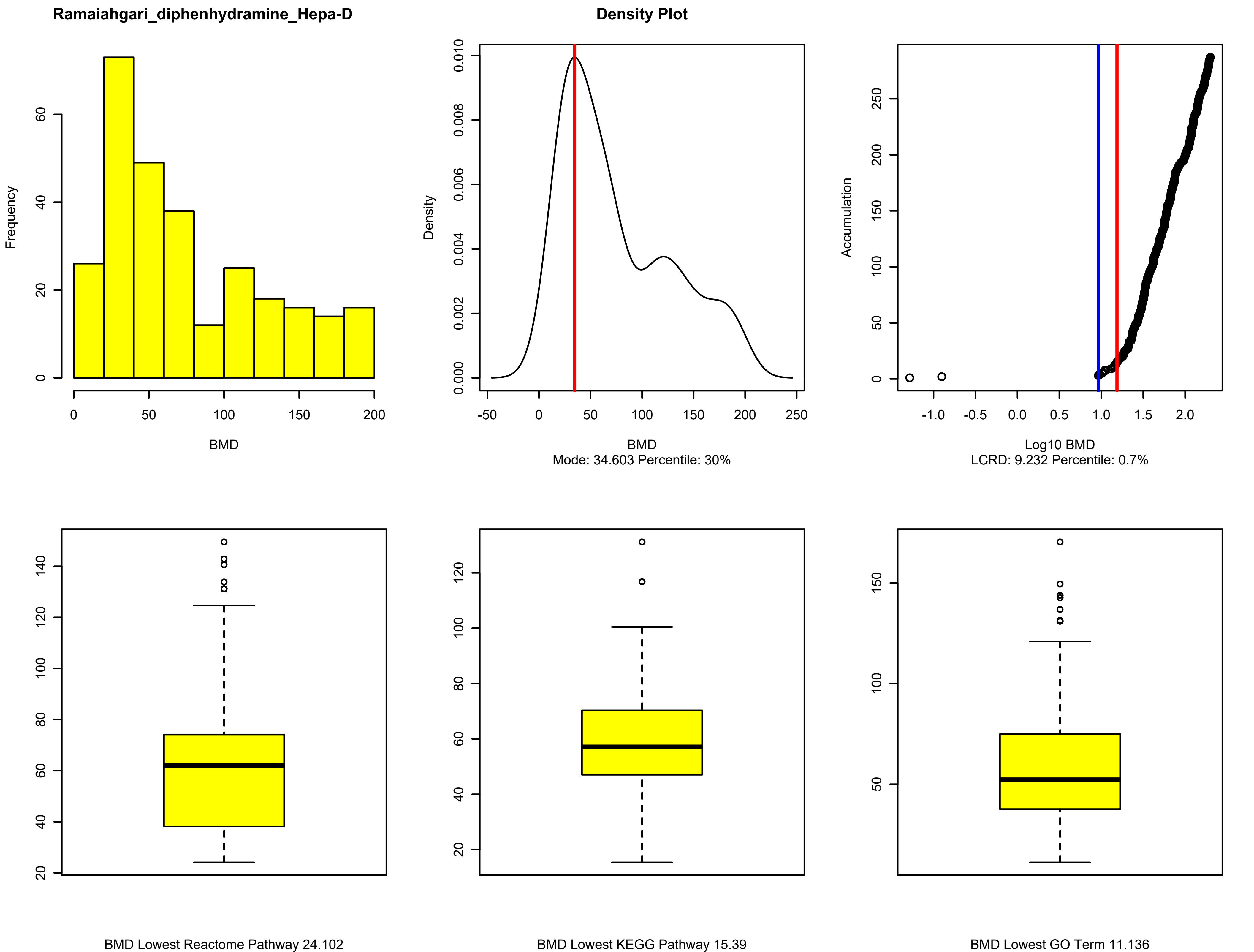

Ramaiahgari_diphenhydramine_Hepa-D
Frequency
BMD
Density Plot
Density
BMD
Mode: 34.603 Percentile: 30%
Accumulation
Log10 BMD
LCRD: 9.232 Percentile: 0.7%
BMD Lowest Reactome Pathway 24.102
BMD Lowest KEGG Pathway 15.39
BMD Lowest GO Term 11.136

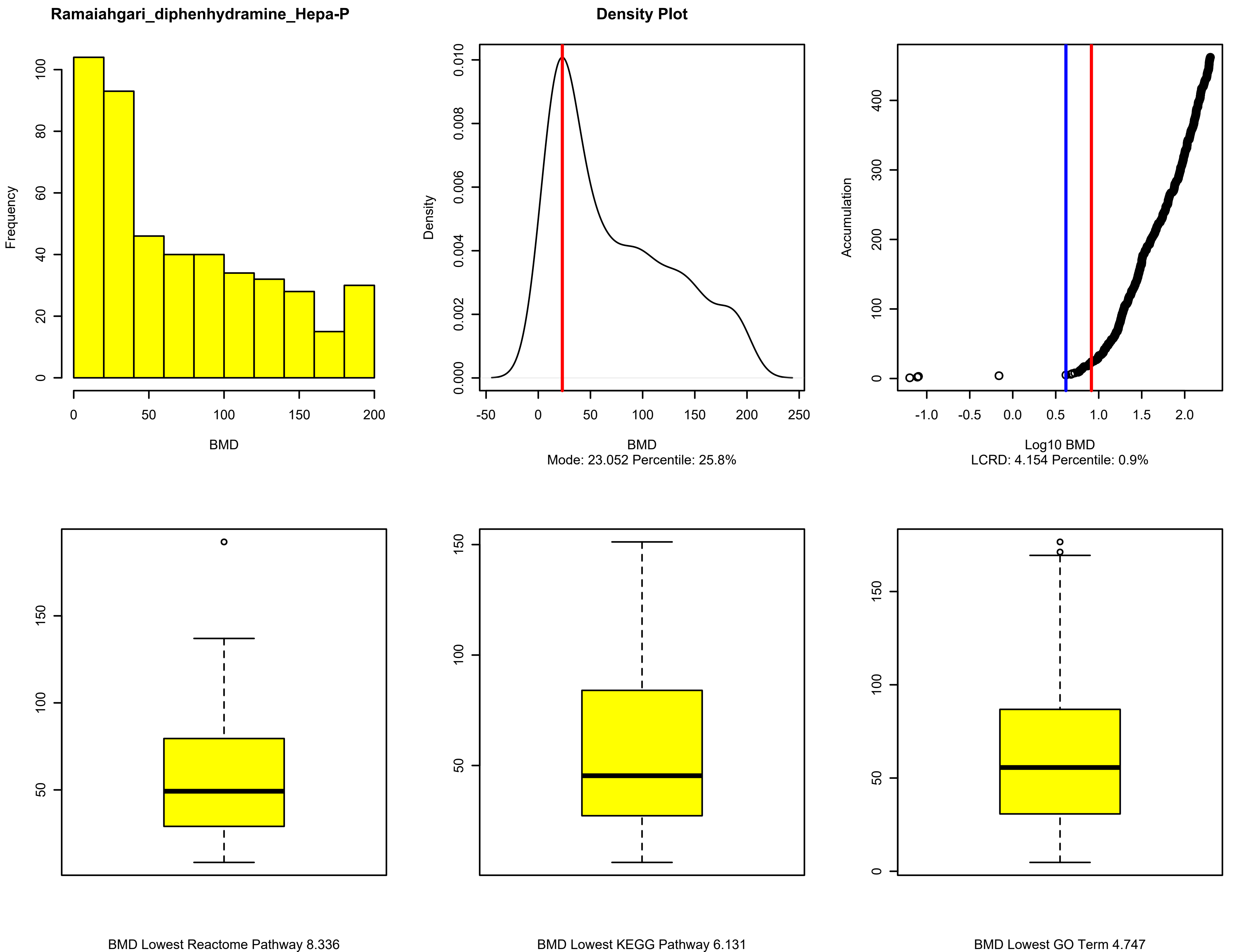
Ramaiahgari_diphenhydramine_Hepa-P
Frequency
BMD
Density Plot
Density
BMD
Mode: 23.052 Percentile: 25.8%
Accumulation
Log10 BMD
LCRD: 4.154 Percentile: 0.9%
BMD Lowest Reactome Pathway 8.336
BMD Lowest KEGG Pathway 6.131
BMD Lowest GO Term 4.747

Ramaiahgari_DMN_Hepa-P

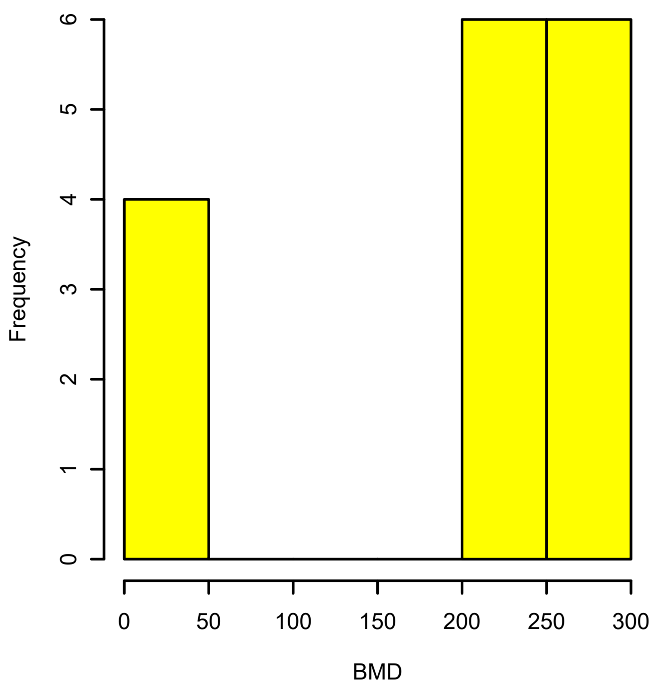

Density Plot

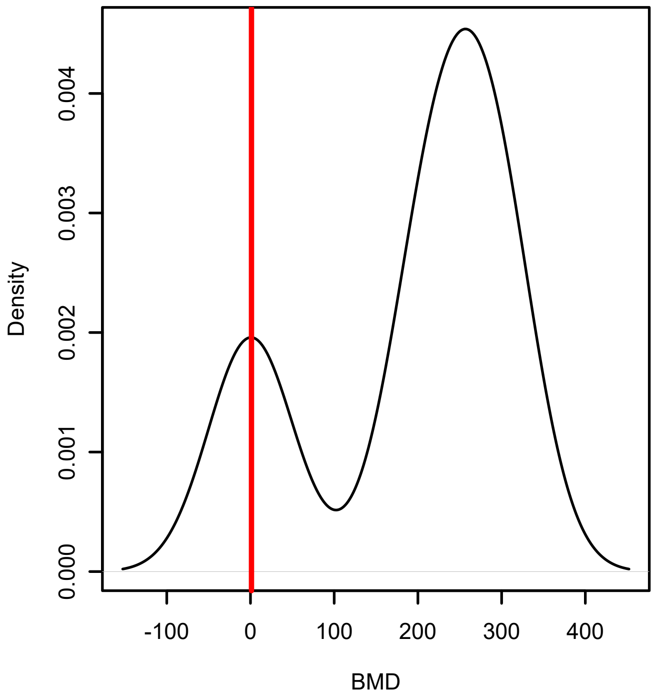

Mode: 1.2 Percentile: 18.8%

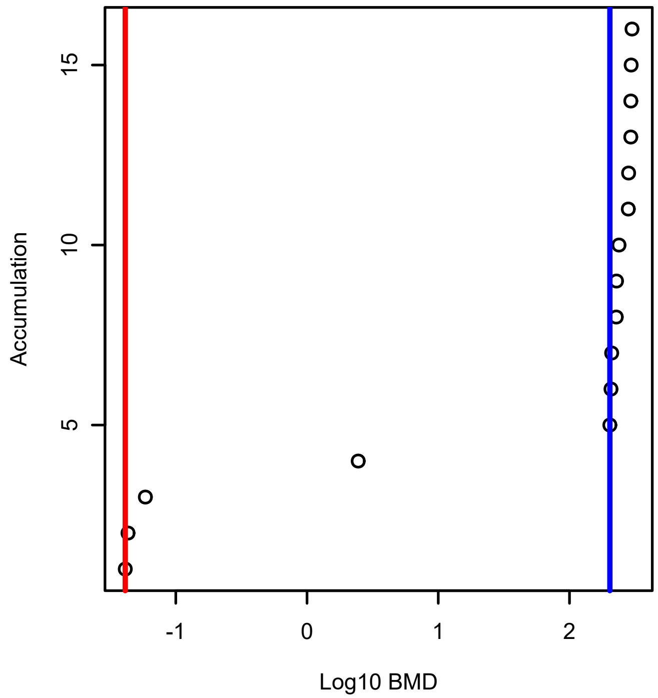

LCRD: 203.144 Percentile: 25%

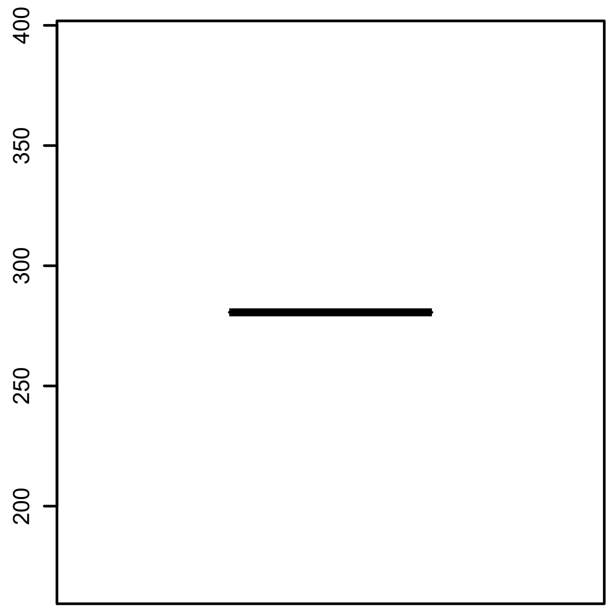

BMD Lowest GO Term 280.627

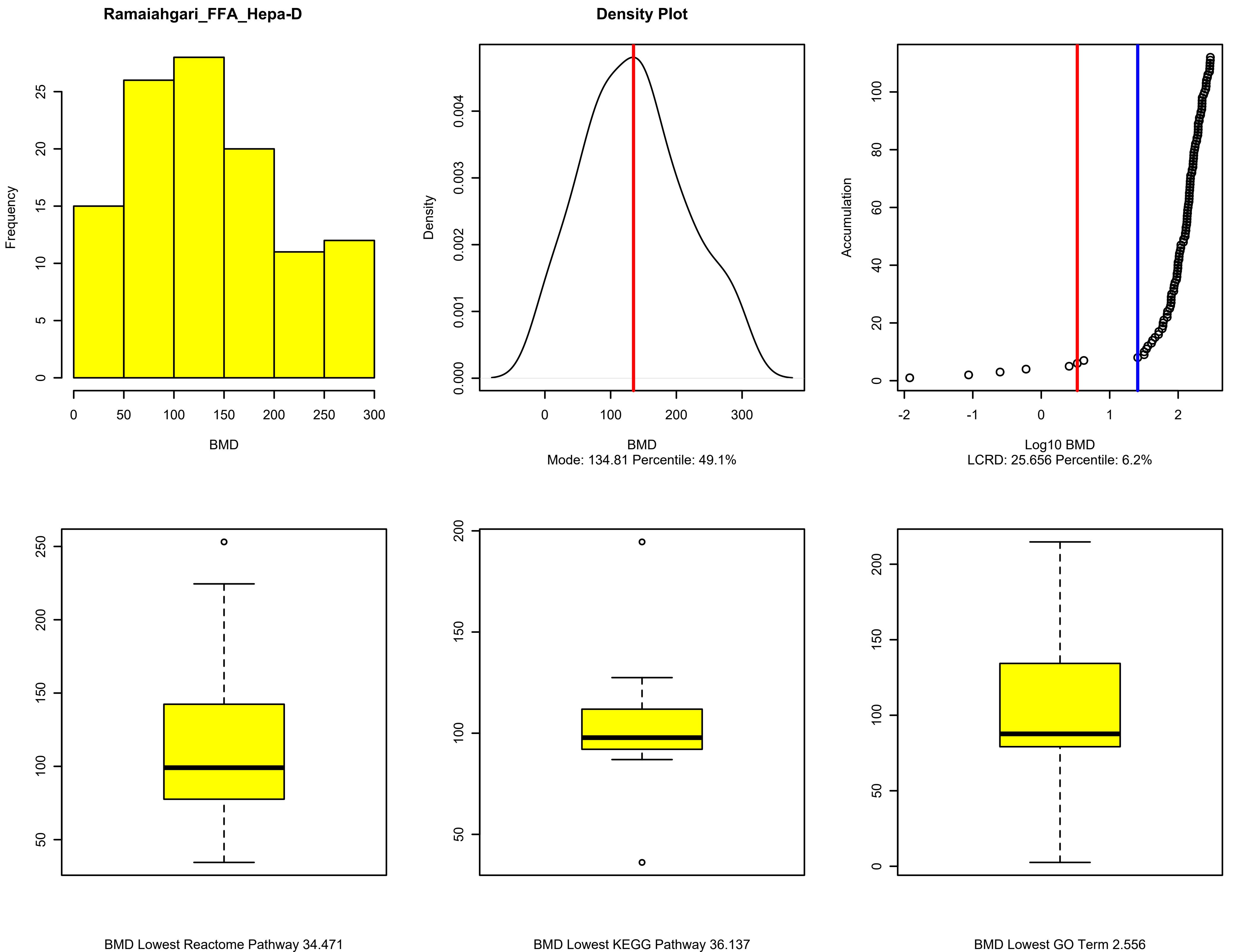

Ramaiahgari_FFA_Hepa-D
Frequency
BMD
Density Plot
Density
BMD
Mode: 134.81 Percentile: 49.1%
Accumulation
Log10 BMD
LCRD: 25.656 Percentile: 6.2%
BMD Lowest Reactome Pathway 34.471
BMD Lowest KEGG Pathway 36.137
BMD Lowest GO Term 2.556

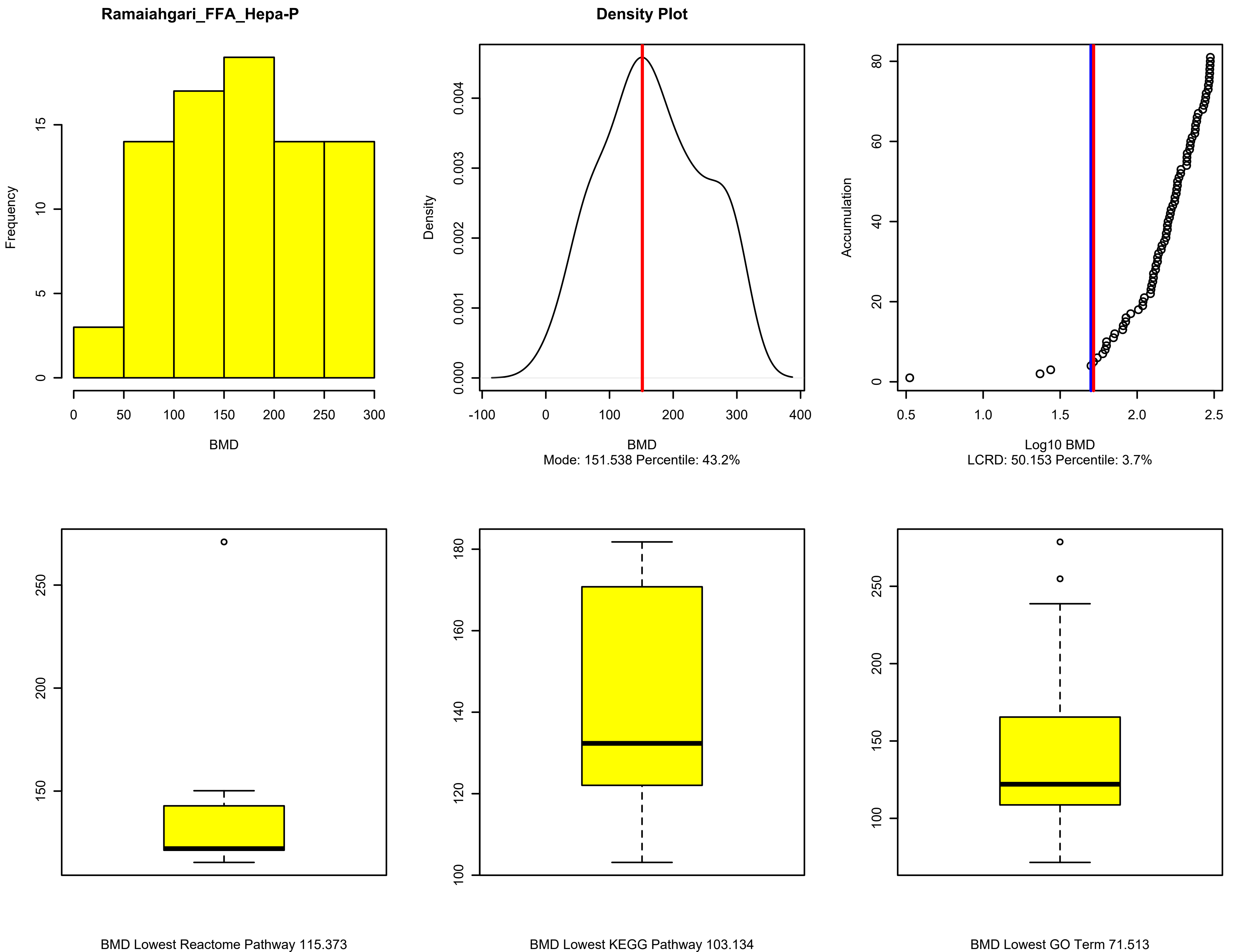
Ramaiahgari_FFA_Hepa-P
Frequency
BMD
Density Plot
Density
BMD
Mode: 151.538 Percentile: 43.2%
Accumulation
Log10 BMD
LCRD: 50.153 Percentile: 3.7%
BMD Lowest Reactome Pathway 115.373
BMD Lowest KEGG Pathway 103.134
BMD Lowest GO Term 71.513

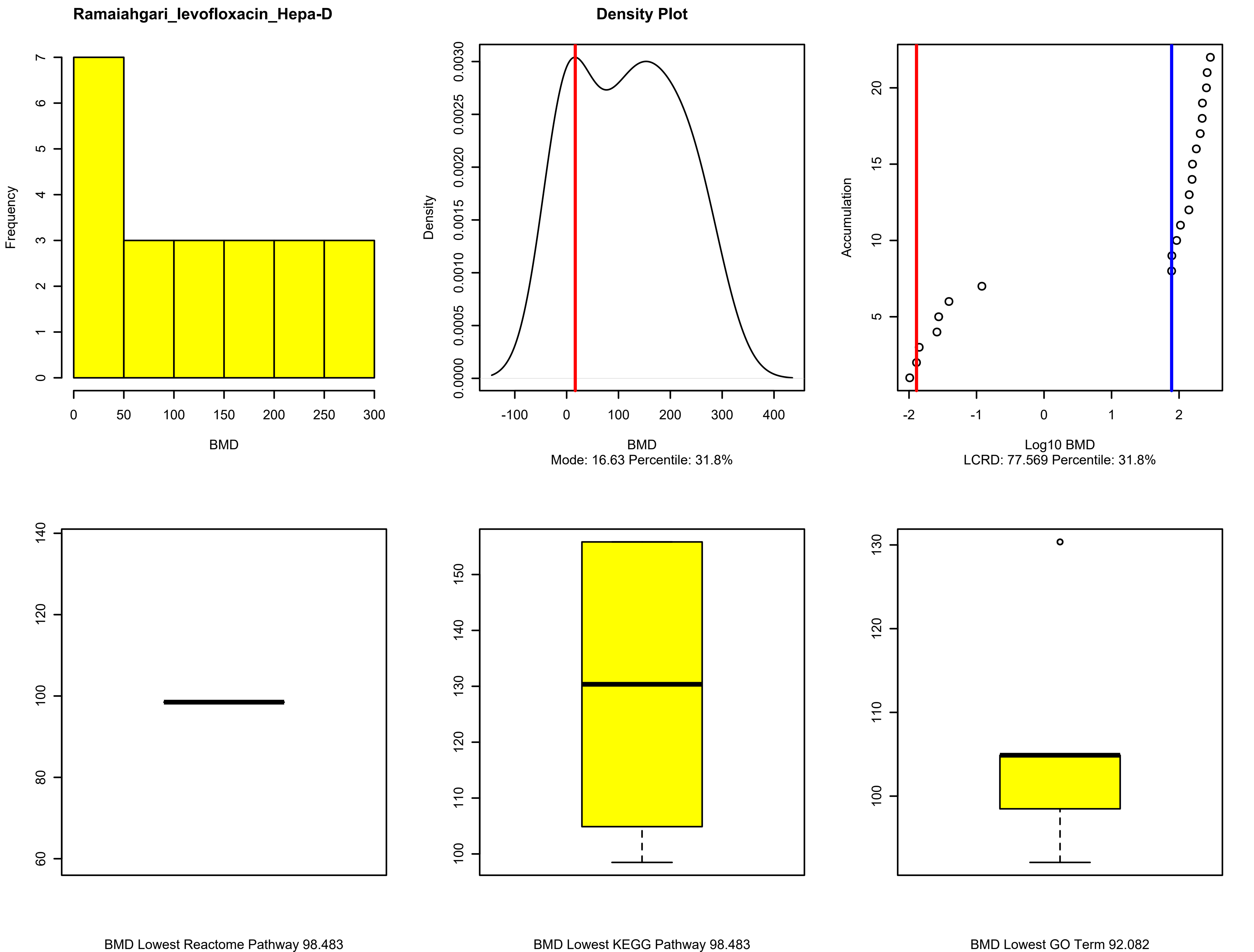

Ramaiahgari_levofloxacin_Hepa-D
Frequency
BMD
Density Plot
Density
BMD
Mode: 16.63 Percentile: 31.8%
Accumulation
Log10 BMD
LCRD: 77.569 Percentile: 31.8%
BMD Lowest Reactome Pathway 98.483
BMD Lowest KEGG Pathway 98.483
BMD Lowest GO Term 92.082

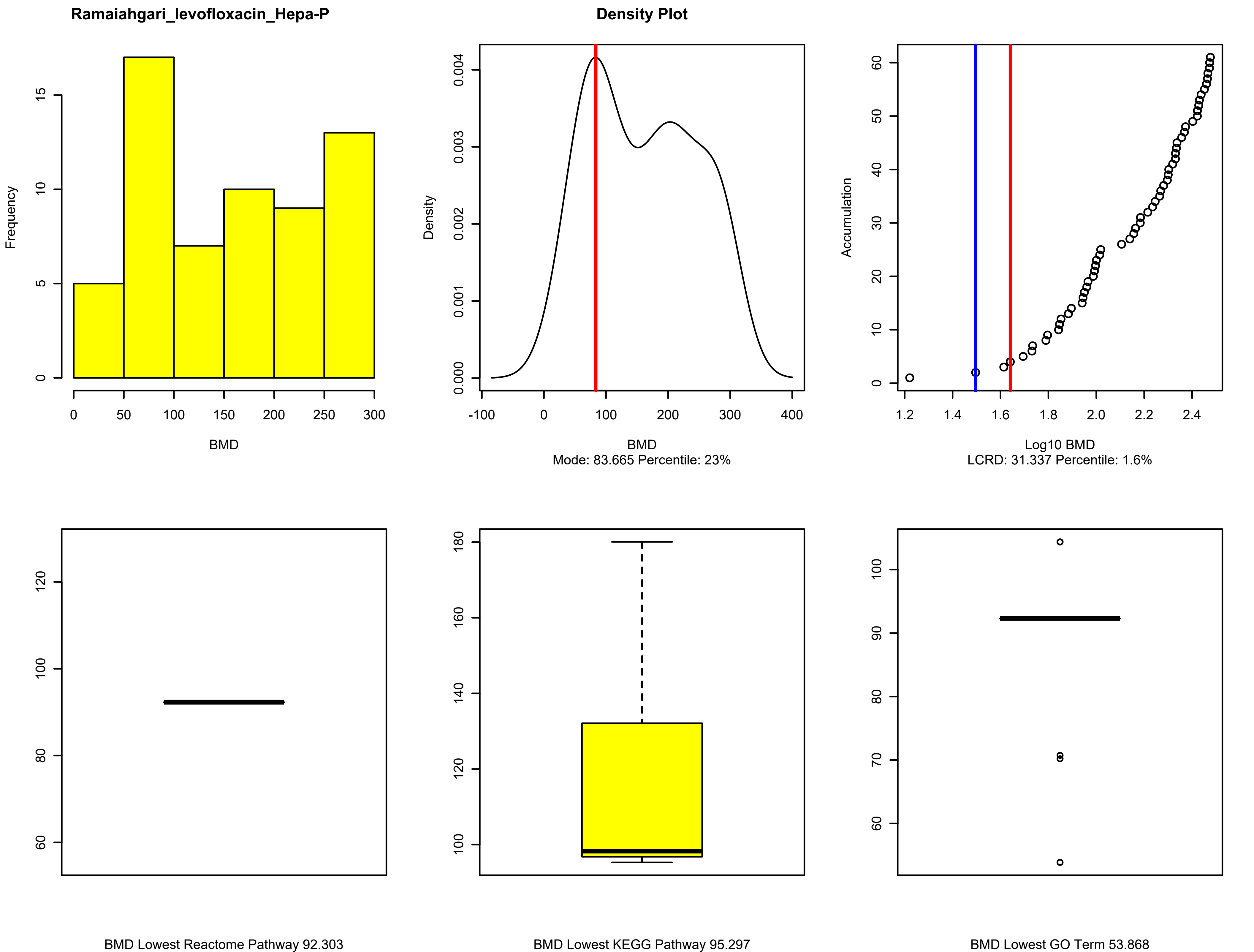
Ramaiahgari_levofloxacin_Hepa-P
Frequency
BMD
Density Plot
Density
BMD
Mode: 83.665 Percentile: 23%
Accumulation
Log10 BMD
LCRD: 31.337 Percentile: 1.6%
BMD Lowest Reactome Pathway 92.303
BMD Lowest KEGG Pathway 95.297
BMD Lowest GO Term 53.868

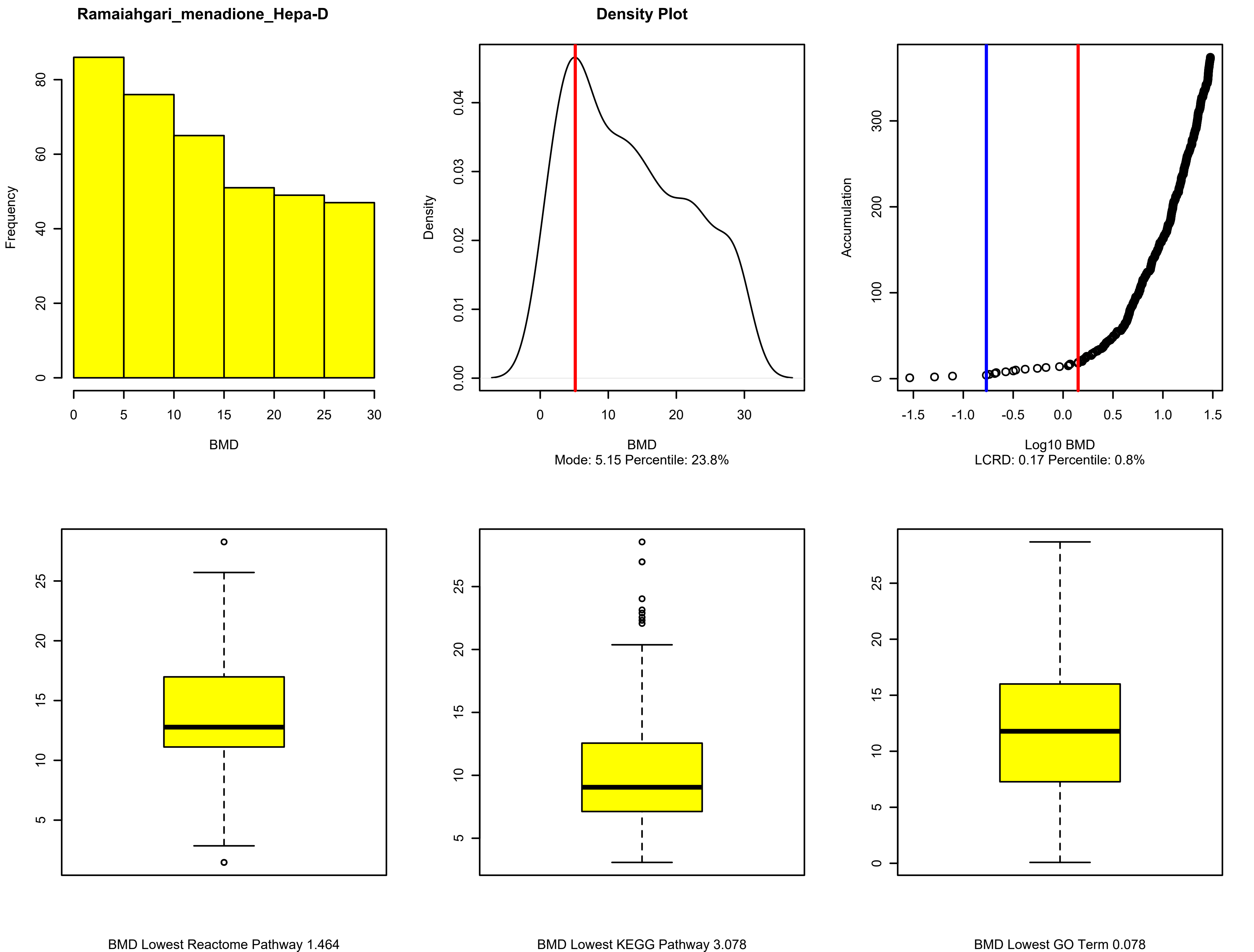

Ramaiahgari_menadione_Hepa-D
Frequency
BMD
Density Plot
Density
BMD
Mode: 5.15 Percentile: 23.8%
Accumulation
Log10 BMD
LCRD: 0.17 Percentile: 0.8%
BMD Lowest Reactome Pathway 1.464
BMD Lowest KEGG Pathway 3.078
BMD Lowest GO Term 0.078

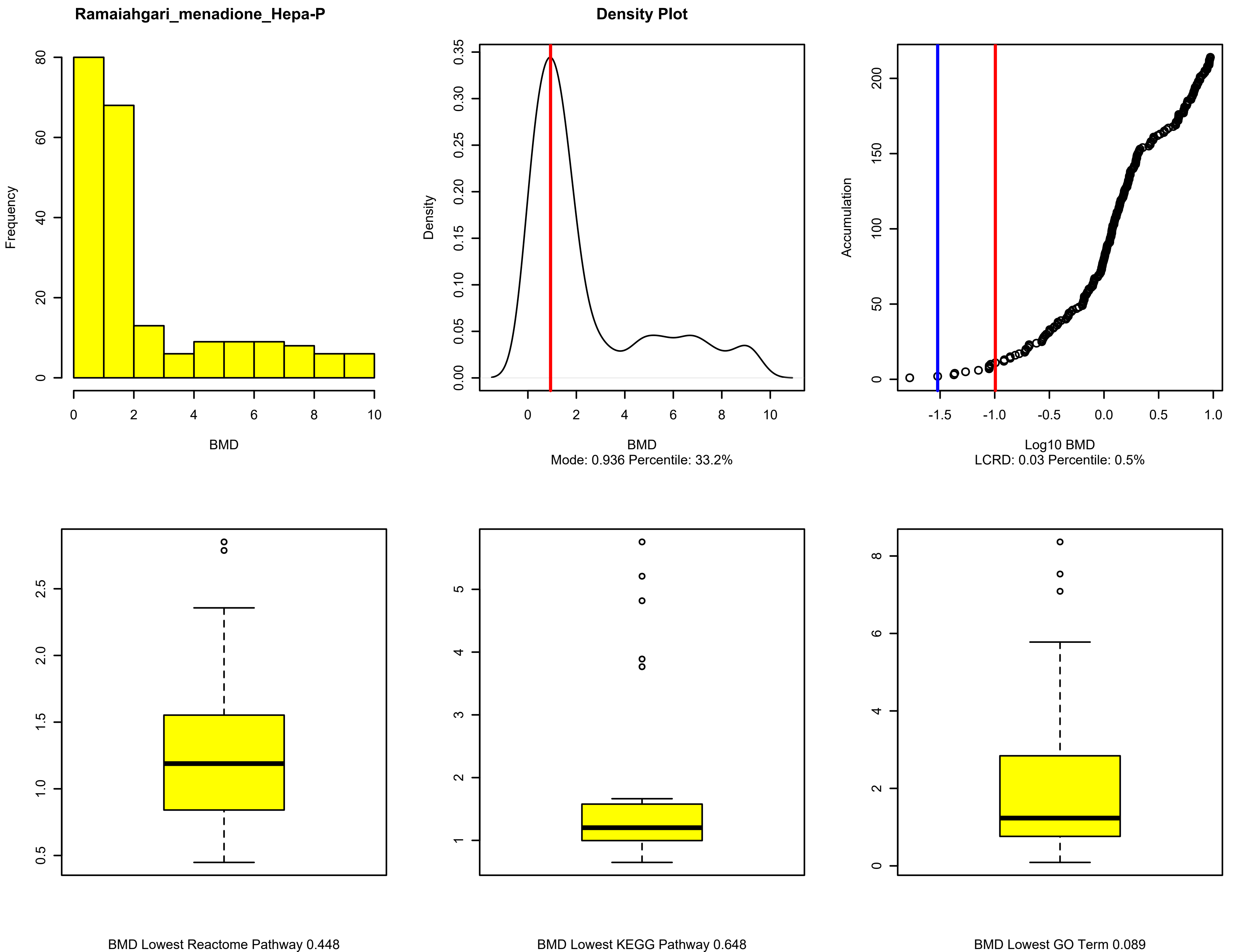

Ramaiahgari_menadione_Hepa-P
Frequency
BMD
Density Plot
Density
BMD
Mode: 0.936 Percentile: 33.2%
Accumulation
Log10 BMD
LCRD: 0.03 Percentile: 0.5%
BMD Lowest Reactome Pathway 0.448
BMD Lowest KEGG Pathway 0.648
BMD Lowest GO Term 0.089

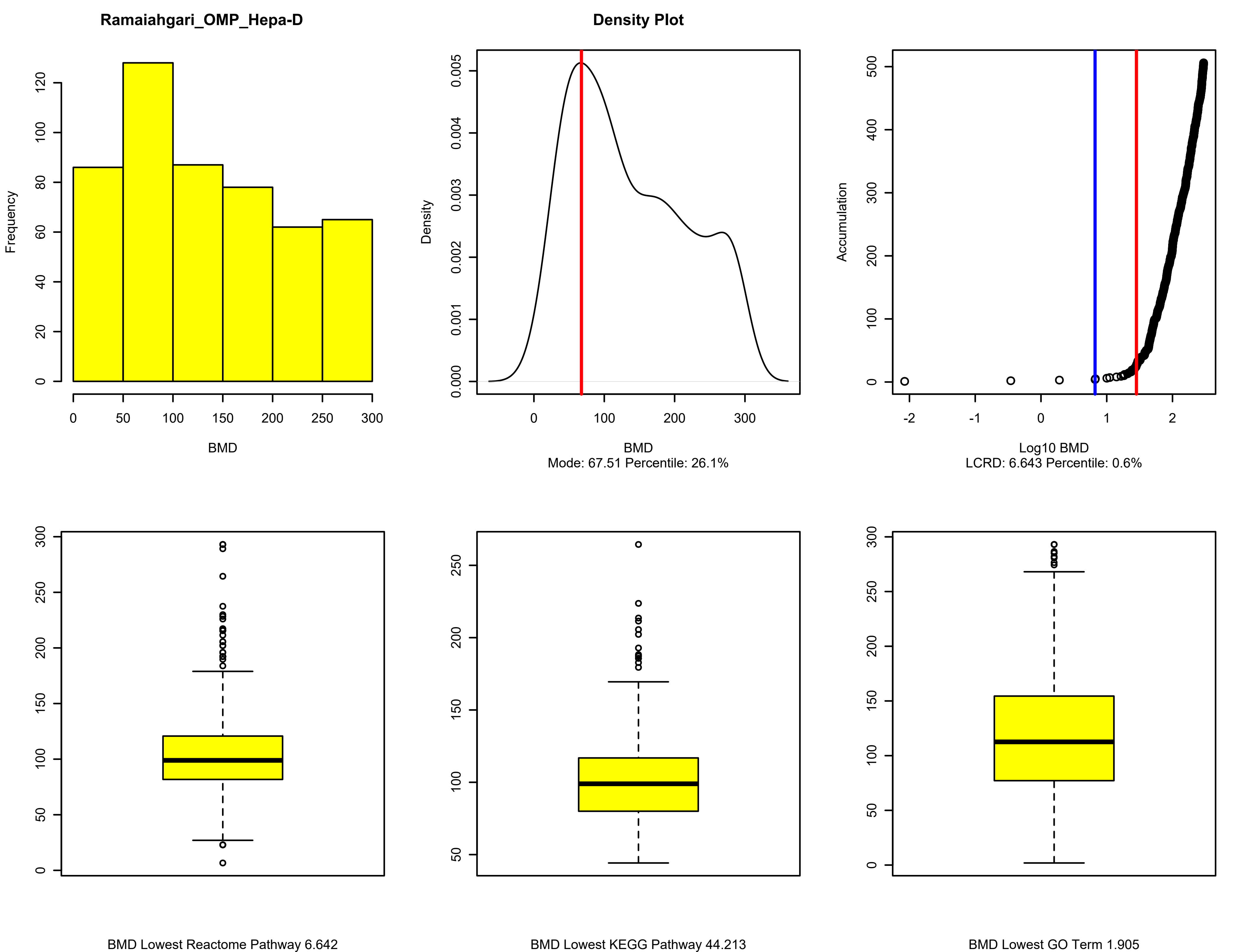
Ramaiahgari_OMP_Hepa-D
Frequency
BMD
Density Plot
Density
BMD
Mode: 67.51 Percentile: 26.1%
Accumulation
Log10 BMD
LCRD: 6.643 Percentile: 0.6%
BMD Lowest Reactome Pathway 6.642
BMD Lowest KEGG Pathway 44.213
BMD Lowest GO Term 1.905

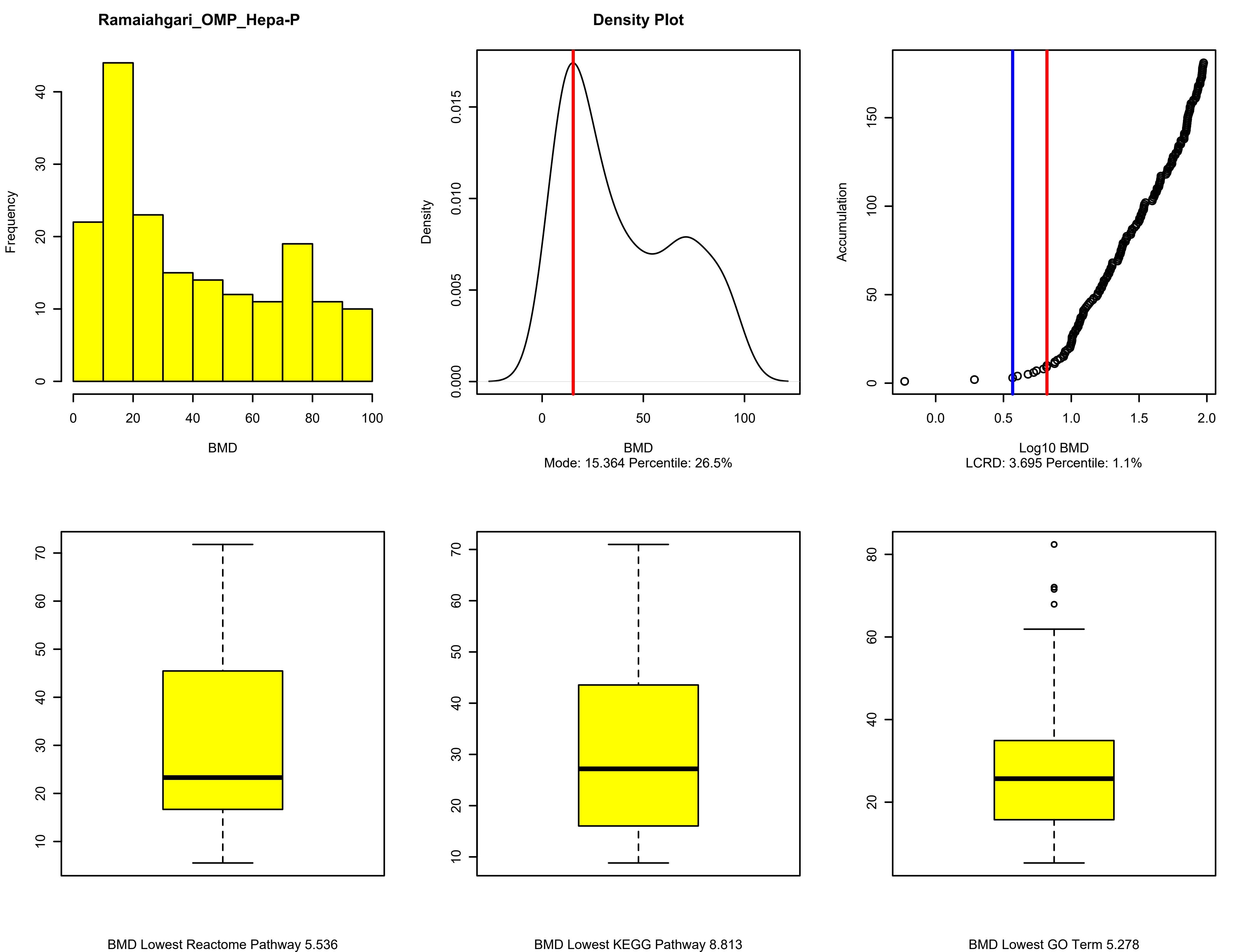

Ramaiahgari_OMP_Hepa-P
Frequency
BMD
Density Plot
Density
BMD
Mode: 15.364 Percentile: 26.5%
Accumulation
Log10 BMD
LCRD: 3.695 Percentile: 1.1%
BMD Lowest Reactome Pathway 5.536
BMD Lowest KEGG Pathway 8.813
BMD Lowest GO Term 5.278

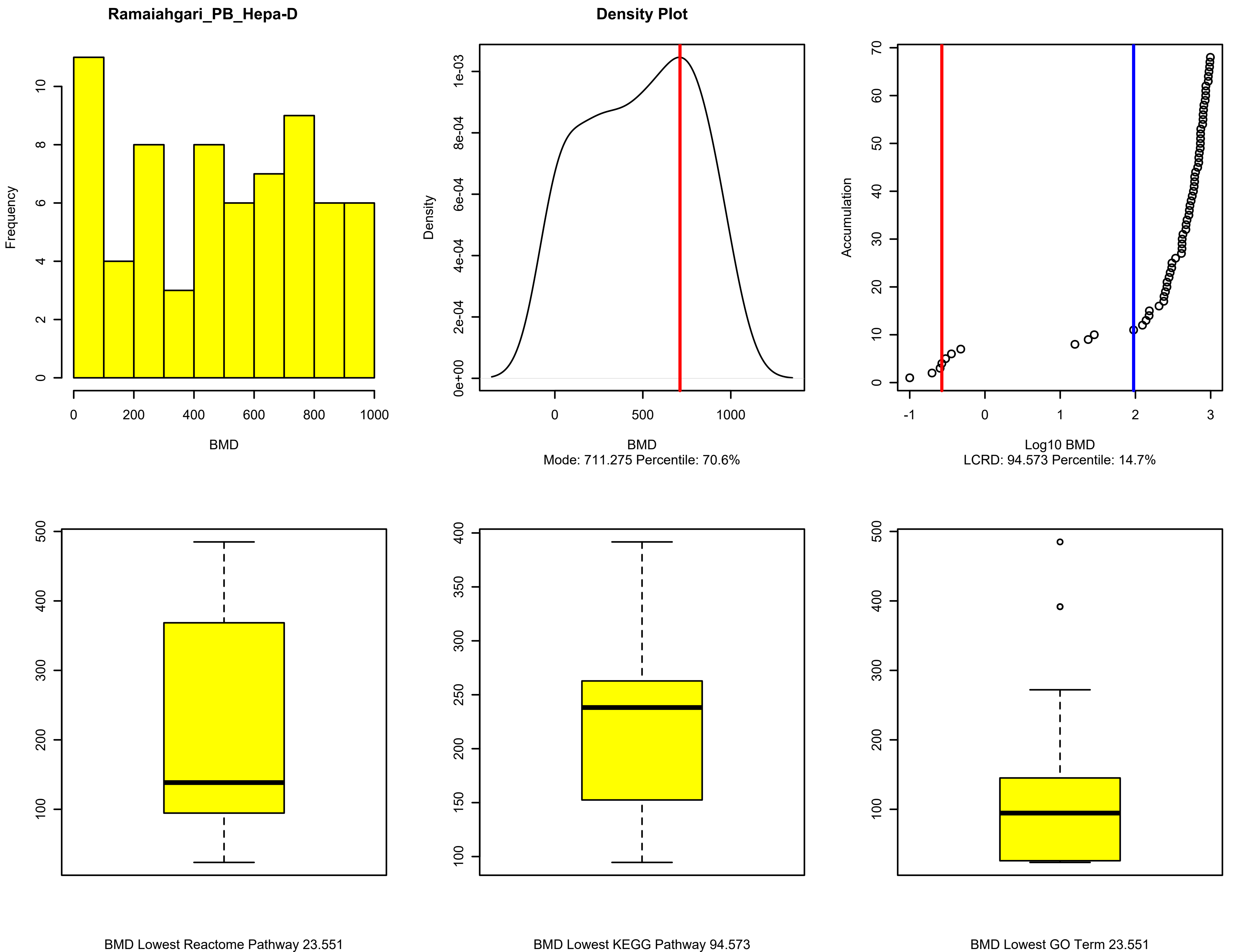

Ramaiahgari_PB_Hepa-D
Frequency
BMD
Density Plot
Density
BMD
Mode: 711.275 Percentile: 70.6%
Accumulation
Log10 BMD
LCRD: 94.573 Percentile: 14.7%
BMD Lowest Reactome Pathway 23.551
BMD Lowest KEGG Pathway 94.573
BMD Lowest GO Term 23.551

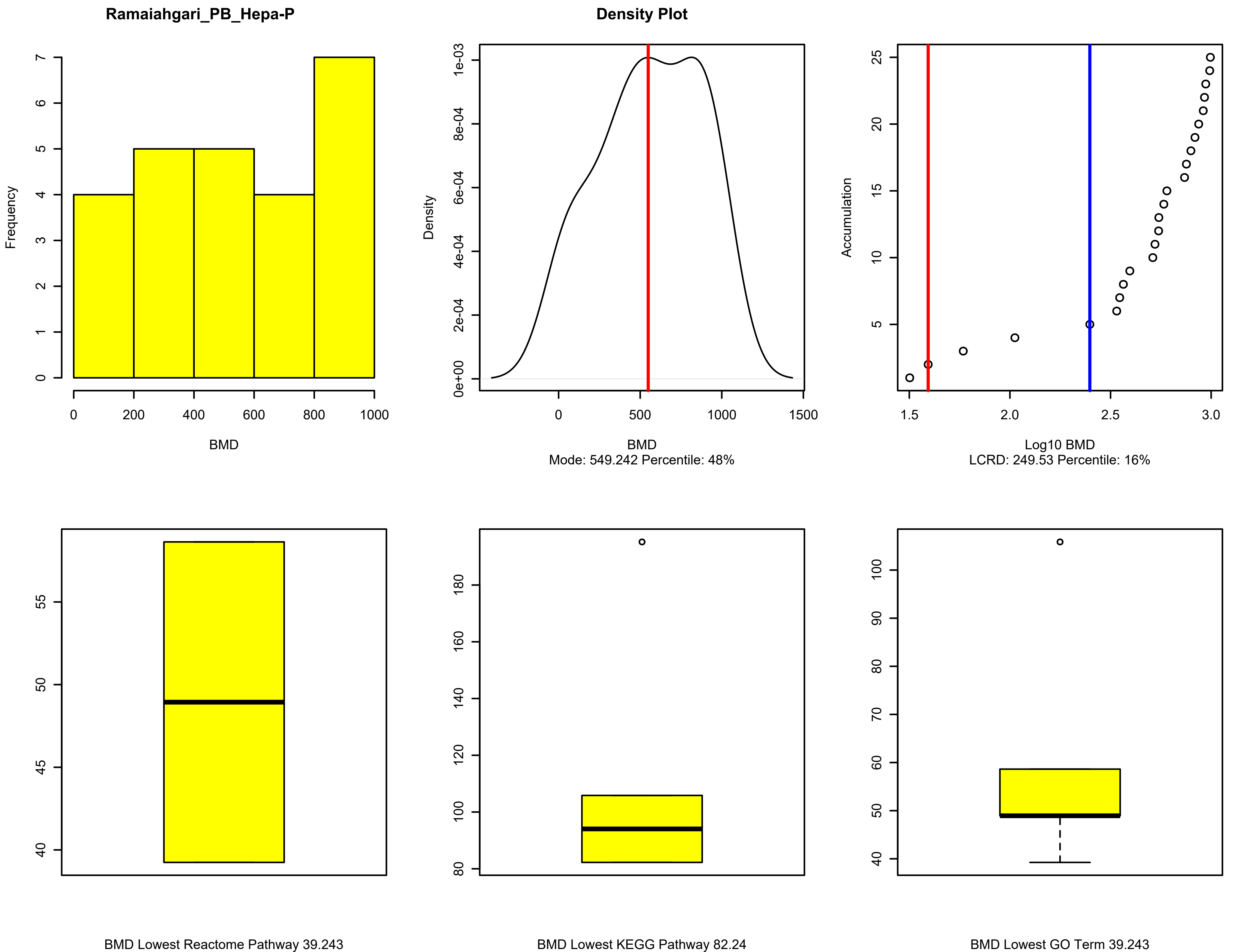

Ramaiahgari_PB_Hepa-P
Frequency
BMD
Density Plot
Density
BMD
Mode: 549.242 Percentile: 48%
Accumulation
Log10 BMD
LCRD: 249.53 Percentile: 16%
BMD Lowest Reactome Pathway 39.243
BMD Lowest KEGG Pathway 82.24
BMD Lowest GO Term 39.243

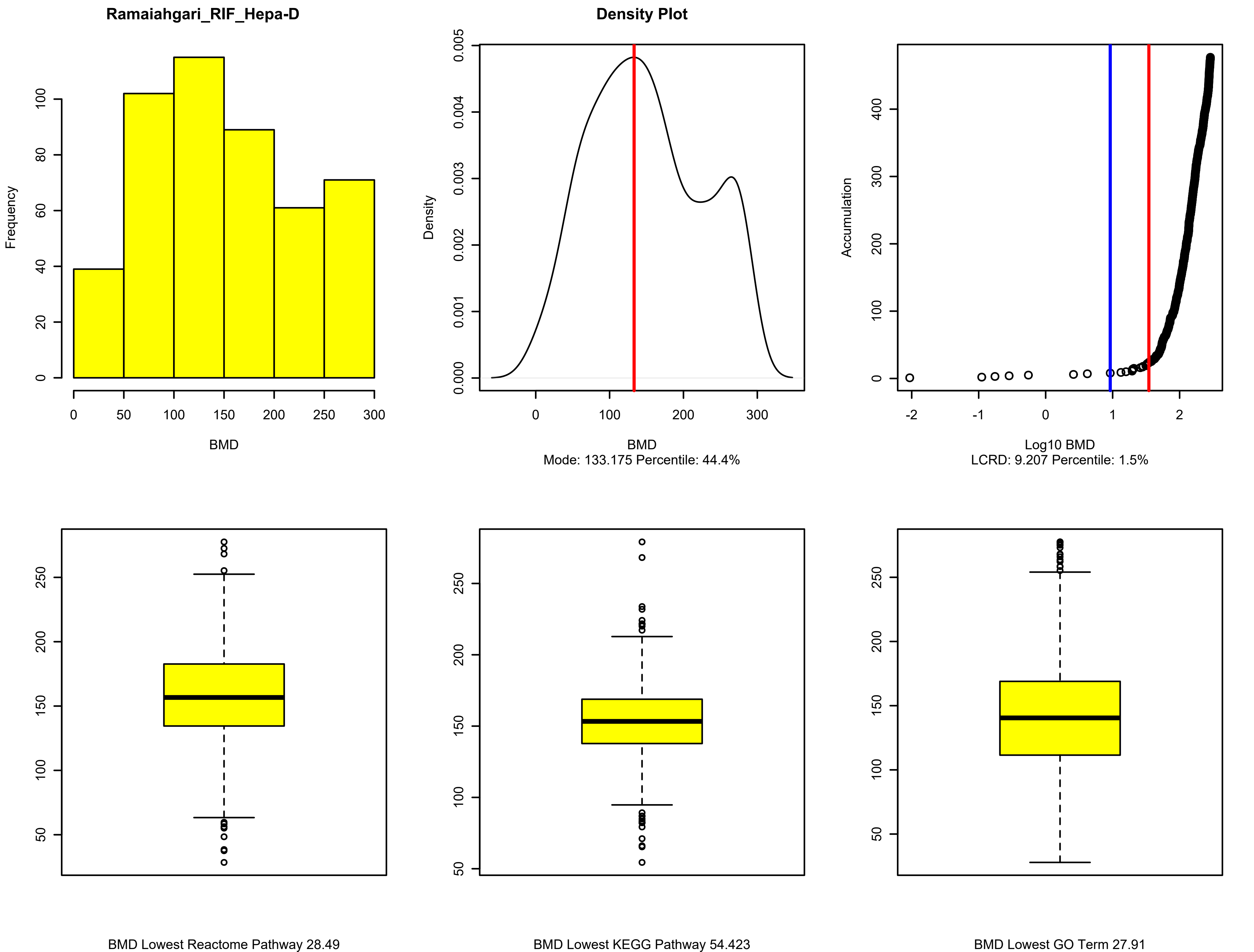

Ramaiahgari_RIF_Hepa-D
Frequency
BMD
Density Plot
Density
BMD
Mode: 133.175 Percentile: 44.4%
Accumulation
Log10 BMD
LCRD: 9.207 Percentile: 1.5%
BMD Lowest Reactome Pathway 28.49
BMD Lowest KEGG Pathway 54.423
BMD Lowest GO Term 27.91

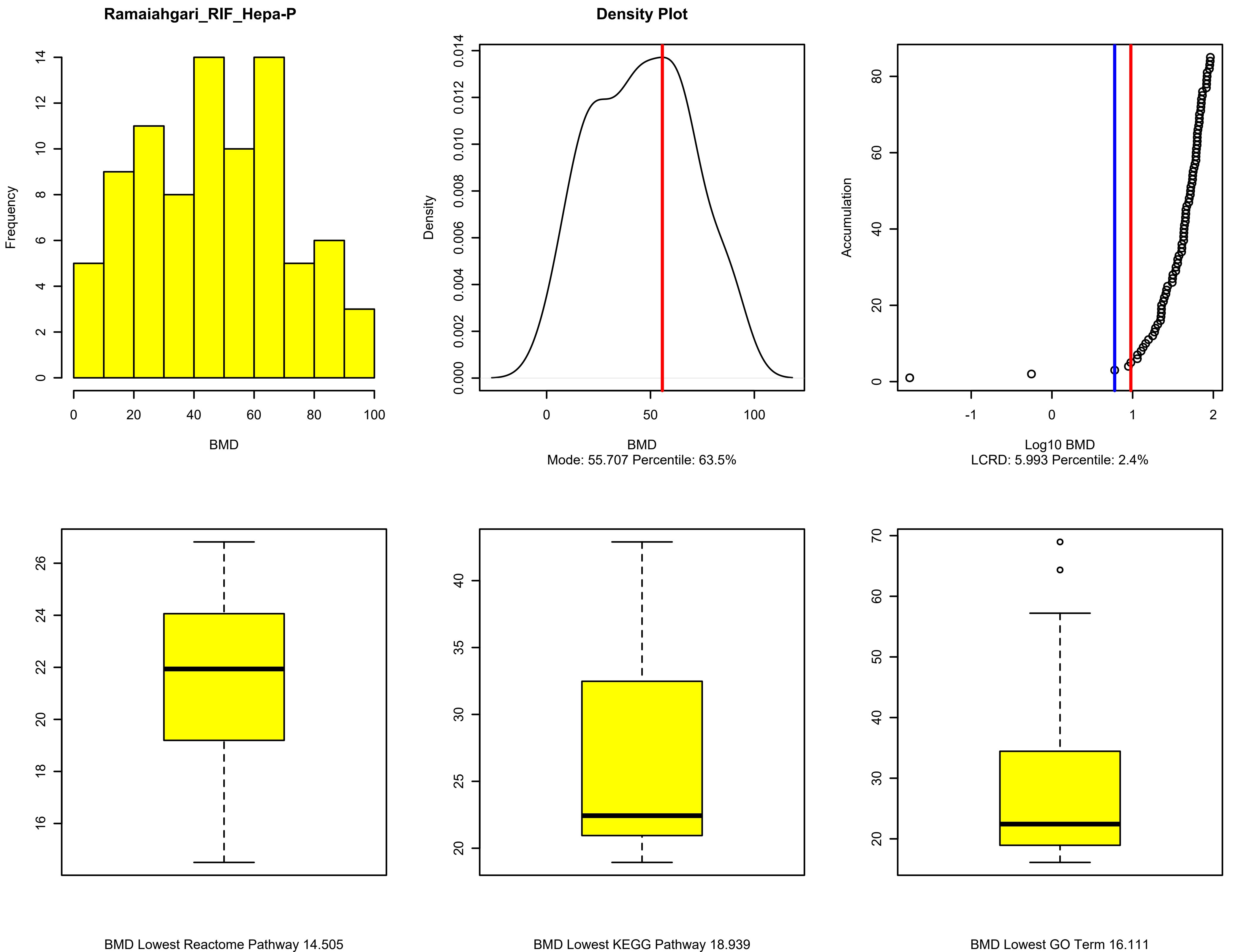

Ramaiahgari_RIF_Hepa-P
Frequency
BMD
Density Plot
Density
BMD
Mode: 55.707 Percentile: 63.5%
Accumulation
Log10 BMD
LCRD: 5.993 Percentile: 2.4%
BMD Lowest Reactome Pathway 14.505
BMD Lowest KEGG Pathway 18.939
BMD Lowest GO Term 16.111

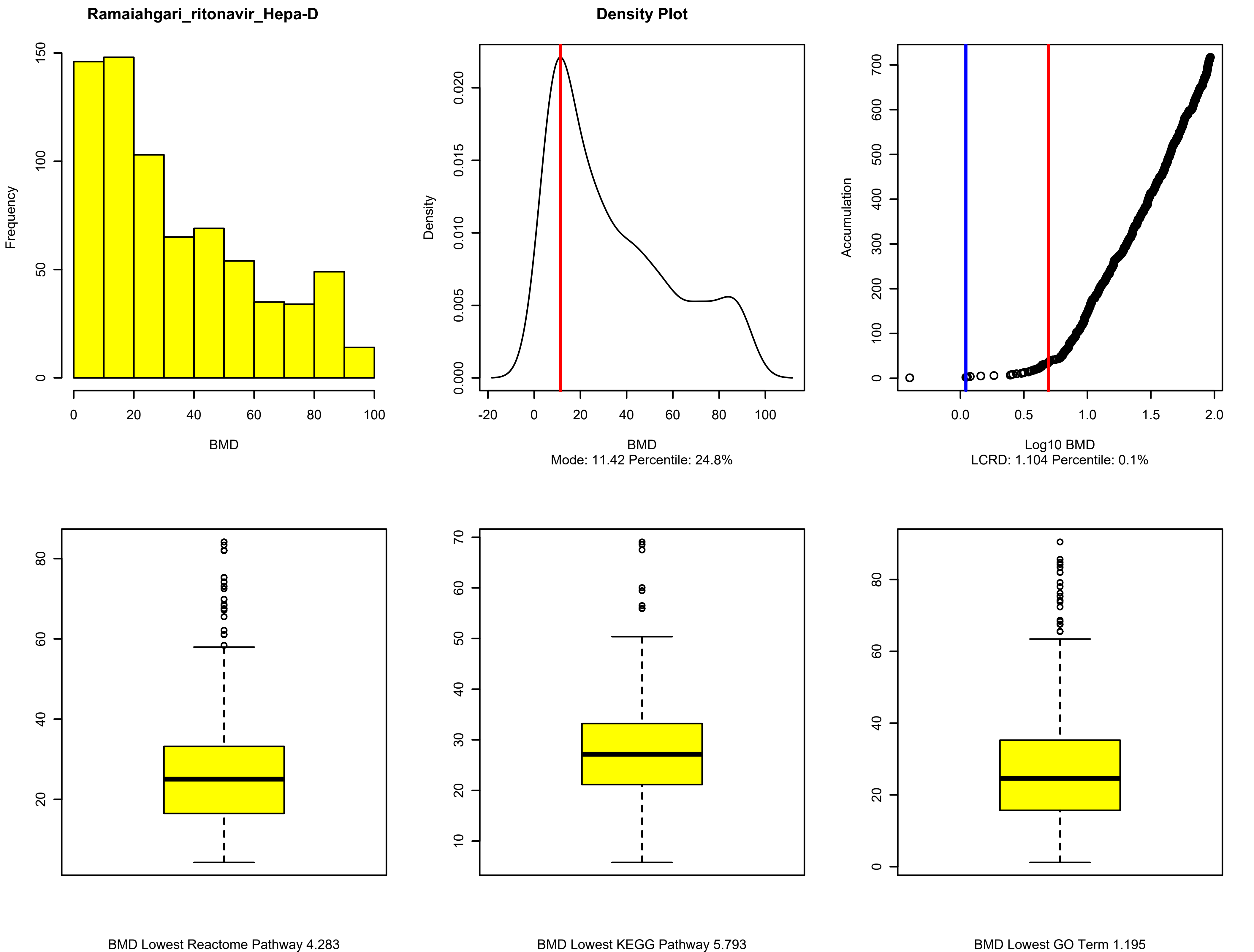

Ramaiahgari_ritonavir_Hepa-D
Frequency
BMD
Density Plot
Density
BMD
Mode: 11.42 Percentile: 24.8%
Accumulation
Log10 BMD
LCRD: 1.104 Percentile: 0.1%
BMD Lowest Reactome Pathway 4.283
BMD Lowest KEGG Pathway 5.793
BMD Lowest GO Term 1.195

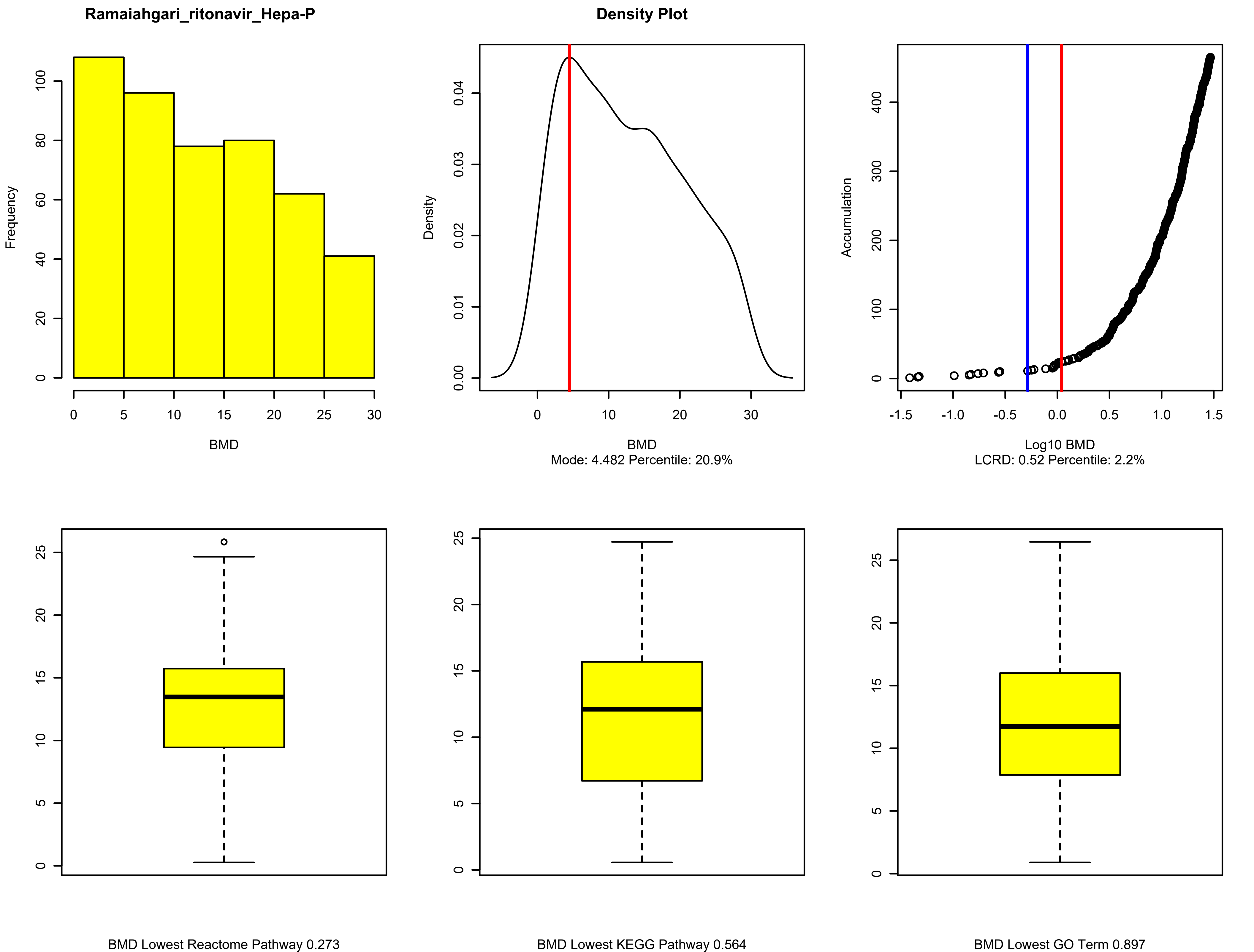
Ramaiahgari_ritonavir_Hepa-P
Frequency
BMD
Density Plot
Density
BMD
Mode: 4.482 Percentile: 20.9%
Accumulation
Log10 BMD
LCRD: 0.52 Percentile: 2.2%
BMD Lowest Reactome Pathway 0.273
BMD Lowest KEGG Pathway 0.564
BMD Lowest GO Term 0.897

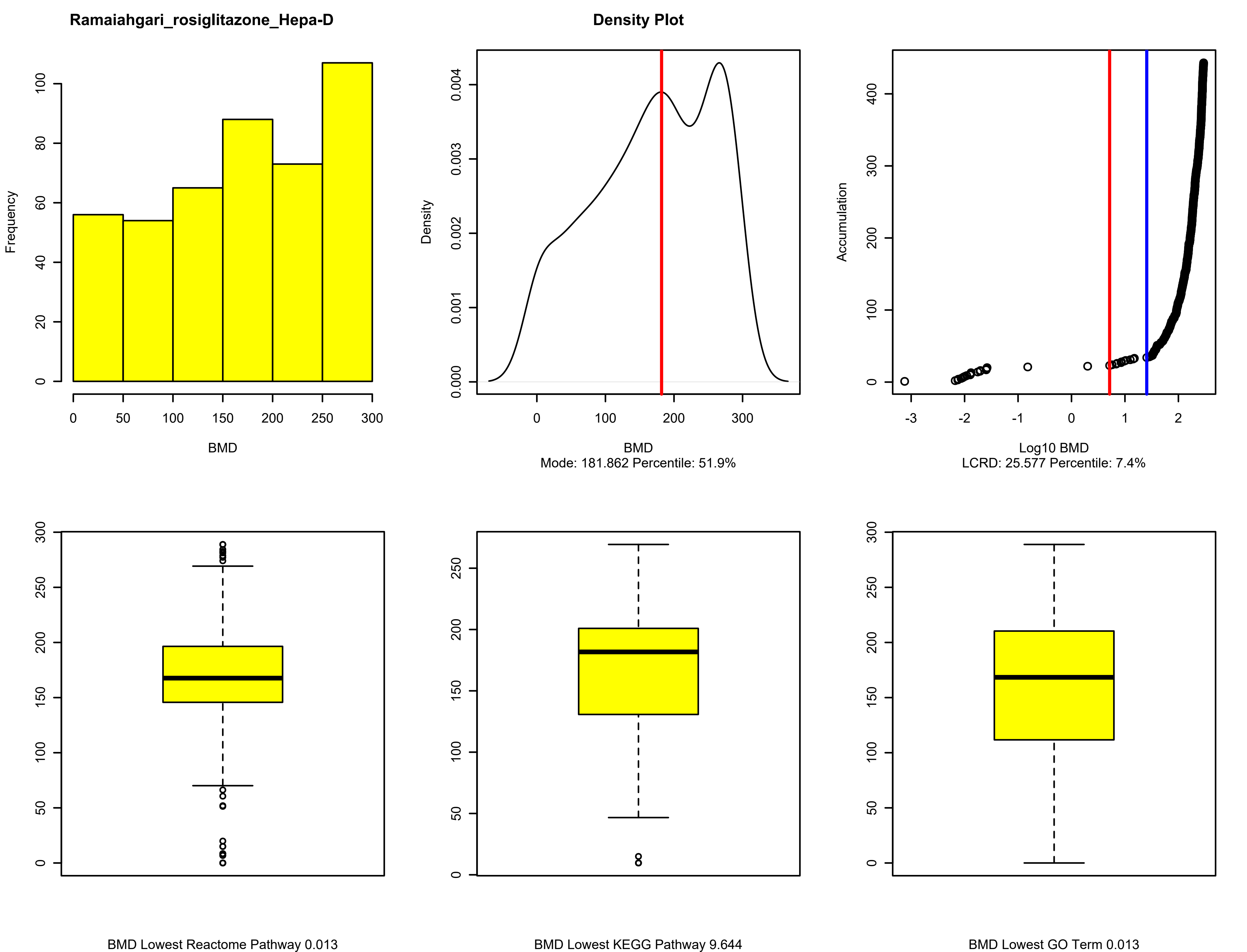
Ramaiahgari_rosiglitazone_Hepa-D
Density Plot
Frequency
BMD
Density
BMD
Mode: 181.862 Percentile: 51.9%
Accumulation
Log10 BMD
LCRD: 25.577 Percentile: 7.4%
BMD Lowest Reactome Pathway 0.013
BMD Lowest KEGG Pathway 9.644
BMD Lowest GO Term 0.013

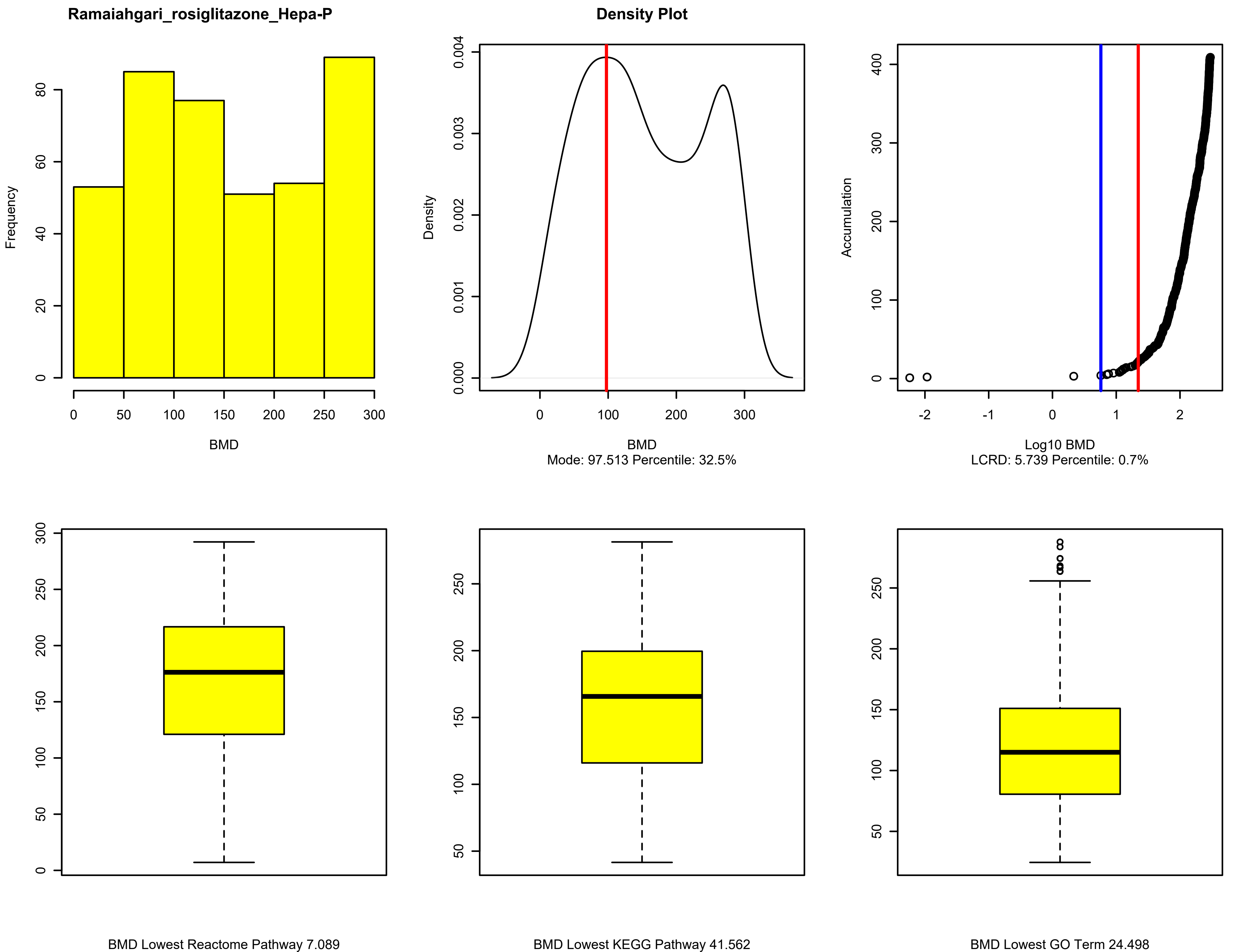

Ramaiahgari_rosiglitazone_Hepa-P
Frequency
BMD
Density Plot
Density
BMD
Mode: 97.513 Percentile: 32.5%
Accumulation
Log10 BMD
LCRD: 5.739 Percentile: 0.7%
BMD Lowest Reactome Pathway 7.089
BMD Lowest KEGG Pathway 41.562
BMD Lowest GO Term 24.498

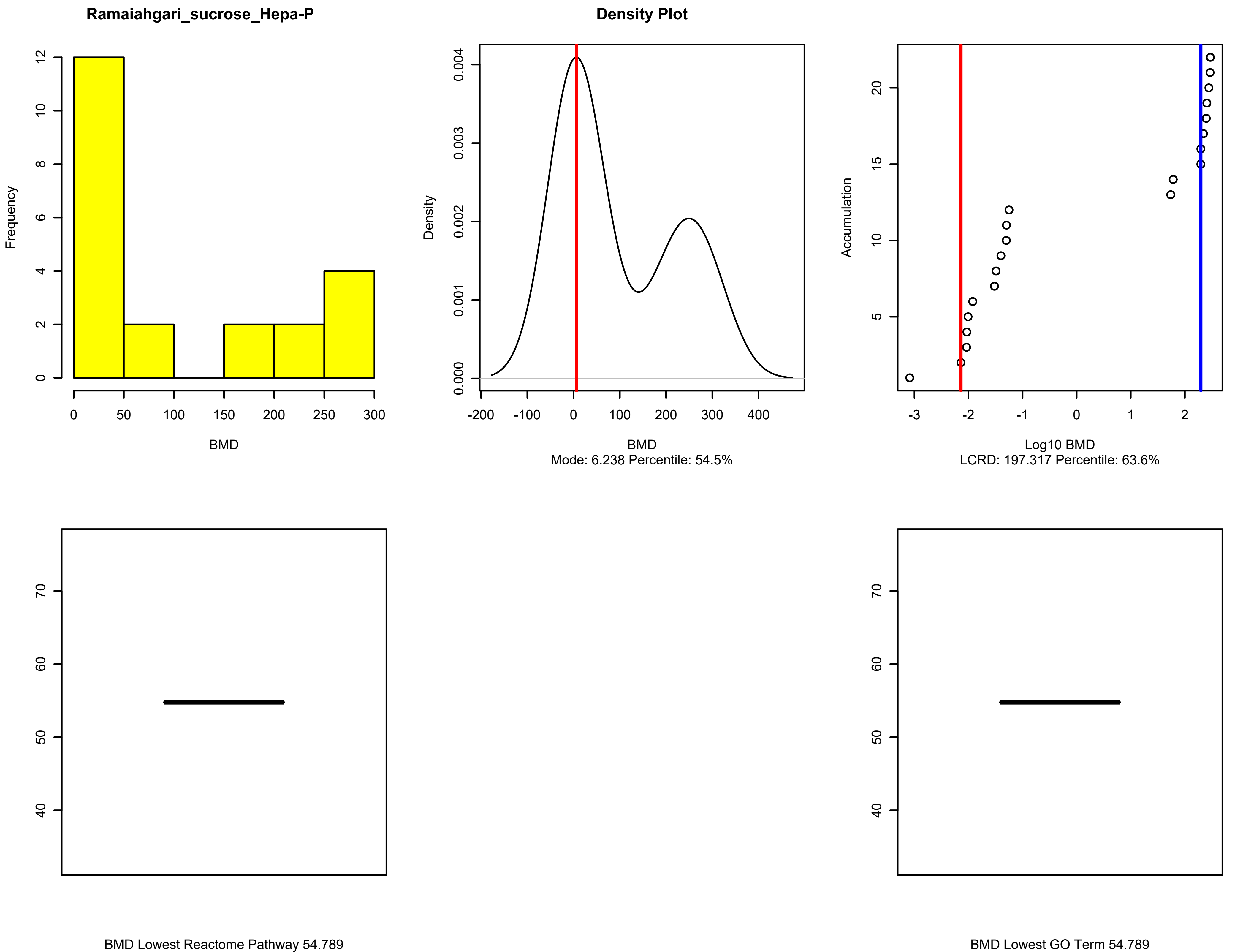

Ramaiahgari_sucrose_Hepa-P
Frequency
BMD
Density Plot
Density
BMD
Mode: 6.238 Percentile: 54.5%
Accumulation
Log10 BMD
LCRD: 197.317 Percentile: 63.6%
BMD Lowest Reactome Pathway 54.789
BMD Lowest GO Term 54.789

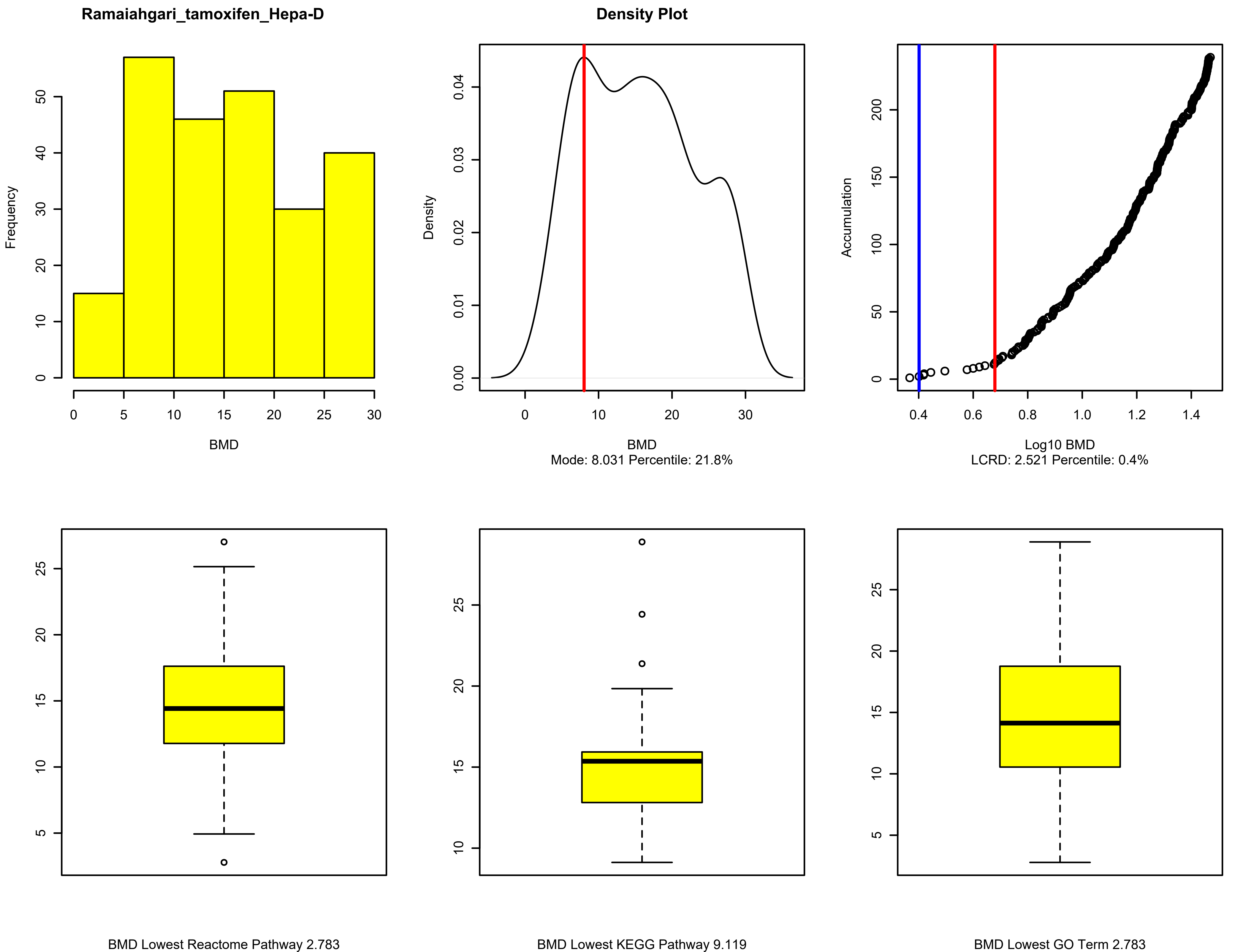

Ramaiahgari_tamoxifen_Hepa-D
Frequency
BMD
Density Plot
Density
BMD
Mode: 8.031 Percentile: 21.8%
Accumulation
Log10 BMD
LCRD: 2.521 Percentile: 0.4%
BMD Lowest Reactome Pathway 2.783
BMD Lowest KEGG Pathway 9.119
BMD Lowest GO Term 2.783

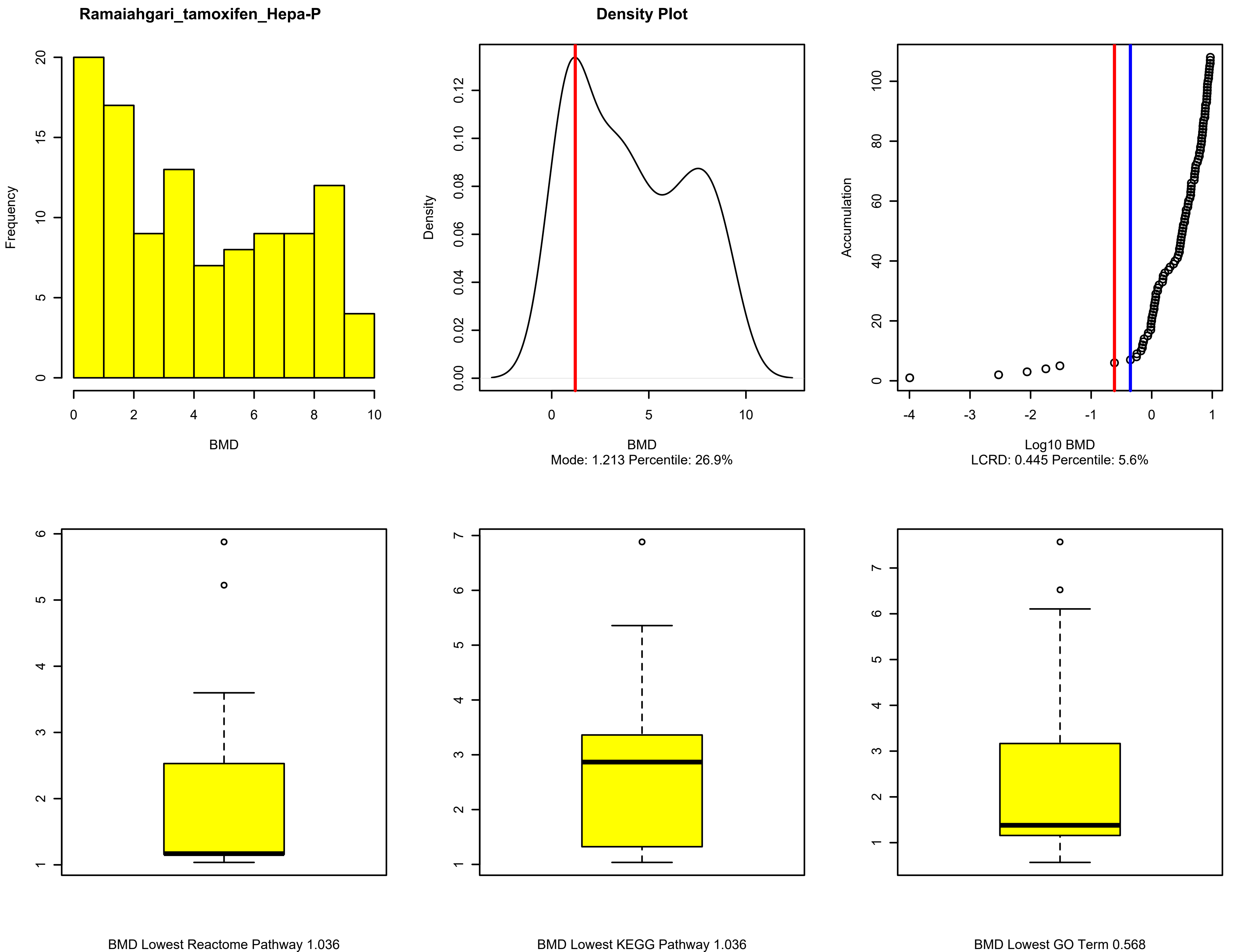
Ramaiahgari_tamoxifen_Hepa-P
Frequency
BMD
Density Plot
Density
BMD
Mode: 1.213 Percentile: 26.9%
Accumulation
Log10 BMD
LCRD: 0.445 Percentile: 5.6%
BMD Lowest Reactome Pathway 1.036
BMD Lowest KEGG Pathway 1.036
BMD Lowest GO Term 0.568

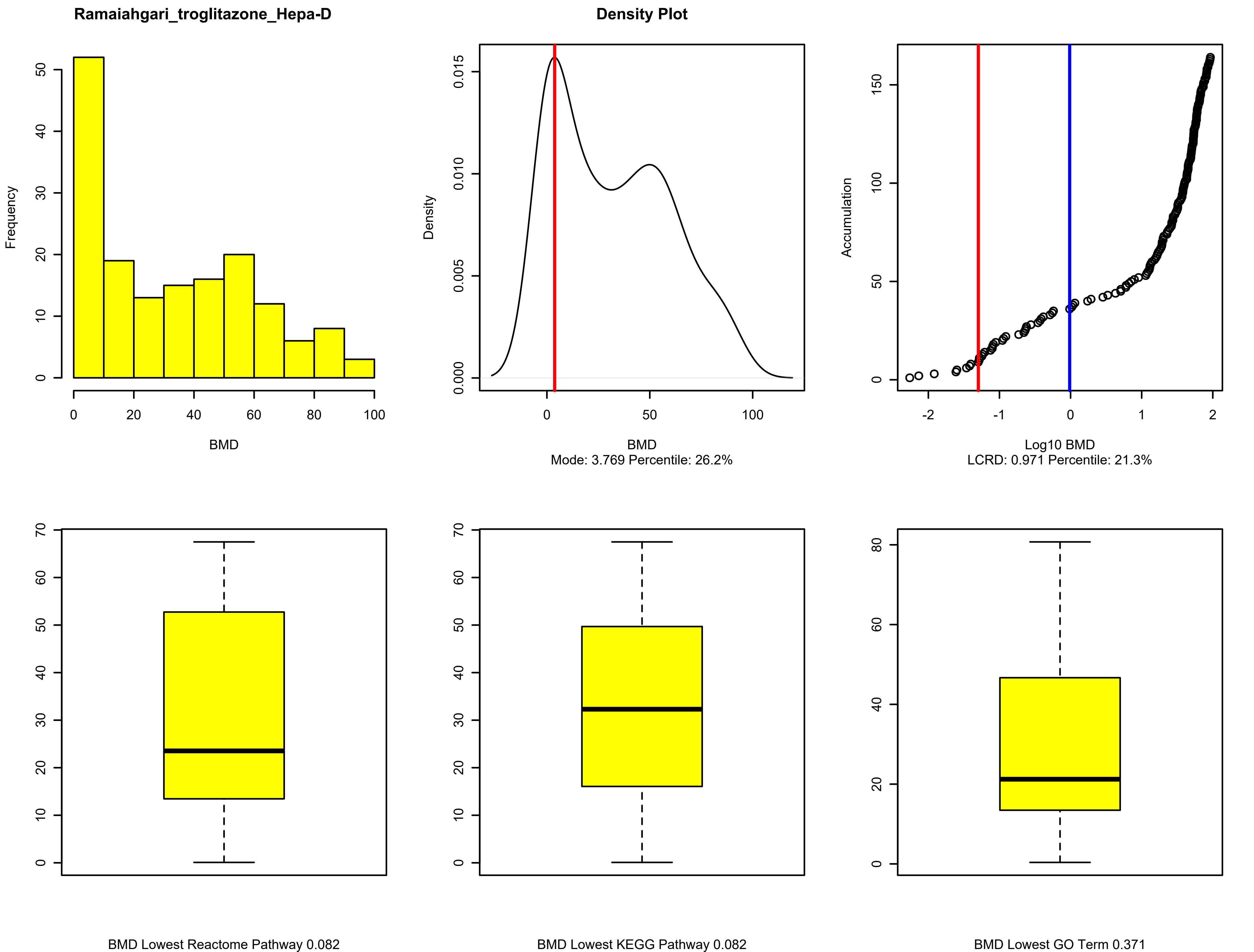

Ramaiahgari_troglitazone_Hepa-D
Frequency
BMD
Density Plot
Density
BMD
Mode: 3.769 Percentile: 26.2%
Accumulation
Log10 BMD
LCRD: 0.971 Percentile: 21.3%
BMD Lowest Reactome Pathway 0.082
BMD Lowest KEGG Pathway 0.082
BMD Lowest GO Term 0.371

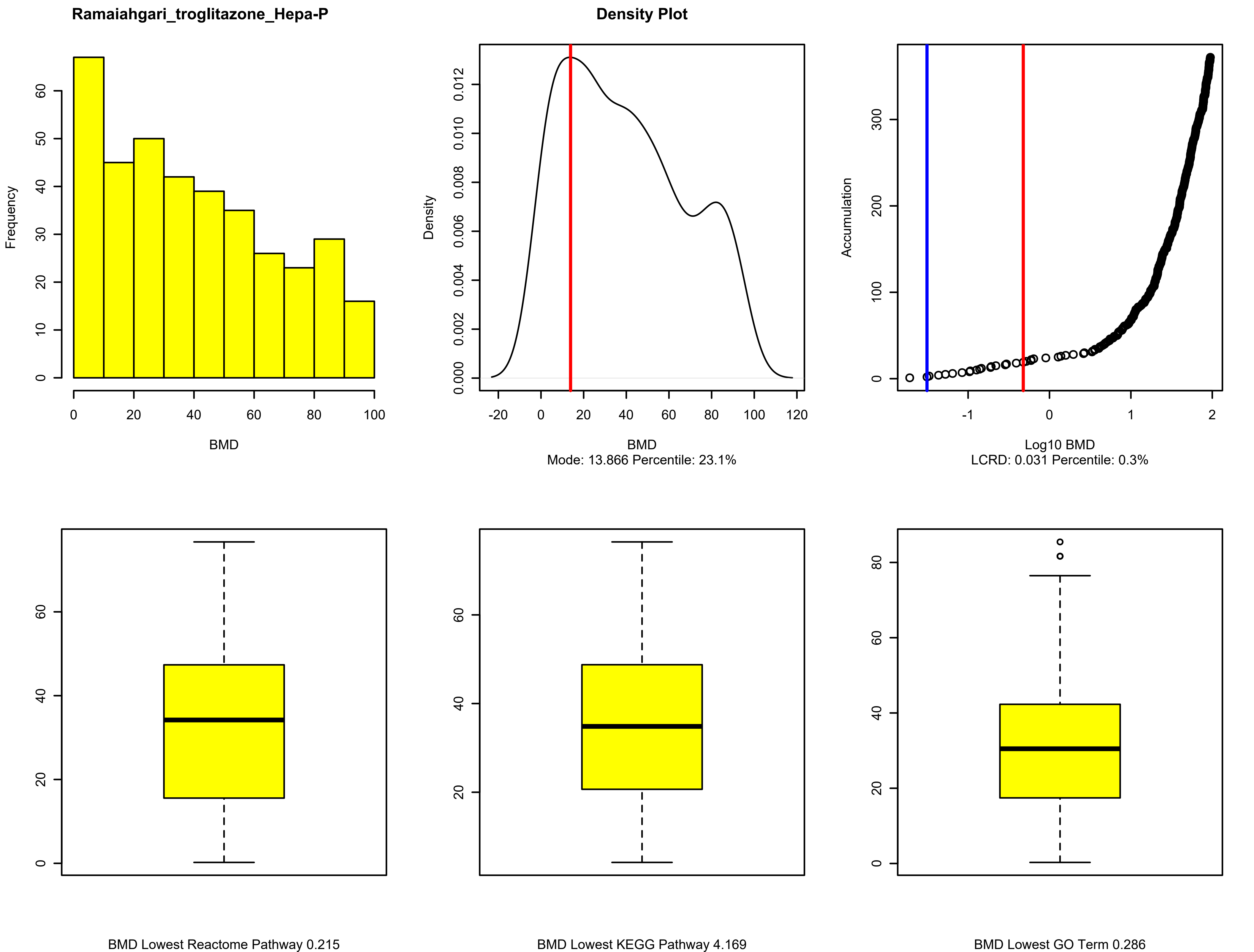
Ramaiahgari_troglitazone_Hepa-P
Frequency
BMD
Density Plot
Density
BMD
Mode: 13.866 Percentile: 23.1%
Accumulation
Log10 BMD
LCRD: 0.031 Percentile: 0.3%
BMD Lowest Reactome Pathway 0.215
BMD Lowest KEGG Pathway 4.169
BMD Lowest GO Term 0.286

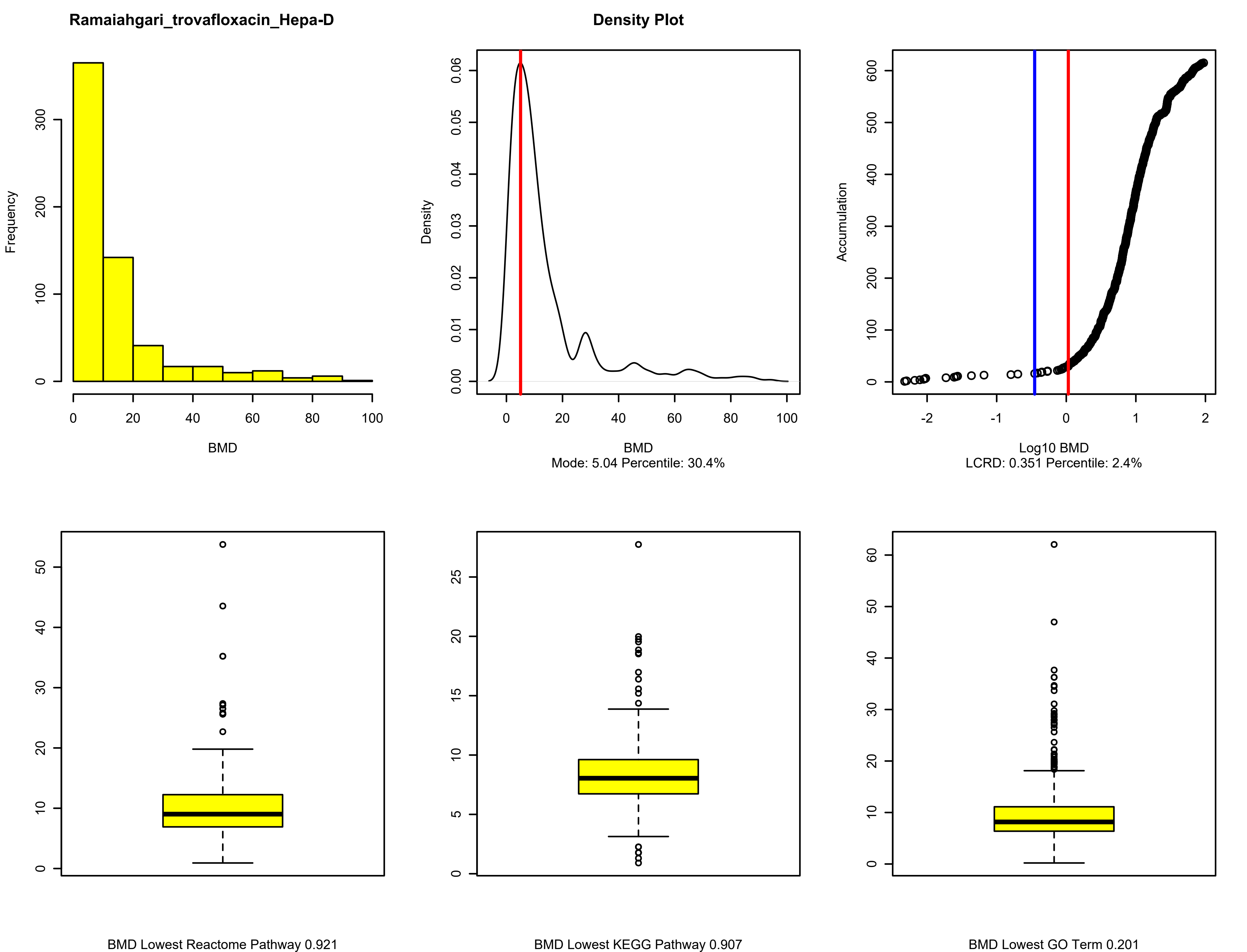
Ramaiahgari_trovafloxacin_Hepa-D
Density Plot
Frequency
BMD
Density
BMD
Mode: 5.04 Percentile: 30.4%
Accumulation
Log10 BMD
LCRD: 0.351 Percentile: 2.4%
BMD Lowest Reactome Pathway 0.921
BMD Lowest KEGG Pathway 0.907
BMD Lowest GO Term 0.201

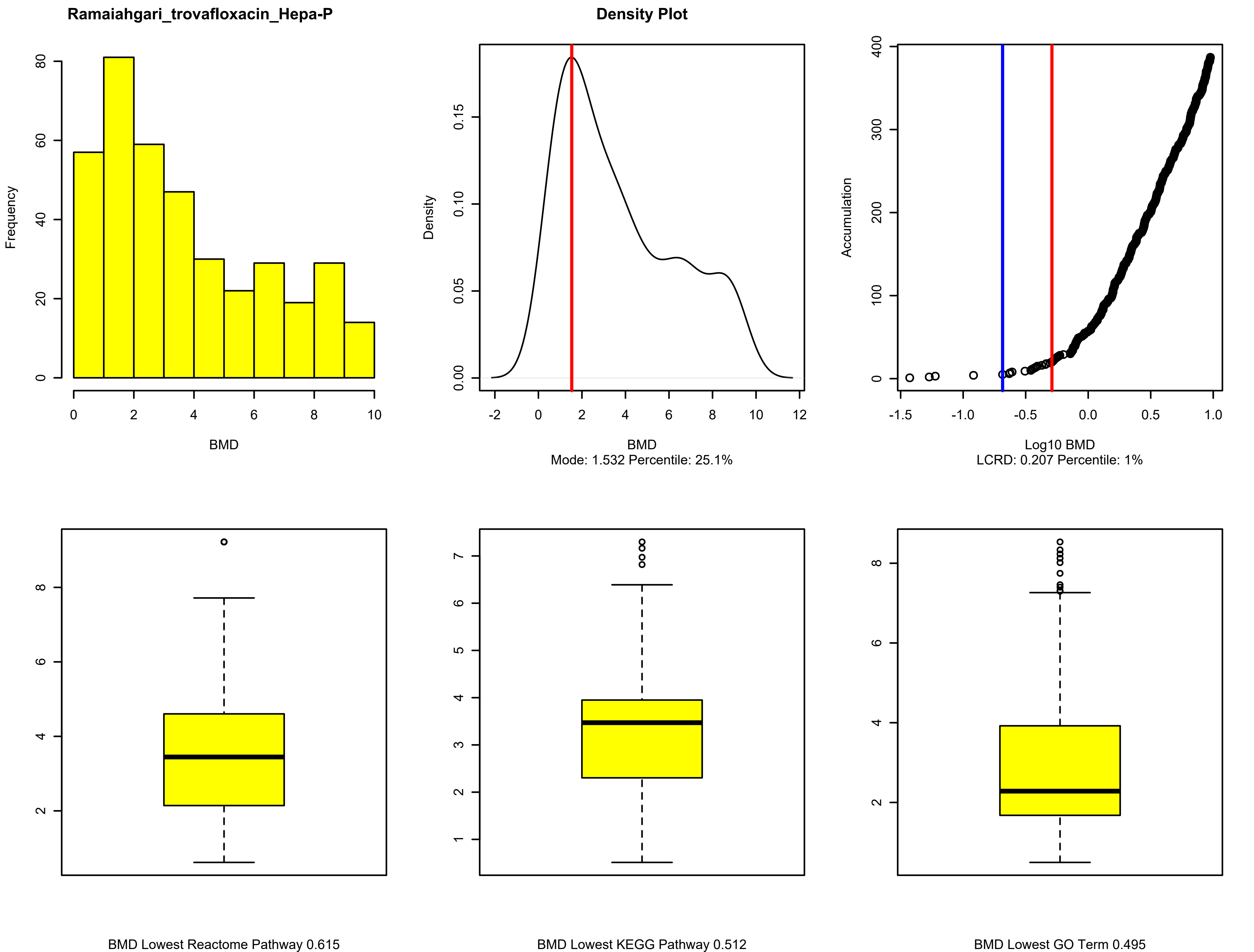
Ramaiahgari_trovafloxacin_Hepa-P
Frequency
BMD
Density Plot
Density
BMD
Mode: 1.532 Percentile: 25.1%
Accumulation
Log10 BMD
LCRD: 0.207 Percentile: 1%
BMD Lowest Reactome Pathway 0.615
BMD Lowest KEGG Pathway 0.512
BMD Lowest GO Term 0.495

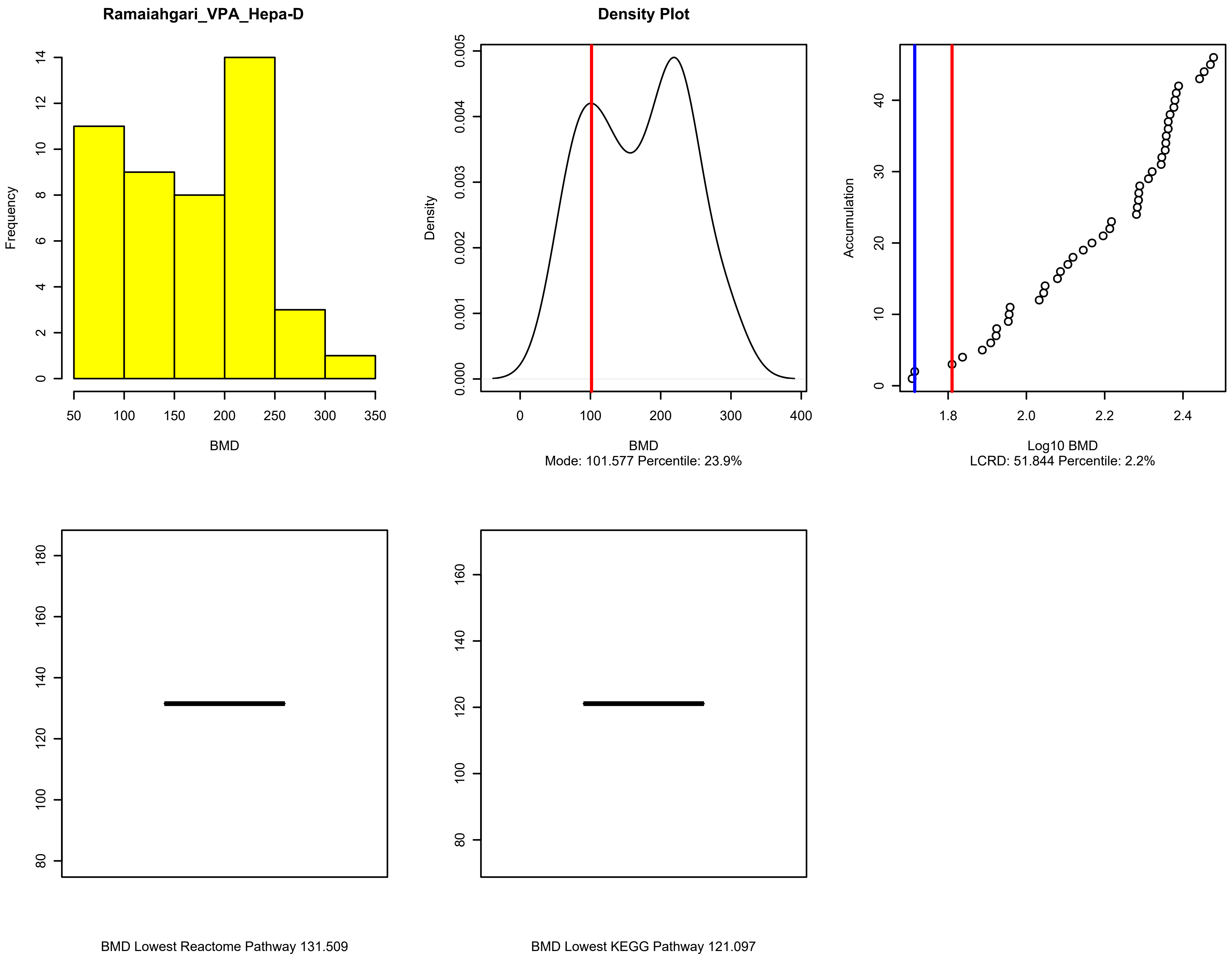
Ramaiahgari_VPA_Hepa-D
Frequency
BMD
Density Plot
Density
BMD
Mode: 101.577 Percentile: 23.9%
Accumulation
Log10 BMD
LCRD: 51.844 Percentile: 2.2%
BMD Lowest Reactome Pathway 131.509
BMD Lowest KEGG Pathway 121.097

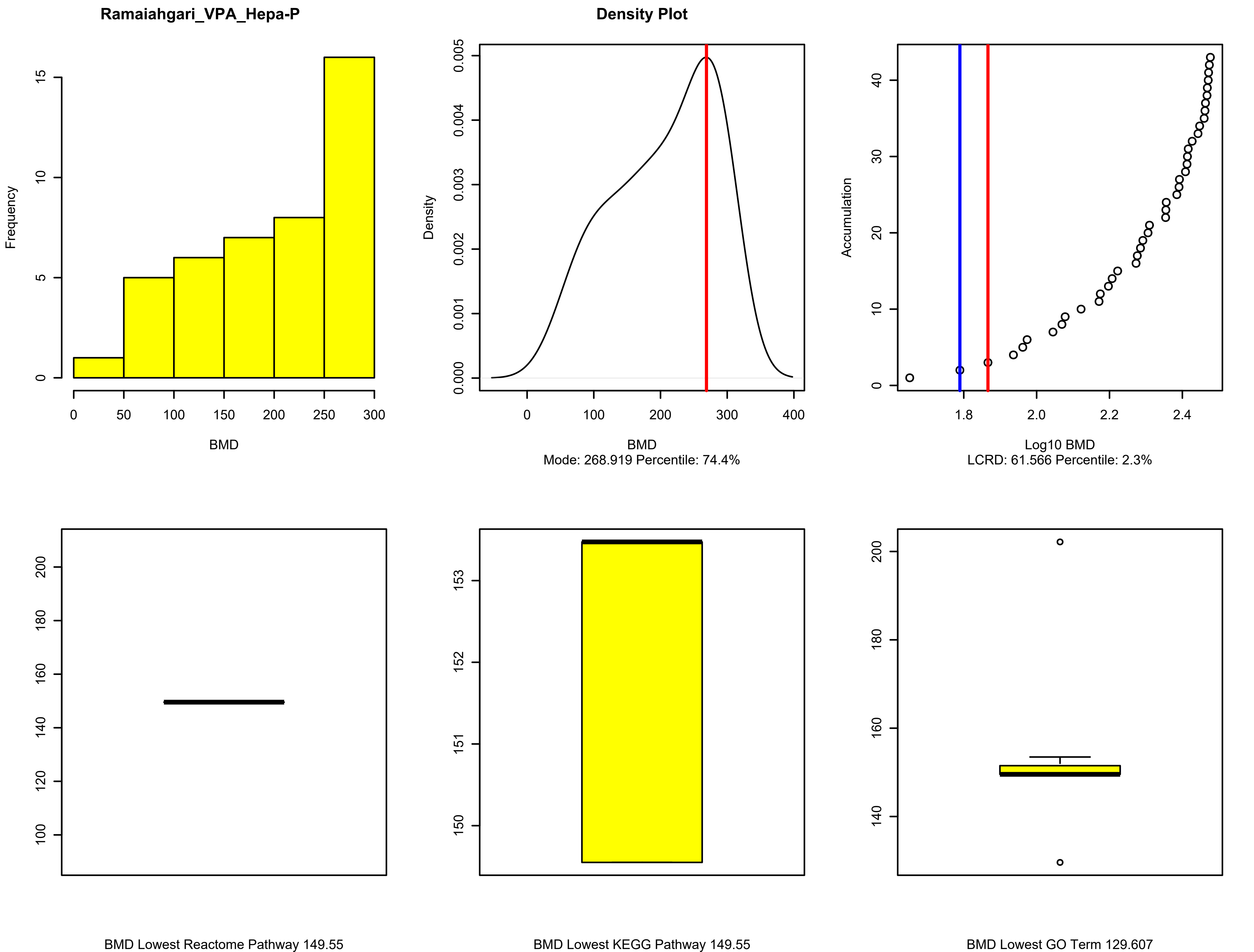

Ramaiahgari_VPA_Hepa-P
Frequency
0
5
10
15
0
50
100
150
200
250
300
BMD
Density Plot
Density
0.000
0.001
0.002
0.003
0.004
0.005
0
100
200
300
400
BMD
Mode: 268.919 Percentile: 74.4%
Accumulation
0
10
20
30
40
1.8
2.0
2.2
2.4
Log10 BMD
LCRD: 61.566 Percentile: 2.3%
100
120
140
160
180
200
BMD Lowest Reactome Pathway 149.55
150
151
152
153
BMD Lowest KEGG Pathway 149.55
140
160
180
200
BMD Lowest GO Term 129.607
