# Supplementary material for: From vision toward best practices: Evaluating in vitro transcriptomic points of departure for application in risk assessment using a uniform workflow
Source: Front Toxicol. 2023 May 23;5:1194895. doi: 10.3389/ftox.2023.1194895 (PMC10242042; doi:10.3389/ftox.2023.1194895)
Supplement: Supplementary file 2 [file DataSheet1.ZIP › Reardon_Supporting Information/SuppIementary Information.docx]

**Supplementary Information**

Evaluating in vitro transcriptomic points of departure for application in human health risk assessment

***Annex A****: Summary details on outliers chemicals comparing derived in vitro AEDs to apical PODs from a search of in vivo databases*

**Atrazine**

Atrazine exposure is associated with fetal developmental delays, endocrine disruption, and mammary tumors (Silva and Iyer, 2014). The derived AED obtained from previous experiments with MCF-7 cells exposed to atrazine for 6 hours; using the current approach from the BMC distribution level, the AED of 0.055 mg/kg-bw/day was derived using the 5^th^ percentile from 62 genes with BMCs, whereas a BMC derived using gene sets was not available. This was comparatively higher than a NOEL of 0.001 mg/kg-bw/day in a neurobehavioral development study of exposed mice (Belloni *et al.*, 2011). In contrast, a recent draft human health risk assessment conducted by the US EPA as part of the registration review established NOAEL values in chronic and reproductive/developmental studies that ranged between 3.3 and 43 mg/kg-bw/day (US EPA, 2018). Although the AED value was lower compared to apical PODs in the draft risk assessment, the increased effect level compared to Belloni *et al*. was combined with the weight of evidence with the other triazine substances and flagged as an outlier using the current workflow.

**Cyanazine**

Cyanazine belongs to this group of [triazines](https://en.wikipedia.org/wiki/Triazine). The derived AED was obtained from MCF-7 cells that were exposed for 6 hours (Harrill *et al.*, 2021). The BMC distribution level AED derived using the 1^st^ mode from 1183 genes with BMCs was 0.23 mg/kg-bw/day, while the gene set level AED was 0.46 mg/kg-bw/day. The lowest available in vivo apical POD collected was a NOAEL of 0.005 mg/kg-bw/day in a 13-week repeat oral dosing study, a value that is 74-fold lower than the derived AED using transcriptomic data and dosing with cyanazine also indicated decreased bodyweight gain (in males) at 0.075 mg/kg-bw/day and above (World Health Organisation, 1998). In contrast to this, the AED value is less than the NOEL of 0.6 mg/kg/day that was used to establish the acceptable daily intake (ADI) for cyanazine by the US EPA (US EPA, 1984). In a review conducted by California EPA, NOEL values for oral chronic, developmental, and reproductive toxicity ranged between 0.15 and 30 mg/kg/day (California EPA, 1997). To maintain consistency in the current workflow, here, the emphasis was on using the lowest/most conservative values available, and cyanazine was identified as an outlier even though the derived AED was within range of the values produced by the California EPA.

**Simazine**

For simazine, the AED was obtained from MCF-7 cells that were exposed for 6 hours (Harrill *et al.*, 2021). The BMC distribution level AED derived using the LCRD from 91 genes with BMCs was 3.25 mg/kg-bw/day, whereas a BMC derived using gene sets was not available. The lowest available POD for simazine was a NOEL of 0.005 mg/kg-bw/day based on the results of decreased body, testicular, and epididymis weight, increased testicular apoptosis, and decreased sperm concentrations observed in male offspring after exposure of females during gestation and lactation (Park and Bae, 2012). Furthermore, the EPA Integrated Risk Information System (IRIS) developed an oral reference dose using the NOAEL of 0.52 mg/kg-bw/day that was determined from a two-year dietary study in rats based on decreased body weight gains and hematologic parameters in females (US EPA, 1993). The lowest calculated AED value of 3.25 obtained using the LCRD metric was approximately 800-fold greater than the NOEL of 0.005 mg/kg-bw/day from a reproductive study in mice (Park and Bae, 2012), and 6.5 fold greater than the NOAEL from the two-year IRIS study (US EPA, 1993).

**Summary statement on triazine herbicides triazine, cyanazine, simazine)**

The triazine chemicals were flagged as outliers in this analysis yielding AEDs that were not lower than apical PODs. These chlorotriazine herbicides (cyanazine, atrazine, and simazine) contain a benzene-like ring with three carbons replaced by nitrogen that target the neuroendocrine regulation of male and female reproductive development (US EPA, 2006; Zorrilla *et al.*, 2010). All three of these chemicals were included within previous work as herbicides inhibiting photosystem II that were not anticipated to be active in within the intended cells of interest (MCF-7), and as a result are outside of the applicability domain for the target cell culture system resulting in non-conservative tPODs and their identification as outliers (Harrill *et al.*, 2021).

**Eugenol**

Eugenol is a colorless to pale yellow, aromatic oily liquid with clove-like scent extracted from certain essential oils. The derived AED from transcriptomic data was obtained from a 2-day in vitro exposure study in differentiated human HepaRG cells with the lowest BMC distribution AED of 54.5 mg/kg-bw/day derived using the LCRD from 567 genes with BMCs and the gene set level AED of 92.7 mg/kg-bw/day (Buick *et al.*, 2021). Previous developmental toxicity studies with rats and rabbits showed the maternal and developmental NOAELs were 100 and 250 mg/kg-bw/day respectively, for both species, without teratogenic effect. Within this context, the EFSA Panel used the maternal NOAEL of 100 mg/kg-bw/day from these developmental studies to establish the Acceptable Daily Intake (ADI) (EFSA Panel on Plant Protection Products and their Residues (PPR), 2012). However, the overall lowest available in vivo POD listed for Eugenol is was a highest no-effect level (HNEL) of 1.9 mg/kg-bw/day derived from human oral study (FDA CSAN COSMOS database retrieved August 5, 2022; Yang *et al.*, 2021). The derived AED using transcriptomic data of 54.5 mg/kg-bw/day was approximately 29-fold greater than this apical POD obtained from the human oral clinical study leading to this chemical being flagged as an outlier. This observation may be attributed to the limited applicability domain of the in vitro models used to derive the tPOD and subsequent AED.

**Cyclophosphamide**

Cyclophosphamide is a genotoxic chemotherapeutic agent that suppresses the immune system. This chemical is carcinogenic in rats and mice (IARC, 1981) and is classified as Category 1A carcinogen (IARC, 2012). The derived AEDs for cyclophosphamide from transcriptomic data were obtained from HepaRG cells exposed to cyclophosphamide for 2 days (Buick *et al.*, 2021). The lowest BMC distribution AED of 40.1 mg/kg-bw/day derived using the LCRD from 394 genes with BMCs and a gene set level AED of 60.6 mg/kg-bw/day. Within the available search criteria, the lowest identified in vivo POD was 0.57 mg/kg-bw/day based on a unitless measure of cancer risk using a human oral clinical study. This in vivo value is 70-fold greater than the lower of the two AEDs derived using transcriptomic data and suggests that the current workflow may not be suitable for cyclophosphamide. However, it should be noted that cyclophosphamide requires metabolic activation, primarily through the liver, in order to induce varying degrees of toxicity (Ayash *et al.*, 1992; Moghe *et al.*, 2015; Groehler *et al.*, 2016). Although HepaRG cells are considered to be more metabolically active compared to simpler in vitro human hepatocyte models (Lübberstedt *et al.*, 2011) they likely do not maintain a high enough activity in conventional 2D static culture models to induce the level of toxicity observed with cyclophosphamide in vivo. A limitation that could be resolved by using a more sophisticated model (e.g., liver spheroids) to provide a more accurate in vitro derived tPOD and subsequent AED.

**Aflatoxin B1 (AFB1)**

AFB1 is a common contaminant in a variety of foods including peanuts, cottonseed meal, corn, and other grains. The derived AEDs from transcriptomic data were obtained from HepaRG cells exposed to AFB1 for 2 days (Buick *et al.*, 2021) and 4 days (Ramaiahgari *et al.*, 2019). The lowest BMC distribution AED of 0.372, and 0.002 mg/kg-bw/day derived using the LCRD and gene set level AEDs of 0.205 and 0.004 mg/kg-bw/day, from 2 and 4 days exposures within each respective study. AFB1 causes acute hepatotoxicity and induces immune suppression. EFSA summarized the results of studies that showed AFB1 could results in impairment of immune system in animals and the derived NOAELs were approximately 0.03 mg/kg-bw/day (EFSA, 2007). However, using human data the lowest available in vivo POD listed for AFB1 was a BMDL_01_ of 0.000078 mg/kg-bw/day and a BMDL_10_ of 0.00087 mg/kg-bw/day derived from human epidemiological data based on incidences of liver cancer, specifically in male carriers of the hepatitis B surface antigen and high background incidence of hepatocellular carcinomas (~10 %) (EFSA, 2007). EFSA also derived MOEs based on the lowest BMDL_10_ value of 0.00017 mg/kg-bw/day calculated based on hepatocarcinogenicity observed in male Fischer rats (EFSA, 2007; Wogan *et al.*, 1974). The calculated lower AED value of 0.002 mg/kg-bw/day derived from the LCRD is approximately 25- and 2.3-fold higher (i.e., less conservative) than the lowest human epidemiological POD, BMDL_01,_ and BMDL_10_, respectively, and 11-fold higher than the lowest oral apical POD value obtained from rodent study. However, AFB1 requires bioactivation in order to enact carcinogenesis and similar to cyclophosphamide, the identification of AFB1 as an outlier may be due the limited metabolic capacity of the target system (HepaRG cells). A limitation that could be resolved by using a more sophisticated model (e.g., liver spheroids) or the addition of a metabolic component (e.g., S9 fraction) to provide a more accurate in vitro derived tPOD and subsequent AED.

**Benzo[a]Pyrene (B[a]P)**

B[a]P is a polycyclic aromatic hydrocarbon commonly formed as a product of incomplete combustion, typically found in coal tar, tobacco smoke, as well as smoked or grilled foods. The derived AEDs from transcriptomic data were obtained from both differentiated and proliferated HepaRG cells exposed to B[a]P for 2 days (Buick *et al.*, 2021) and differentiated cells exposed for 4 days (Ramaiahgari *et al.*, 2019). The lowest BMC distribution value from exposed proliferated cells was 0.87 mg/kg-bw/day derived using the LCRD, and the gene set level AED was 1.36 mg/kg-bw/day (Ramaiahgari *et al.*, 2019). Previous work has shown that there are multiple routes of exposure to B[a]P, and the EPA IRIS program revealed that exposure results in developmental toxicity, reproductive toxicity and to a lesser extent immunotoxicity (US EPA, 2017). The lowest available AED (0.87 mg/kg-bw/day) was ~17-fold greater than the lowest available in vivo POD (0.05 mg/kg-bw/day).

**References:**

Ayash, L.J., Wright, J.E., Tretyakov, O., Gonin, R., Elias, A., Wheeler, C., Eder, J.P., Rosowsky, A., Antman, K., and Frei, E. (1992). Cyclophosphamide pharmacokinetics: Correlation with cardiac toxicity and tumor response. *J. Clin. Oncol.*, **10**, 995–1000.

Belloni, V., Dessì-Fulgheri, F., Zaccaroni, M., Di Consiglio, E., De Angelis, G., Testai, E., Santochirico, M., Alleva, E., and Santucci, D. (2011). Early exposure to low doses of atrazine affects behavior in juvenile and adult CD1 mice. *Toxicology*, **279**, 19–26.

Buick, J.K., Williams, A., Meier, M.J., Swartz, C.D., Recio, L., Gagné, R., Ferguson, S.S., Engelward, B.P., and Yauk, C.L. (2021). A Modern Genotoxicity Testing Paradigm: Integration of the High-Throughput CometChip® and the TGx-DDI Transcriptomic Biomarker in Human HepaRG^TM^ Cell Cultures. *Front. Public Heal.*, **9**, 1–19.

California EPA (1997). Cyanazine - Risk Characterization Document.

EFSA (2007). Opinion of the scientific panel on contaminants in the food chain on a request from the commision related to the potential increase of consumer health risk by a possible increase of the existing maximum levels for aflatoxins in almonds, hazelnuts and pist. *EFSA J.*, **446**, 1–127.

EFSA Panel on Plant Protection Products and their Residues (PPR) (2012). Scientific Opinion on Evaluation of the Toxicological Relevance of Pesticide Metabolites for Dietary Risk Assessment. *EFSA J.*, **10**, 1–187.

Groehler, A., Villalta, P.W., Campbell, C., and Tretyakova, N. (2016). Covalent DNA-Protein Cross-Linking by Phosphoramide Mustard and Nornitrogen Mustard in Human Cells. *Chem. Res. Toxicol.*, **29**, 190–202.

Harrill, J.A., Everett, L.J., Haggard, D.E., Sheffield, T., Bundy, J.L., Willis, C.M., Thomas, R.S., Shah, I., and Judson, R.S. (2021). High-Throughput Transcriptomics Platform for Screening Environmental Chemicals. *Toxicol. Sci.*, **181**, 68–89.

IARC (2012). Monographs on the Evaluation of Carcinogenic Risks to Humans: Pharmaceuticals.

IARC (1981). Monographs on the Evaluation of the Carcinogenic Risk of Chemicals to Humans. Some Antineoplastic and Immunosuppressive Agents.

Lübberstedt, M., Müller-Vieira, U., Mayer, M., Biemel, K.M., Knöspel, F., Knobeloch, D., Nüssler, A.K., Gerlach, J.C., and Zeilinger, K. (2011). HepaRG human hepatic cell line utility as a surrogate for primary human hepatocytes in drug metabolism assessment in vitro. *J. Pharmacol. Toxicol. Methods*, **63**, 59–68.

Moghe, A., Ghare, S., Lamoreau, B., Mohammad, M., Barve, S., McClain, C., and Joshi-Barve, S. (2015). Molecular mechanisms of acrolein toxicity: Relevance to human disease. *Toxicol. Sci.*, **143**, 242–255.

Park, H.O. and Bae, J. (2012). Disturbed Relaxin Signaling Pathway and Testicular Dysfunction in Mouse Offspring upon Maternal Exposure to Simazine. *PLoS One*, **7**.

Ramaiahgari, S.C., Auerbach, S.S., Saddler, T.O., Rice, J.R., Dunlap, P.E., Sipes, N.S., Devito, M.J., Shah, R.R., Bushel, P.R., Merrick, B.A., *et al.* (2019). The power of resolution: Contextualized understanding of biological responses to liver injury chemicals using high-throughput transcriptomics and benchmark concentration modeling. *Toxicol. Sci.*, **169**, 553–566.

Silva, M. and Iyer, P. (2014). Toxicity endpoint selections for a simazine risk assessment. *Birth Defects Res. Part B - Dev. Reprod. Toxicol.*, **101**, 308–324.

US EPA (2018). Atrazine. Draft Human Health Risk Assessment for Registration Review.

US EPA (2006). Decision Documents for Atrazine.

US EPA (1984). Health And Environmental Effects Profile for Cyanazine Washington, DC.

US EPA (2017). IRIS Toxicological Review of Benzo[A]Pyrene (Final Report) Washington, DC,.

US EPA (1993). Simazine - IRIS Chemical Assessment Summary.

Wogan, G.N., Paglialunga, S., and Newberne, P.. (1974). Effects of Low Dietary. *Food Cosmet. Toxicol.*, **12**, 681–685.

World Health Organisation (1998). Cyanazine in Drinking-water.

Yang, C., Cronin, M.T.D., Arvidson, K.B., Bienfait, B., Enoch, S.J., Heldreth, B., Hobocienski, B., Muldoon-Jacobs, K., Lan, Y., Madden, J.C., *et al.* (2021). COSMOS next generation – A public knowledge base leveraging chemical and biological data to support the regulatory assessment of chemicals. *Comput. Toxicol.*, **19**, 100175.

Zorrilla, L.M., Gibson, E.K., and Stoker, T.E. (2010). The effects of simazine, a chlorotriazine herbicide, on pubertal development in the female Wistar rat. *Reprod. Toxicol.*, **29**, 393–400.
